# B(C_6_F_5_)_3_-Catalyzed Dehydrogenation of Pyrrolidines to Form Pyrroles

**DOI:** 10.1021/acscatal.3c05444

**Published:** 2024-03-18

**Authors:** Ana Alvarez-Montoya, Joseph P. Gillions, Laura Winfrey, Rebecca R. Hawker, Kuldip Singh, Fabrizio Ortu, Yukang Fu, Yang Li, Alexander P. Pulis

**Affiliations:** † School of Chemistry, University of Leicester, Leicester LE1 7RH, U.K.; ‡ School of Chemical Engineering, Dalian University of Technology, No. 2 Linggong Road, Dalian 116024, P. R. China

**Keywords:** dehydrogenation, pyrrolidines, pyrroles, *N*-heterocycles, catalysis, boranes

## Abstract

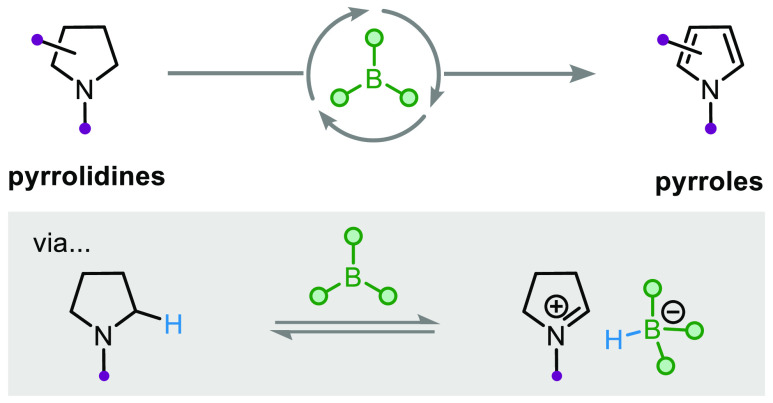

Pyrroles are important *N*-heterocycles found in medicines and materials. The formation of pyrroles from widely accessible pyrrolidines is a potentially attractive strategy but is an underdeveloped approach due to the sensitivity of pyrroles to the oxidative conditions required to achieve such a transformation. Herein, we report a catalytic approach that employs commercially available B(C_6_F_5_)_3_ in an operationally simple procedure that allows pyrrolidines to serve as direct synthons for pyrroles. Mechanistic studies have revealed insights into borane-catalyzed dehydrogenative processes.

## Introduction

**Scheme 1:**
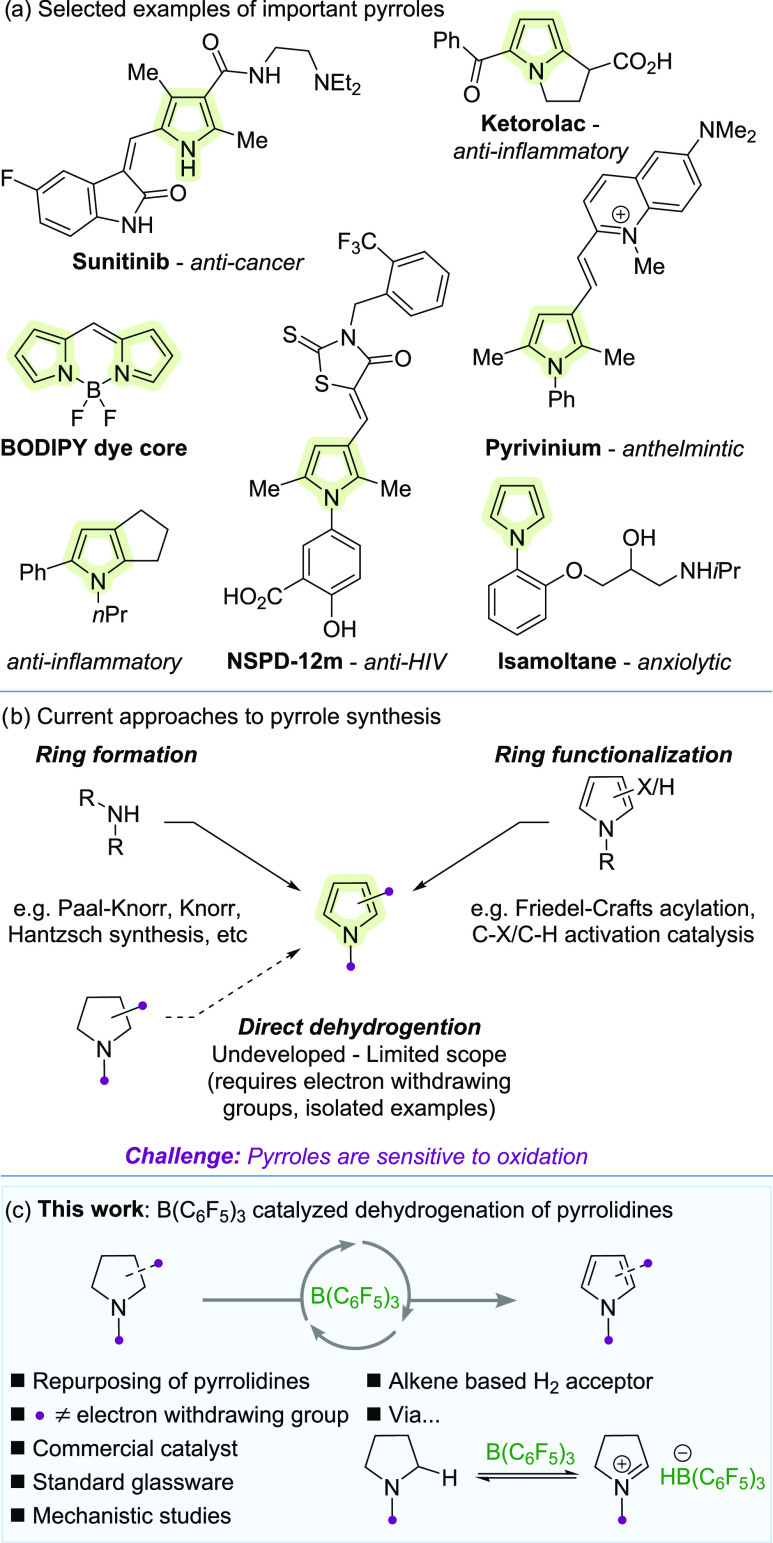
Dehydrogenation of Pyrrolidines

Pyrroles are important structural motifs^1^ that, for example, appear in FDA-approved drugs^2−4^ and have antitumor,^2^ anti-inflammatory,^3,5^ anthelmintic,^4^ anti-HIV,^6^ and anxiolytic^7^ properties (Scheme 1a). Pyrroles are also found in materials such as dyes,^8^ semiconductors,^9^ and sensors,^10^ showcasing the versatility of the scaffold.

The synthesis of pyrroles typically involves ring formation or functionalization of an existing pyrrole (Scheme 1b).^11^ A potentially complementary approach forms pyrroles directly via the dehydrogenation of pyrrolidines. Pyrrolidines are among the most commonly encountered saturated *N*-heterocycles^12^ and are readily accessed via well-established synthetic methods (e.g., Buchwald–Hartwig, Chan–Lam, Ullmann couplings, and annulation via bis-alkylations).^13^ In addition, the advantages of using pyrrolidines as synthetic equivalents to pyrroles lie in their orthogonal reactivity. While pyrroles are sensitive to acids,^14^ oxidants,^15^ and react with electrophiles through the carbon skeleton,^16^ pyrrolidines do not. Therefore, a pyrrolidine may be more robust and able to be carried through a synthetic sequence for a pyrrole to be revealed at a later stage. While the dehydrogenation of most saturated (and partially saturated) *N*-heterocycles is relatively straightforward,^17^ particularly those that are benzo-fused, the dehydrogenation of pyrrolidines is more challenging as pyrroles are sensitive to oxidizing conditions.^15^ Despite the potential utility, the few methods for the direct dehydrogenation of pyrrolidines are generally limited to forming pyrroles with electron-withdrawing groups and/or are reported as isolated cases.^18−24^ Stoichiometric reagents such as MnO_2_,^18^ and DDQ^19^ have been used to dehydrogenate pyrrolidines that are substituted with electron-withdrawing groups (typically carboxylic acid derivatives).^20^ Hu reported the formation of di- and tricarboxyl substituted pyrroles using Cu/TEMPO catalyst and O_2_.^21^ Brayton dehydrogenated unfunctionalized perhydroindoles and perhydrocarbazoles using an iridium pincer catalyst.^22^ Other catalytic approaches have been reported as isolated examples.^23^ For example, Konig discovered an iridium–nickel dual photocatalytic dehydrogenation of 1-(*tert*-butyl)2-methyl pyrrolidine-1,2-dicarboxylate.^23a^

Herein, we report a catalytic approach for the direct dehydrogenation of pyrrolidines, where new classes of pyrrolidines serve as direct synthons for pyrroles for the first time (Scheme 1c). Importantly, the method does not require pyrrolidines to be substituted with electron-withdrawing groups. We employ commercially available B(C_6_F_5_)_3_ used as received from the supplier, weighed in air, and a glovebox is not required. Mechanistic investigations have revealed that the reaction proceeds via initial borane-mediated α-nitrogen hydride abstraction over a low energy barrier and that later hydride abstraction can occur at either an α-nitrogen or unusually at the γ-nitrogen positions of dihydropyrrole intermediates. The structure of key intermediates and the crucial role of the alkene-based hydrogen acceptor have also been demonstrated.

## Results and Discussion

### Optimization

We began by considering if the ability of B(C_6_F_5_)_3_ to oxidize amines via α-nitrogen hydride abstraction^25^ could be used for the catalytic dehydrogenation of pyrrolidines. Recently, Grimme and Paradies,^26^ Kanai,^27^ and Ogoshi and Hoshimoto^28^ reported the B(C_6_F_5_)_3_-catalyzed dehydrogenation of various benzo-fused *N*-heterocycles, such as indolines^29^ and 1,2,3,4-tetrahydroquinolines, but pyrrolidines were absent from the scope in all cases. B(C_6_F_5_)_3_ also catalyzes the dehydrogenative β-functionalization of amines.^30^ Notably, Yang and Ma reported a B(C_6_F_5_)_3_-catalyzed β-functionalization of pyrrolidines with isatins and subsequent dehydrogenation of the intermediate exocyclic alkenyl products (tautomeric equivalents to a dihydropyrrole).^30a^ However, the B(C_6_F_5_)_3_-catalyzed direct dehydrogenation of pyrrolidines has not been reported. Furthermore, the challenges of forming pyrroles from pyrrolidines in a B(C_6_F_5_)_3_-catalyzed process is highlighted by Resconi and co-workers where pyrroles are reported to react directly with B(C_6_F_5_)_3_ in an S_E_Ar manner to form boronate products, providing a potential route to catalyst poisoning.^31^

**Scheme 2:**
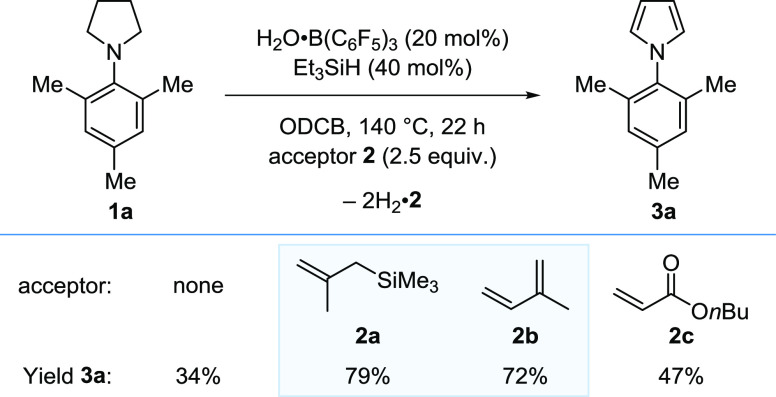
Optimization of B(C_6_F_5_)_3_-Catalyzed Dehydrogenation of Pyrrolidines Reaction performed with **1a** (0.2 mmol). Yields determined by ^1^H NMR analysis of the crude reaction mixtures with an internal standard. equiv = equivalents. H_2_·**2** = hydrogenated acceptor **2**.

Despite this unfavorable precedence, we started with the dehydrogenation of *N*-mesityl pyrrolidine (**1a**) in an acceptorless approach (Scheme 2). We were pleased to obtain an initial hit with the formation of pyrrole **3a** in 34% yield using B(C_6_F_5_)_3_ (20 mol %) and 1,2-dichlorobenzene (ODCB) as the solvent. Despite extensive optimization,^32^ including varying solvents and catalytic additives, we were unable to improve the yield.

We then investigated an acceptor-based approach (see below for mechanistic discussion). A variety of alkenes **2** were efficient acceptors in the B(C_6_F_5_)_3_-catalyzed dehydrogenation of pyrrolidines **1**.^32^ In particular, commercially available trimethyl(2-methylallyl)silane (**2a**) and isoprene (**2b**) allowed the formation of pyrrole **3a** in 79 and 72% yield, respectively.

It should be noted that B(C_6_F_5_)_3_ is often supplied as H_2_O·B(C_6_F_5_)_3_ requiring purification before use and is normally handled in its water-free form inside an inert atmosphere glovebox. With a view to operational simplicity and accessibility, our procedure uses commercially available B(C_6_F_5_)_3_ as supplied, which is weighed in air on the open bench. We use commercially available Et_3_SiH [2 equiv, relative to B(C_6_F_5_)_3_] to free B(C_6_F_5_)_3_ from its water adduct *in situ*,^33,34^ and therefore standard glassware and syringe septa techniques can be used.

### Reaction Scope

**Scheme 3:**
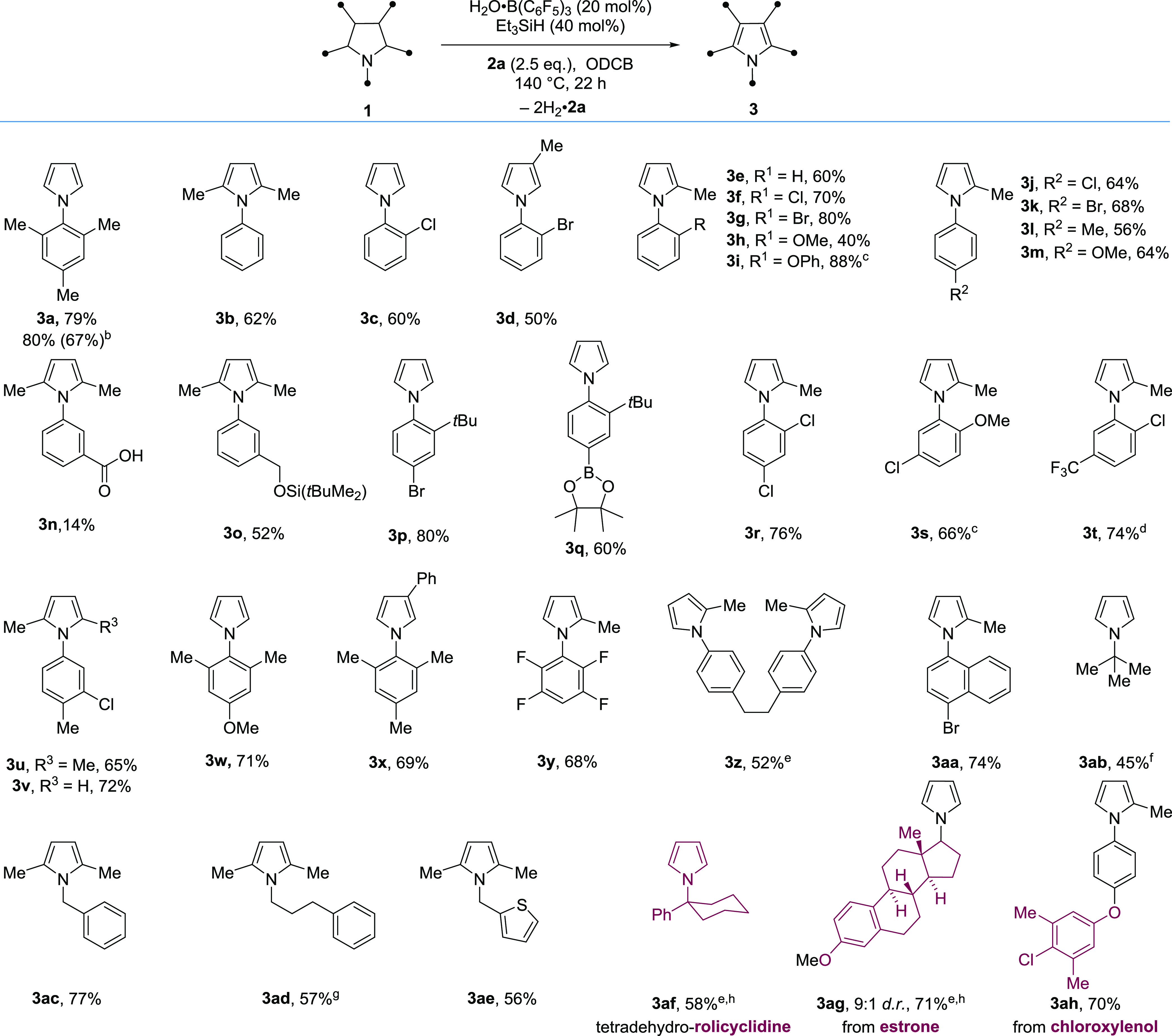
Scope of the B(C_6_F_5_)_3_-Catalyzed Dehydrogenation of Pyrrolidines Reaction performed with **1** (0.2 mmol). Yields are determined by ^1^H NMR analysis of the crude reaction mixtures with an internal standard. All compounds were isolated to confirm identity. Using **1a** (3 mmol), isolated yield in parentheses. Using DCE as the solvent at 85 °C. Using *p*-xylene as the solvent at 120 °C. Using **2a** (5 equiv). Reaction performed over 46 h. Using purified (water-free) B(C_6_F_5_)_3_ (20 mol %). Using B(C_6_F_5_)_3_ (40 mol %) and Et_3_SiH (80 mol %). H_2_·**2a** = isobutyltrimethylsilane.

We evaluated the scope of the reaction using acceptor **2a**(34) and found that a variety of *N*-aryl and *N*-alkyl pyrrolidines were successfully dehydrogenated using the B(C_6_F_5_)_3_-catalyzed approach (Scheme 3). Pyrrolidines bearing 1-, 1,2-, 1,3- and 1,2,5-substitution were tolerated. Pyrroles containing a variety of important groups, including bromo (**3d**, **3g**, **3k**, **3p**, **3aa**), chloro (**3c**, **3f**, **3j**, **3r**, **3s**, **3t**-**v**), ethers (**3h**, **3i**, **3m**, **3s**, **3w**), silyl ether (**3o**), trifluoromethyl (**3t**), tetrafluorophenyl (**3y**), thiophene (**3ae**), and pinacol boronic ester (**3q**), were successfully formed. The pyrroles formed bearing halides and boronic ester functionality are valuable synthetic intermediates as both can engage in palladium-catalyzed cross-coupling.^35^ Carboxylic acid containing pyrrole **3n** was synthesized, albeit in a lower yield (14%). Interestingly, bis-pyrrole **3z** was formed via the formal removal of four H_2_ molecules. Substrates bearing *ortho*-aryl substitution were also compatible and did not interfere with the critical B(C_6_F_5_)_3_ α-nitrogen hydride abstraction. These included the *ortho*-*tert*-butyl substituted pyrrolidines (*cf*. **3p** and **3q**) that are advanced intermediates of the FDA-approved acne treatment, trifarotene.^36^

*N*-Alkyl-substituted pyrrolidines are more challenging substrates, as they bear a more basic nitrogen (that may potentially poison the catalyst) as well as offer an alternative site for unproductive α-nitrogen hydride abstraction (see the mechanistic discussion below). Despite this, *N*-alkyl pyrroles (**3ab-ae**) were successfully formed from *N*-alkyl pyrrolidines.

Pyrrole **3a** was also prepared on a 3 mmol scale and isolated in 67% yield. In addition, we applied the methodology to the dehydrogenation of anesthetic rolicyclidine to form tetradehydro-rolicyclidine **3af** (58%) and the formation of estrone derivative **3ag** (71%) and **3ah** (70%), a derivative of the antiseptic chloroxylenol (Scheme 3).

**Scheme 4:**
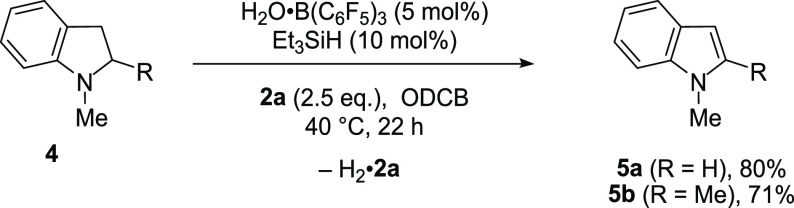
B(C_6_F_5_)_3_-Catalyzed Dehydrogenation of Indolines Reaction performed with **1** (0.2 mmol). Yields are determined by ^1^H NMR analysis of the crude reaction mixtures with an internal standard.

There are several methods for the conversion of indolines to indoles,^37^ but elevated temperatures are typically required. For example, Grimme and Paradies reported the B(C_6_F_5_)_3_ (5 mol %)-catalyzed acceptorless dehydrogenation of a variety of indolines in high yield at 120 °C.^26^ Using our acceptor-based approach, we have developed a complementary method and found that indoles **5** were efficiently formed under mild conditions (40 °C) using 5 mol % of B(C_6_F_5_)_3_ without the need for a glovebox (Scheme 4).

### Mechanistic Investigations

**Scheme 5:**
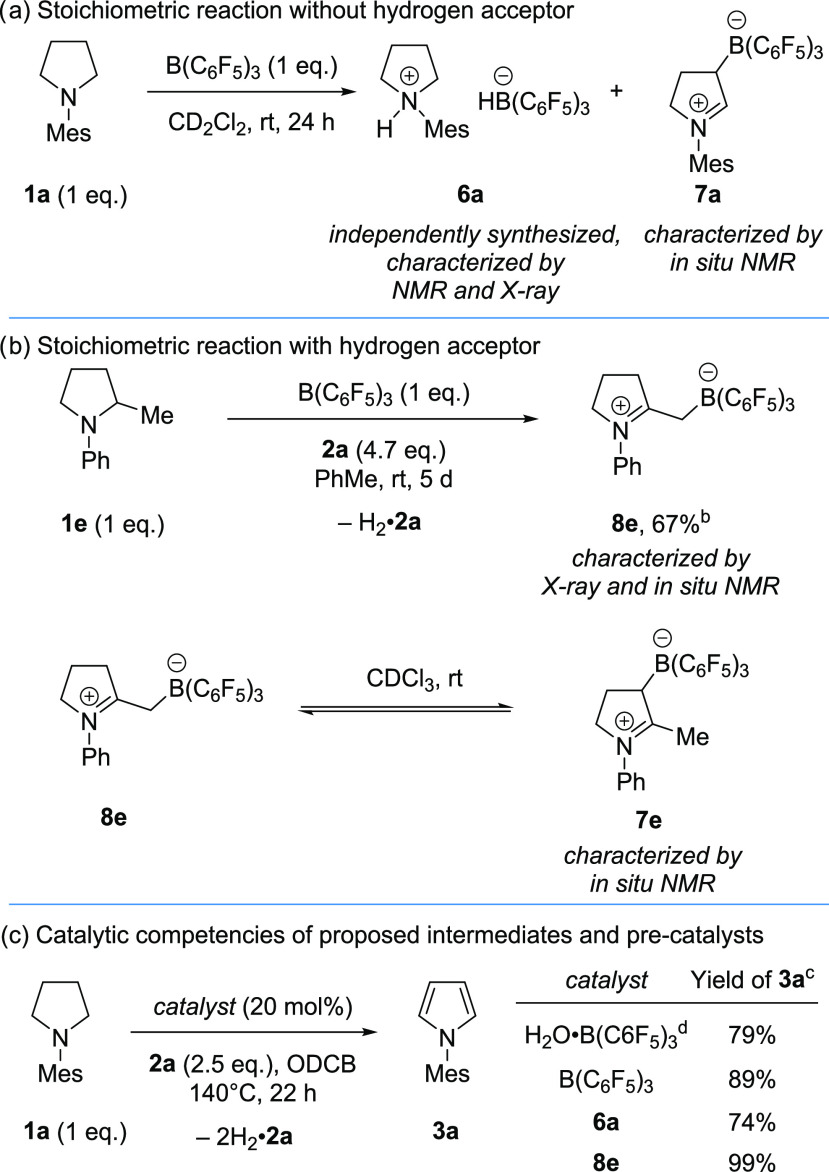
Mechanistic Investigation Unless otherwise stated, purified B(C_6_F_5_)_3_ (i.e., water-free) was used, and reactions were performed in an Ar glovebox. Isolated yield. Determined by ^1^H NMR analysis of the crude reaction mixtures with an internal standard. H_2_O·B(C_6_F_5_)_3_ weighed in air and treated *in situ* with Et_3_SiH (40 mol %). Mes = 2,4,6-trimethylphenyl.

The mechanism that underpins the B(C_6_F_5_)_3_-catalyzed dehydrogenation of pyrrolidines was probed experimentally and via DFT calculations. The reaction of one equivalent of pyrrolidine **1a** and one equivalent of B(C_6_F_5_)_3_ at ambient temperature formed quantitatively a 1:1 mixture of ammonium borohydride **6a** and zwitterion **7a** (Scheme 5a). Ammonium borohydride **6a** was independently synthesized (via the corresponding ammonium chloride, H_2_O·B(C_6_F_5_)_3_ and triethylsilane)^38^ and characterized by NMR spectroscopy and X-ray crystallography. The borohydride counterion is readily identified in the ^11^B NMR spectrum as a doublet at −25.1 ppm (*J*_HB_ = 88.0 Hz).^39^ The structural parameters of **6a** are unremarkable, except for the observed NH---HB distance of 2.21(3) Å, potentially indicating a weak dihydrogen bond in the solid state.^40^

Zwitterion **7a** was characterized *in situ* via ^1^H, ^11^B, ^13^C, and ^19^F NMR, and the data was consistent with similar zwitterions derived from B(C_6_F_5_)_3_ and an enamine.^41^ We were unable to isolate **7a** from the reaction or independently synthesize it for further structural characterization.

We also performed a 1:1 reaction between B(C_6_F_5_)_3_ and 2-methyl pyrrolidine **1e** in the presence of alkene **2a** and observed the precipitation of zwitterion **8e**. Zwitterion **8e** was characterized via X-ray crystallography and *in situ* NMR spectroscopy (Scheme 5b). Presumably, **8e** is formed via the addition of an isomeric enamine to B(C_6_F_5_)_3_ (*cf*. enamine/dihydropyrrole **10** and discussion below). In CDCl_3_, **8e** slowly equilibrated to a 1:1 mixture with a compound assigned as zwitterion **7e** (an analogous structure to **7a**), which was characterized *in situ* via NMR spectroscopy. To note, the hydrogenation product of alkene **2a** (isobutyltrimethylsilane, H_2_·**2a**) was observed in all acceptor-based reactions (see SI).^42^ It is also worth noting that while pyrroles are reported to react directly with B(C_6_F_5_)_3_ in a S_E_Ar manner to form boronate products,^31^ we did not observe any S_E_Ar products that would have otherwise removed B(C_6_F_5_)_3_ from the catalytic cycle.

We investigated the catalytic competency of the potential intermediates ammonium borohydride **6a** and zwitterion **8e** in the dehydrogenation of **1a** under the optimized conditions. We found that in both cases, pyrrole **3a** was formed in similar quantities to when B(C_6_F_5_)_3_ [prepared *in situ* from H_2_O·B(C_6_F_5_)_3_/Et_3_SiH or pure B(C_6_F_5_)_3_] was used (Scheme 5c).

**Figure 1:**
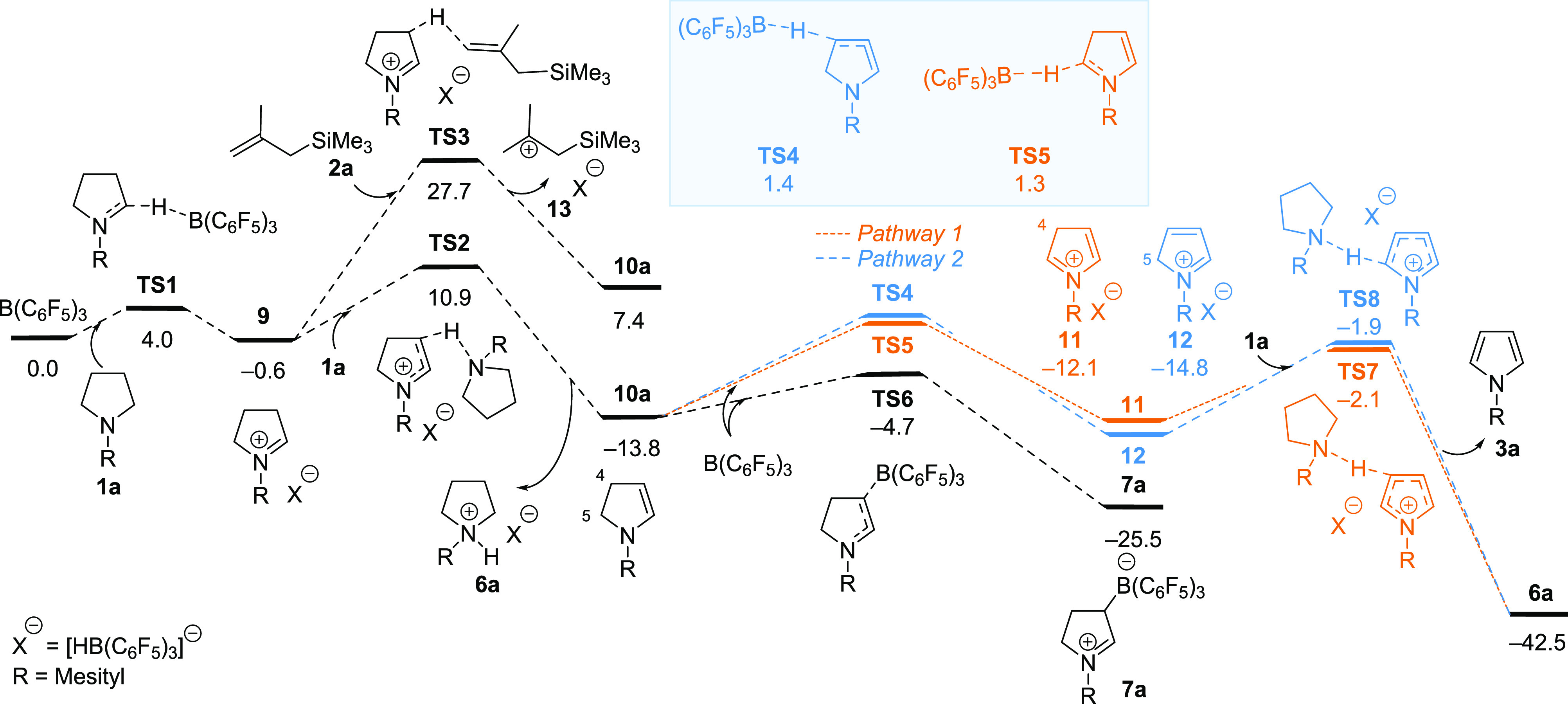
Gibbs free energy profiles (in kcal/mol) for the catalytic dehydrogenation of pyrrolidines.

DFT calculations at the B3LYP-D3/6-311G(d,p)//(SMD)M06–2*x*/6-311G(d,p) level of theory provided detailed mechanistic insight into the catalytic dehydrogenation of pyrrolidine **1a** leading to the formation of pyrrole **3a** (Figure 1). B(C_6_F_5_)_3_ abstracts hydride from the α-nitrogen position of **1a**, resulting in the formation of the iminium ion pair **9**. The formation of **9** occurs through a low barrier transition state **TS1** with a free energy barrier of 4.0 kcal/mol. Deprotonation of **9** by allyl silane **2a** via transition state **TS3** is computed to have a free energy barrier of 28.3 kcal/mol. In contrast, the deprotonation of **9** by pyrrolidine **1a**, resulting in the formation of ammonium borohydride **6a** and dihydropyrrole **10**, was observed to be more favorable kinetically, with a lower energy barrier of 11.5 kcal/mol at transition state **TS2**. Subsequently, two potential competing pathways leading to the formation of pyrroles **3a** and ammonium borohydride **6a** have been calculated. In pathway 1 (orange, **10a**-**TS5**-**11**-**TS7**), abstraction of hydride at C5 of dihydropyrrole **10a** (α-nitrogen) precedes deprotonation at C4. Alternatively, in pathway 2 (blue, **10a**-**TS4**-**12**-**TS8**), hydride abstraction at C4 (γ-nitrogen) takes place before deprotonation at C5. The calculated results reveal that the free energy barriers of transition states **TS4** and **TS5** (1.4 vs 1.3 kcal/mol), as well as **TS7** and **TS8** (−2.1 vs −1.9 kcal/mol), are similar, signifying that both pathways are viable options. Of note, borane-mediated γ-nitrogen hydride abstraction has only been previously observed with Hantzsch esters^43^ and 2-alkylideneimidazolines.^44^

The formation of out-of-cycle zwitterion **7a** from **10a** and B(C_6_F_5_)_3_ was also calculated and exhibited a lower energy barrier via **TS6** of 9.1 kcal/mol compared to the barriers observed in either pathway 1 or 2. Upon analysis of the free energy curves thermodynamically, it becomes apparent that the decrease in free energy of 11.7 kcal/mol observed for **7a** is outweighed by the significantly more exothermic formation of pyrrole **3a**, as indicated by a substantial decrease in free energy of 28.7 kcal/mol.

**Figure 2:**
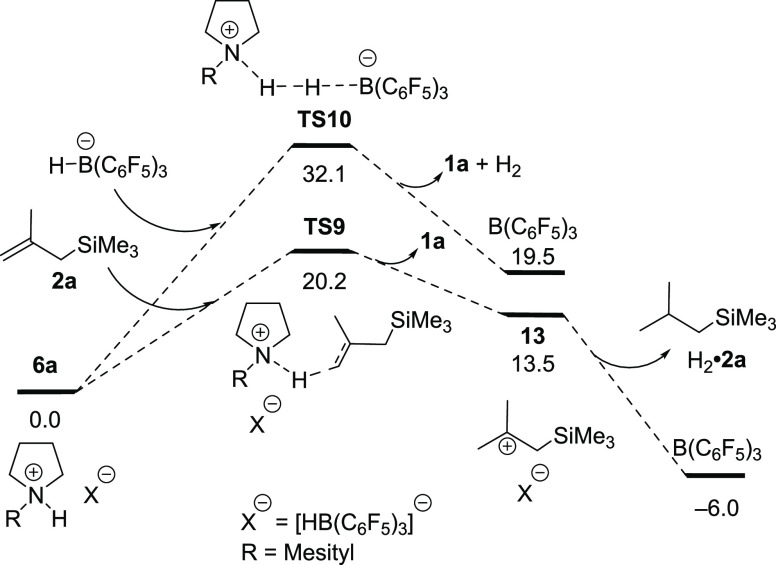
Gibbs free energy profiles (in kcal/mol) for the acceptor versus acceptorless catalytic dehydrogenation of pyrrolidines.

To evaluate the regeneration of the B(C_6_F_5_)_3_ catalyst, the free energy curves in Figure 2 were utilized. The acceptorless reaction involving the direct H_2_ formation from **6a** proceeded via **TS10**. The acceptor-based process proceeds via the two-step hydrogenation of alkene **2a** by ammonium borohydride **6a**, where carbocation **13** is formed via transition state **TS9**, and subsequent hydride transfer forms B(C_6_F_5_)_3_ and alkane H_2_·**2a**. The free energy of **TS10** (32.1 kcal/mol) was significantly higher than that **of TS9** (20.2 kcal/mol), indicating the hydrogenation of alkene **2a** by ammonium borohydride **6a** is more favorable and is consistent with the experimental observations. This result highlights the critical importance of the alkene acceptor.

**Scheme 6:**
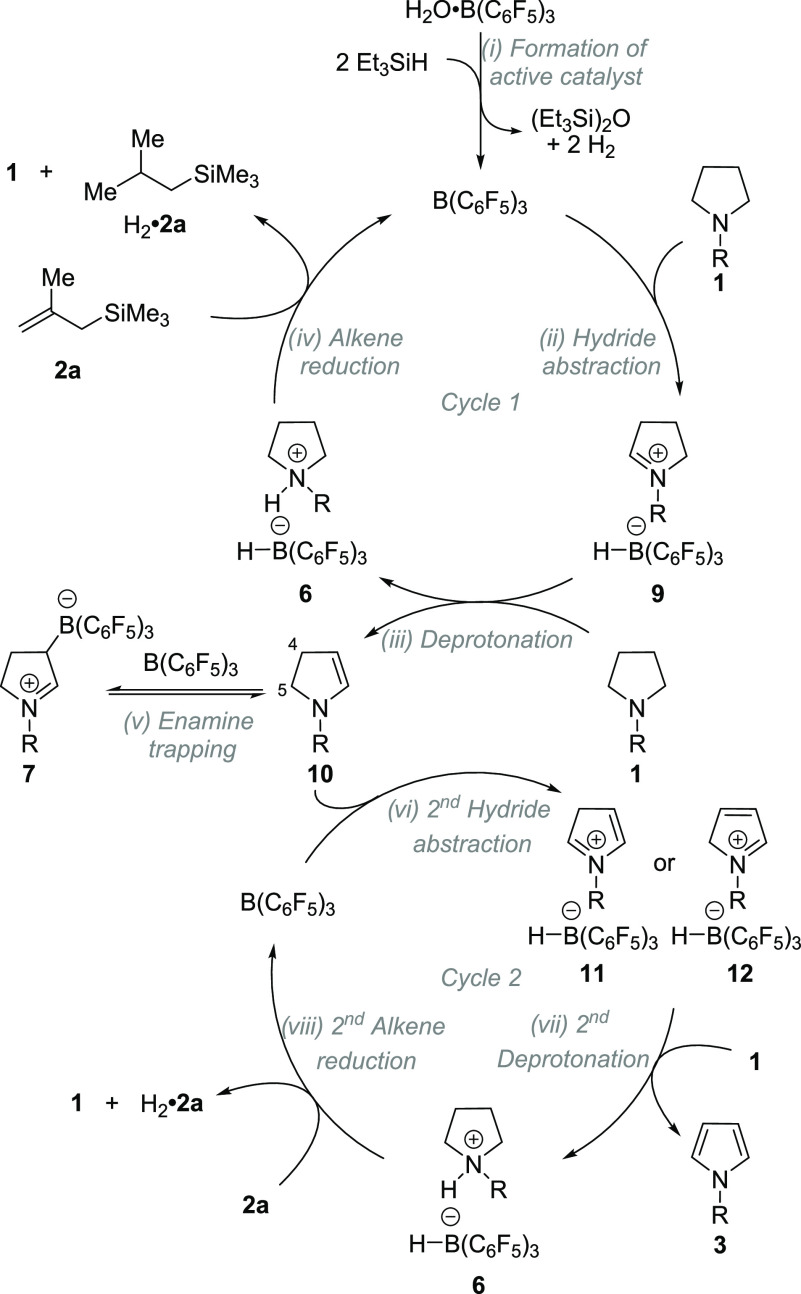
Proposed Mechanism

Based on the experimental and computational evidence, we propose the following mechanism (Scheme 6). Active B(C_6_F_5_)_3_ is formed *in situ* from H_2_O·B(C_6_F_5_)_3_ and Et_3_SiH. B(C_6_F_5_)_3_ mediated α-nitrogen hydride abstraction in pyrrolidine **1** forms iminium borohydride **9**. The iminium portion of **9** is deprotonated by starting pyrrolidine **1** to form ammonium borohydride **6** and dihydropyrrole **10**, thus removing the first equivalent of H_2_ from the amine. The B(C_6_F_5_)_3_ catalyst is regenerated during the irreversible hydrogenation of alkene **2a** by ammonium borohydride **6** to form alkane H_2_·**2a**,^42^ completing Cycle 1. Dihydropyrrole **10** (an enamine) is reversibly trapped with B(C_6_F_5_)_3_ to form the zwitterion **7**, and it is the likely resting state of the catalyst. Zwitterions **7** may serve as a reservoir for otherwise highly reactive dihydropyrroles **10**. Dihydropyrrole **10** can then undergo B(C_6_F_5_)_3_ mediated hydride abstraction at either C5 (α-nitrogen) or C4 (γ-nitrogen) to form iminium borohydrides **11** or **12**, respectively. Deprotonation of **11** or **12** yields pyrrole **3** and ammonium borohydride **6**, which in turn hydrogenates alkene **2a**, closing Cycle 2.

## Conclusions

In conclusion, we have reported the direct borane-catalyzed dehydrogenation of pyrrolidines to form pyrroles. The relative stability of pyrrolidines means that they can be carried through a synthetic sequence, and the pyrrole can be unmasked at a later stage. While pyrroles are sensitive to oxidative conditions and, therefore, previous methods typically required the presence of electron-withdrawing groups, the method presented here does not have these constraints, and new classes of pyrrolidines can serve as direct synthons for pyrroles for the first time. The borane catalyst is commercially available, and the method is operationally simple where standard glassware and techniques are used. These factors, and the wide and easy accessibility of pyrrolidines, render the borane-catalyzed approach presented here as a valuable strategy for the synthesis of pyrroles. In addition, we report a mild method for the dehydrogenation of indolines to form indoles. The mechanism has been studied and is proposed to operate via facile B(C_6_F_5_)_3_-mediated α-nitrogen hydride abstraction in pyrrolidines, followed by deprotonation of the generated iminium salt to form a dihydropyrrole and an ammonium borohydride. The dihydropyrrole is reversibly trapped by B(C_6_F_5_)_3_ to form adducts that may serve as reservoirs for the reactive dihydropyrrole. A second hydride abstraction event occurs either at the α- or unusually at the γ-nitrogen positions. Observation of this unusual regioselectivity may lead to new catalytic processes for the remote functionalization of amines. Regeneration of B(C_6_F_5_)_3_ via direct hydrogen evolution from ammonium borohydride was found to be extremely high in energy, but this challenge was solved by the addition of a crucial alkene hydrogen acceptor.

## Supporting Information Available

CCDC reference numbers for X-ray crystallography data: 2305442 (**6a**); X-ray crystal structure of **6a** (CIF)
CCDC reference numbers for X-ray crystallography data: 2296869 (**8e**); X-ray crystal structure of **8e** (CIF)
Calculated coordinates (XYZ)
Experimental procedures, computational details, and characterization data including NMR spectra and X-ray data (PDF)

## Supplementary Material

cs3c05444_si_001.cif

cs3c05444_si_002.cif

cs3c05444_si_003.xyz
