# Supplementary material for: B(C6F5)3-Catalyzed Dehydrogenation of Pyrrolidines to Form Pyrroles
Source: ACS Catal. 2024 Mar 18;14(7):4856–64. doi: 10.1021/acscatal.3c05444 (PMC11002826; doi:10.1021/acscatal.3c05444)
Supplement: Supplementary file 4 — cs3c05444_si_004.pdf [file cs3c05444_si_004.pdf]

Supporting information

$B(C_6F_5)_3$-Catalyzed dehydrogenation of pyrrolidines to form pyrroles

Ana Alvarez-Montoya,[¥‡] Joseph P. Gillions,[¥‡] Laura Winfrey,[¥] Rebecca R. Hawker,[¥] Kuldip Singh,[¥] Fabrizio Ortu,[¥] Yukang Fu,[†] Yang Li,[†]* Alexander P. Pulis[¥]*

[¥] *School of Chemistry, University of Leicester, Leicester, LE1 7RH, United Kingdom*

[†] *School of Chemical Engineering, Dalian University of Technology, No. 2 Linggong Road, Dalian, 116024, P.R.C.*

* Correspondence to a.pulis@leicester.ac.uk and chyangli@dlut.edu.cn

Contents

1. General considerations.....5
1.1 Materials.....5
1.2 Analysis.....5
2. Preparation of starting materials and NMR spectra.....6
2.1 Synthesis of pyrrolidines.....6
General procedure 1: Alkylation of anilines using K_2CO_3.....6
General procedure 2: Alkylation of anilines.....6
General procedure 3: Synthesis of 1-substituted-2,5-dimethyl pyrrolidines.....6
1-Mesitylpyrrolidine 1a.....7
2,5-Dimethyl-1-phenylpyrrolidine 1b.....8
1-(2-Chlorophenyl)pyrrolidine 1c.....9
1-(2-Bromophenyl)-3-methyl-1*H*-pyrrole-2,5-dione S1.....10
1-(2-Bromophenyl)-3-methylpyrrolidine-2,5-dione S2.....11
1-(2-Bromophenyl)-3-methylpyrrolidine 1d.....12
2-Methyl-1-phenylpyrrolidine 1e.....13
1-(2-Chlorophenyl)-2-methylpyrrolidine 1f.....14
1-(2-Bromophenyl)-2-methylpyrrolidine 1g.....15
1-(2-Methoxyphenyl)-2-methylpyrrolidine 1h.....16
2-Methyl-1-(2-phenoxyphenyl)pyrrolidine 1i.....17
1-(4-Chlorophenyl)-2-methylpyrrolidine 1j.....18
1-(4-Bromophenyl)-2-methylpyrrolidine 1k.....19
2-Methyl-1-(*p*-tolyl)pyrrolidine 1l.....20
1-(4-methoxyphenyl)-2-methylpyrrolidine 1m.....21
3-(2,5-dimethylpyrrolidin-1-yl)benzoic acid 1n.....22
1-(3-(((*tert*-Butyldimethylsilyl)oxy)methyl)phenyl)-2,5-dimethylpyrrolidine 1o.....23
4-Bromo-2-*tert*-butylaniline S3.....25
1-(4-Bromo-2-(*tert*-butyl)phenyl)pyrrolidine, 1p.....26
1-(2-(*tert*-Butyl)-4-(4,4,5,5-tetramethyl-1,3,2-dioxaborolan-2-yl)phenyl)pyrrolidine, 1q.....27
1-(2,4-Dichlorophenyl)-2-methylpyrrolidine 1r.....29
1-(5-Chloro-2-methoxyphenyl)-2-methylpyrrolidine 1s.....30
1-(2-Chloro-5-(trifluoromethyl)phenyl)-2-methylpyrrolidine 1t.....31
1-(3-Chloro-4-methylphenyl)-2,5-dimethylpyrrolidine 1u.....33
1-(3-Chloro-4-methylphenyl)-2-methylpyrrolidine 1v.....35

1-(4-Methoxy-2,6-dimethylphenyl)pyrrolidine 1w 36
1-Mesityl-3-phenylpyrrolidine-2,5-dione S4 37
1-Mesityl-3-phenylpyrrolidine 1x 38
2-Methyl-1-(2,3,5,6-tetrafluorophenyl)pyrrolidine 1y 39
1,2-Bis(4-(2-methylpyrrolidin-1-yl)phenyl)ethane 1z 41
1-(4-Bromonaphthalen-1-yl)-2-methylpyrrolidine 1aa 42
1-(*tert*-Butyl)pyrrolidine 1ab 43
1-Benzyl-2,5-dimethylpyrrolidine 1ac 44
2,5-Dimethyl-1-(3-phenylpropyl)pyrrolidine 1ad 45
2,5-Dimethyl-1-(thiophen-2-ylmethyl)pyrrolidine 1ae 46
1-(1-Phenylcyclohexyl)pyrrolidine (rolicyclidine) 1af 47
Estrone 3-methyl ether S5 48
1-(3-Methoxy-14-methyl-7,8,9,11,12,13,14,15,16,17-decahydro-6H-cyclopenta[a]phenanthren-15-yl)pyrrolidine 1ag 49
1-(4-(4-Chloro-3,5-dimethylphenoxy)phenyl)-2-methylpyrrolidine 1ah 50
2.2 Synthesis of indolines 51
1-Methylindoline 4a 51
1,2-Dimethyl indoline 4b 52
3. Optimization 53
4. Experimental procedures for dehydrogenation 54
General procedure 4: Preparation of a solution of water-free $B(C_6F_5)_3$ [in situ preparation of water-free $B(C_6F_5)_3$] 54
General procedure 5: Dehydrogenation of *N*-heterocycles with silane drying technique. 54
General procedure 6: Preparation and isolation of water-free $B(C_6F_5)_3$. 54
General procedure 7: Dehydrogenation of pyrrolidines with isolated water-free $B(C_6F_5)_3$. 54
1-Mesityl-1*H*-pyrrole 3a 55
Using 2a as the drying agent and acceptor to form 3a: 55
Preparative scale reaction to form 3a: 55
2,5-Dimethyl-1-phenyl-1*H*-pyrrole 3b 57
1-(2-Chlorophenyl)pyrrole 3c 58
1-(2-Bromophenyl)-3-methyl-1H-pyrrole 3d 59
2-Methyl-1-phenyl-pyrrole 3e 60
1-(2-Chlorophenyl)-2-methylpyrrole 3f 61
1-(2-Bromophenyl)-2-methyl-1*H*-pyrrole 3g 62
1-(2-Methoxyphenyl)-2-methyl-1*H*-pyrrole 3h 63
2-Methyl-1-(2-phenoxyphenyl)-1*H*-pyrrole 3i 64
1-(4-Chlorophenyl)-2-methyl-*1H*-pyrrole 3j 65
1-(4-Bromophenyl)-2-methyl-1*H*-pyrrole 3k 66
2-Methyl-1-(*p*-tolyl)-1*H*-pyrrole 3l 67
1-(4-Methoxyphenyl)-2-methyl-1*H*-pyrrole 3m 68

3-(2,5-Dimethyl-1H-pyrrol-1-yl)benzoic acid 3n 69
1-(3-(((*tert*-Butyldimethylsilyl)oxy)methyl)phenyl)-2,5-dimethyl-1*H*-pyrrole 3o 70
1-(4-Bromo-2-(*tert*-butyl)phenyl)-1*H*-pyrrole 3p 71
1-(2-(*tert*-Butyl)-4-(pinacolboryl)phenyl)pyrrole 3q 72
1-(2,4-Dichlorophenyl)-2-methyl-1*H*-pyrrole 3r 74
1-(5-Chloro-2-methoxyphenyl)-2-methyl-1H-pyrrole 3s 75
1-(2-Chloro-5-(trifluoromethyl)phenyl)-2-methyl-1*H*-pyrrole 3t 76
1-(3-Chloro-4-methylphenyl)-2,5-dimethyl-1*H*-pyrrole 3u 78
1-(3-Chloro-4-methylphenyl)-2,5-dimethyl-1*H*-pyrrole 3v 79
1-(4-Methoxy-2,6-dimethylphenyl)-1*H*-pyrrole 3w 80
1-Mesityl-3-phenyl-1H-pyrrole 3x 81
2-Methyl-1-(2,3,5,6-tetrafluorophenyl)pyrrole 3y 82
1,2-Bis(4-(2-methyl-1*H*-pyrrol-1-yl)phenyl)ethane 3z 84
1-(4-Bromonaphthalen-1-yl)-2-methyl-1*H*-pyrrole 3aa 85
N-*tert*-Butylpyrrole 3ab 86
N-t*ert*-Butylpyrrole, 3ab 87
1-Benzyl-2,5-dimethyl-1*H*-pyrrole 3ac 88
2-Methyl-1-(3-phenylpropyl)-1*H*-pyrrole 3ad 89
2,5-Dimethyl-1-(thiophen-2-ylmethyl)-1*H*-pyrrole 3ae 90
1-(1-Phenylcyclohexyl)pyrrole (tetradehydro-rolicyclidine) 3af 91
1-(3-Methoxy-14-methyl-7,8,9,11,12,13,14,15,16,17-decahydro-6H-cyclopenta[a]phenanthren-15-yl)pyrrole 3ag 92
1-(4-(4-Chloro-3,5-dimethylphenoxy)phenyl)-2-methylpyrrole 3ah 93
Optimization of 3af, 3ag and 3ah 94
Dehydrogenation of indolines 95
1-Methyl-1*H*-indole 5a 95
1,2-Dimethyl-1*H*-indole 5b 96
5. Mechanistic studies 97
5.1 Identification of reaction intermediates 97
Stoichiometric reaction of N-mesityl pyrrolidine and $B(C_6F_5)_3$ 97
Synthesis of ammonium borohydrides: 99
General procedure 8: Synthesis of ammonium borohydrides 99
1-Mesitylpyrrolidin-1-ium trispentafluorophenylhydridoborate 6a 99
1-(2-Chlorophenyl)pyrrolidin-1-ium trispentafluorophenylhydridoborate 6c 102
Tris(pentafluorophenyl)((1-phenyl-3,4-dihydro-2H-pyrrol-1-ium-5-yl)methyl)borate, 8n 104
Isobutyltrimethylsilane $H_2{\cdot}2a$ 108
5.2 Reactivity of reaction intermediates 109
General procedure 9: Catalytic competency of isolated intermediates 109
Using isolated water-free $B(C_6F_5)_3$: 109
Using 6a: 110

Using 8e: 110
Reduction of methallyltrimethylsilane with 1-(2-chlorophenyl)pyrrolidin-1-ium trispentafluorophenylhydridoborate, 6c 111
6. DFT calculations 113
6.1 Computational methods 113
6.2 Cartesian coordinates of the B3LYP-D3 /6-311G(d,p) computed structures 113
$B(C_6F_5)_3$ 113
$HB(C_6F_5)_3^-$ 113
1a 114
9 114
10a 114
6a 115
7a 115
11 116
12 117
3a 117
2a 117
13 118
H_2·2a 118
TS1 118
TS2 119
TS3 120
TS4 121
TS5 122
TS6 122
TS7 123
TS8 124
TS9 125
TS10 126
7. Crystallographic data 127
8. References 130

1. General considerations

1.1 Materials

Reagents were purchased from commercial suppliers and used without further purification unless stated otherwise. $B(C_6F_5)_3$ was purchased from Alfa Aesar and Accela ChemBio and was received as $H_2O{\cdot}B(C_6F_5)_3$, and unless specified used without further purification, where it was weighed in air on the open bench prior to use. Where reactions required a nitrogen atmosphere, a Schlenk line using house nitrogen gas (with a $Ca(OH)_2$ drying column), oven dried glassware (cooled under vacuum), and standard syringe-septa techniques were used. Where reactions required an argon atmosphere, a Schlenk line or Vigor glovebox using Pureshield argon cylinders were used in conjunction with standard glovebox/Schlenk line manipulation techniques. Flash column chromatography was performed using silica gel 60 (Fisher Scientific) and a suitable eluent. Automated column chromatography was performed using silica gel 60 (Fisher Scientific) and a suitable eluent on a Biotage Isolera One. TLC was performed on silica TLC plates (Merck-Millipore 60 F254) with the given solvent system and spots were observed using a UV lamp (254 nm) and then developed with potassium permanganate or I_2 vapour. For NMR of air sensitive species, C_6D_6 was dried by refluxing with potassium, and isolated by vacuum transfer, whilst $CDCl_3$ and CD_2Cl_2 were dried by refluxing over CaH_2, and isolated by vacuum transfer.

1.2 Analysis

High resolution mass spectrometry (HRMS) was carried out on a Waters Acquity XEVO Q ToF spectrometer (electrospray). Peaks are reported in units of mass to charge ratio (m/z). NMR spectra were obtained using Bruker 400 MHz, Bruker 500 MHz, and Bruker Avance NEO 800 MHz NMR (equipped with a 5 mm 13C-optimised triple resonance {H, C/N, D TXO} cryoprobe) spectrometers at ambient temperatures unless otherwise stated. Chemical shifts (δ) are quoted in parts per million (ppm) and coupling constants (*J*) are in hertz (Hz). ^{13}C NMR spectra are ^{1}H decoupled. Residual solvent peaks were used as the internal reference for proton and carbon chemical shifts. Spectroscopic yields were determined by analysis of ^{1}H NMR spectra of reaction mixtures or crude mixtures, using nitromethane or 1,3,5-trimethoxy benzene as an internal standard, in comparison with spectra of the isolated and characterised products. IR data was obtained from the neat sample using a Bruker Alpha II ATR-IR, with frequency quoted in cm^{-1}.

2. Preparation of starting materials and NMR spectra

2.1 Synthesis of pyrrolidines

General procedure 1: Alkylation of anilines using K_2CO_3

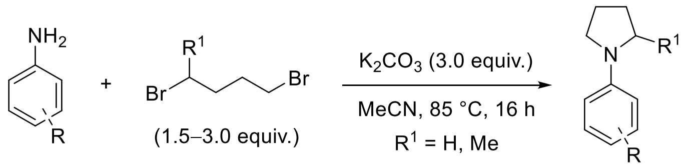

A two-neck round-bottomed flask equipped with reflux condenser and stirrer bar was charged with aniline (1.0 equiv.), K_2CO_3 (3.0 equiv.) dibromoalkane (1.5-3.0 equiv.) and MeCN (2.5 mL/mmol), before being heated to 85 °C overnight (approx. 16 h). Upon completion, benzylamine (1.0 equiv.) was added to quench unreacted alkyl bromide that would otherwise affect purification. The reaction heated to 85 °C for a further 2 h. After cooling, the mixture was quenched with $NaHCO_3$ (aq., sat.) and the aqueous layer extracted with EtOAc (×3). The organic phases were combined, washed with water and brine, dried over $MgSO_4$, filtered and concentrated in vacuo. The crude product was purified by column chromatography eluting the specified solvent systems to give the pure products.

General procedure 2: Alkylation of anilines

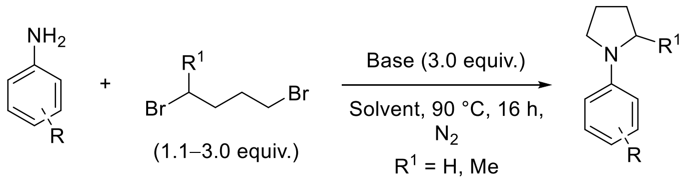

A 2-neck round-bottomed flask equipped with reflux condenser, septa, and stirrer bar was charged with K_2CO_3 (if used as base, 3.0 equiv.), aniline (1.0 equiv.), and solvent (2.5 mL/mmol) under a nitrogen atmosphere. If NaH used, NaH (60% dispersion in mineral oil, 3.0 equiv.) was added portionwise to the aniline solution, before the slow addition of 1,4-dibromopentane to the aniline solution (1.1-3.0 equiv.). The reaction was heated to 90 °C overnight (approx. 16 h). Upon completion, benzylamine (1.0 equiv.) was added, and the reaction heated to 90 °C for a further 2 h. After cooling, the mixture was quenched with saturated $NaHCO_3$ (aq., sat.) and the aqueous layer extracted with EtOAc (×3). The organic phases were combined, washed with water and brine, dried over $MgSO_4$, filtered and concentrated in vacuo. The crude product was purified by column chromatography eluting the specified solvent systems to give the pure products.

General procedure 3: Synthesis of 1-substituted-2,5-dimethyl pyrrolidines

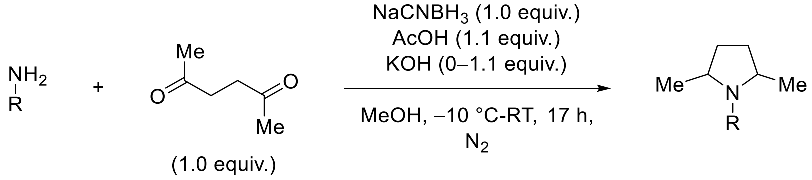

A round-bottomed flask equipped with stirrer bar was charged with aniline (1.0 equiv.), acetic acid (1.1 equiv.), potassium hydroxide (0-1.1 equiv.) and methanol (0.56 mL/mmol). The reaction mixture was cooled to -10 ℃ (NaCl/ice bath) before 2,5-hexadione (1.0 equiv.) and sodium cyanoborohydride (1.0 equiv.) were added, before the reaction was left to stir for 17 h, gradually warming to room temperature. The reaction mixture was quenched with HCl (aq., 4 M) and the methanol removed in vacuo. The aqueous solution was washed with diethyl ether before basification by addition of K_2CO_3. The product was extracted with diethyl ether before being washed with brine, dried with Na_2SO_4, filtered and concentrated in vacuo. The crude product was purified by column chromatography eluting the specified solvent systems to give the pure products.

1-Mesitylpyrrolidine 1a

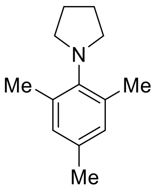

The title compound was prepared according to general procedure 1 using 2,4,6-trimethylaniline (3.10 mL, 22.1 mmol), K_2CO_3 (6.78 g, 49.1 mmol), and 1,4-dibromobutane (4.00 mL, 33.5 mmol). Purification by flash column chromatography on silica gel (eluent = 20% DCM in pet. ether), gave the title compound **1a** as a light orange oil (2.23 g, 11.8 mmol, 53%). **R_f** = 0.6 (eluent = 5% EtOAc in pet. ether); **^{1}H NMR** (400 MHz, $CDCl_3$) δ_H = 6.86 (2H, s), 3.22–3.12 (4H, m), 2.26 (3H, s), 2.23 (6H, s), 2.03–1.92 (4H, m); **^{13}C NMR** (101 MHz, $CDCl_3$) δ_C = 142.8 (C), 138.2 (2×C), 134.5 (C), 129.2 (2×CH), 50.2 (2×CH_2), 26.6 (2×CH_2), 20.8 (CH_3), 18.6 (2×CH_3). Spectroscopic data in accordance with that stated in the literature.[1]

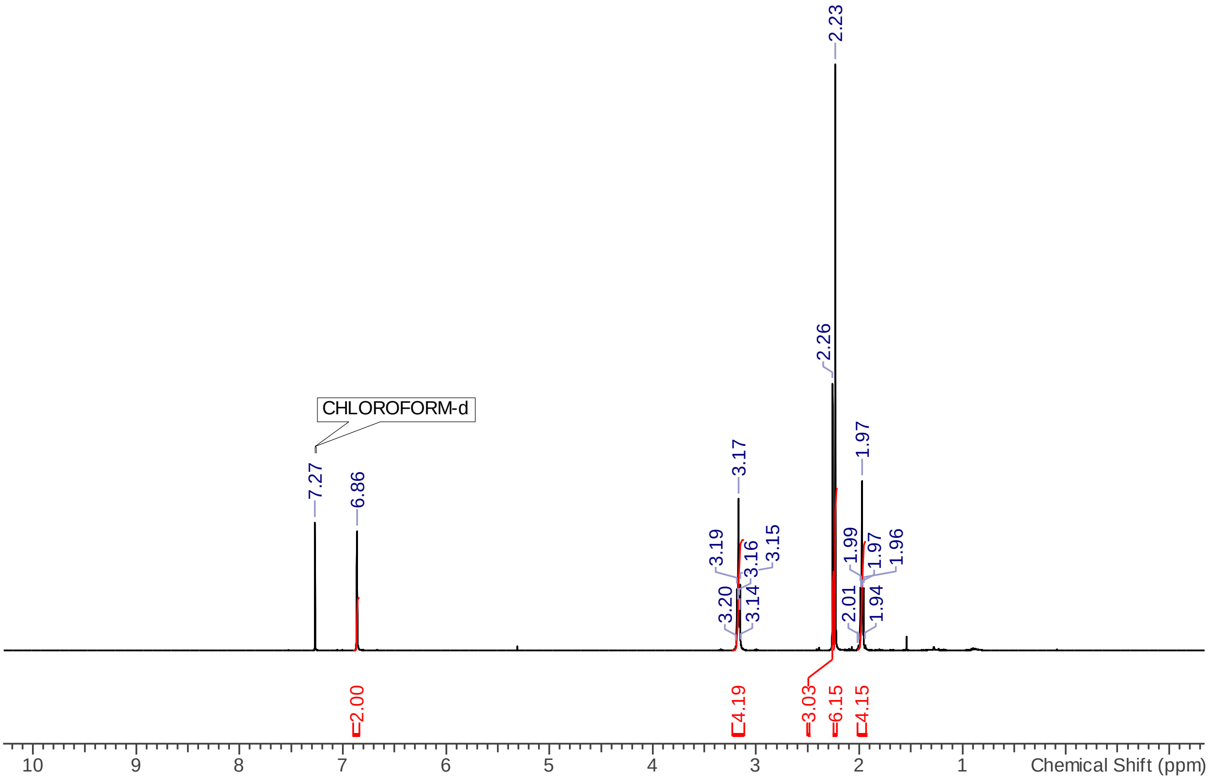

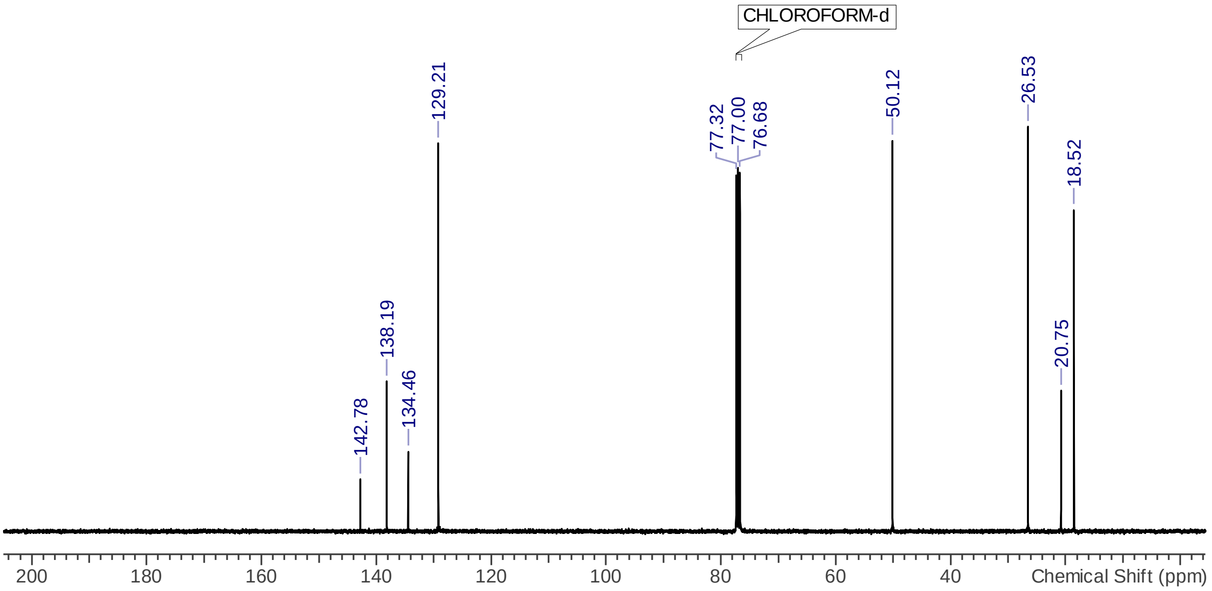

2,5-Dimethyl-1-phenylpyrrolidine 1b

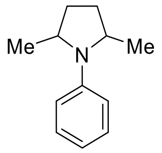

The title compound was prepared according to general procedure 3 using aniline (1.00 g, 10.7 mmol), AcOH (0.68 mL, 1.18 mmol), KOH (150 mg, 0.27 mmol), 2,5-hexadione (1.25 mL, 10.7 mmol) and $NaBH_3CN$ (672 mg, 10.7 mmol). This yielded a crude mixture of pyrrole and pyrrolidine (600 mg) which was redissolved in AcOH (20 mL), cooled to 10°C (ice/water), and $NaBH_3CN$ (1.10 g, 17.5 mmol) added, before stirring at room temperature and stirring for 17 h. Upon completion, the reaction mixture was concentrated in vacuo, basified with NaOH (aq., 2M), and extracted with Et_2O (3 × 30 mL), before the combined organics were concentrated in vacuo. The crude materials were purified by flash column chromatography on silica gel (eluent = 10% DCM in pet. ether), to give the title compound **1b** as a white solid (200 mg, 1.16 mmol, 33%, 93:7 mixture of diastereomers). $\mathbf{R_f}$ = 0.31 (eluent = 10% DCM in pet. ether); **^{1}H NMR** (400 MHz, $CDCl_3$) **Common signals:** δ_H = 7.25–7.17 (2H, m), 6.69–6.56 (3H, m); **Major diastereomer:** δ_H = 4.02 (2H, app. quin, *J* = 6.1 Hz), 2.32–2.18 (2H, m), 1.67–1.59 (2H, m), 1.12 (6H, d, *J* = 6.1 Hz); **Minor diastereomer:** δ_H = 3.81–3.77 (2H, m), 2.08–2.02 (2H, m), 1.76–1.69 (2H, m), 1.30 (6H, d, *J* = 6.1 Hz); **^{13}C NMR** (101 MHz, $CDCl_3$) δ_C = 147.3 (C)*, 145.2 (C), 130.9 (2×CH)*, 129.1 (2×CH), 115.2 (CH)*, 114.5 (CH) 113.3 (2×CH), 111.9 (2×CH)*, 55.9 (2×CH)*, 52.7 (2×CH), 32.3 (2×CH_2)*, 30.3 (2×CH_2), 21.8 (2×CH_3), 18.1 (2×CH_3). Spectroscopic data in accordance with that stated in the literature.[2] **Signals attributed to minor diastereomer.*

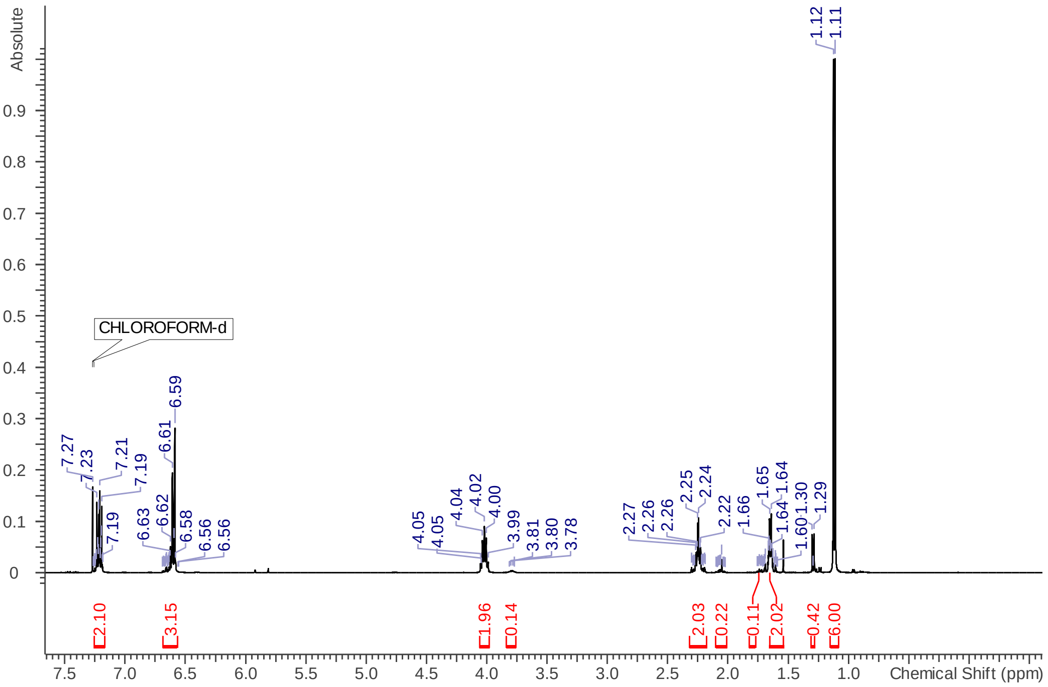

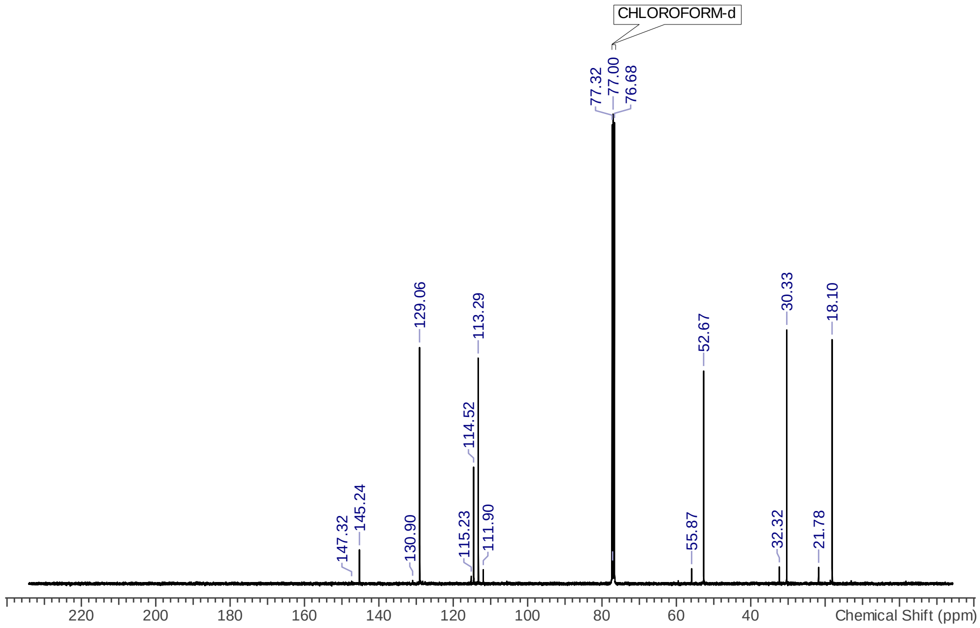

1-(2-Chlorophenyl)pyrrolidine 1c

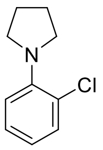

The title compound was prepared according to general procedure 2 using 2-chloroaniline (1.06 mL, 10.1 mmol), K_2CO_3 (1.52 g, 11.0 mmol), 1,4-dibromobutane (1.30 mL, 11.0 mmol), and DMF (10 mL). Purification by flash column chromatography on silica gel (eluent = 20% DCM in pet. ether), gave the title compound **1c** as a yellow oil (774 mg, 4.37 mmol, 44%). **R_f** = 0.29 (eluent = 20% DCM in pet. ether); **^{1}H NMR** (400 MHz, $CDCl_3$) δ_H = 7.30 (1H, dd, *J* = 7.8, 1.4 Hz), 7.15 (1H, ddd, *J* = 8.2, 7.2, 1.4 Hz), 6.90 (1H, dd, *J* = 8.2, 1.2 Hz), 6.79 (1H, ddd, *J* = 7.8, 7.2, 1.2 Hz), 3.45–3.32 (4H, m), 2.01–1.89 (4H, m); **^{13}C NMR** (101 MHz, $CDCl_3$) δ_C = 147.1 (C), 131.2 (CH), 127.1 (C), 123.4 (CH), 120.1 (CH), 117.0 (CH), 50.9 (2×CH_2), 25.1 (2×CH_2). Spectroscopic data in accordance with that stated in the literature.[3]

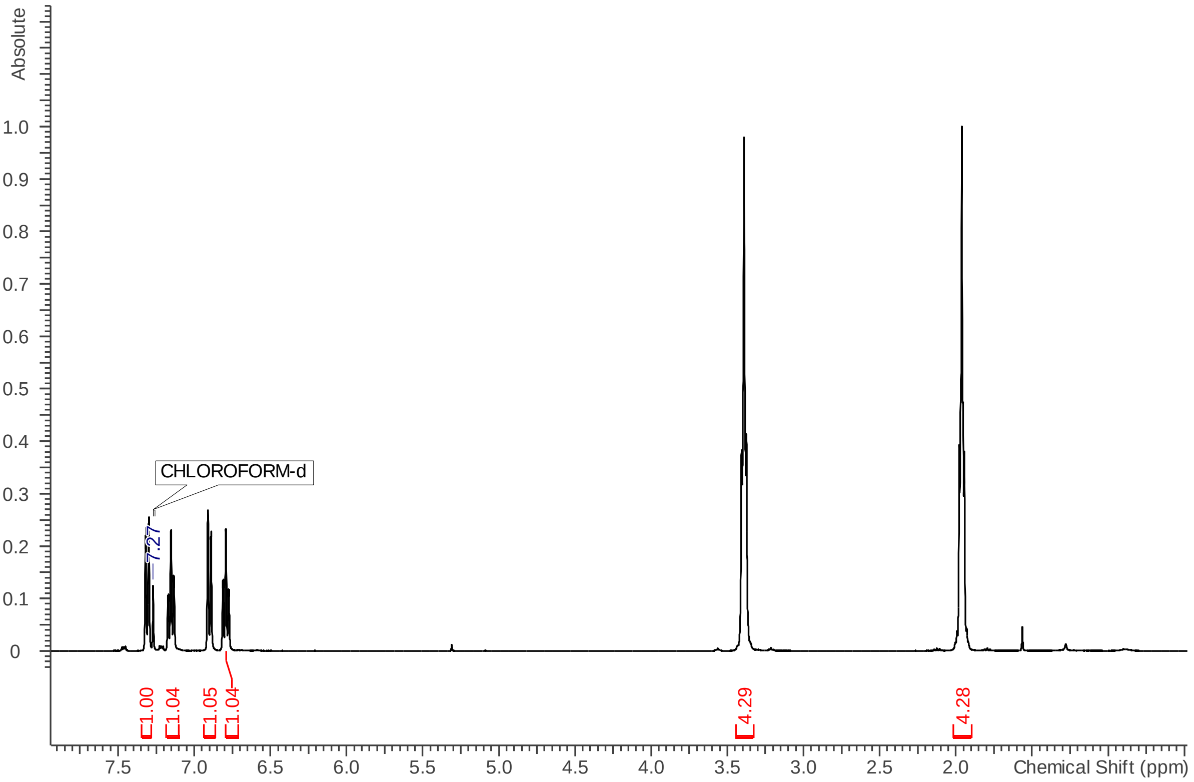

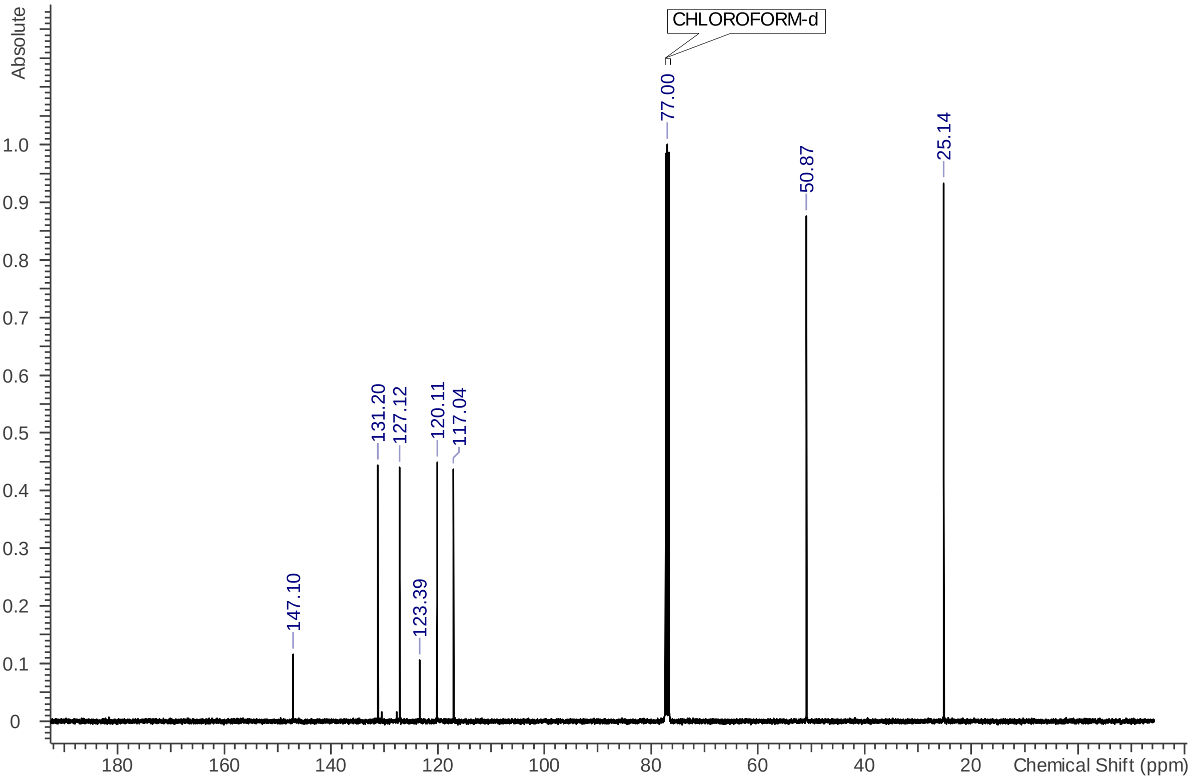

1-(2-Bromophenyl)-3-methyl-1*H*-pyrrole-2,5-dione S1

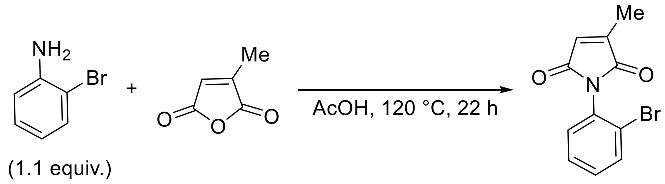

A 250 mL round bottomed flask equipped with stirrer bar and reflux condenser was charged with citraconic anhydride (0.80 mL, 8.95 mmol), 2-bromoaniline (1.10 mL, 10.0 mmol) and AcOH (25 mL) before being heated to 120 °C for 22 h. Upon completion, excess AcOH was removed in vacuo, yielding a yellow oil. Purification by flash column chromatography on silica gel (eluent = 20–33% EtOAc in pet. ether) gave the title compound **S1** as a cream solid (2.01 g, 7.6 mmol, 85%, 73:27 mix of atropisomers). **R_f** = 0.18 (eluent = 20% EtOAc/petroleum ether); **^{1}H NMR** (400 MHz, $CDCl_3$) **Common signals:** δ_H = 7.73–7.65 (1H, m), 7.45–7.37 (1H, m), 7.34–7.27 (1H, m), 7.26–7.23 (1H, m), 6.53–6.47 (1H, m); **Major atropisomer:** δ_H = 2.16 (3H, d, *J* = 1.5 Hz); **Minor atropisomer:** δ_H = 2.19 (3H, d, *J* = 1.7 Hz); **^{13}C NMR** (101 MHz, $CDCl_3$) δ_C = 169.79 (C=O), 169.78 (C=O)*, 168.67 (C=O), 168.66 (C=O)*, 146.04 (C), 146.03 (C)*, 133.35 (CH)*, 133.33 (CH), 131.07 (C), 131.05 (C)*, 130.73 (CH), 130.71 (CH)*, 130.67 (CH), 130.65 (CH)*, 128.25 (CH), 128.23 (CH)*, 127.65 (CH)*, 127.64 (CH)*, 123.17 (C)*, 123.16 (C), 11.09 (CH_3)*, 11.07 (CH_3); **HRMS** (ESI+): calculated for $[C_{11}H_9{}^{79}BrNO_2]^+$ $(M+H)^+$ m/z: 265.9817; found 265.9819; calculated for $[C_{11}H_9{}^{81}BrNO_2]^+$ $(M+H)^+$ m/z: 267.9796; found 267.9798; **FTIR** (cm^{-1}) ν = 3278, 1779, 1701 (both C=O), 1642 (C=C), 1577, 1526, 1483, 1433, 1388, 1296, 1268, 1210, 1174, 1140, 1101, 1061, 1045, 1026, 1012, 995, 875, 850, 760, 721, 700, 662, 644, 615, 571, 542, 467, 443. **Signals attributed to minor atropisomer.*

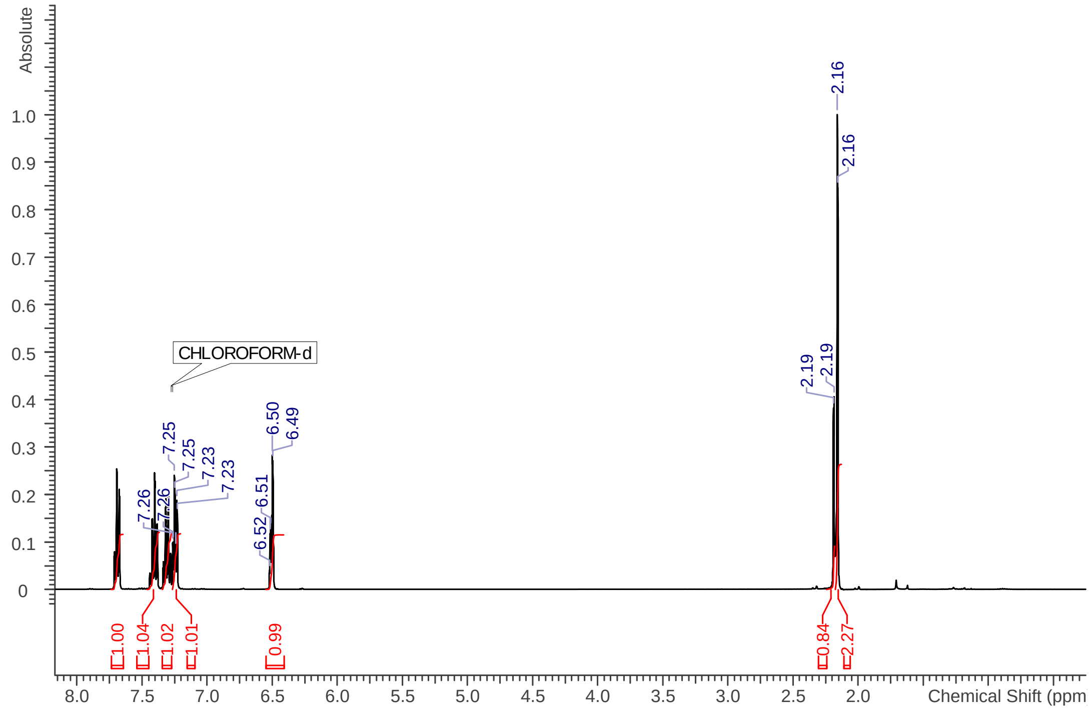

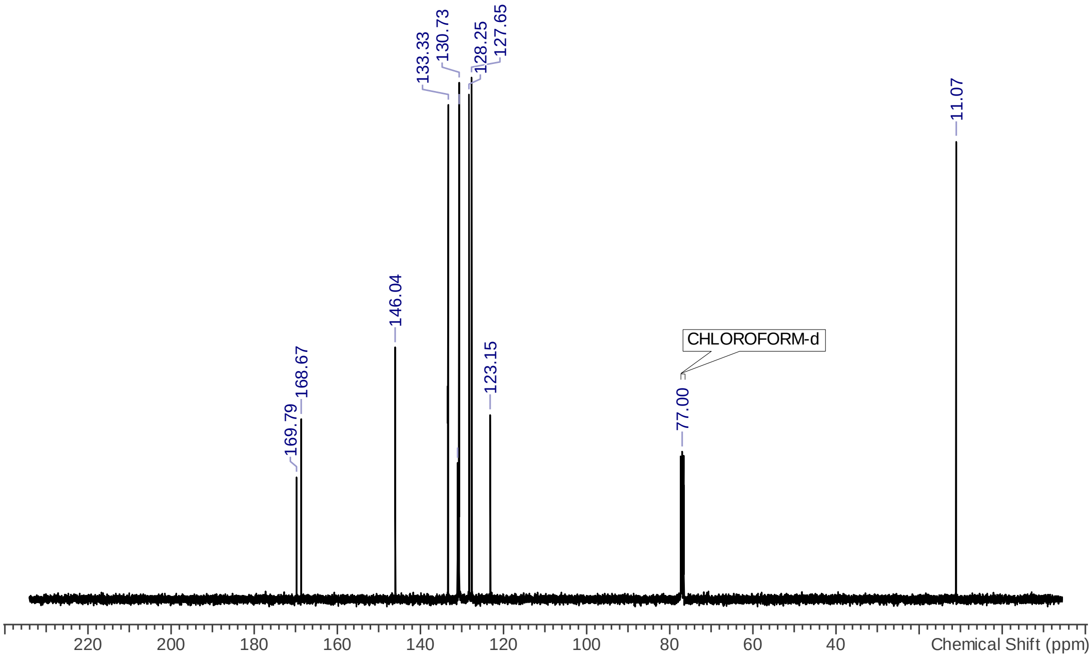

1-(2-Bromophenyl)-3-methylpyrrolidine-2,5-dione S2

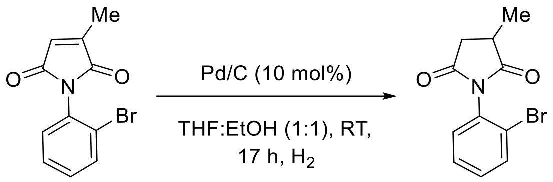

A 250 mL round bottomed flask equipped with stirrer bar was charged with 1-(2-Bromophenyl)-3-methyl-1*H*-pyrrole-2,5-dione (220 mg, 0.833 mmol), Pd/C (5.0 mg, 10% Pd by wt., 5.0 µmol) and THF:EtOH (1:1, 10 mL). A septa was fitted, before the atmosphere was exchanged for hydrogen (3 × balloon), and allowed to stir at ambient temperature for 17 h. Upon completion, the reaction mixture was filtered through Celite™ (washed with EtOAc) and concentrated in vacuo to give the title compound **S2** as a brown oil (142 mg, 0.53 mmol, 64%, 57:43 mixture of diastereomers) with no further purification required. **R_f** = 0.06 (eluent = 20% EtOAc/petroleum ether); **Common signals: ^{1}H NMR** (400 MHz, $CDCl_3$) δ_H = 7.71 (1H, dd, *J* = 8.0, 1.3 Hz), 7.44 (1H, app. td, *J* = 7.8, 1.3 Hz), 7.33 (1H, dd, *J* = 8.0, 7.8 Hz), 7.22 (1H, m), 3.22–3.01 (2H, m), 2.63–2.48 (1H, m). **Major diastereomer:** δ_H = 1.50 (3H, d, *J* = 7.2 Hz); **Minor diastereomer:** δ_H = 1.47 (3H, d, *J* = 7.2 Hz); **^{13}C NMR** (101 MHz, $CDCl_3$) δ_C = 178.71 (C=O), 178.69 (C=O)*, 174.7 (C=O), 174.5 (C=O)*, 133.52 (CH)*, 133.50 (CH), 131.74 (C)*, 131.65 (C), 130.94 (CH), 130.92 (CH)*, 130.0 (CH), 129.9 (CH)*, 128.53 (CH), 128.46 (CH)*, 122.3 (C), 122.2 (C)*, 36.84 (CH_2), 36.80 (CH_2)*, 35.29 (CH), 35.25 (CH)*, 17.3 (CH_3), 16.5 (CH_3)*; **HRMS** (ESI+): calculated for $[C_{11}H_{11}{}^{79}BrNO_2]^+$ $(M+H)^+$ m/z: 267.9973; found 267.9974; calculated for $[C_{11}H_{11}{}^{81}BrNO_2]^+$ $(M+H)^+$ m/z: 269.9953; found 269.9962. **FTIR** (cm^{-1}) ν = 2975, 2934 (both C-H), 1779, 1706 (both C=O), 1598, 1501, 1478, 1457, 1386, 1371, 1289, 1265, 1170, 1051, 1025, 993, 929, 909, 867, 760, 746, 716, 698, 654, 623, 598, 502, 446. **Signals attributed to minor diastereomer.*

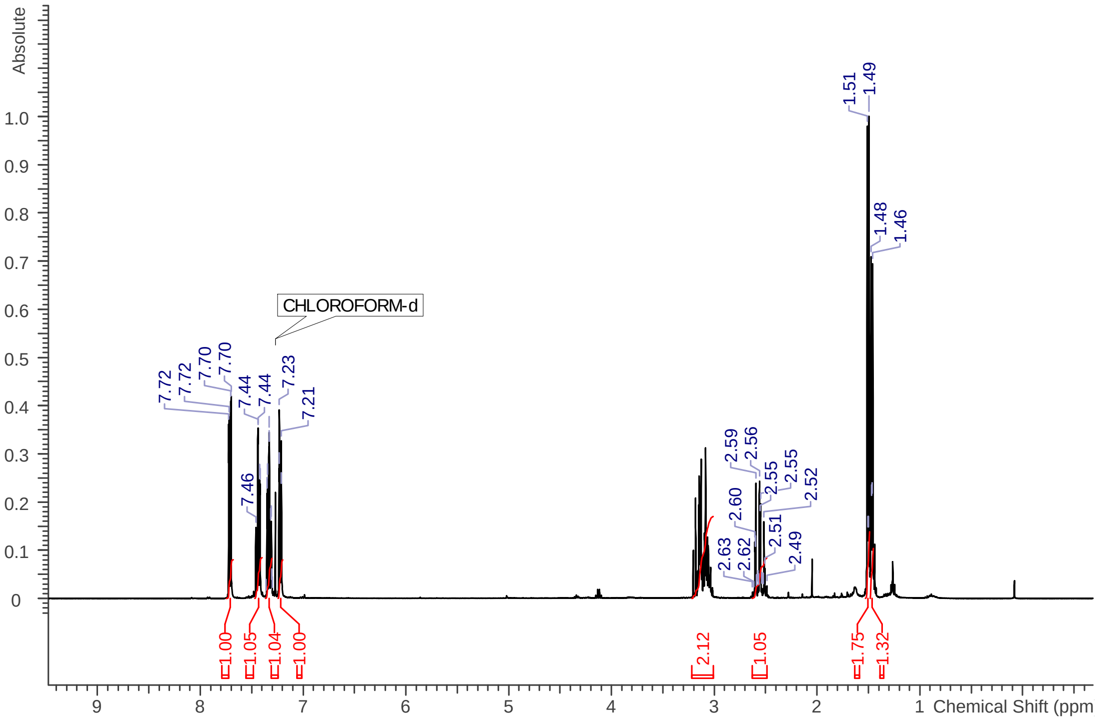

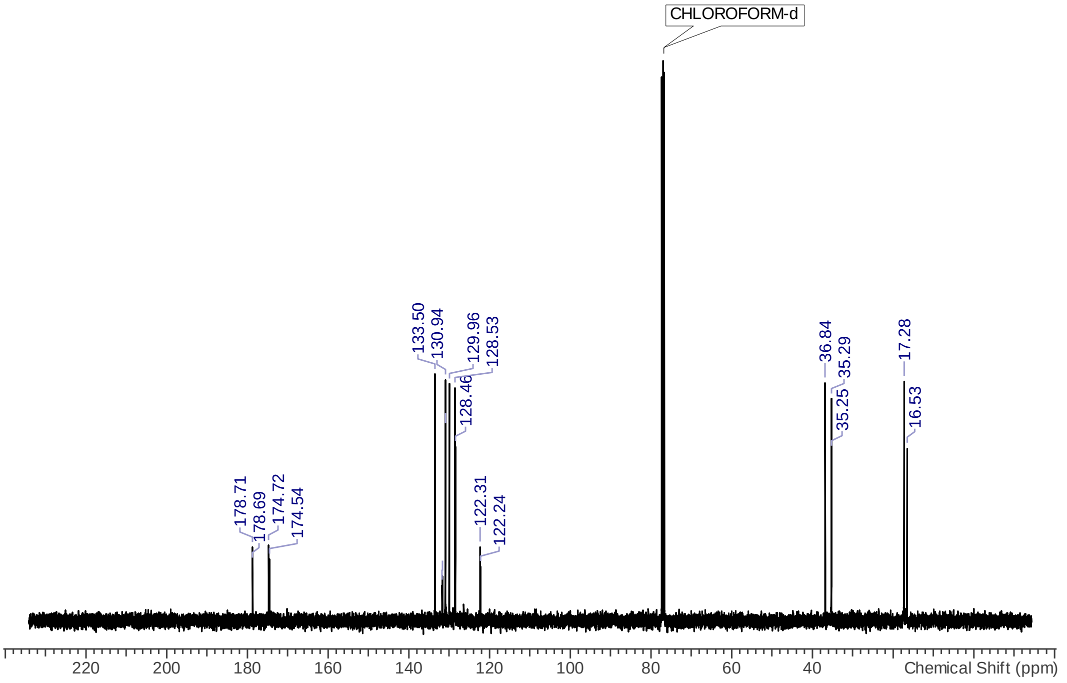

1-(2-Bromophenyl)-3-methylpyrrolidine 1d

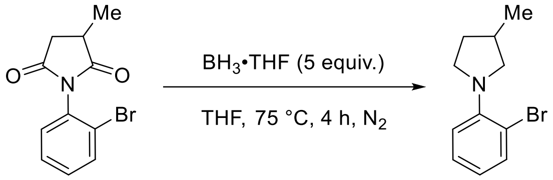

A 100 mL 2 neck round bottomed flask equipped with stirrer bar, reflux condenser and septa, was charged with 1-(2-bromophenyl)-3-methylpyrrolidine-2,5-dione, **S2** (400 mg, 1.49 mmol), and THF (10 mL) under a nitrogen atmosphere, before BH_3.THF (7.5 mL, 1.0 M, 7.5 mmol) was added dropwise. The reaction was then heated to 75 °C for 4 h, until TLC indicated completion. The reaction was quenched with MeOH (5 mL), before solvent was removed in vacuo. The resultant residue was heated to 110 °C with HCl (aq., 6M, 2 mL) for 30 mins, before the solution was basified with NaOH (aq., 15%) The product was then extracted with Et_2O (3 × 20 mL), before the combined organics were dried over $MgSO_4$, and concentrated in vacuo. Purification by flash column chromatography on silica gel (eluent = 25% DCM in pet. ether) gave the title compound **1d** as a colourless oil (85 mg, 0.35 mmol, 23%). **R_f** = 0.86 (eluent = 20% EtOAc in pet. ether); **^{1}H NMR** (400 MHz, $CDCl_3$) δ_H = 7.54 (1H, dd, *J* = 7.7, 1.6 Hz), 7.21 (1H, ddd, *J* = 8.3, 7.2, 1.6 Hz), 6.91 (1H, dd, *J* = 8.3, 1.5 Hz), 6.75 (1H, ddd, *J* = 7.7, 7.2, 1.5 Hz), 3.61 (1H, ddd, *J* = 9.3, 7.9, 7.2 Hz), 3.46 (1H, dd, *J* = 9.1, 7.0 Hz), 3.33 (1H, ddd, *J* = 9.3, 8.0, 4.1 Hz), 3.12 (1H, dd, *J* = 9.1, 7.8 Hz), 2.44–2.29 (1H, m), 2.13 (1H, dtd, *J* = 11.7, 7.2, 4.1 Hz), 1.64–1.52 (1H, m), 1.16 (3H, d, *J* = 6.6 Hz). **^{13}C NMR** (101 MHz, $CDCl_3$) δ_C = 148.6 (C), 134.6 (CH), 127.7 (CH), 120.8 (CH), 117.4 (CH), 112.9 (C), 58.8 (CH_2), 51.1 (CH_2), 33.5 (CH_2), 33.1 (CH), 18.6 (CH_3). **HRMS** (ESI$^+$): calculated for $[C_{11}H_{15}{}^{79}BrN]^+$ $(M+H)^+$ m/z: 240.0388; found 240.0386; calculated for $[C_{11}H_{15}{}^{81}BrN]^+$ $(M+H)^+$ m/z: 242.0367; found 242.0368. **FTIR** (cm^{-1}) ν = 2956, 2869, 2815 (all C-H), 1687, 1587, 1472, 1434, 1377, 1324, 1269, 1202, 1164, 1143, 1111, 1076, 1055, 1019, 953, 927, 907, 878, 827, 741, 671, 626, 545, 492, 442.

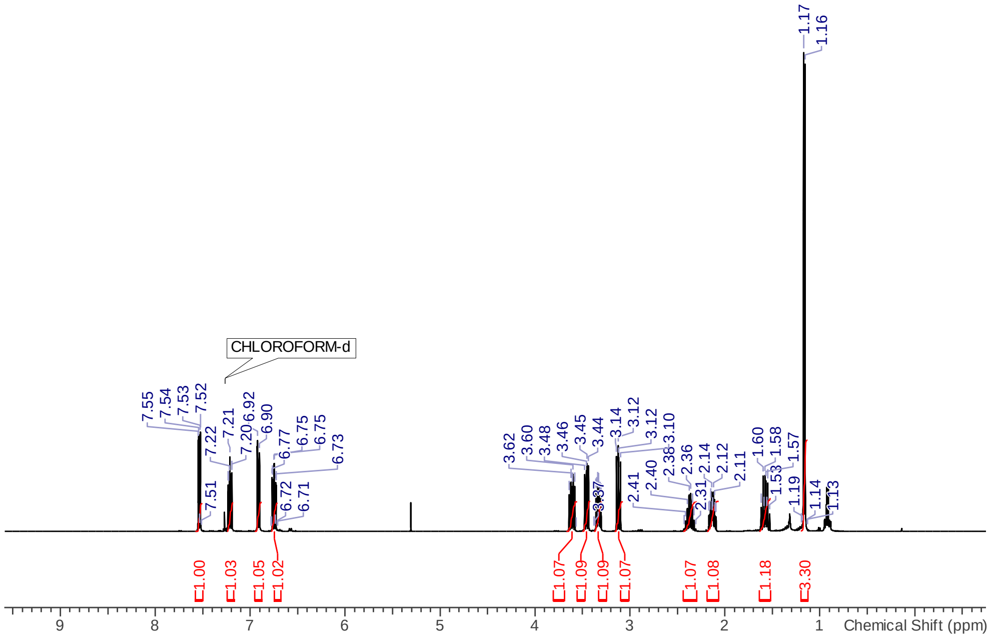

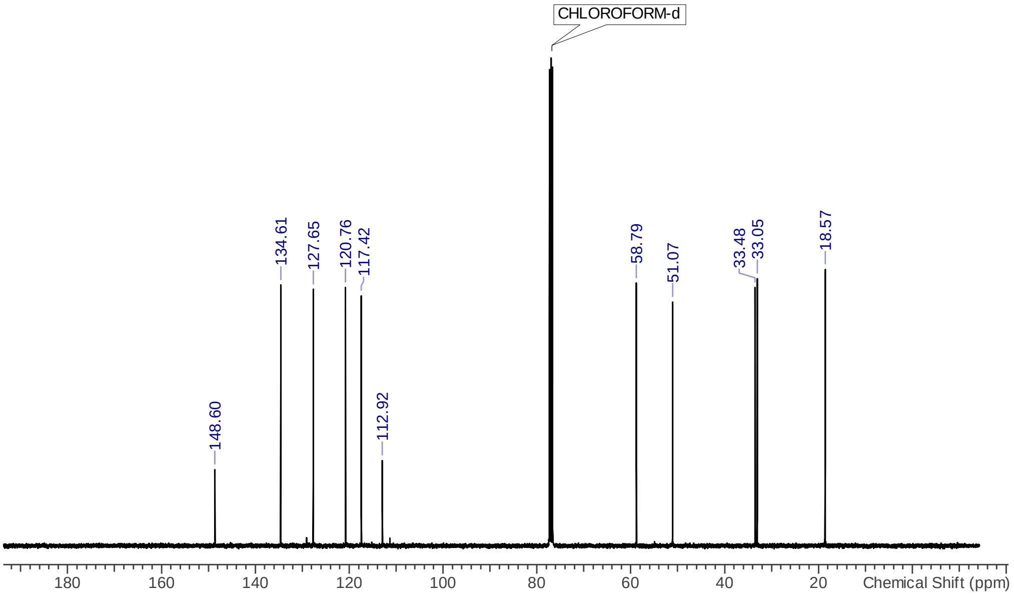

2-Methyl-1-phenylpyrrolidine 1e

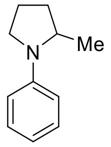

The title compound was prepared according to general procedure 1 using aniline (0.72 mL, 8.0 mmol), K_2CO_3 (3.32 g, 24.0 mmol), and 1,4-dibromopentane (1.20 mL, 8.8 mmol). Purification by flash column chromatography on silica gel (eluent = 20% DCM in pet. ether), gave the title compound **1e** as a brown oil (940 mg, 5.83 mmol, 73%). $\mathbf{R_f}$ = 0.33 (eluent = 20% DCM in pet. ether); **^{1}H NMR** (400 MHz, $CDCl_3$) δ_H = 7.27–7.21 (2H, m), 6.68–6.64 (1H, m), 6.63–6.58 (2H, m), 3.89 (1H, app. quind, *J* = 6.3, 1.7 Hz), 3.47–3.42 (1H, m), 3.22–3.13 (1H, m), 2.16–1.95 (3H, m), 1.78–1.63 (1H, m), 1.19 (3H, d, *J* = 6.3 Hz); **^{13}C NMR** (101 MHz, $CDCl_3$) δ_C = 147.2 (C), 129.1 (2×CH), 115.1 (CH), 111.7 (2×CH), 53.6 (CH), 48.1 (CH_2), 33.1 (CH_2), 23.3 (CH_2), 19.4 (CH_3); Spectroscopic data in accordance with that stated in the literature.[4]

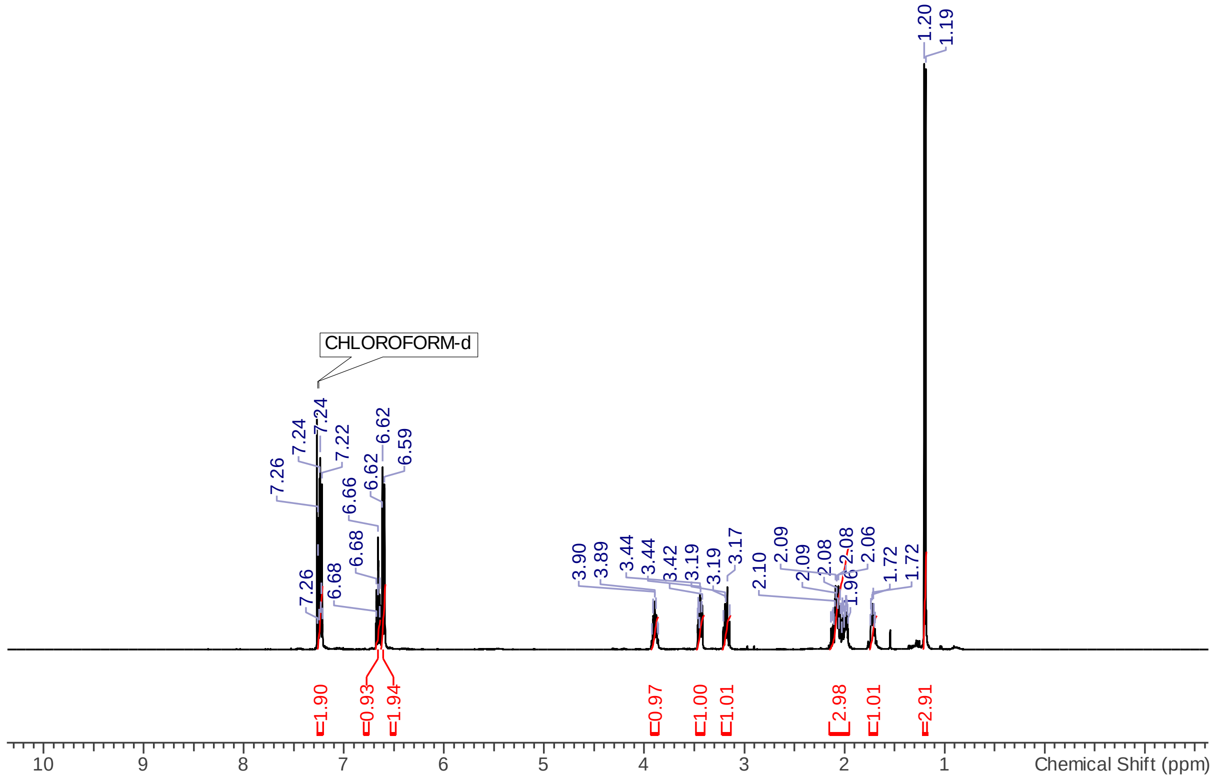

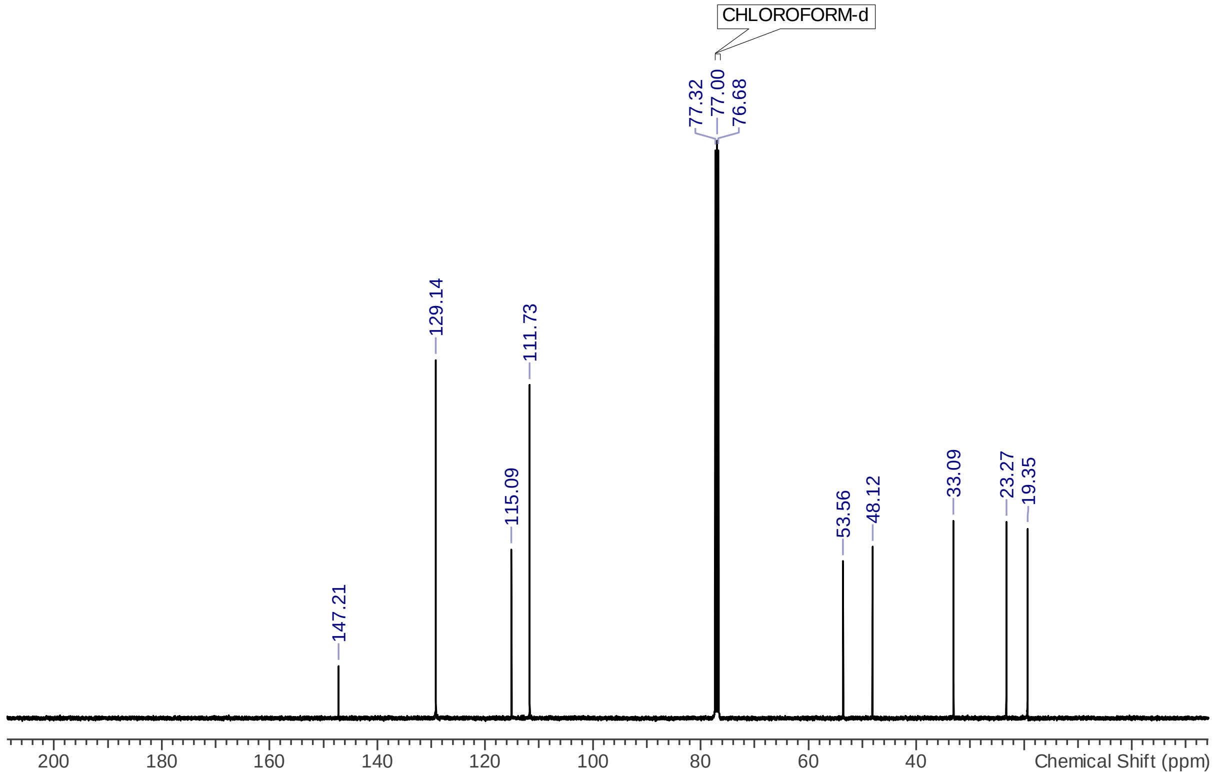

1-(2-Chlorophenyl)-2-methylpyrrolidine 1f

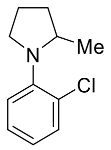

The title compound was prepared according to general procedure 2 using 2-chloroaniline (1.64 mL, 15.7 mmol), NaH (0.75 g, 31.4 mmol), 1,4-dibromopentane (1.98 mL, 15.7 mmol) and DMF (14 mL). Purification by flash column chromatography on silica gel (eluent = 20% DCM in pet. ether), gave the title compound **1f** as a light yellow oil (1.50 g, 7.70 mmol, 49%). **R_f** = 0.4 (eluent = 20% DCM in pet. ether); **^{1}H NMR** (400 MHz, $CDCl_3$) δ_H = 7.32 (1H, dd, *J* = 7.9, 1.6 Hz), 7.17 (1H, ddd, *J* = 8.2, 7.3, 1.6 Hz), 6.95 (1H, dd, *J* = 8.2, 1.3 Hz), 6.84 (7.9, 7.3, 1.3 Hz), 3.96 (1H, app. dquin, *J* = 7.9, 6.3 Hz), 3.85 (1H, app. td, *J* = 8.9, 7.3 Hz), 2.97 (1H, app. td, *J* = 8.9, 3.2 Hz), 2.25–2.15 (1H, m), 2.00–1.93 (1H, m), 1.87–1.76 (1H, m), 1.62 (1H, app. ddt, *J* = 12.0, 10.3, 8.1 Hz), 1.05 (3H, d, *J* = 6.3 Hz); **^{13}C NMR** (101 MHz, $CDCl_3$) δ_C = 146.4 (C), 130.9 (CH), 126.9 (CH), 126.2 (C), 121.0 (CH), 119.2 (CH), 54.5 (CH), 52.3 (CH_2) 33.9 (CH_2), 23.9 (CH_2), 19.0 (CH_3). Spectroscopic data in accordance with that stated in the literature.[5]

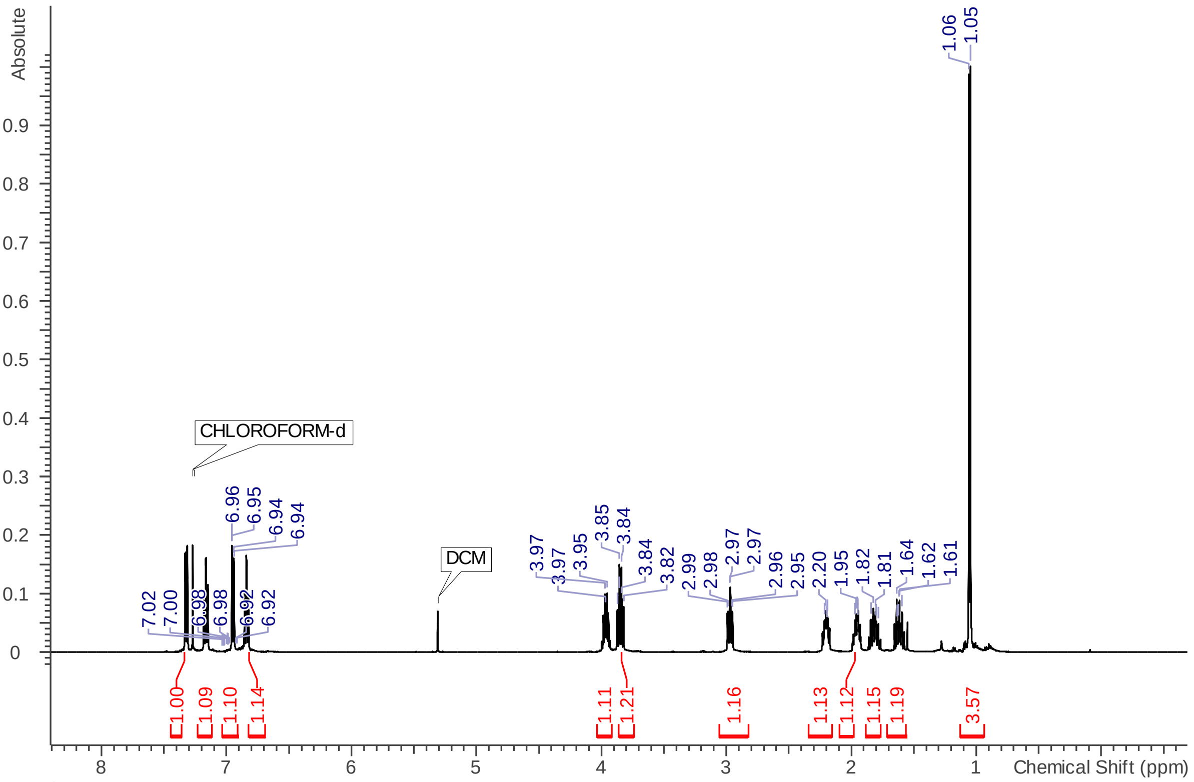

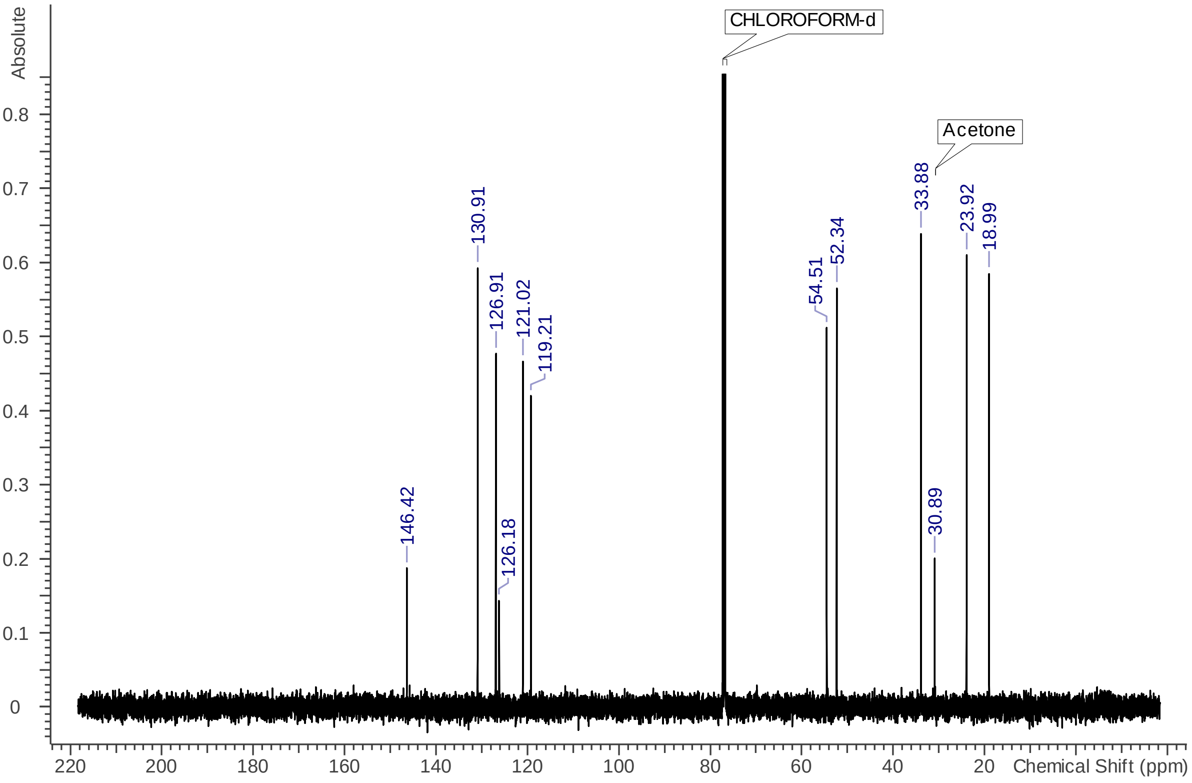

1-(2-Bromophenyl)-2-methylpyrrolidine 1g

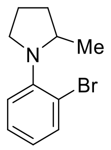

The title compound was prepared according to general procedure 2 using 2-bromoaniline (700 mg, 4.09 mmol), K_2CO_3 (1.41 g, 10.2 mmol), 1,4-dibromopentane (0.61 mL, 4.5 mmol), and DMF (10 mL). Purification by flash column chromatography on silica gel (eluent = 20% DCM in pet. ether), gave the title compound **1g** as a colourless oil (100 mg, 0.42 mmol, 10%). **R$_f$** = 0.5 (eluent = 20% DCM in pet. ether); **^{1}H NMR** (400 MHz, $CDCl_3$) δ_H = 7.53 (1H, dd, *J* = 8.0, 1.6 Hz), 7.22 (1H, ddd, *J* = 8.2, 7.1, 1.6 Hz), 6.98 (1H, dd, *J* = 8.2, 1.5 Hz), 6.80 (1H, ddd, *J* = 8.0, 7.1, 1.5 Hz), 3.97–3.85 (2H, m), 2.88 (1H, app td, *J* = 8.8, 3.8 Hz), 2.24–2.14 (1H, m), 2.02–1.91 (1H, m), 1.86–1.76 (1H, m), 1.67–1.54 (1H, m), 1.03 (3H, d, *J* = 6.1 Hz); **^{13}C NMR** (101 MHz, $CDCl_3$) δ_C = 148.0 (C), 134.2 (CH), 127.6 (CH), 122.2 (CH), 120.3 (CH), 117.4 (C), 54.8 (CH), 52.8 (CH_2), 33.7 (CH_2), 23.8 (CH_2), 18.9 (CH_3); **HRMS** (ESI$^+$) calculated for $[C_{11}H_{15}{}^{79}BrN]^+$ $(M+H)^+$ m/z: 240.0380; found 240.0388; calculated for $[C_{11}H_{15}{}^{81}BrN]^+$ $(M+H)^+$ m/z: 242.0367; found 242.0361.

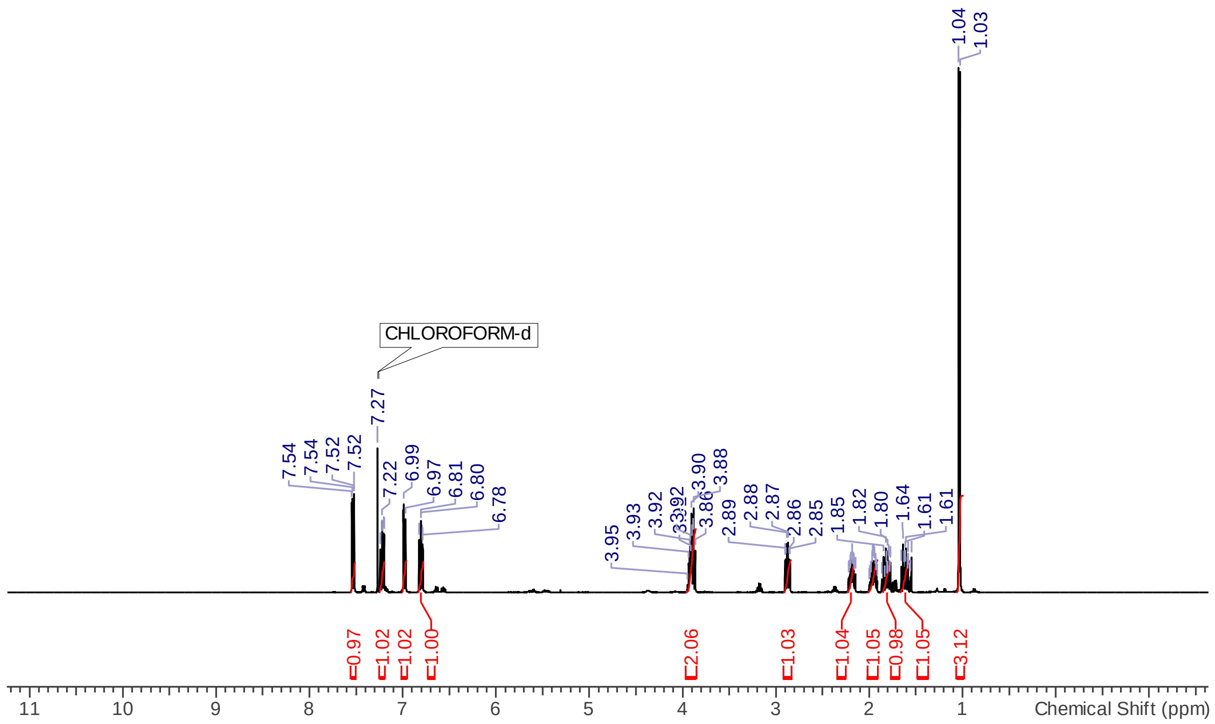

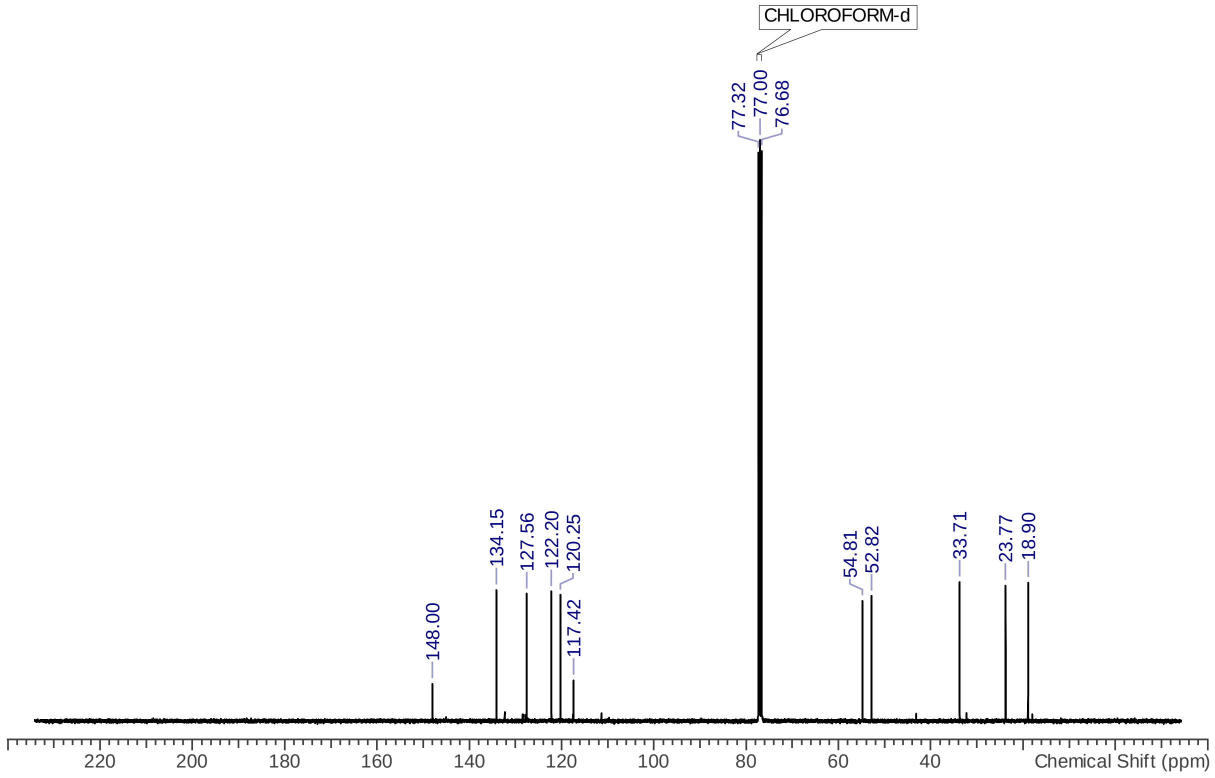

1-(2-Methoxyphenyl)-2-methylpyrrolidine 1h

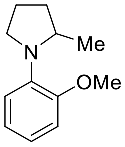

The title compound was prepared according to general procedure 1 using 2-anisidine (2.75 mL, 24.4 mmol), K_2CO_3 (10.0 g, 73.2 mmol), and 1,4-dibromopentane (4.94 mL, 36.6 mmol). Purification by flash column chromatography on silica gel (eluent = 10% EtOAc in pet. ether), gave the title compound **1h** as a black oil (2.76 g, 14.4 mmol, 59%). Rf = 0.25 (eluent = 10% EtOAc in pet. ether); **^{1}H NMR** (400 MHz, $CDCl_3$) δ_H = 6.93–6.81 (4H, m), 3.94 (1H, app. sxt, *J* = 6.5 Hz), 3.85 (3H, s), 3.71 (1H, dt, *J* = 9.4, 7.8 Hz), 2.96 (1H, td, *J* = 9.4, 3.8 Hz), 2.23–2.14 (1H, m), 1.99–1.89 (1H, m), 1.88–1.76 (1H, m), 1.67–1.55 (1H, m), 1.04 (3H, d, *J* = 6.3 Hz); **^{13}C NMR** (101 MHz, $CDCl_3$) δ_C = 151.4 (C), 138.8 (C), 120.8 (CH), 120.1 (CH), 117.1 (CH), 111.5 (CH), 55.4 (CH), 54.2 (CH_3), 51.4 (CH_2), 33.6 (CH_2), 23.4 (CH_2), 18.9 (CH_3); **HRMS** (ESI$^+$) calculated for $[C_{12}H_{18}NO]^+$ (M+H)$^+$ m/z: 192.1388; found 192.1388.

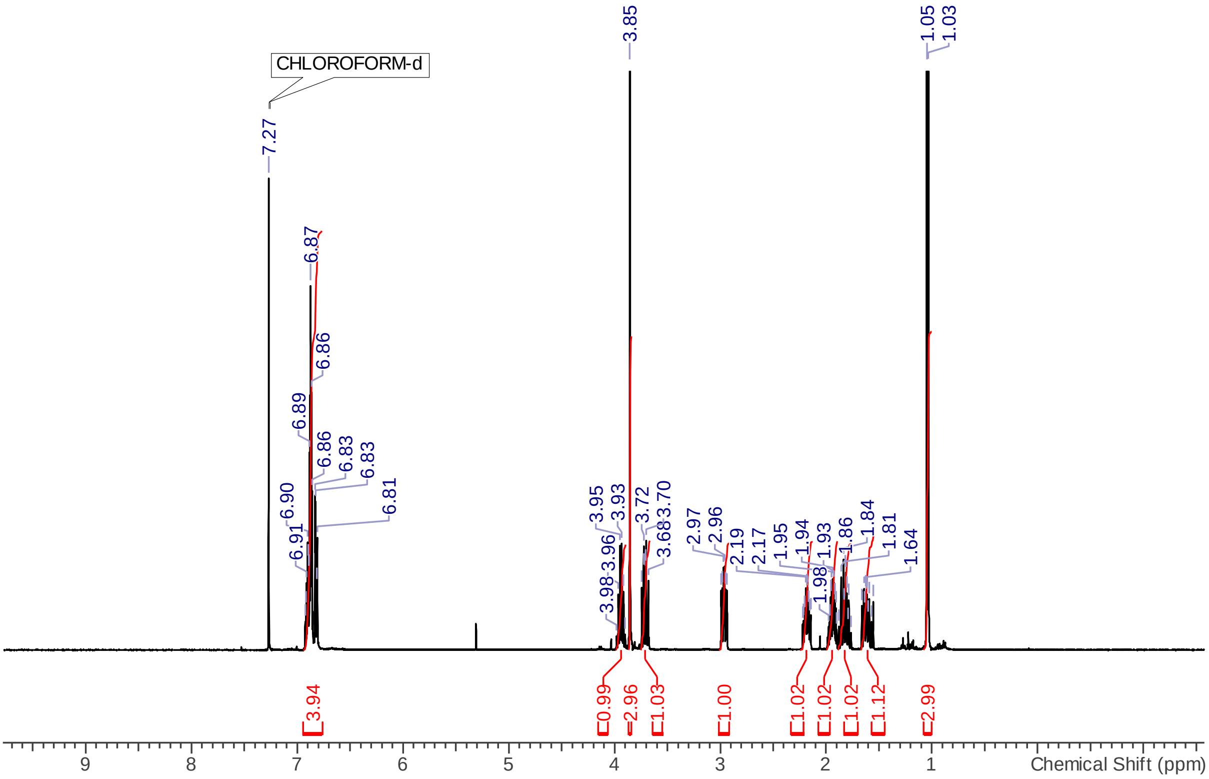

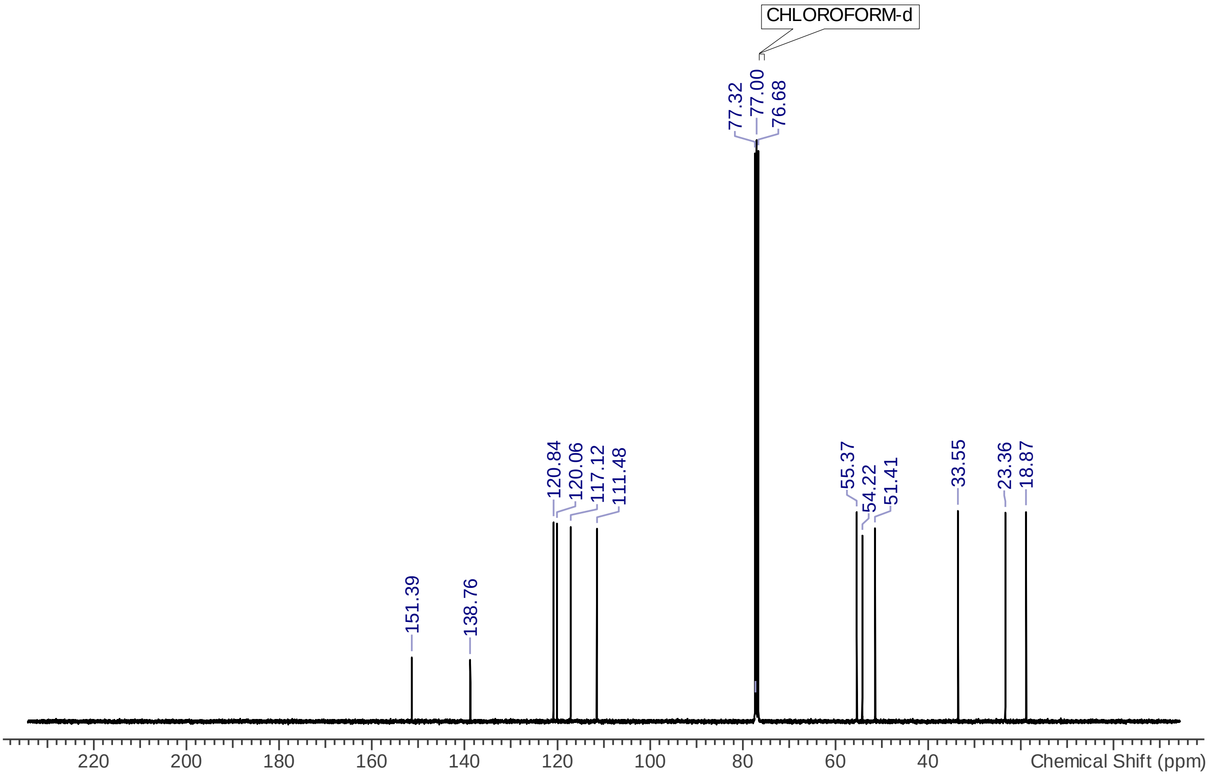

2-Methyl-1-(2-phenoxyphenyl)pyrrolidine 1i

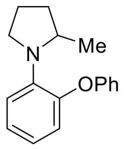

The title compound was prepared according to general procedure 1 using 2-phenoxyaniline (700 mg, 3.78 mmol), K_2CO_3 (1.57 g, 11.3 mmol), and 1,4-dibromopentane (1.03 mL, 8.57 mmol). Purification by flash column chromatography on silica gel (eluent = 5% EtOAc in pet. ether), gave the title compound **1i** as a brown oil (400 mg, 1.50 mmol, 40%). **R$_f$** = 0.50 (eluent = 5% EtOAc in pet. ether); **^{1}H NMR** (400 MHz, $CDCl_3$) δ_H = 7.31–7.24 (2H, m), 7.08 (1H, ddd, *J* = 8.3, 7.3, 1.6 Hz), 7.01 (1H, app. tt, *J* = 7.4, 1.0 Hz), 6.94 (1H, dd, *J* = 7.9, 1.6 Hz), 6.92–6.89 (2H, m), 6.86 (1H, dd, *J* = 8.2, 1.4 Hz), 6.77 (1H, app. td, *J* = 7.9, 1.6 Hz), 3.92 (1H, app. sxt, *J* = 6.3 Hz), 3.60 (1H, ddd, *J* = 9.3, 7.9, 7.7 Hz), 3.16 (1H, ddd, *J* = 9.3, 8.3, 3.8 Hz), 2.07 (1H, dtd, *J* = 11.7, 7.2, 4.3 Hz), 1.84 (1H, app. dtd, *J* = 11.7, 7.7, 3.7 Hz), 1.79–1.67 (1H, m), 1.53–1.45 (1H, m), 1.10 (3H, d, *J* = 6.3 Hz); **^{13}C NMR** (101 MHz, $CDCl_3$) δ_C = 158.3 (C), 145.2 (C), 141.4 (C), 129.3 (2×CH), 124.8 (CH), 122.2 (CH), 121.7 (CH), 118.6 (CH), 117.0 (CH), 116.7 (CH), 54.1 (CH), 51.4 (CH_2), 33.8 (CH_2), 23.8 (CH_2), 19.2 (CH_3); **HRMS** (ESI$^+$) calculated for $[C_{17}H_{19}NO]^+$ $(M+H)^+$ m/z: 254.1545; found 254.1555.

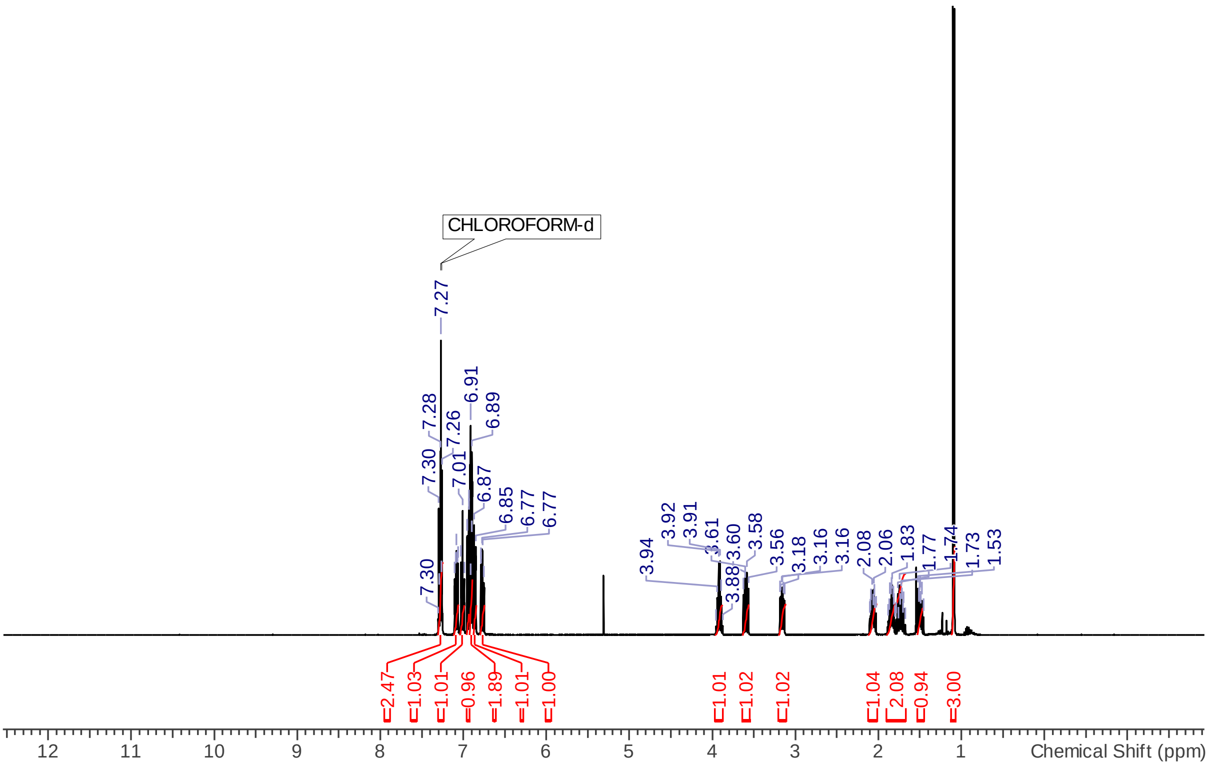

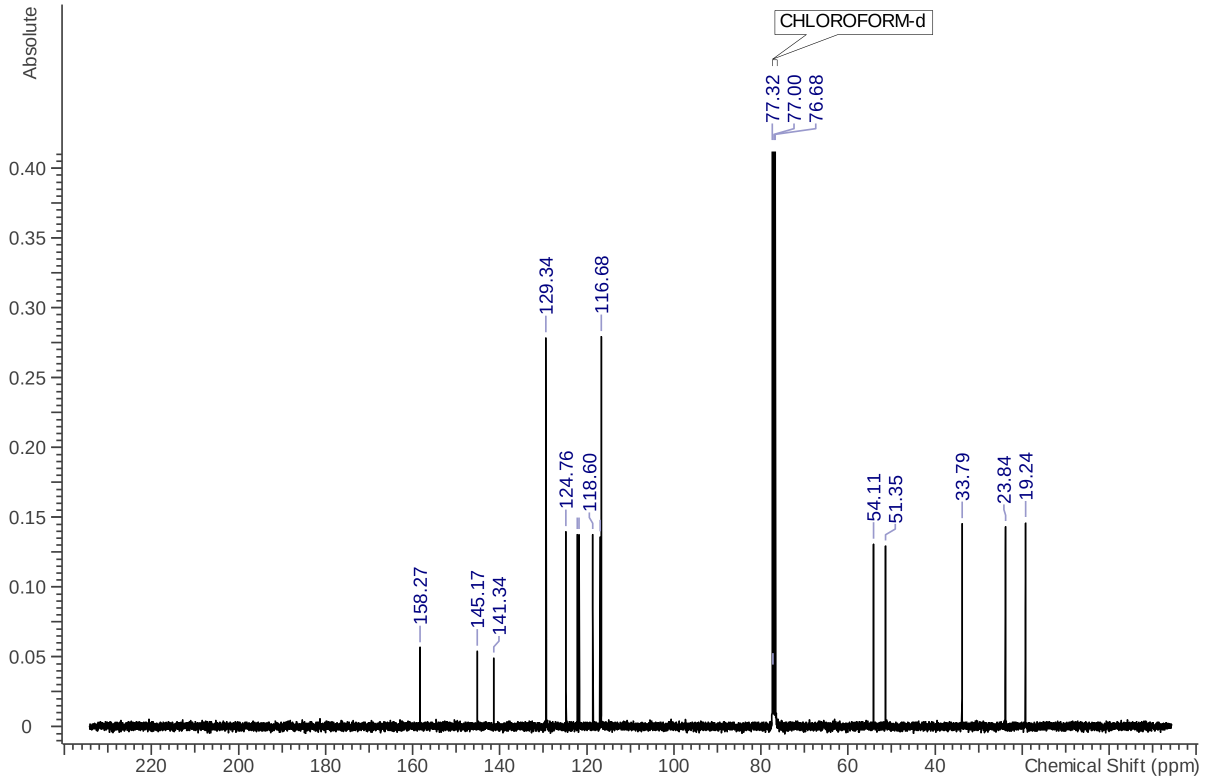

1-(4-Chlorophenyl)-2-methylpyrrolidine 1j

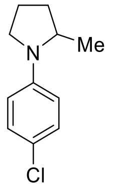

The title compound was prepared according to general procedure 2 using 4-chloroaniline (700 mg, 6.05 mmol), K_2CO_3 (1.90 g, 13.8 mmol), 1,4-dibromopentane (0.82 mL, 6.41 mmol), and DMF (6 mL). Purification by flash column chromatography on silica gel (eluent = 20% DCM in pet. ether), gave the title compound **1j** as a grey solid (568 mg, 2.91 mmol, 53%). **R**$_f$ = 0.4 (eluent = 20% DCM in pet. ether); ^{1}**H NMR** (400 MHz, $CDCl_3$) δ_H = 7.20–7.10 (2H, m), 6.53–6.45 (2H, m), 3.83 (1H, app. quind, *J* = 6.2, 1.9 Hz), 3.44–3.35 (1H, m), 3.19–3.09 (1H, m), 2.15–1.93 (3H, m), 1.77–1.67 (1H, m), 1.16 (3H, d, *J* = 6.2 Hz); ^{13}**C NMR** (101 MHz, $CDCl_3$) δ_C = 145.7 (C), 128.9 (2×CH), 119.9 (C), 112.8 (2×CH), 53.8 (CH), 48.3 (CH_2), 33.1 (CH_2), 23.3 (CH_2), 19.1 (CH_3). Spectroscopic data in accordance with that stated in the literature.[5]

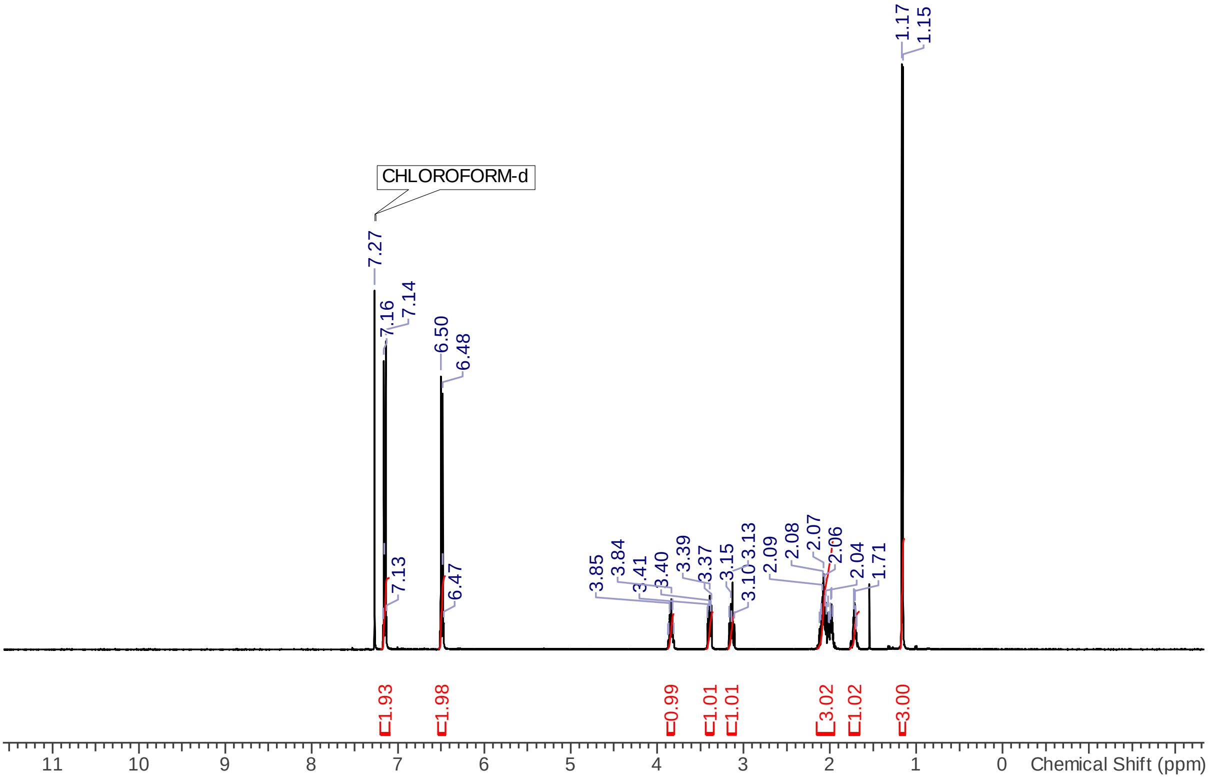

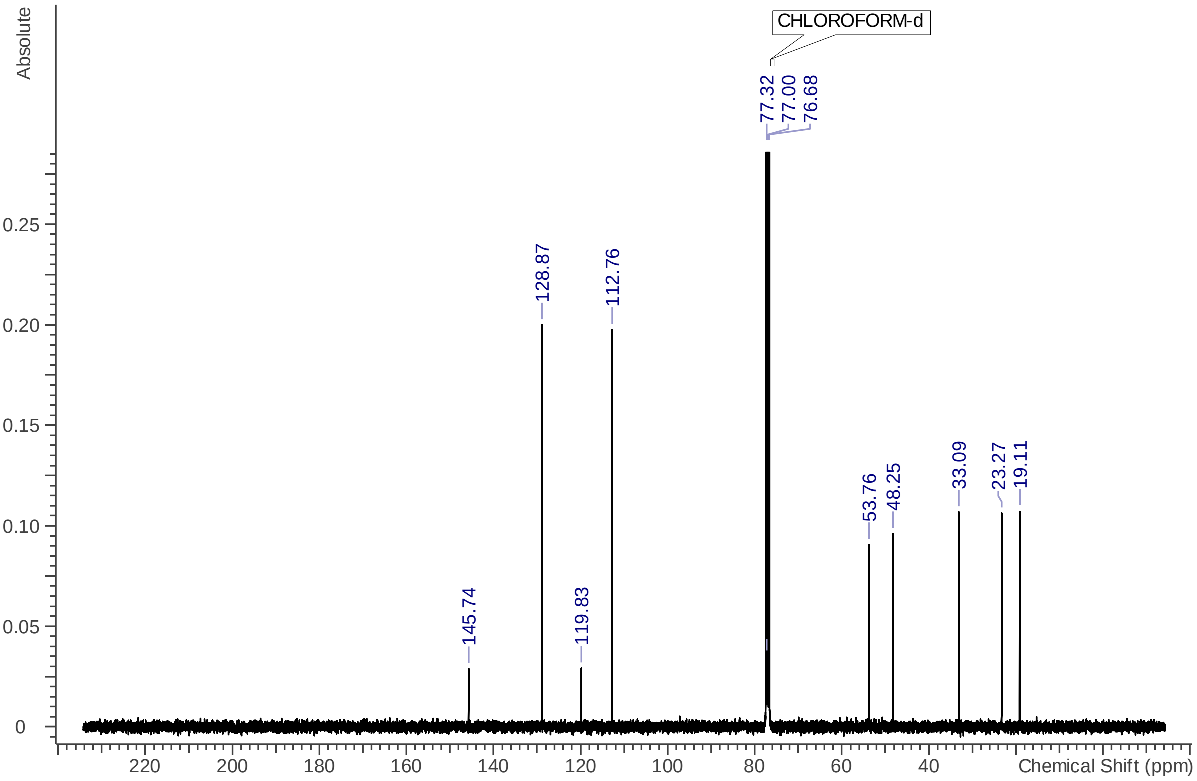

1-(4-Bromophenyl)-2-methylpyrrolidine 1k

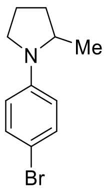

The title compound was prepared according to general procedure 2 using 4-bromoaniline (1.00 g, 5.84 mmol), K_2CO_3 (2.42 g, 17.5 mmol), 1,4-dibromopentane (0.82 mL, 6.41 mmol), and DMF (9 mL). Purification by flash column chromatography on silica gel (eluent = 20% DCM in pet. ether), gave the title compound **1k** as a white solid (670 mg, 2.28 mmol, 48%). **R$_f$** = 0.6 (eluent = 20% DCM in pet. ether); **^{1}H NMR** (400 MHz, $CDCl_3$) δ_H = 7.32–7.29 (2H, m), 6.49–6.41 (2H, m), 3.84 (1H, app. quind, *J* = 6.2, 1.6 Hz) 3.43–3.33 (1H, m), 3.18–3.07 (1H, m), 2.16–1.95 (3H, m), 1.76–1.66 (1H, m), 1.16 (3H, d, *J* = 6.2 Hz); **^{13}C NMR** (101 MHz, $CDCl_3$) δ_C = 146.1 (C), 131.7 (2×CH), 113.3 (2×CH), 106.8 (C), 53.7 (CH), 48.2 (CH_2), 33.1 (CH_2), 23.3 (CH_2), 19.0 (CH_3); Spectroscopic data in accordance with that stated in the literature.[5]

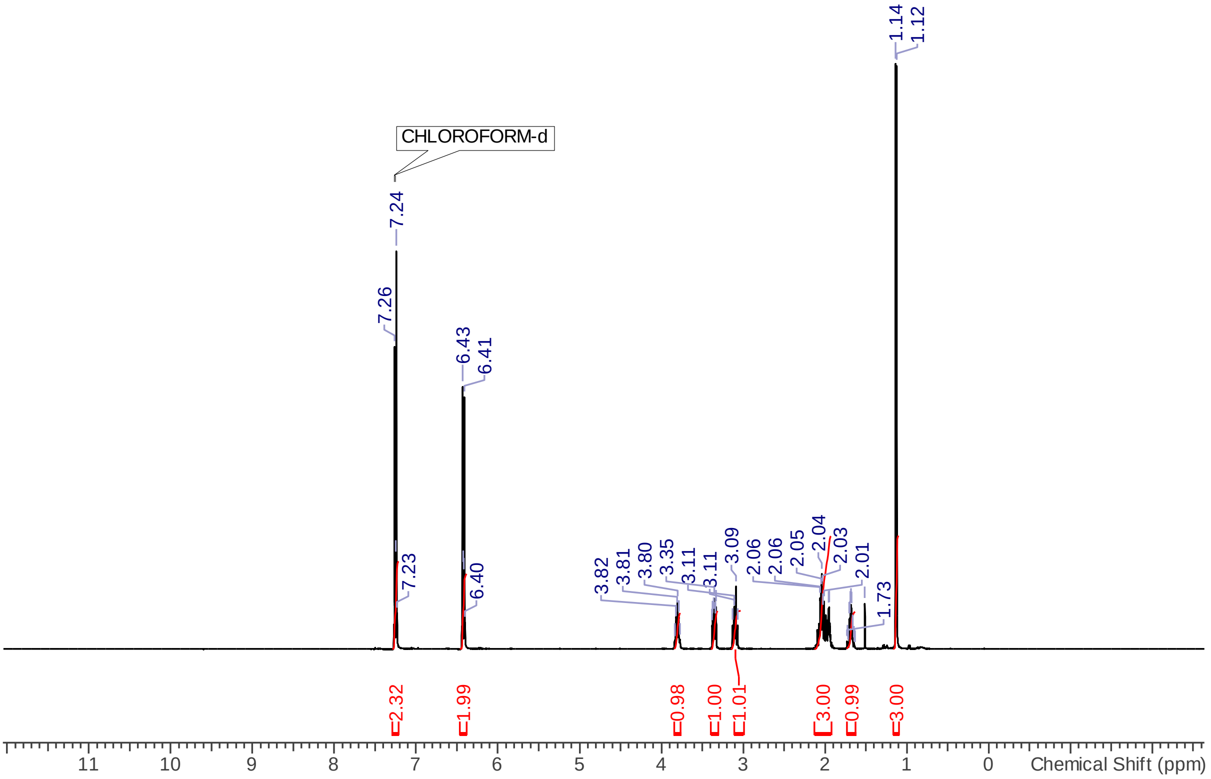

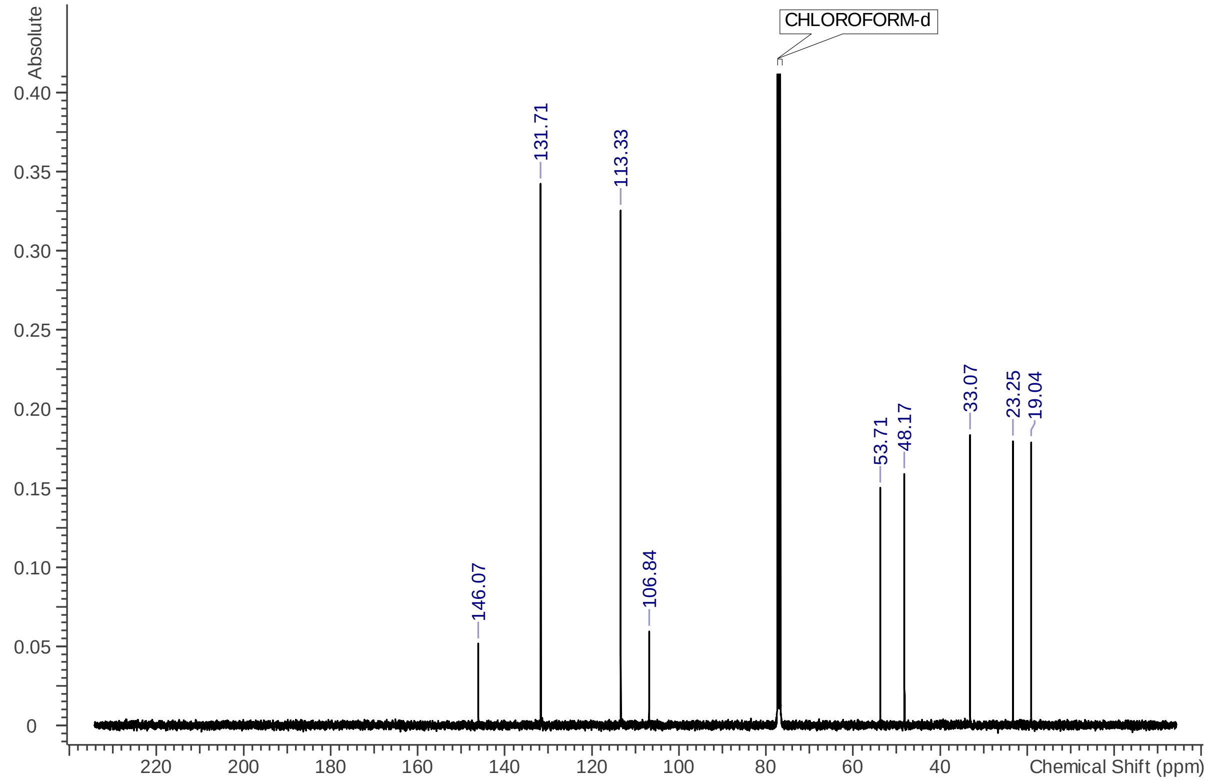

2-Methyl-1-(*p*-tolyl)pyrrolidine 1l

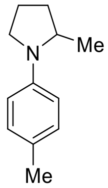

The title compound was prepared according to general procedure 2 using 4-toluidine (800 mg, 7.47 mmol), K_2CO_3 (2.56 g, 18.5 mmol), 1,4-dibromopentane (1.10 mL, 8.14 mmol), and DMF (9 mL). Purification by flash column chromatography on silica gel (eluent = 10% EtOAc in pet. ether), gave the title compound **1l** as a red oil (948 mg, 5.41 mmol, 72%). $\mathbf{R_f}$ = 0.4 (eluent = 10% EtOAc in pet. ether); **^{1}H NMR** (400 MHz, $CDCl_3$) δ_H = 7.09–7.00 (2H, m), 6.56–6.50 (2H, m), 3.85 (1H, app. quind, *J* = 6.3, 1.9 Hz), 3.47–3.37 (1H, m), 3.20–3.08 (1H, m), 2.26 (3H, s), 2.16–1.92 (3H, m), 1.75-1.63 (1H, m), 1.18 (3H, d, *J* = 6.3 Hz); **^{13}C NMR** (101 MHz, $CDCl_3$) δ_C = 145.3 (C), 129.7 (C), 124.2 (CH), 111.9 (CH), 53.7 (CH), 48.5 (CH_2), 33.2 (CH_2), 23.4 (CH_2), 20.2 (CH_3), 19.5 (CH_3); Spectroscopic data in accordance with that stated in the literature.[5]

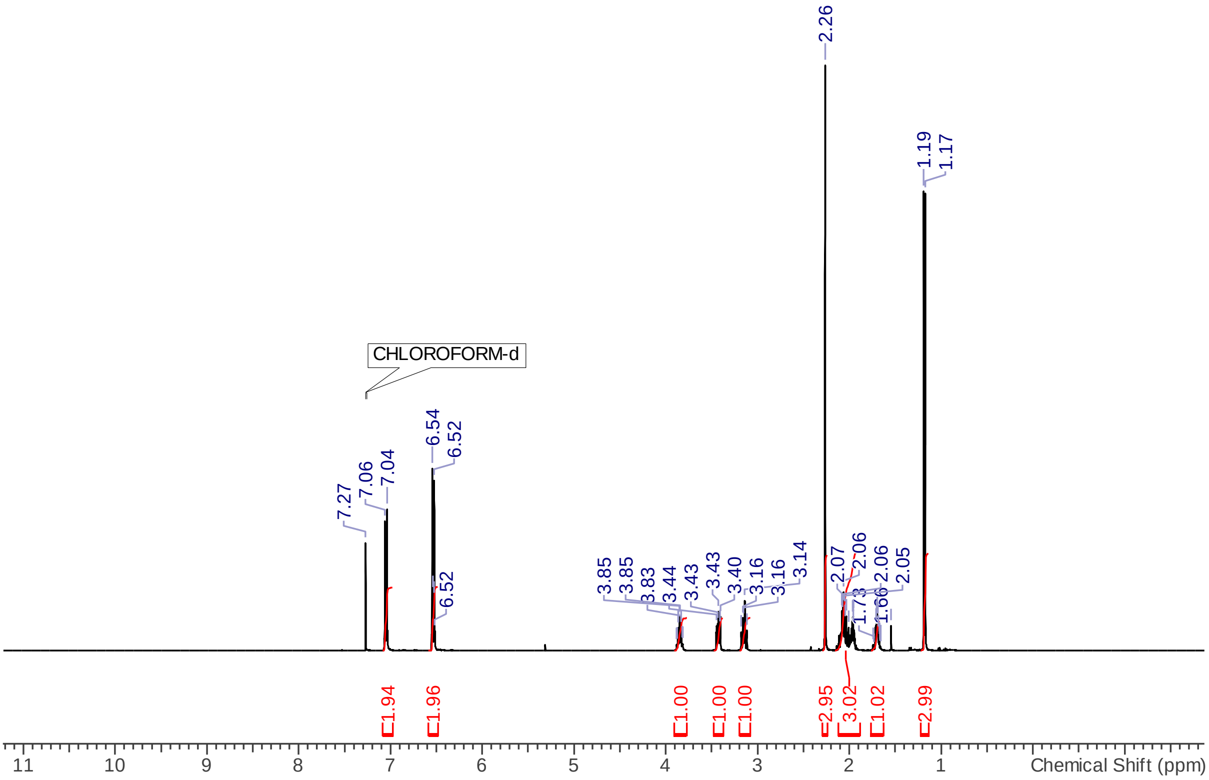

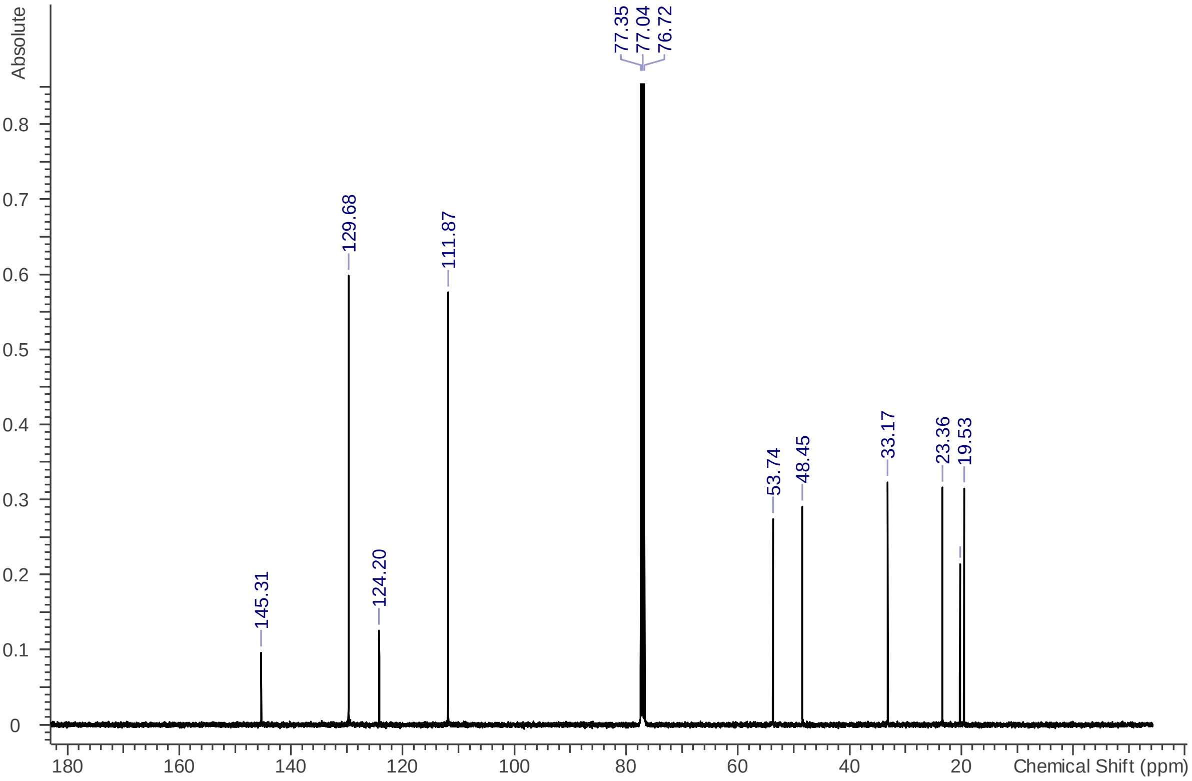

1-(4-methoxyphenyl)-2-methylpyrrolidine 1m

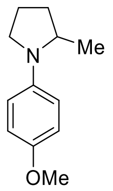

The title compound was prepared according to general procedure 1 using 4-methoxyaniline (0.92 mL, 8.10 mmol), K_2CO_3 (3.36 g, 24.3 mmol), and 1,4-dibromopentane (1.06 mL, 8.91 mmol). Purification by flash column chromatography on silica gel (eluent = 10% EtOAc in pet. ether), gave the title compound **1m** as a black oil (758 mg, 3.97 mmol, 45%). **R_f** = 0.5 (eluent = 10% EtOAc in pet. Ether); **^{1}H NMR** (400 MHz, $CDCl_3$) δ_H 6.90–6.82 (2H, m), 6.60–6.53 (2H, m), 3.84–3.77 (1H, m), 3.77 (3H, s) 3.45–3.37 (1H, m), 3.12 (1H, app. dd, *J* = 15.9, 8.6 Hz) 2.12–1.90 (3H, m) 1.74–1.63 (1H, m), 1.17 (3H, d, J = 6.1 Hz); **^{13}C NMR** (101 MHz, $CDCl_3$) δ_C = 150.9 (C), 142.7 (C), 115.4 (2×CH), 113.0 (2×CH), 56.3 (CH_3), 54.4 (CH), 49.2 (CH_2), 33.5 (CH_2), 23.7 (CH_2), 20.0 (CH_2); Spectroscopic data in accordance with that stated in the literature.[5]

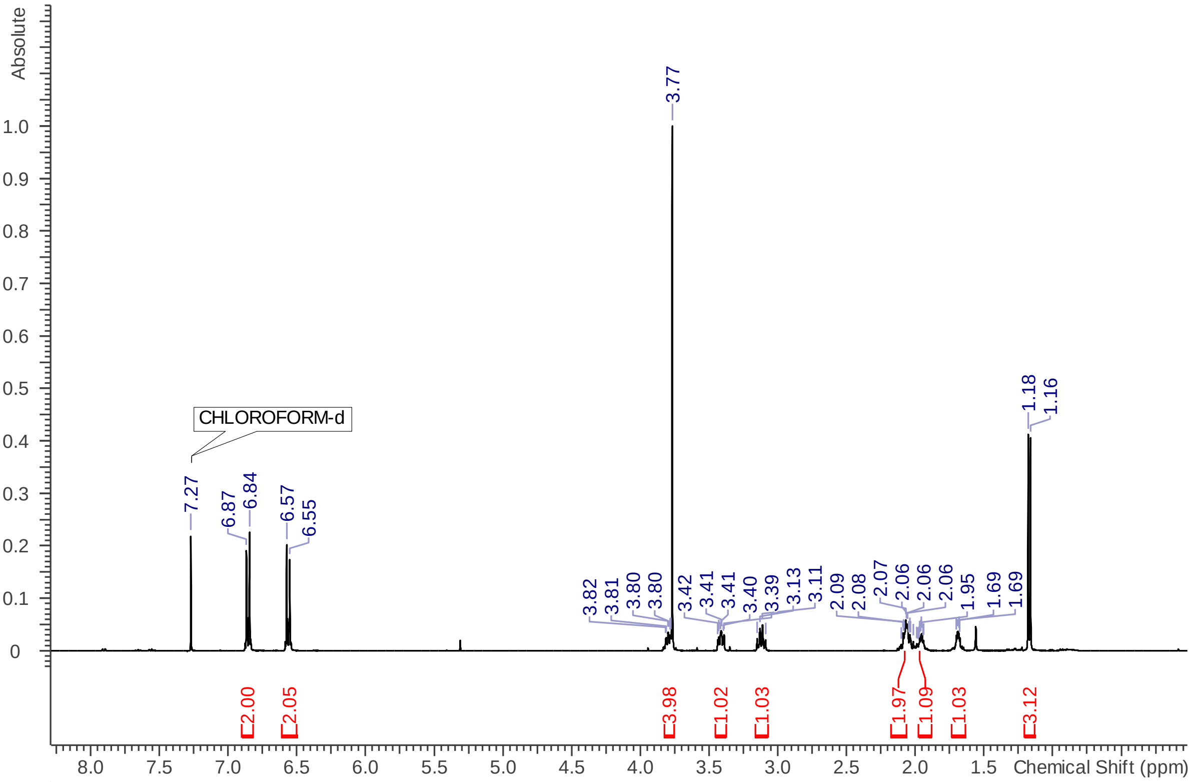

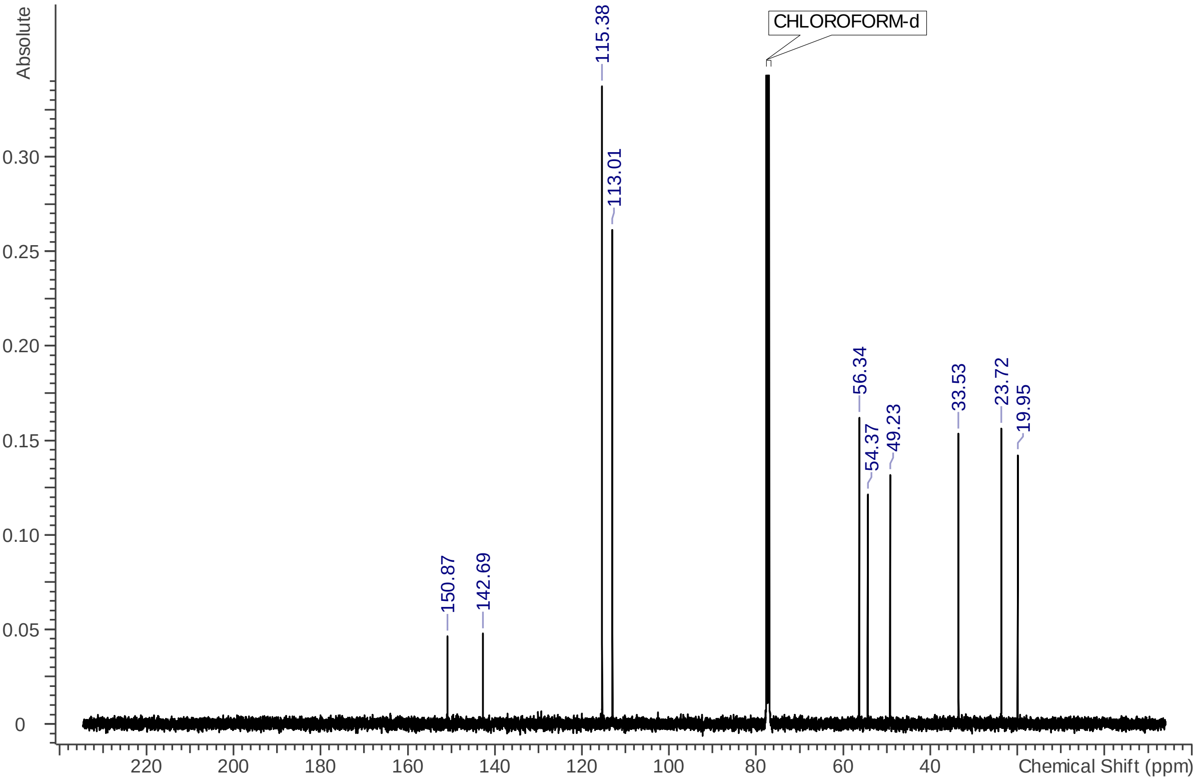

3-(2,5-dimethylpyrrolidin-1-yl)benzoic acid 1n

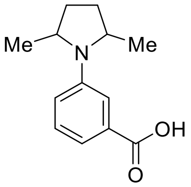

The title compound was prepared according to general procedure 3 using 3-aminobenzoic acid (2.00 g, 14.6 mmol), AcOH (0.92 mL, 16 mmol), KOH (992 mg, 17.7 mmol), 2,5-hexadione (1.71 mL, 14.6 mmol) and $NaBH_3CN$ (918 mg, 14.6 mmol). Purification by flash column chromatography on silica gel (eluent = 10% MeOH in 3:2 pet. ether:DCM), gave the title compound **1n** as a white solid (1.25 g, 5.70 mmol, 39%, 92:8 mixture of diastereomers). R_f = 0.35 (eluent = 10% MeOH in 3:2 pet. ether:DCM); **^{1}H NMR** (400 MHz, $CDCl_3$) **Common signals:** δ_H = 12.57 (1H, br. s), 7.40–7.17 (3H, m), 6.83–6.73 (1H, m); **Major diastereomer:** δ_H = 4.01 (2H, app. quin, *J* = 6.1 Hz), 2.27–2.13 (2H, m), 1.68–1.55 (2H, m), 1.08 (6H, d, *J* = 6.1 Hz); **Minor diastereomer:** δ_H = 3.85–3.72 (2H, m), 2.08–1.98 (2H, m), 1.76–1.68 (2H, m), 1.26 (6H, d, *J* = 6.1 Hz); **^{13}C NMR** (101 MHz, $CDCl_3$) δ_C = 173.42 (C=O), 173.36 (C=O)*, 147.2 (C)*, 145.2 (C), 123.0 (C), 129.9 (C)*, 129.1 (CH), 129.0 (CH)*, 118.4 (CH), 117.0 (CH)*, 116.9 (C)*, 116.3 (CH), 114.5 (CH), 113.1 (CH)*, 55.9 (2×CH)*, 52.9 (2×CH), 32.2 (2×CH_2)*, 30.3 (2×CH_2), 21.5 (2×CH_3)*, 17.9 (2×CH_3). **HRMS** (ESI$^+$): calculated for $[C_{13}H_{18}NO_2]^+$ $(M+H)^+$ m/z: 220.1338; found 220.1340. **Signals attributed to minor diastereomer.*

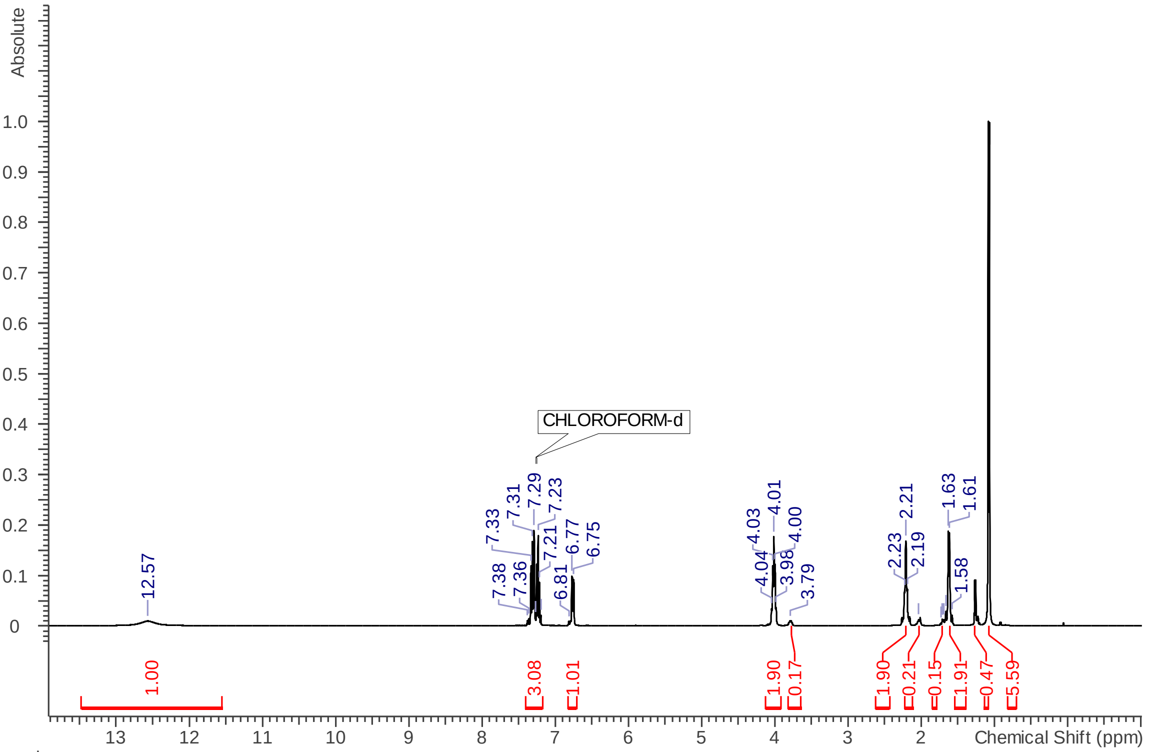

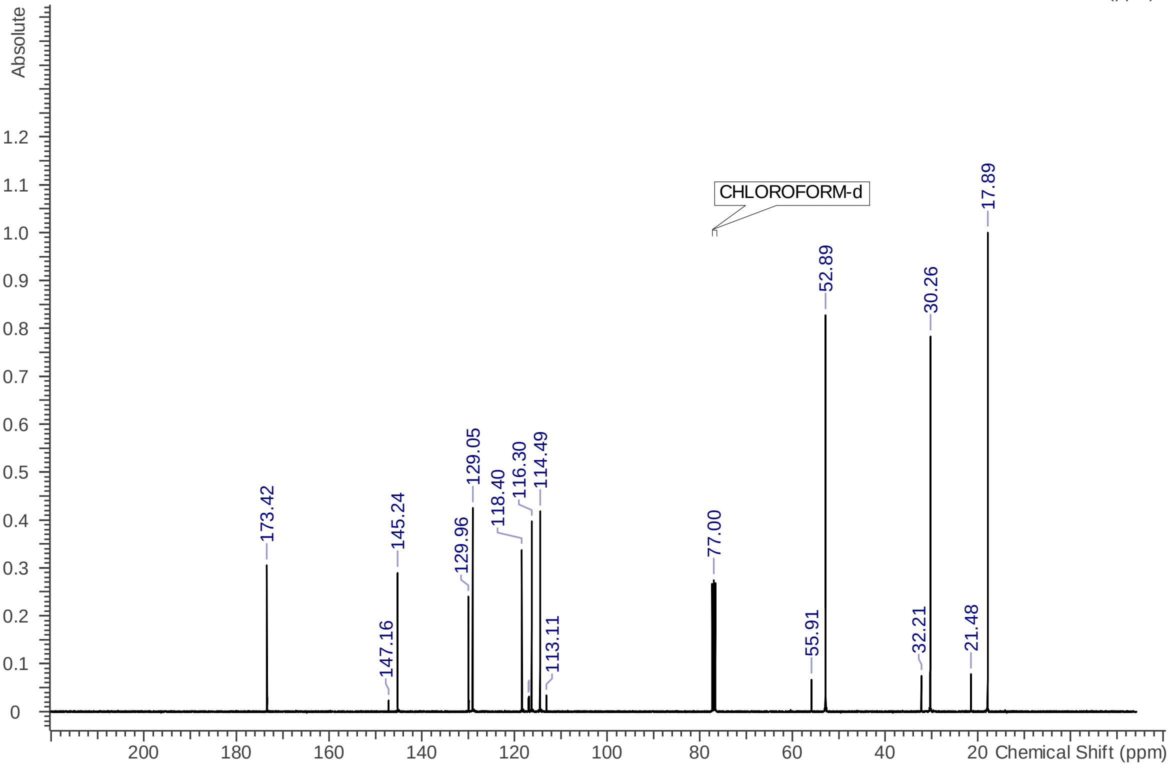

1-(3-(((*tert*-Butyldimethylsilyl)oxy)methyl)phenyl)-2,5-dimethylpyrrolidine 1o

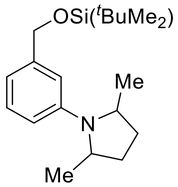

A 20 mL J. Youngs ampoule equipped with stirrer bar was charged with 3-(2,5-dimethylpyrrolidin-1-yl)benzoic acid **1n** (600 mg, 2.74 mmol) and THF (14 mL) under a nitrogen atmosphere. The reaction mixture was cooled to 0 °C, before $LiAlH_4$ (200 mg, 5.27 mmol) was added portionwise. The reaction was sealed and left to stir and gradually warm to room temperature over 17 h, before the reaction was cooled to 0 °C and quenched by the sequential dropwise addition of H_2O (0.15 mL), KOH (aq., 15%, 0.15 mL), and H_2O (0.5 mL). Upon warming to ambient temperature the mixture was stirred for 30 mins, before being filtered through Celite™. The organics were concentrated in vacuo to give intermediate (3-(2,5-dimethylpyrrolidin-1-yl)phenyl)methanol as a colourless oil.

A 10 mL round bottomed flask equipped with stirrer bar was charged with (3-(2,5-dimethylpyrrolidin-1-yl)phenyl)methanol (300 mg, 1.46 mmol), imidazole (298 mg, 4.38 mmol), TBDMSCl (0.35 mL, 2.04 mmol), and DMF (4 mL), before being stirred at room temperature overnight. Upon completion, the reaction was diluted with water, before being extracted with EtOAc. The organic layer was dried with $MgSO_4$, filtered, and concentrated in vacuo. Purification by flash column chromatography on silica gel (eluent = 2% EtOAc in pet. ether) gave the title compound **1o** as a colourless oil (285 mg, 0.89 mmol, 61%). $\mathbf{R_f}$ = 0.34 (eluent = 2% EtOAc in pet. ether, 74:26 mixture of diastereomers); **^{1}H NMR** (400 MHz, $CDCl_3$) **Common signals:** δ_H = 7.22–7.12 (1H, m); **Major diastereomer: ^{1}H NMR** (400 MHz, $CDCl_3$) δ_H = 6.64–6.60 (1H, s), 6.55–6.51 (1H, m), 6.47 (1H, dd, *J* = 8.2, 2.4 Hz), 4.71 (2H, s), 4.02 (2H, app. quin, *J* = 6.3 Hz), 2.28–2.20 (2H, m), 1.68–1.62 (2H, m), 1.11 (6H, d, *J* = 6.3 Hz), 0.96 (9H, s), 0.12 (6H, s); **Minor diastereomer:** δ_H = 6.68 (1H, s), 6.60–6.56 (1H, m), 6.50 (1H, dd, *J* = 8.2, 2.4 Hz), 4.72 (2H, s), 3.79 (2H, app. dq, *J* = 10.9, 5.7 Hz), 2.10–2.00 (2H, m), 1.75–1.64 (2H, m), 1.28 (6H, d, *J* = 6.1 Hz), 0.97 (9H, s), 0.12 (6H, s); **^{13}C NMR** (101 MHz, $CDCl_3$) δ_C = 147.4 (C)*, 145.3 (C), 142.30 (C)*, 142.25 (C), 128.83 (CH), 128.80 (CH)*, 112.9 (CH)*, 112.3 (CH), 111.9 (CH), 110.9 (CH), 110.4 (CH)*, 109.5 (CH)*, 65.3 (CH_2), 65.2 (CH_2)*, 55.9 (2×CH)*, 52.7 (2×CH), 32.3 (2×CH_2)*, 30.3 (2×CH_2), 26.0 (3×CH_3)†, 21.8 (2×CH_3)*, 18.4 (C)†, 18.2 (2×CH_3), −5.24 (2×CH_3)†; **HRMS** (ESI$^+$): calculated for $[C_{19}H_{34}NOSi]^+$ $(M+H)^+$ m/z: 320.2410; found 320.2410. **Signals attributed to minor diastereomer.* †*Signals attributed to both major and minor diastereomers.*

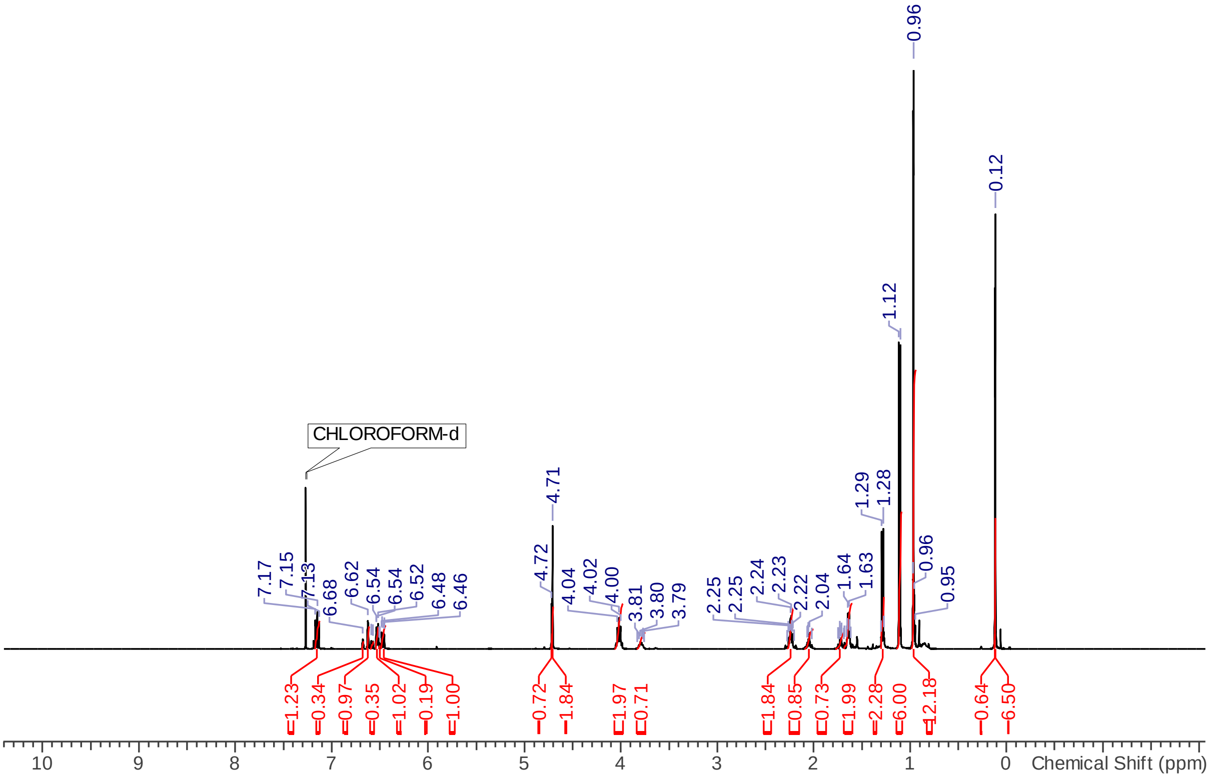

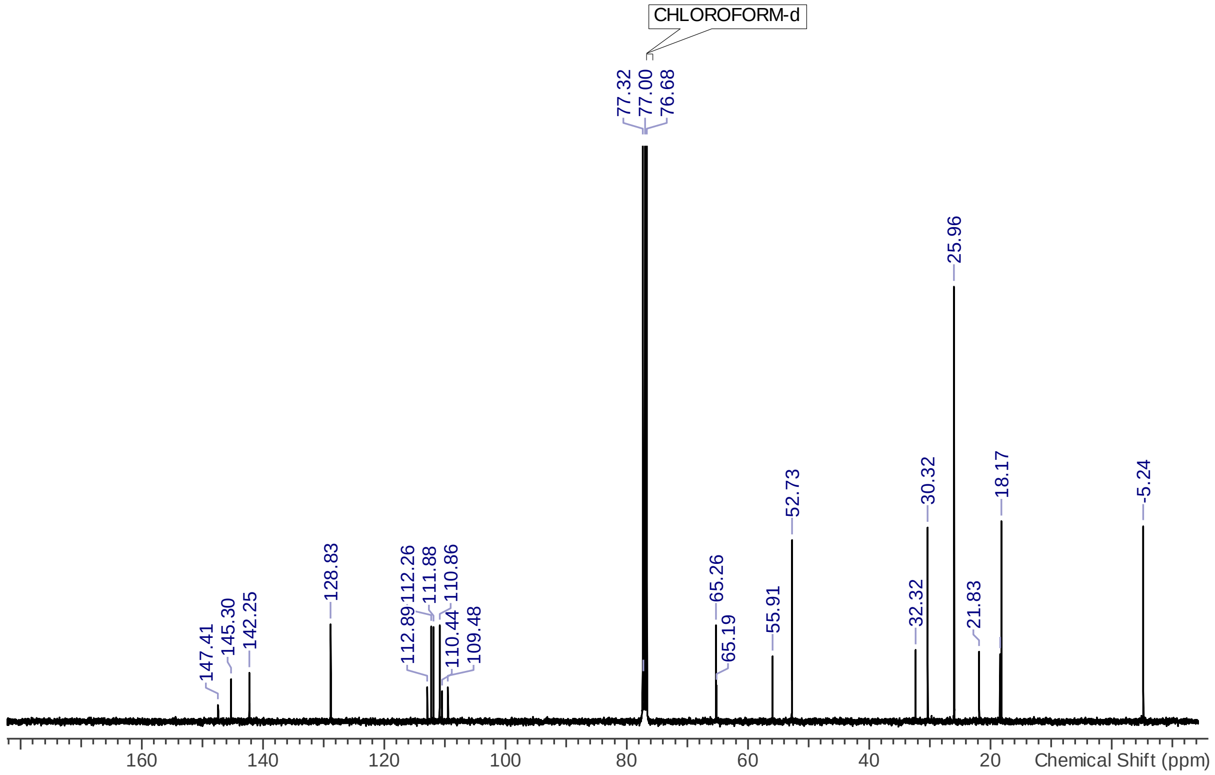

CHLOROFORM-d
77.32
77.00
76.68
25.96
52.73
30.32
18.17
5.24
128.83
112.26
111.88
110.86
112.89
110.44
109.48
147.41
145.30
142.25
65.26
65.19
55.91
32.32
21.83
160
140
120
100
80
60
40
20
Chemical Shift (ppm)

4-Bromo-2-*tert*-butylaniline S3

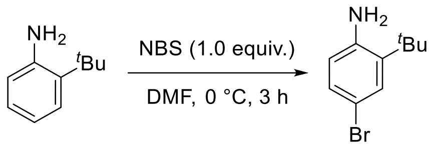

2-*tert*-Butylaniline, (1.0 mL, 6.7 mmol) was dissolved in DMF (20 mL) and cooled to 0 °C (using an ice bath). *N*-Bromosuccinimide (1.19 g, 6.69 mmol) was then added dropwise, before the reaction was stirred for 3 h, maintained at 0 °C. Upon completion, the reaction was diluted with water, before being extracted with Et_2O (3 × 30 mL). The combined organics were then washed with brine (50 mL), dried with $MgSO_4$, and filtered before the solvent was removed *in vacuo*. Purification by flash column chromatography on silica gel (eluent = 10%–25% EtOAc in pet. ether) gave the title compound **S3** as a red oil (1.41 g, 6.18 mmol, 92%). $\mathbf{R_f}$ = 0.29 (eluent = 10% EtOAc in pet. ether); **^{1}H NMR** (400 MHz, $CDCl_3$) δ_H = 7.31 (1H, d, *J* = 2.3 Hz), 7.12 (1H, dd, *J* = 8.4, 2.3 Hz), 6.52 (1H, d, *J* = 8.4 Hz), 3.82 (2H, br. s), 1.41 (9H, s); **^{13}C NMR** (101 MHz, $CDCl_3$) δ_C = 143.6 (C), 135.8 (C), 129.5 (2×CH), 119.2 (CH), 110.7 (C), 34.4 (C), 29.3 (3×CH_3); **HRMS** (ESI$^+$) calculated for $[C_{10}H_{15}{}^{79}BrN]^+$ (M+H)$^+$ m/z: 228.0383; found 228.0390; calculated for $[C_{10}H_{15}{}^{81}BrN]^+$ (M+H)$^+$ m/z: 230.0362; found 230.0370.

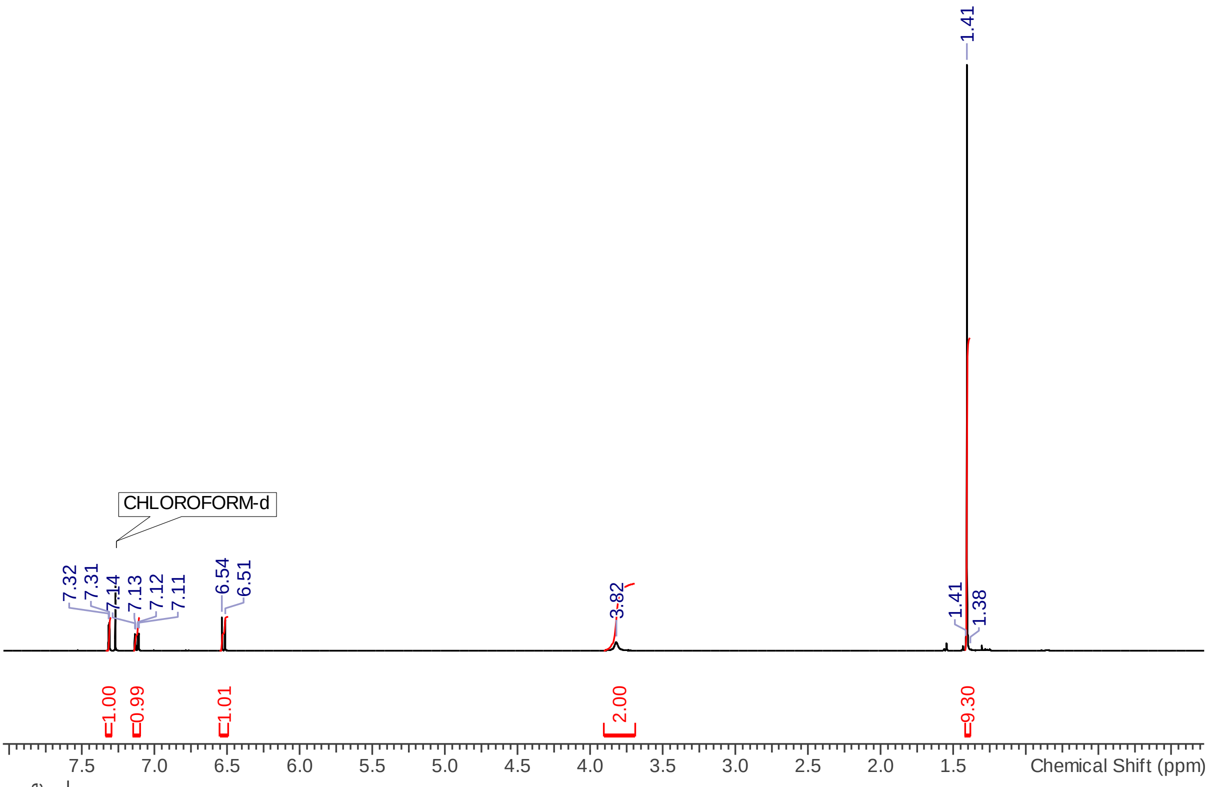

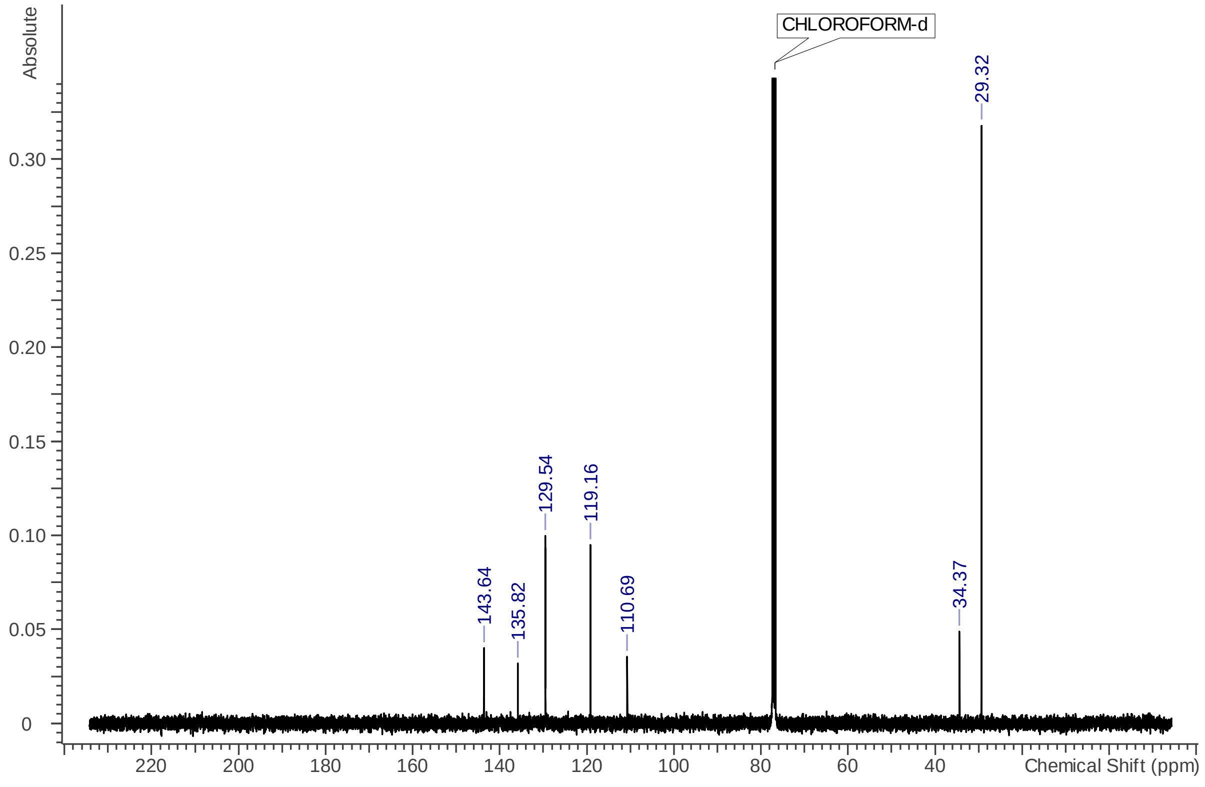

1-(4-Bromo-2-(*tert*-butyl)phenyl)pyrrolidine, 1p

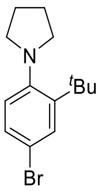

The title compound was prepared according to general procedure 1 using 4-bromo-2-*tert*-butylaniline, **S3** (1.41 g, 6.18 mmol), K_2CO_3 (1.94 g, 14.0 mmol), and 1,4-dibromobutane (1.46 mL, 12.4 mmol). Purification by flash column chromatography on silica gel (eluent = 2.5%–20% EtOAc in pet. ether), gave the title compound **1p** as an orange oil (1.04 g, 3.69 mmol, 59%). **R$_f$** = 0.24 (eluent = 2.5% EtOAc in pet. ether); **^{1}H NMR** (400 MHz, $CDCl_3$) δ_H = 7.46 (1H, d, J = 2.4 Hz), 7.33 (1H, dd, J = 8.4, 2.4 Hz), 7.22 (1H, d, J = 8.4 Hz), 2.99–2.87 (4H, m), 1.96–1.85 (4H, m), 1.40 (9H, s); **^{13}C NMR** (101 MHz, $CDCl_3$) δ_C = 151.6 (C), 150.8 (C), 130.0 (CH), 129.9 (CH), 128.6 (CH), 119.0 (C), 56.2 (2×CH_2), 35.6 (C), 30.8 (3×CH_3), 24.6 (2×CH_2); **HRMS** (ESI$^+$) calculated for $[C_{14}H_{21}{}^{79}BrN]^+$ $(M+H)^+$ m/z: 282.0852; found 282.0853; calculated for $[C_{14}H_{21}{}^{81}BrN]^+$ $(M+H)^+$ m/z: 284.0832; found 284.0838.

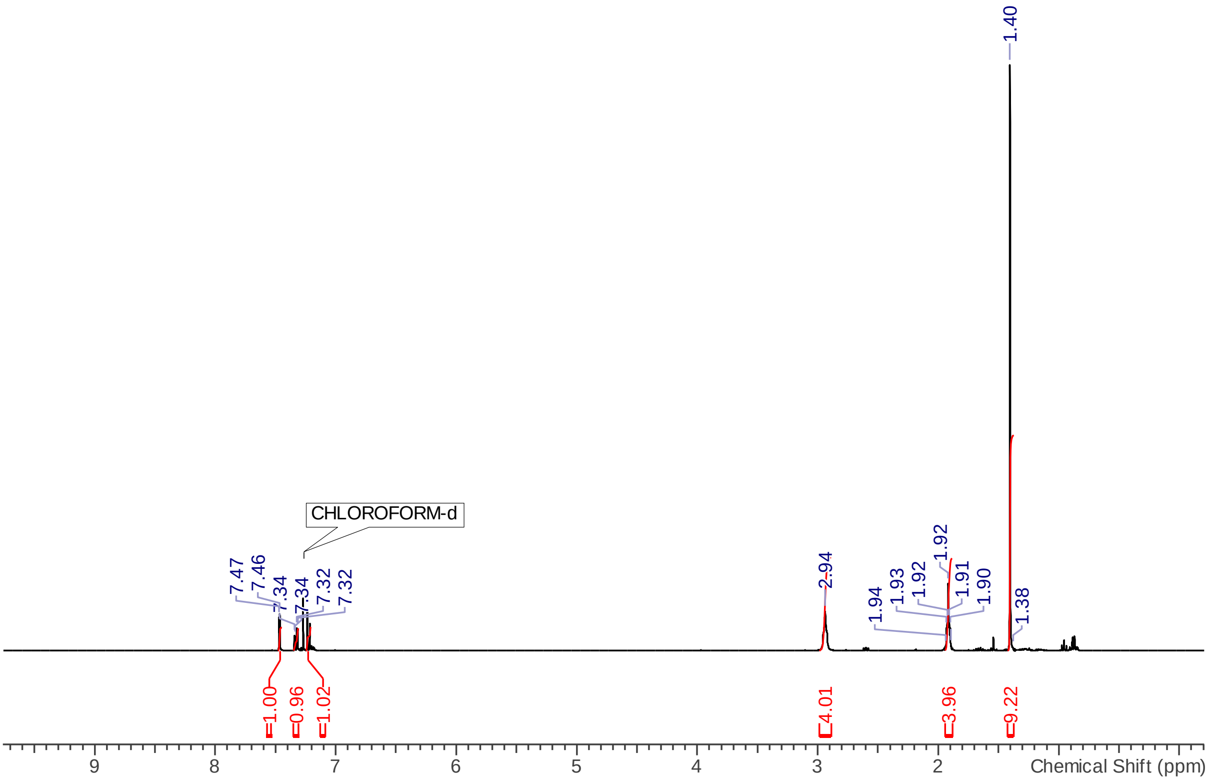

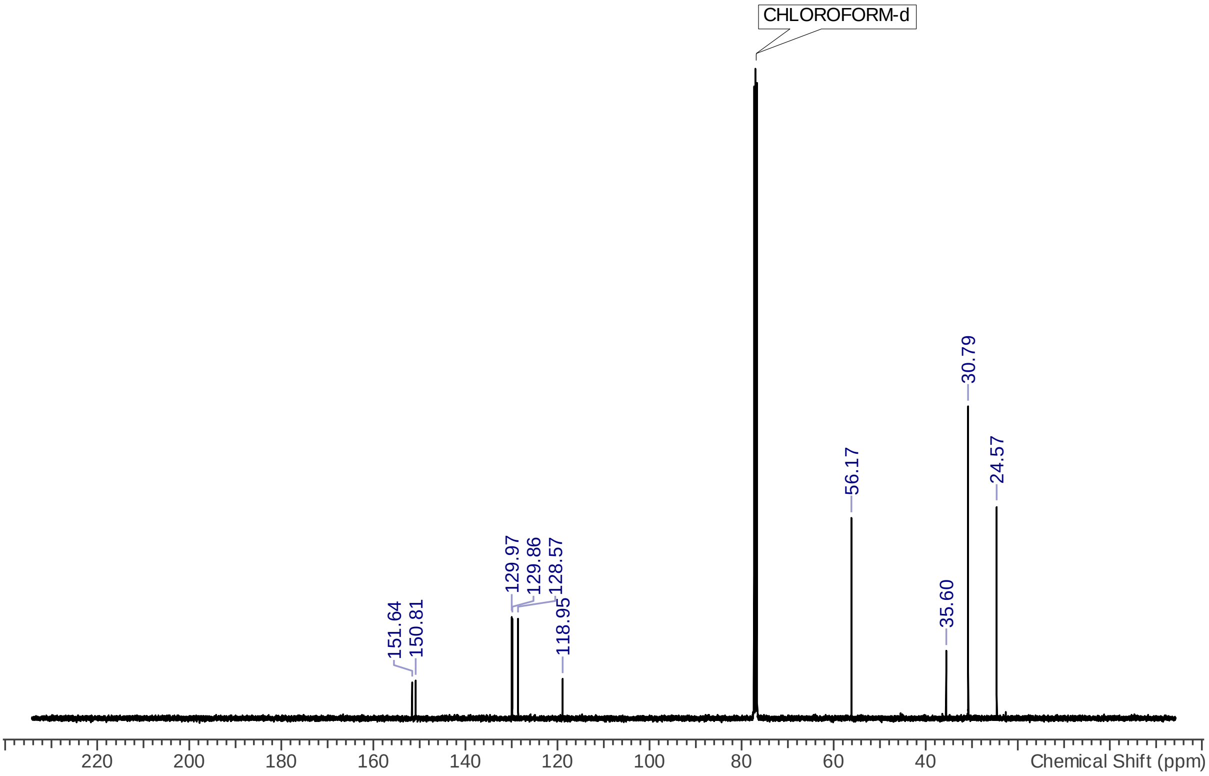

1-(2-(*tert*-Butyl)-4-(4,4,5,5-tetramethyl-1,3,2-dioxaborolan-2-yl)phenyl)pyrrolidine, 1q

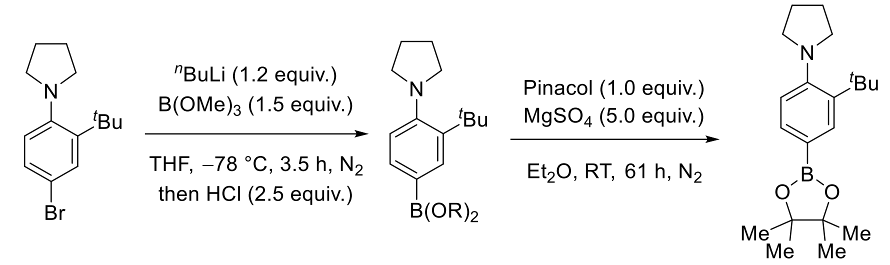

A J. Youngs ampoule equipped with stirrer bar was charged with 1-(4-Bromo-2-(*tert*-butyl)phenyl)pyrrolidine **1p** (303 mg, 1.07 mmol), which was dissolved in THF (4 mL) under a nitrogen atmosphere and cooled to −78 °C (using an acetone/dry ice bath). *n*-Butyllithium (0.83 mL, 1.6 M in hexanes, 1.3 mmol) was added slowly, causing a slight colour change from colourless to pale yellow. The reaction mixture was stirred at −78 °C for 1 h, before trimethylborate (0.18 mL, 1.6 mmol) was added slowly. After stirring for 15 mins at −78 °C, the reaction mixture was allowed to warm to room temperature, and stirred for a further 2.5 h. The reaction mixture was cooled to −78 °C once more, before HCl (aq., 1M, 2.7 mL, 2.7 mmol) was added slowly. The temperature was then raised to 0 °C (using an ice bath), and allowed to stir for 20 mins. The reaction was then diluted with water (20 mL) and extracted with EtOAc (2 × 20 mL). The combined organics were then washed with water, dried over $MgSO_4$, filtered and concentrated *in vacuo* to yield a crude yellow residue - which was shown to be a mixture of 2/3 different species by ^{11}B NMR.

A 25 mL round bottomed flask equipped with septa and stirrer bar was then charged with the crude residue, which was dissolved in dry Et_2O under a nitrogen atmosphere, before pinacol (0.13 mL, 1.07 mmol) was added, and the mixture allowed to stir at room temperature for 1 h. $MgSO_4$ (640 mg, 5.32 mmol) was then added, and the reaction stirred for 60 h. The reaction mixture was filtered, before being concentrated *in vacuo*. Purification by flash column chromatography on silica gel (eluent = 0%–10% EtOAc in 1:1 pet. ether:DCM) gave the title compound **1q** as a white amorphous solid (90 mg, 0.27 mmol, 26%). **R$_f$** = 0.47 (eluent = 50% DCM in petroleum ether); **^{1}H NMR** (400 MHz, $CDCl_3$) δ_H = 7.83 (1H, d, *J* = 1.5 Hz), 7.69 (1H, dd, *J* = 7.8, 1.5 Hz), 7.37 (1H, d, *J* = 7.8 Hz), 3.03–2.90 (4H, m), 1.98–1.85 (4H, m), 1.45 (9H, s), 1.34 (12H, s). **^{11}B NMR (**128 MHz, $CDCl_3$) δ_B = 31.2. **^{13}C NMR** (101 MHz, $CDCl_3$) δ_C = 155.1 (C), 148.2 (C), 133.7 (CH), 133.2 (CH), 126.1 (CH), 83.6 (2×C), 56.1 (2×CH_2), 35.5 (2×C), 31.1 (3×CH_3), 24.8 (4×CH_3), 24.7 (2×CH_2). Carbon attached to boron not seen due to coupling of quaternary carbon. **HRMS** (ESI$^+$) calculated for $[C_{20}H_{33}{}^{11}BNO_2]^+$ $(M+H)^+$ m/z: 330.2604; found 330.2614.

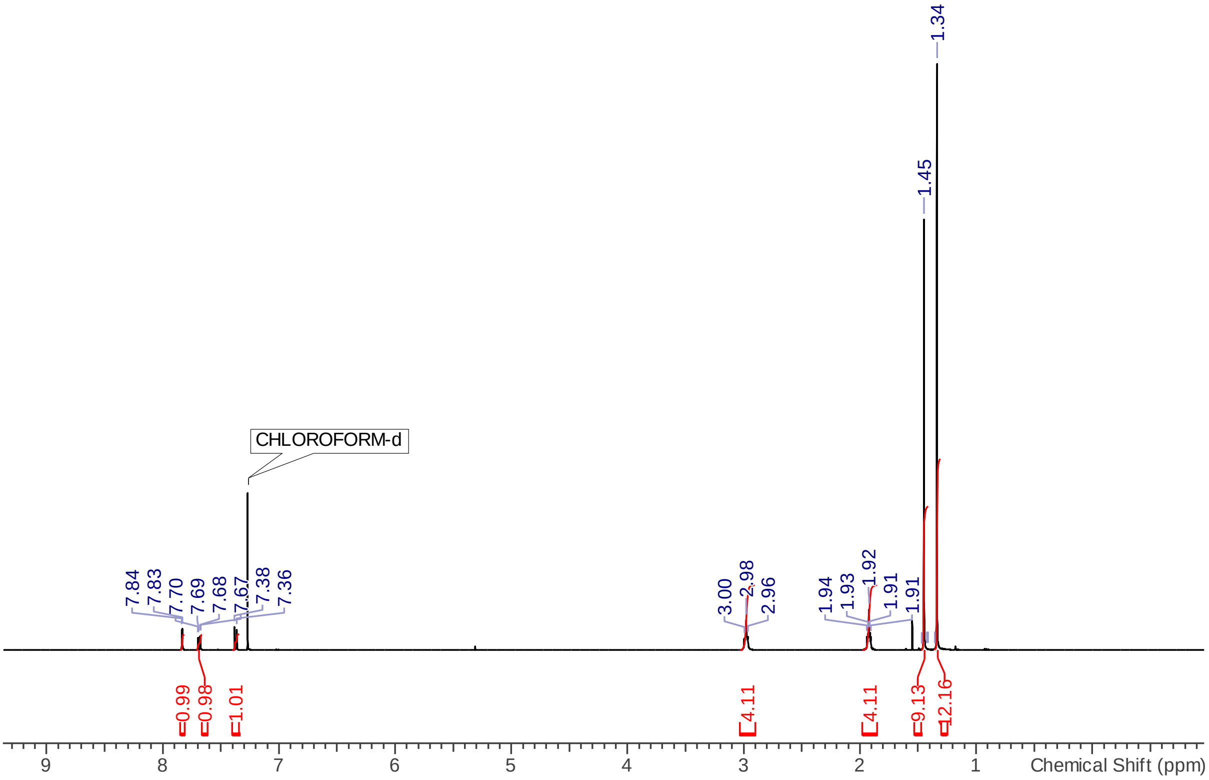

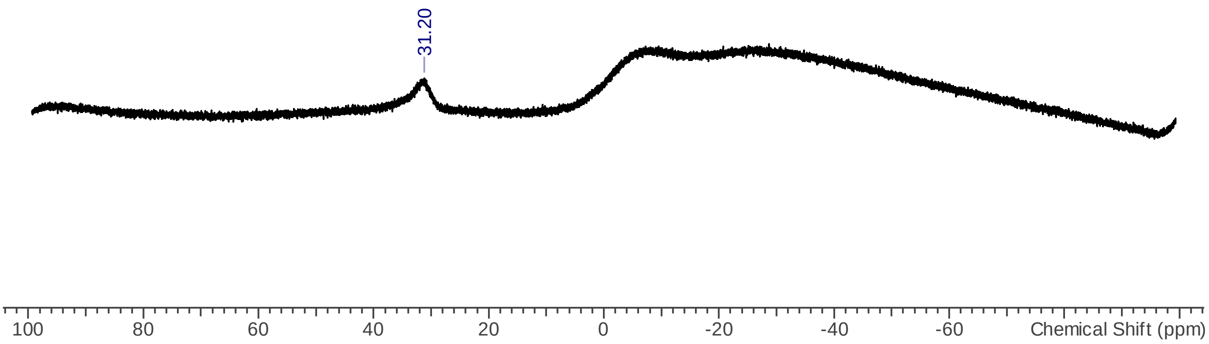
31.20
100
80
60
40
20
0
-20
-40
-60
Chemical Shift (ppm)

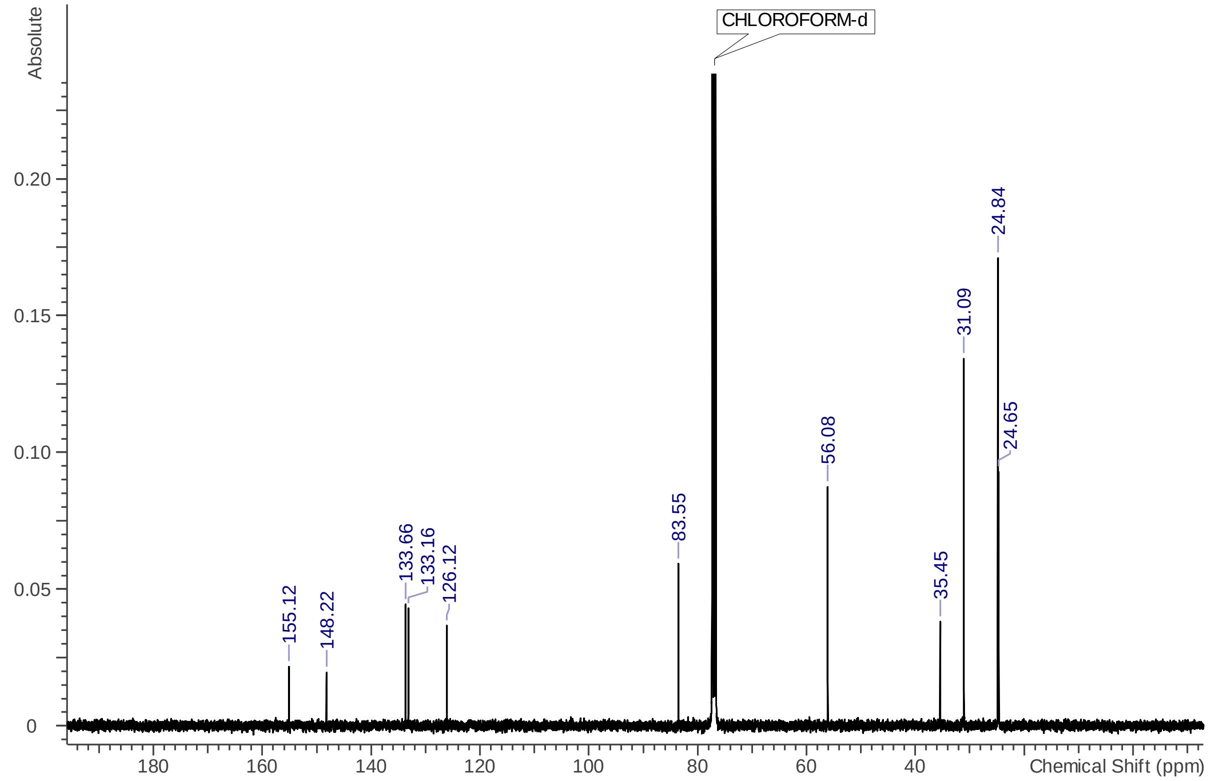
CHLOROFORM-d
Absolute
0.20
0.15
0.10
0.05
0
155.12
148.22
133.66
133.16
126.12
83.55
56.08
35.45
31.09
24.84
24.65
180
160
140
120
100
80
60
40
Chemical Shift (ppm)

1-(2,4-Dichlorophenyl)-2-methylpyrrolidine 1r

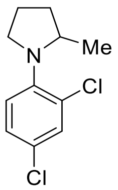

The title compound was prepared according to general procedure 2 using 3-chloro-4-methylaniline (1.00 g, 6.21 mmol), NaH (447 mg, 18.6 mmol), 1,4-dibromopentane (0.82 mL, 6.83 mmol), and DMF (8 mL). Purification by flash column chromatography on silica gel (eluent = 20% DCM in pet. ether), gave the title compound **1r** as a light yellow oil (650 mg, 2.84 mmol, 46%). **R_f** = 0.4 (eluent = 20% DCM in pet. ether); **^{1}H NMR** (400 MHz, $CDCl_3$) δ_H = 7.31 (1H, d, *J* = 2.6 Hz), 7.12 (1H, dd, *J* = 8.7, 2.6 Hz), 6.84 (1H, d, *J* = 8.7 Hz), 3.93 (1H, app. dquin, *J* = 8.2, 6.0 Hz), 3.80 (1H, app. td, *J* = 8.9, 7.1 Hz), 2.95 (1H, app. td, *J* = 8.9, 3.0 Hz), 2.20 (1H, app. dtd, *J* = 11.9, 7.1 Hz), 2.02–1.90 (1H, m), 1.84–1.80 (1H, m), 1.60 (1H, app. ddt, *J* = 11.9, 10.4, 8.2 Hz), 1.03 (3H, d, *J* = 6.0 Hz); **^{13}C NMR** (101 MHz, $CDCl_3$) δ_C = 145.2 (C), 130.4 (CH), 127.0 (CH), 126.4 (C), 125.1 (C), 119.8 (CH), 54.7 (CH), 52.4 (CH_2), 33.9 (CH_2), 24.0 (CH_2), 18.9 (CH_3); **HRMS** (ESI^+) calculated for $[C_{11}H_{14}N^{35}Cl_2]^+$ $(M+H)^+$ m/z: 230.0503; found 230.0503; calculated for $[C_{11}H_{14}N^{35}Cl^{37}Cl]^+$ $(M+H)^+$ m/z: 232.0474; found 232.0475; calculated for $[C_{11}H_{14}N^{37}Cl_2]^+$ $(M+H)^+$ m/z: 234.0444; found 234.0439.

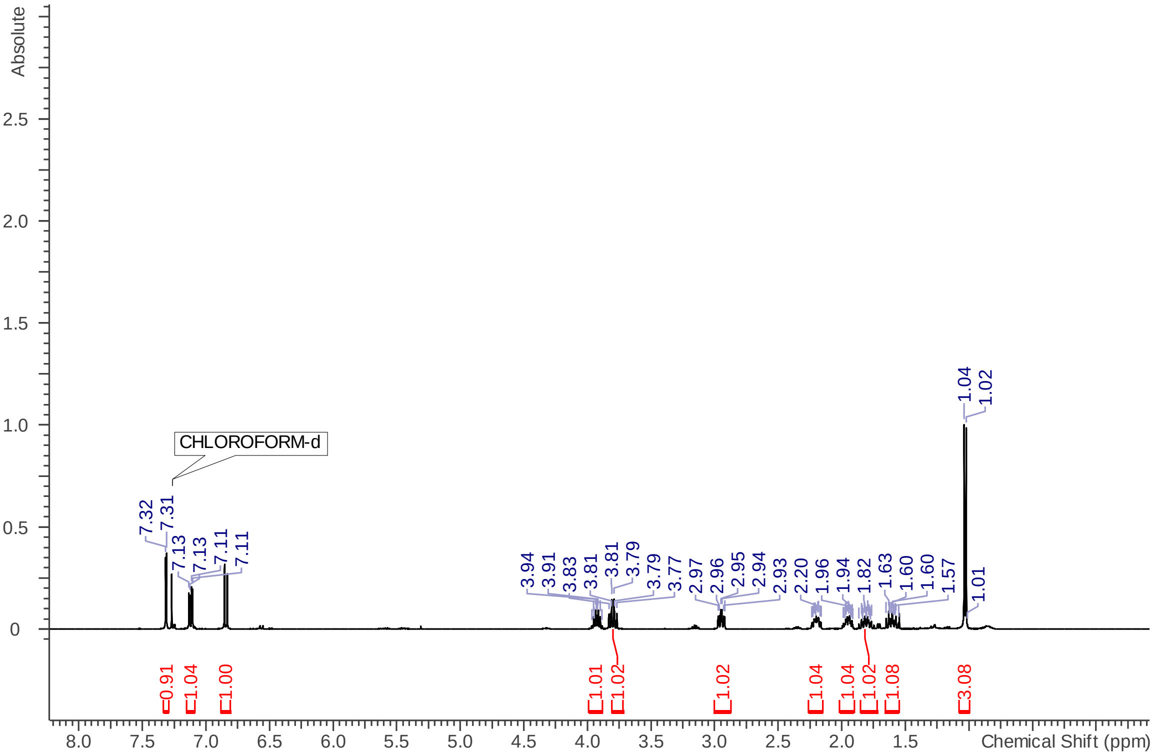

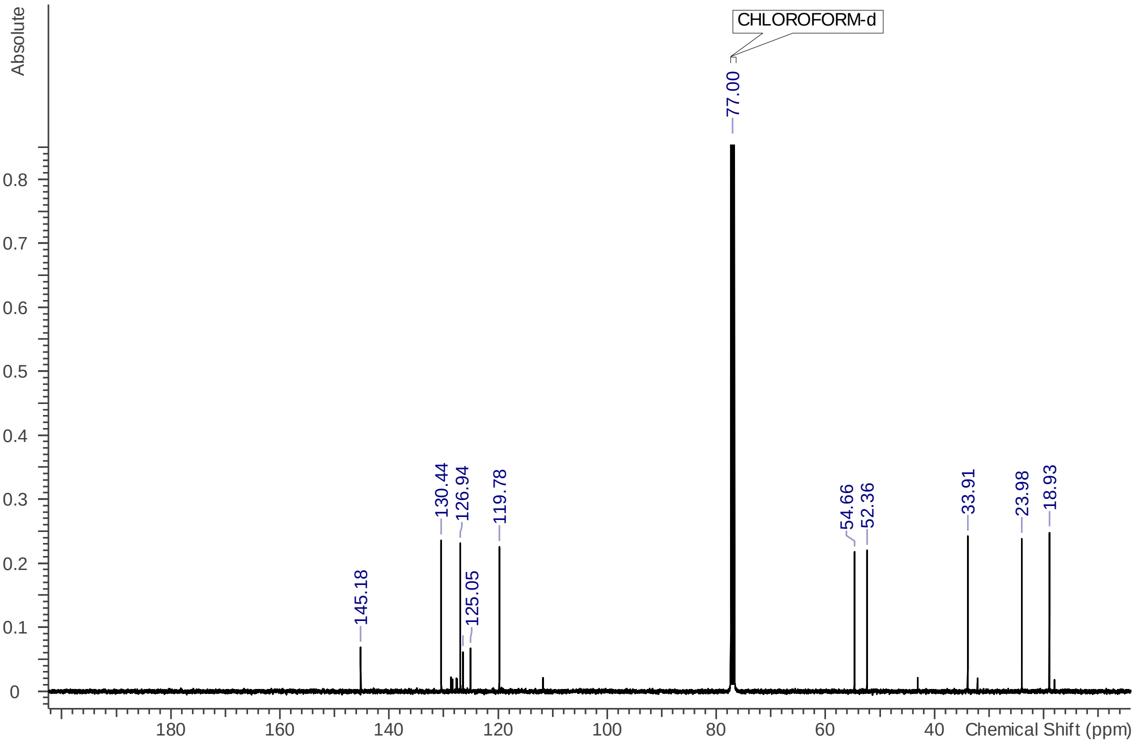

1-(5-Chloro-2-methoxyphenyl)-2-methylpyrrolidine 1s

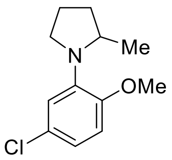

The title compound was prepared according to general procedure 2 using 5-chloro-2-methoxyaniline (1.00 g, 6.37 mmol), NaH (306 mg, 12.7 mmol), 1,4-dibromopentane (0.88 mL, 7.01 mmol), and DMF (7 mL). Purification by flash column chromatography on silica gel (eluent = 60% DCM in pet. ether), gave the title compound **1s** as a colourless oil (402 mg, 1.80 mmol, 28%). **R$_f$** = 0.27 (eluent = 60% DCM in pet. ether); **^{1}H NMR** (400 MHz, $CDCl_3$) δ_H = 6.83–6.68 (3H, m), 3.99 (1H, app. sxt, *J* = 6.3 Hz), 3.82 (3H, s), 3.67–3.56 (1H, m), 3.01 (1H, ddd, *J* = 9.5, 8.3, 3.5 Hz), 2.23–2.13 (1H, m), 1.99–1.89 (1H, m), 1.87–1.75 (1H, m), 1.67-1.54 (1H, m), 1.03 (3H, d, *J* = 6.3 Hz); **^{13}C NMR** (101 MHz, $CDCl_3$) δ_C = 149.6 (C), 139.8 (C), 126.0 (C), 118.8 (CH), 116.8 (CH), 112.3 (CH), 55.7 (CH), 54.4 (CH_3), 51.1 (CH_2), 33.6 (CH_2), 23.5 (CH_2), 19.0 (CH_3); **HRMS** (ESI$^+$) calculated for $[C_{12}H_{17}NO^{35}Cl]^+$ $(M+H)^+$ m/z: 226.0999; found 226.0999; calculated for $[C_{12}H_{17}NO^{37}Cl]^+$ $(M+H)^+$ m/z: 228.0969; found 228.0973.

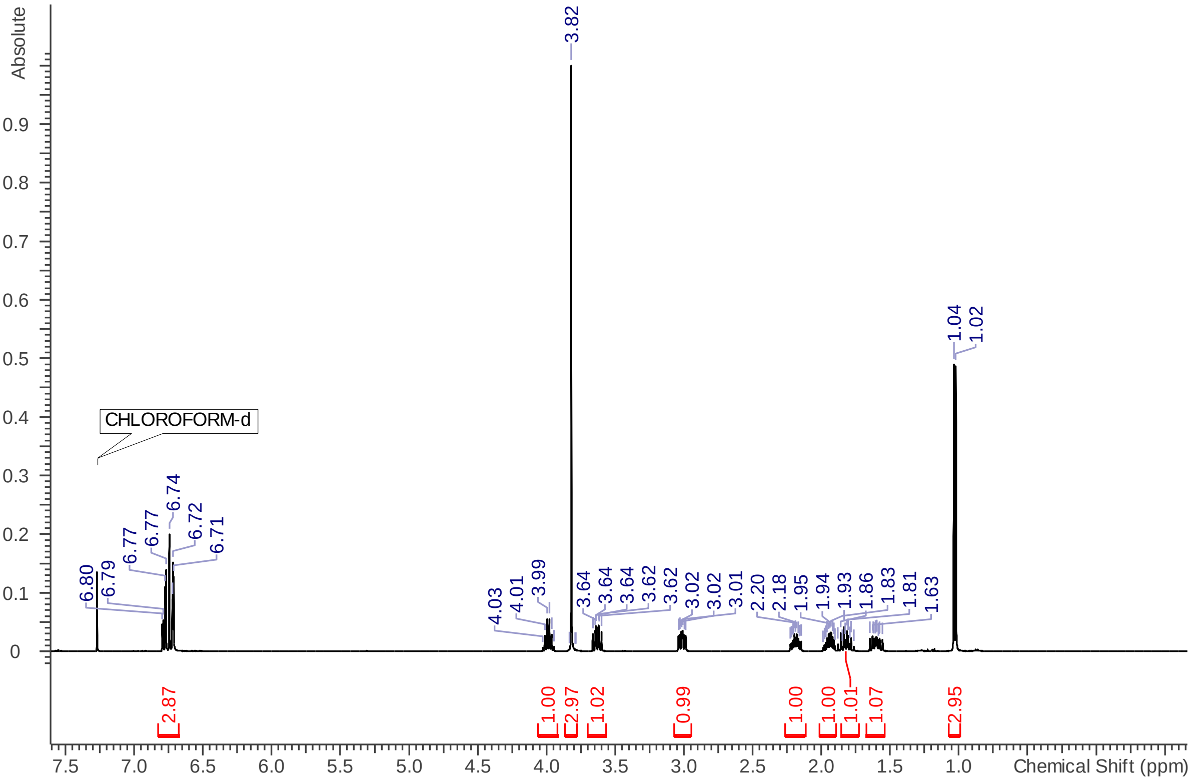

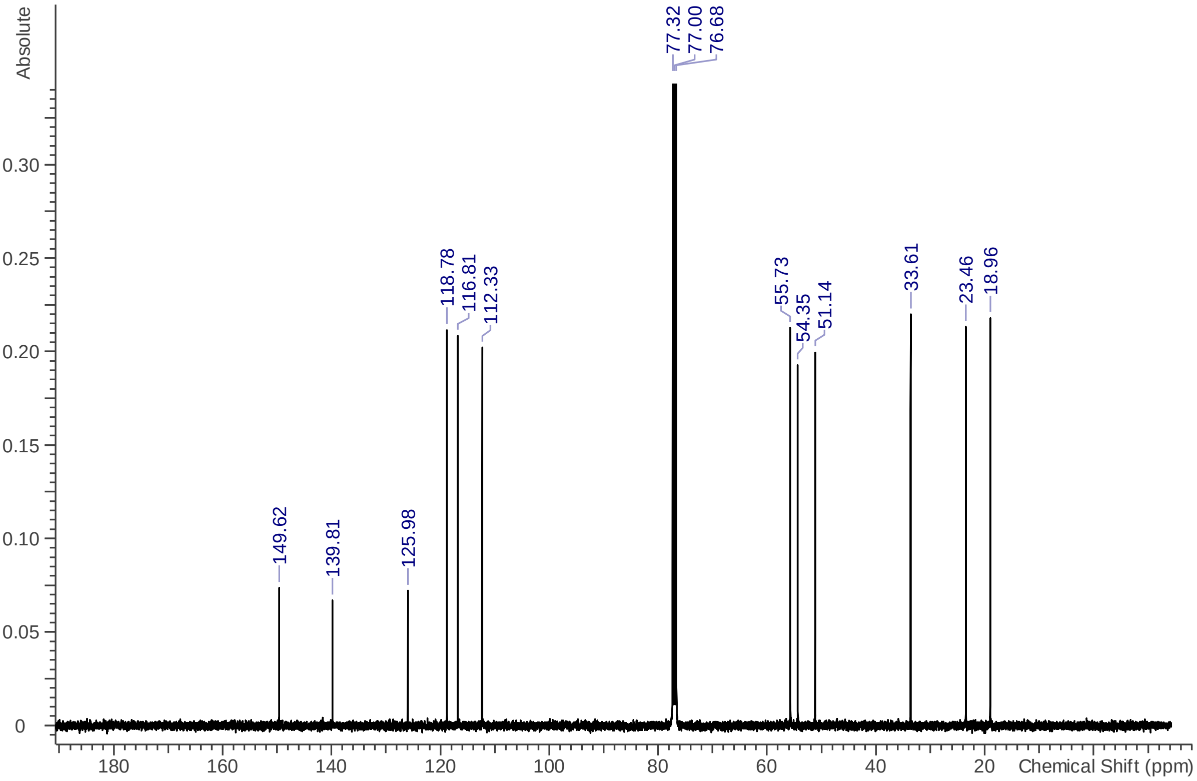

1-(2-Chloro-5-(trifluoromethyl)phenyl)-2-methylpyrrolidine 1t

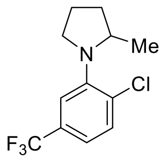

The title compound was prepared according to general procedure 2 using 2-chloro-5-(trifluoromethyl)aniline (0.64 mL, 4.71 mmol), NaH (564 mg, 14.1 mmol), 1,4-dibromopentane (1.6 mL, 11.8 mmol), and DMF (8 mL). Purification by flash column chromatography on silica gel (eluent = 5% DCM in pet. ether), gave the title compound **1t** as a colourless oil (103 mg, 0.39 mmol, 8%); $\mathbf{R_f}$ = 0.5 (eluent = 20% DCM in pet. ether). **^{1}H NMR** (400 MHz, $CDCl_3$) δ_H = 7.39 (1H, d, J = 8.2 Hz), 7.10 (1H, d, J = 2.0 Hz), 7.04 (1H, dd, J = 8.2, 2.0 Hz), 4.05 (1H, app. dquin, J = 8.3, 6.1 Hz), 3.87 (1H, app. td, J = 9.4, 6.8 Hz), 3.09–3.04 (1H, m), 2.28–2.19 (1H, m), 2.02–1.91 (1H, m), 1.88–1.75 (1H, m) 1.70–1.56 (1H, m), 1.07 (3H, d, J = 6.1 Hz); **^{13}C NMR** (101 MHz, $CDCl_3$) δ_C = 146.7 (C), 131.4 (CH), 129.4 (C, q, $^2J_{C\text{-}F}$ = 32.1), 128.6 (C) 124.1 (C, q, $^1J_{C\text{-}F}$ = 272.4 Hz), 116.9 (CH, q, $^3J_{C\text{-}F}$ = 3.9 Hz), 115.3 (CH, q, $^3J_{C\text{-}F}$ = 3.8 Hz), 54.7 (CH), 52.4 (CH_2), 34.1 (CH_2), 24.2 (CH_2), 19.0 (CH_3); **^{19}F NMR** (376 MHz, $CDCl_3$): δ_F = −62.55 ppm (3F, s). **HRMS** (ESI$^+$) calculated for $[C_{12}H_{14}N^{35}ClF_3N]^+$ $(M+H)^+$ m/z: 264.0767; found 264.0766; calculated for $[C_{12}H_{14}{}^{37}ClF_3N]^+$ $(M+H)^+$ m/z: 266.0741; found 266.0737.

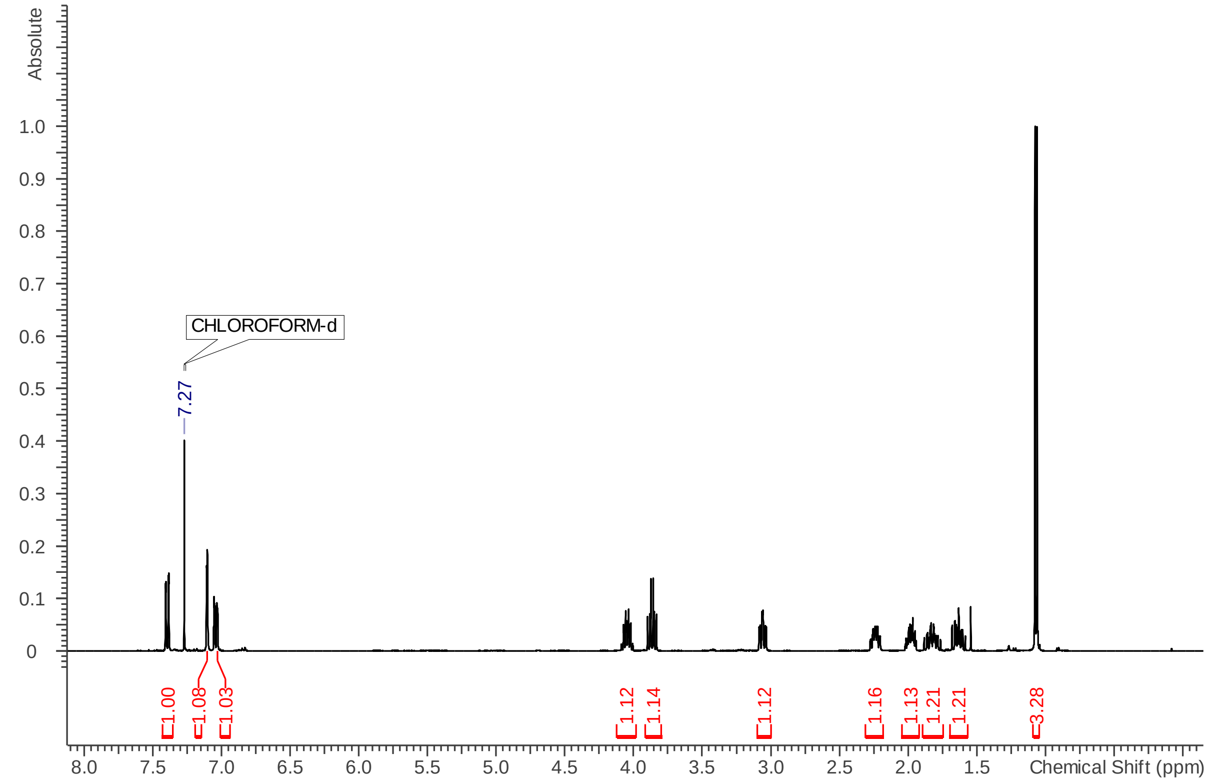

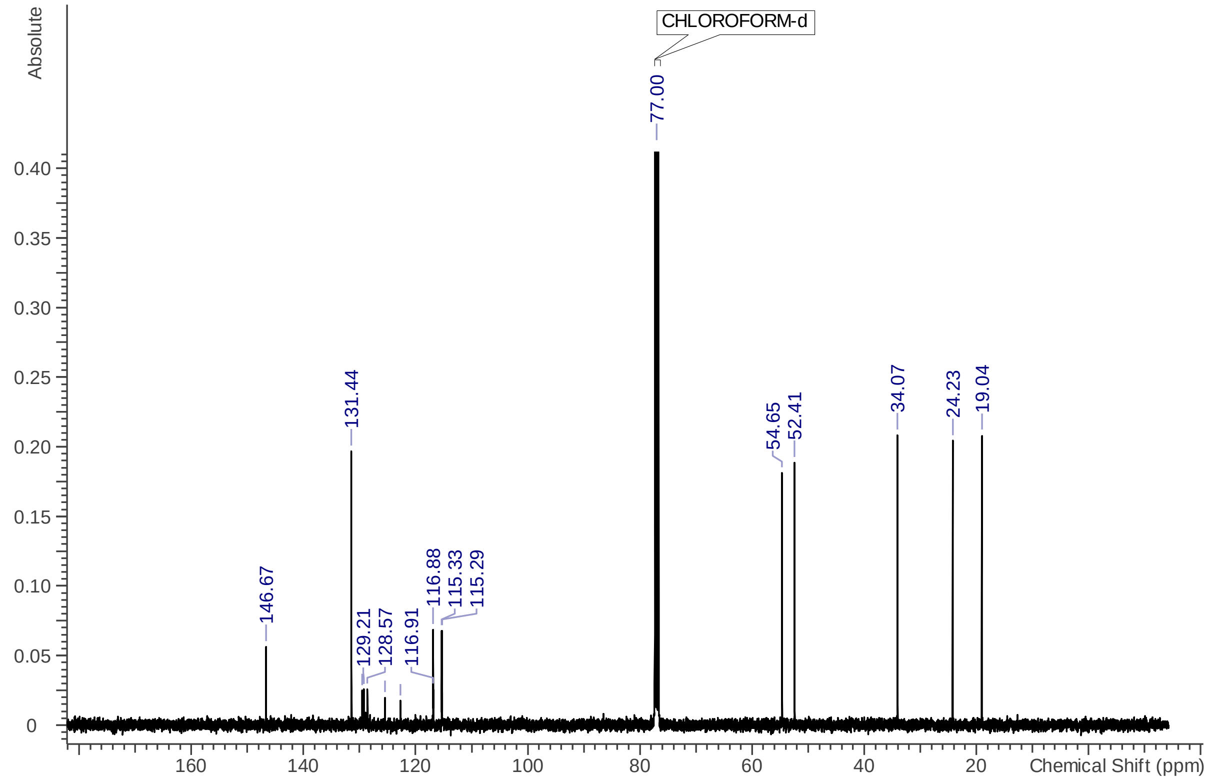

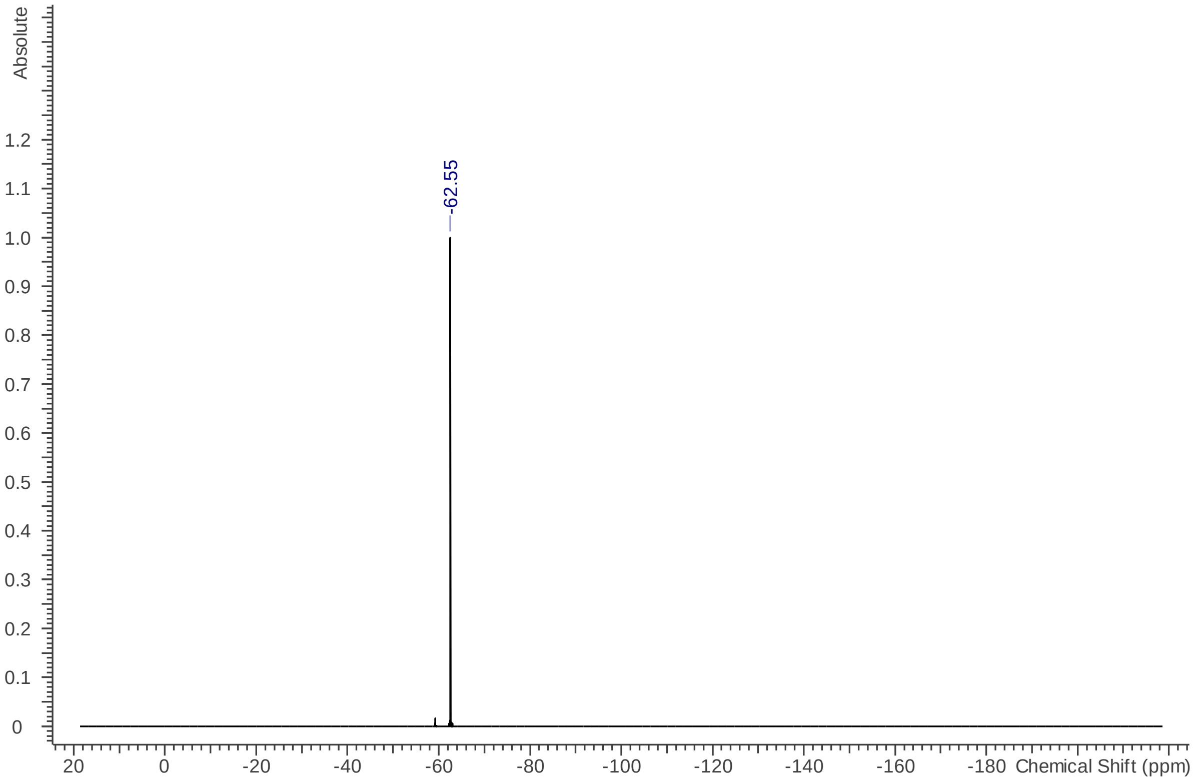
Absolute
1.2
1.1
1.0
0.9
0.8
0.7
0.6
0.5
0.4
0.3
0.2
0.1
0
-62.55
20
0
-20
-40
-60
-80
-100
-120
-140
-160
-180
Chemical Shift (ppm)

1-(3-Chloro-4-methylphenyl)-2,5-dimethylpyrrolidine 1u

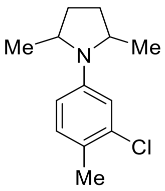

The title compound was prepared according to general procedure 3 using 3-chloro-4-methylaniline (1.00 g, 7.09 mmol), AcOH (0.45 mL, 7.80 mmol), KOH (99.5 mg, 1.77 mmol), 2,5-hexadione (0.83 mL, 7.09 mmol) and $NaBH_3CN$ (446 mg, 7.09 mmol). This yielded a crude mixture of pyrrole and pyrrolidine (600 mg) which was redissolved in AcOH (30 mL), cooled to 10°C (ice/water), and $NaBH_3CN$ (861 mg, 13.7 mmol) added, before stirring at room temperature and stirring for 17 h. Upon completion, the reaction mixture was concentrated in vacuo, basified with NaOH (aq., 2M), and extracted with Et_2O (3 × 30 mL), before the combined organics were concentrated in vacuo. Purification by flash column chromatography on silica gel (eluent = 10% MeOH in 4:1 pet. ether:DCM), gave the title compound **1u** as an orange oil (556 mg, 2.49 mmol, 91%, 68:32 mixture of diastereomers seen). **R_f** = 0.26 (eluent = 10% MeOH in 4:1 pet. ether:DCM); **^{1}H NMR** (400 MHz, $CDCl_3$) **Major diastereomer:** δ_H = 7.03–7.01 (1H, m), 6.62 (1H, d, J = 2.6 Hz), 6.41 (1H, dd, J = 8.3, 2.6 Hz), 3.95 (2H, app. quin, J = 6.4 Hz), 2.26 (3H, s), 2.24–2.20 (2H, m), 1.65–1.61 (2H, m), 1.09 (6H, d, J = 6.2 Hz); **Minor diastereomer: ^{1}H NMR** (400 MHz, $CDCl_3$) δ_H = 7.05-7.02 (1H, m), 6.58 (1H, d, J = 2.6 Hz), 6.44 (1H, dd, J = 8.3, 2.6 Hz), 3.72 (2H, app. dq, J = 11.1, 5.8 Hz), 2.26 (3H, s), 2.10–1.98 (2H, m), 1.76–1.57 (2H, m), 1.27 (6H, d, J = 6.2 Hz); **^{13}C NMR** (101 MHz, $CDCl_3$) δ_C = 148.6 (C)*, 144.5 (C), 134.82 (C), 134.79 (C), 131.1 (CH), 131.0 (CH)*, 122.0 (C)*, 121.2 (C), 113.5 (CH), 112.2 (CH)*, 112.0 (CH), 110.6 (CH)*, 56.1 (2×CH)*, 52.9 (2×CH), 32.3 (2×CH_2)*, 30.3 (2×CH_2), 21.7 (2×CH_3)*, 18.7 (CH_3)†, 18.0 (2×CH_3); **HRMS** (ESI$^+$): calculated for $[C_{13}H_{19}N^{35}Cl]^+$ $(M+H)^+$ m/z: 349.2644; found 224.1196; calculated for $[C_{13}H_{19}N^{37}Cl]^+$ $(M+H)^+$ m/z: 226.1177; found 226.1170. **Signals attributed to minor diastereomer. †Signals attributed to both major and minor diastereomers.*

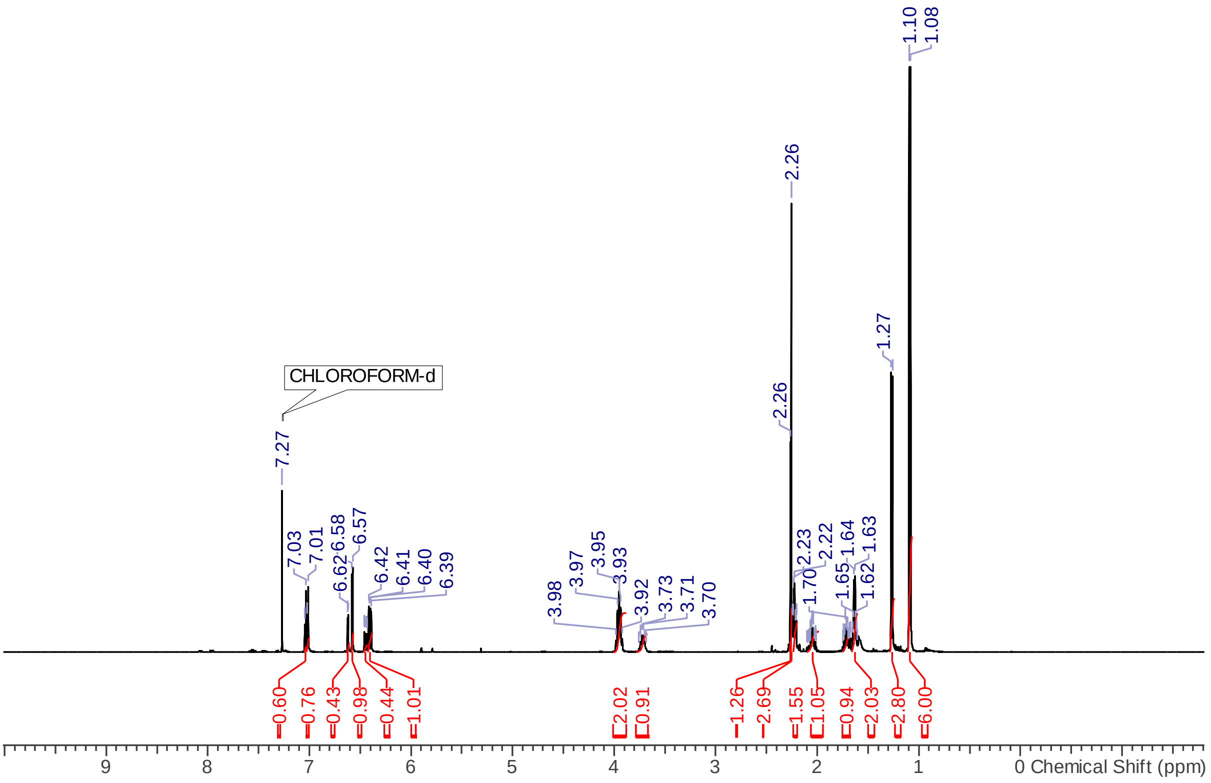

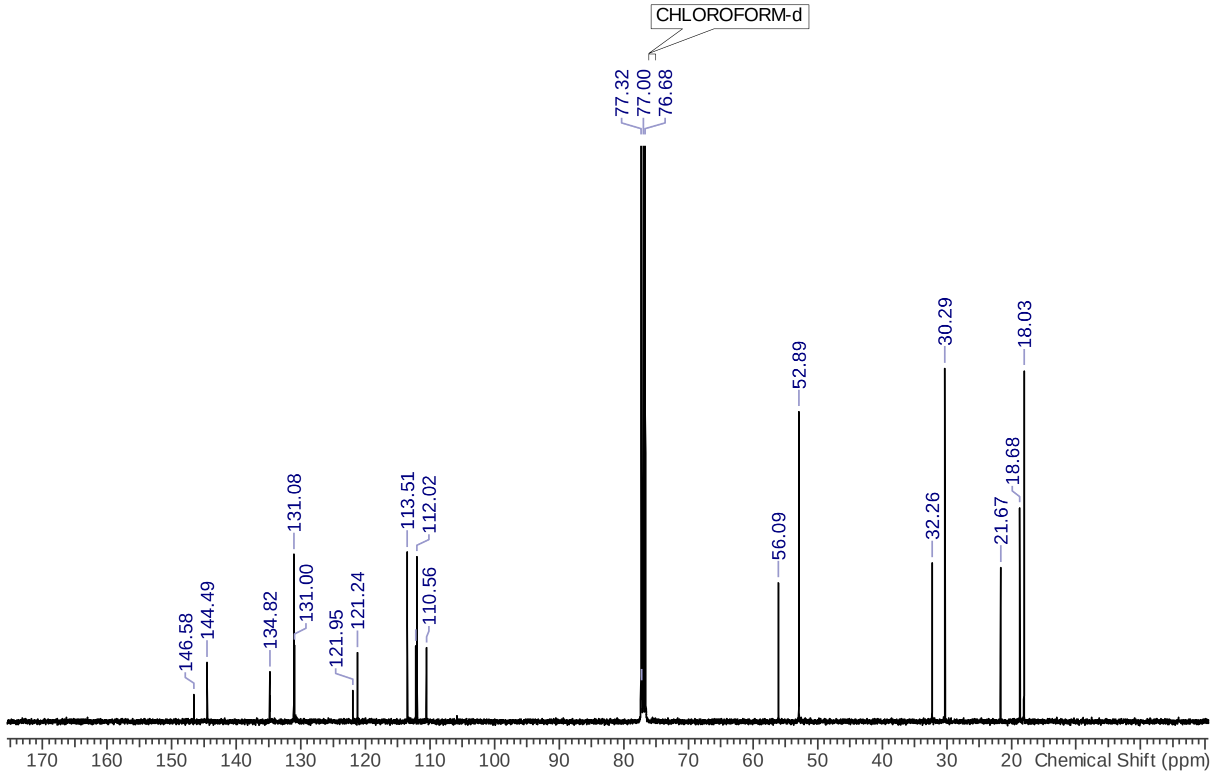
CHLOROFORM-d
77.32
77.00
76.68
146.58
144.49
134.82
131.08
131.00
121.95
121.24
113.51
112.02
110.56
56.09
52.89
32.26
30.29
21.67
18.68
18.03
170
160
150
140
130
120
110
100
90
80
70
60
50
40
30
20
Chemical Shift (ppm)

1-(3-Chloro-4-methylphenyl)-2-methylpyrrolidine 1v

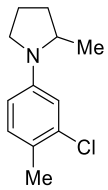

The title compound was prepared according to general procedure 2 using 3-chloro-4-methylaniline (1.00 g, 7.10 mmol), NaH (341 mg, 14.2 mmol), 1,4-dibromopentane (0.98 mL, 7.81 mmol), and DMF (7 mL). Purification by flash column chromatography on silica gel (eluent = 30% DCM in pet. ether), gave the title compound **1v** as a light yellow oil (803 mg, 3.84 mmol, 54%). **R$_f$** = 0.6 (eluent = 30% DCM in pet. ether); **^{1}H NMR** (400 MHz, $CDCl_3$) δ_H = 7.04 (1H, d, *J* = 8.3 Hz), 6.58 (1H, d, *J* = 2.6 Hz), 6.41 (1H, dd, *J* = 8.3, 2.6 Hz), 3.82 (1H, app. quind, *J* = 6.3, 1.7 Hz), 3.43–3.35 (1H, m), 3.16–3.10 (1H, m), 2.27 (3H, s), 2.13–1.93 (3H, m), 1.74–1.67 (1H, m), 1.16 (3H, d, J = 6.3 Hz); **^{13}C NMR** (101 MHz, $CDCl_3$) δ_C = 146.4 (C), 134.8 (C), 131.1 (CH), 121.7 (C), 112.1 (CH), 110.4 (CH), 53.7 (CH), 48.3 (CH_2), 33.1 (CH_2), 23.2 (CH_2), 19.2 (CH_3), 18.7 (CH_3); **HRMS** (ESI$^+$) calculated for $[C_{12}H_{17}N^{35}Cl]^+$ $(M+H)^+$ m/z: 210.1049; found 210.1050; calculated for $[C_{12}H_{17}N^{37}Cl]^+$ $(M+H)^+$ m/z: 212.1020; found 212.1021.

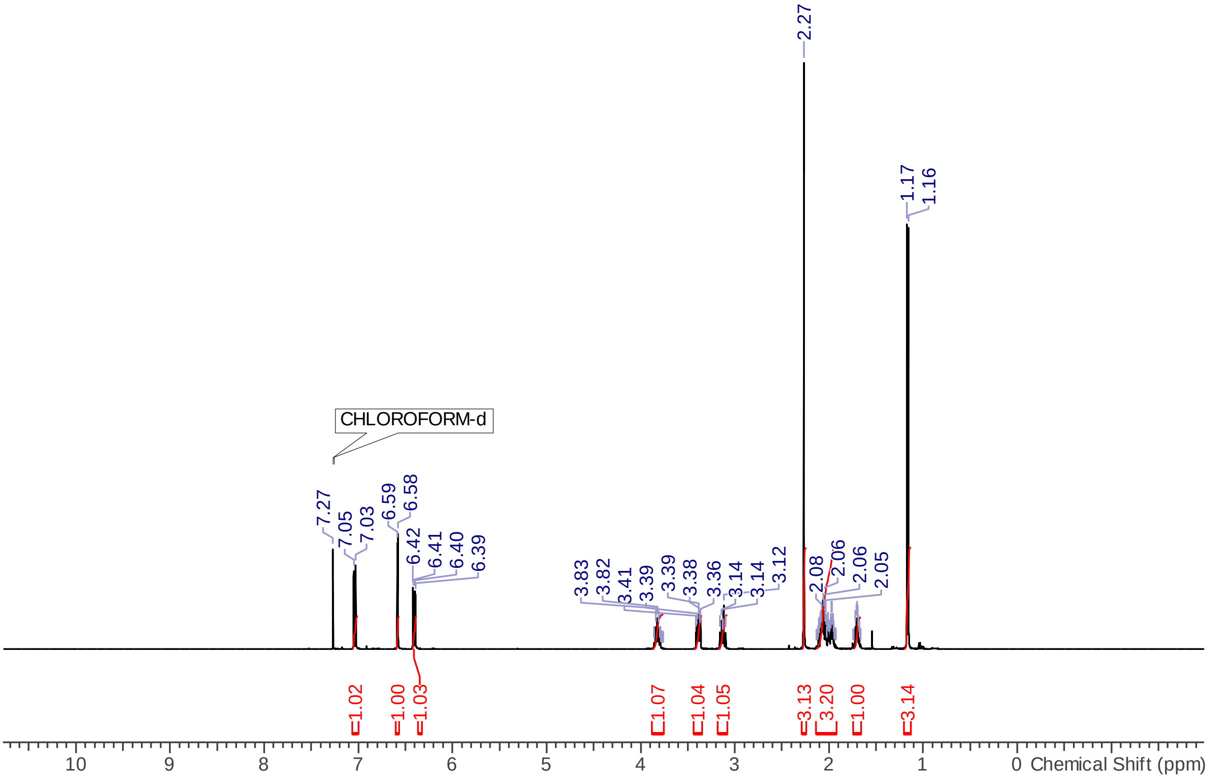

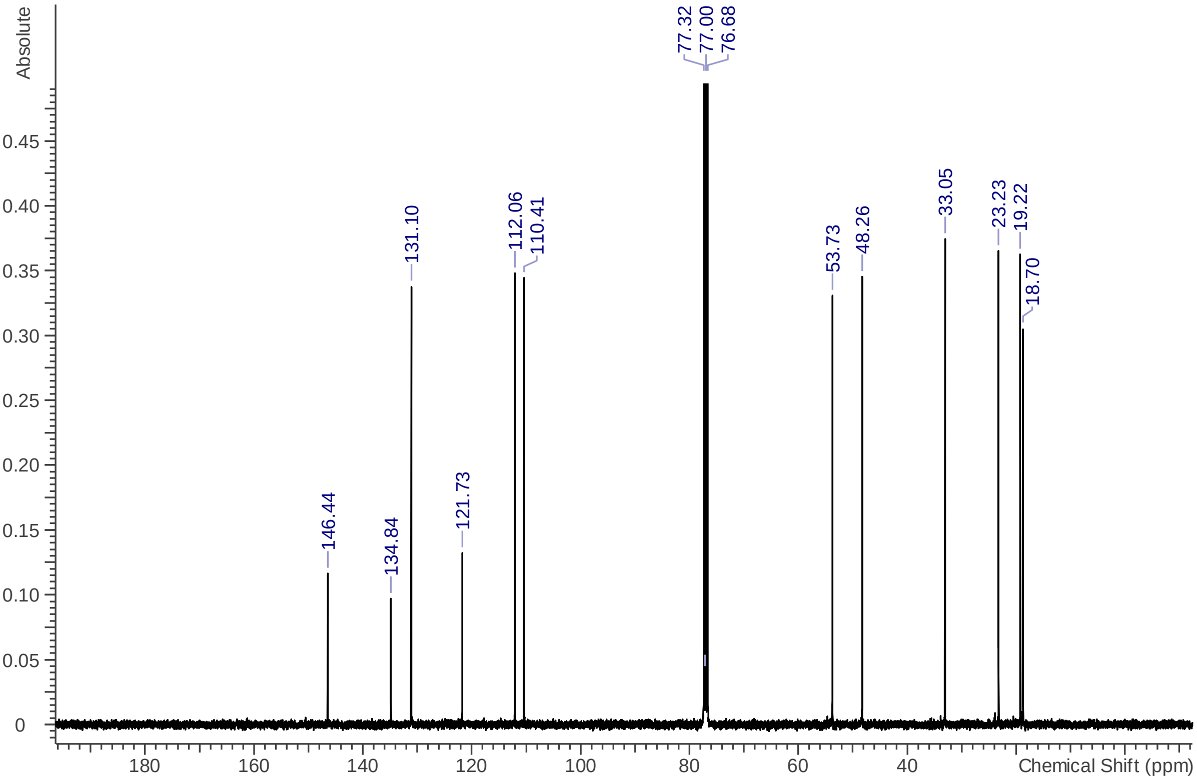

1-(4-Methoxy-2,6-dimethylphenyl)pyrrolidine 1w

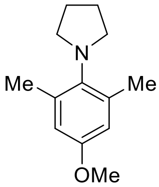

The title compound was prepared according to general procedure 1 using 4-methoxy-2,6-dimethylaniline (350 mg, 2.55 mmol), K_2CO_3 (1.10 g, 7.65 mmol), and 1,4-dibromobutane (0.46 mL, 3.8 mmol). Purification by flash column chromatography on silica gel (eluent = 10% EtOAc in pet. ether), gave the title compound **1w** as a brown oil (244 mg, 1.80 mmol, 47%). $\mathbf{R_f}$ = 0.6 (eluent = 10% EtOAc in pet. ether); **^{1}H NMR** (400 MHz, $CDCl_3$) δ_H = 6.60 (2H, s), 3.76 (3H, s), 3.15 (4H, dt, *J* = 6.4, 4.1 Hz), 2.23 (6H, s), 1.97 (4H, dt, *J* = 6.4, 3.1 Hz). ^{13}C NMR (101 MHz, $CDCl_3$) δ_C = 156.5 (C), 139.7 (C), 138.5 (2×C), 113.4 (2×CH), 55.2 (CH_3), 50.3 (2×CH_2), 26.6 (2×CH_2), 18.8 (2×CH_3); Spectroscopic data in accordance with that stated in the literature.[1]

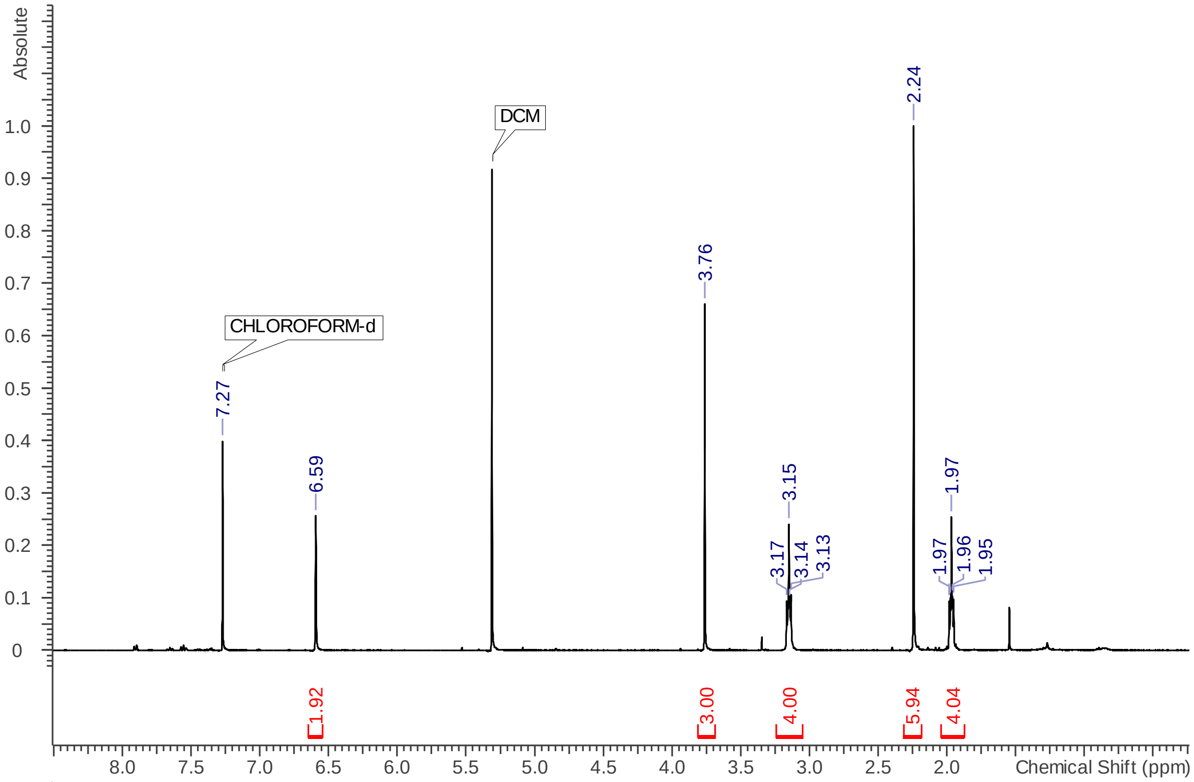

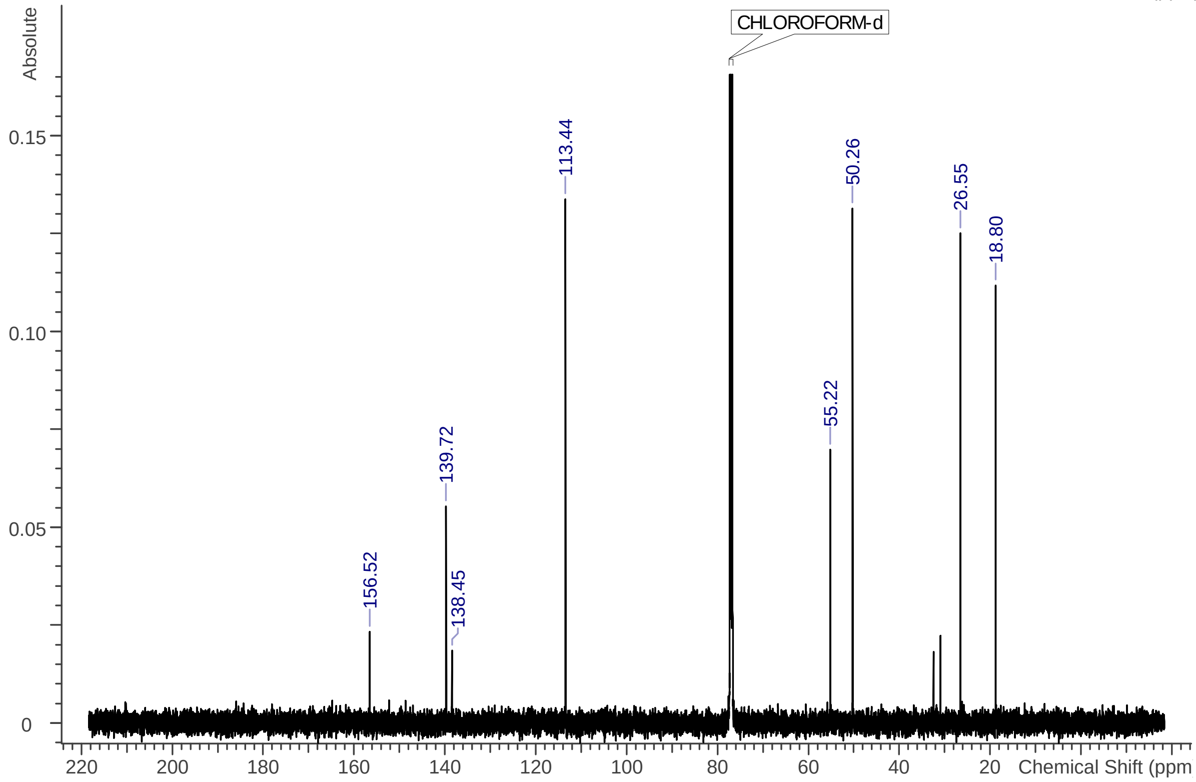

1-Mesityl-3-phenylpyrrolidine-2,5-dione S4

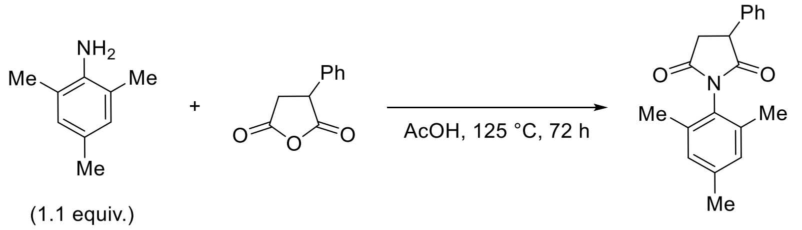

A 250 mL round bottomed flask equipped with stirrer bar and reflux condenser was charged with 2-phenylsuccinic anhydride (1.04 g, 5.91 mmol) and AcOH (20 mL), before mesitylamine (0.88 mL, 6.3 mmol) was added. The reaction was heated to 125 °C for 72 h, before the product was separated between EtOAc (30 mL) and water (20 mL). The aqueous layer was extracted with EtOAc (2 × 30 mL), before the combined organics were washed with water (2 × 20 mL), dried with MgSO4, filtered, and concentrated in vacuo. Purification by recrystallisation from the minimal amount of hot EtOH gave the desired compound **S4** as an off-white crystalline solid (1.21 g, 4.11 mmol, 70%). **^{1}H NMR** (400 MHz, $CDCl_3$) δ_H = 7.45–7.40 (2H, m), 7.39–7.33 (3H, m), 7.00–6.98 (1H, m), 6.98–6.96 (1H, m), 4.26 (1H, dd, *J* = 9.7, 4.9 Hz), 3.44 (1H, dd, *J* = 18.7, 9.7 Hz), 3.07 (1H, dd, *J* = 18.7, 4.9 Hz), 2.31 (3H, s), 2.13 (3H, s), 2.10 (3H, s); **^{13}C NMR** (101 MHz, $CDCl_3$) δ_C = 176.4 (C=O), 175.2 (C=O), 139.5 (C), 137.2 (C), 135.3 (C), 135.2 (C), 129.5 (CH), 129.4 (CH), 129.3 (2×CH), 128.1 (CH), 127.5 (C, 2×CH), 46.4 (CH), 37.4 (CH_2), 21.1 (CH_3), 17.9 (CH_3), 17.8 (CH_3); Spectroscopic data in accordance with that stated in the literature.[6]

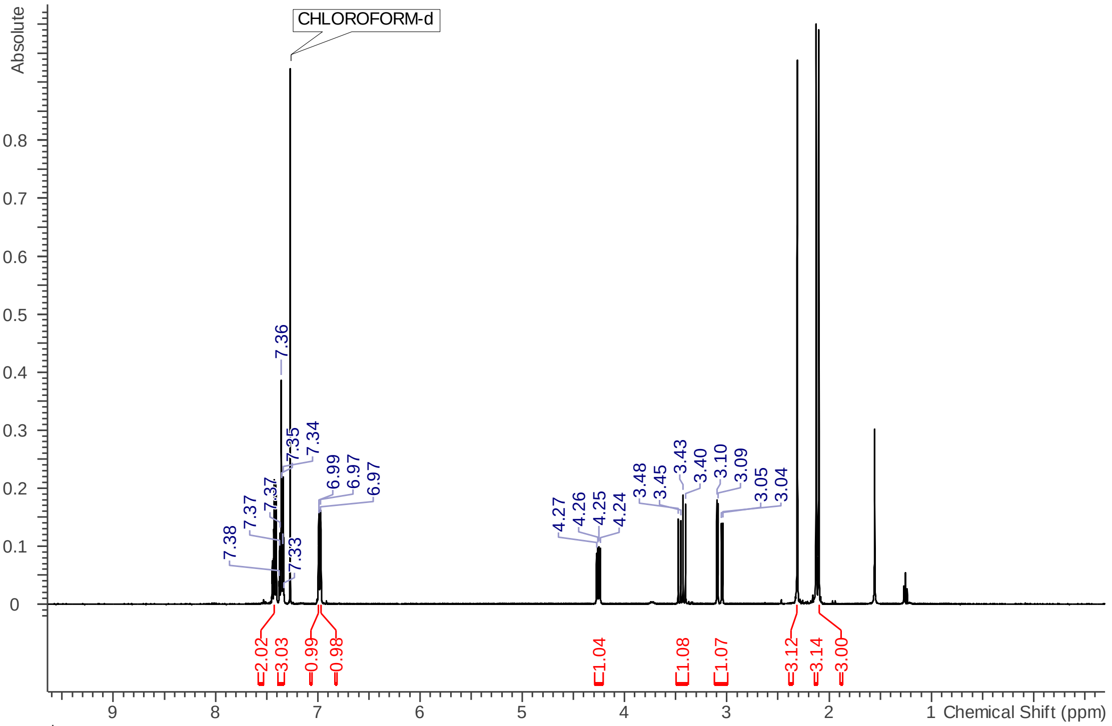

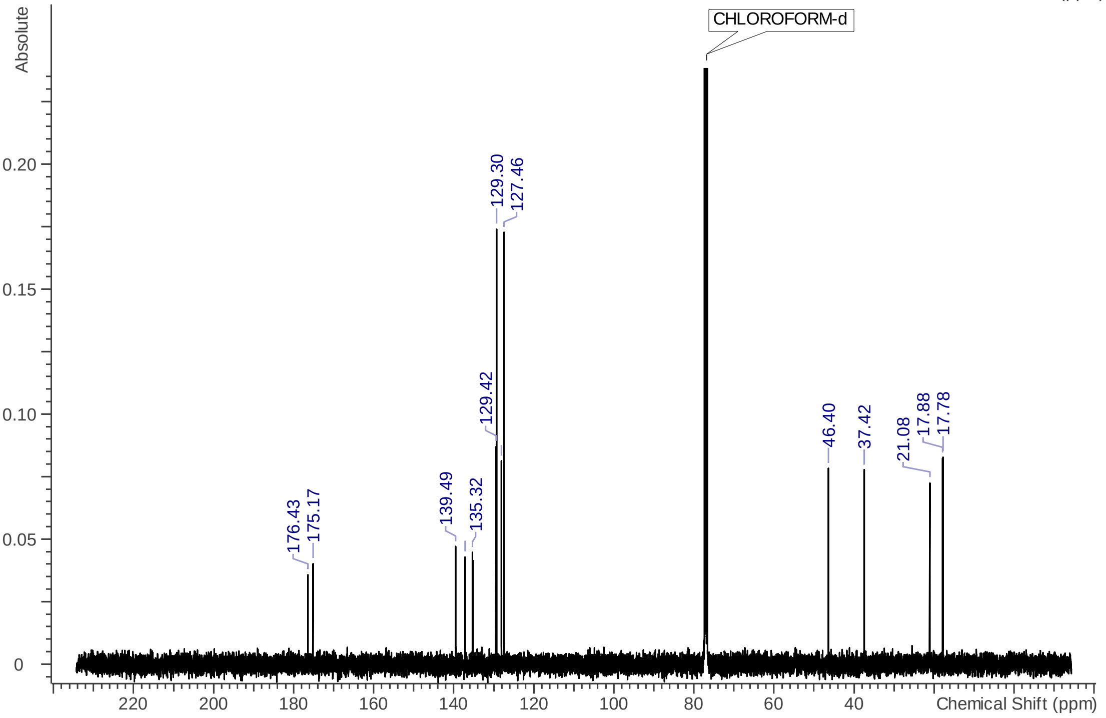

1-Mesityl-3-phenylpyrrolidine 1x

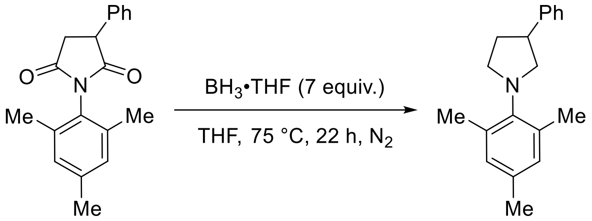

A 50 mL 2-necked round bottomed flask equipped with stirrer bar, reflux condenser and septa was charged with 1-mesityl-3-phenylpyrrolidine-2,5-dione **S4** (250 mg, 0.85 mmol) and THF (4 mL) under a nitrogen atmosphere. BH_3.THF (6.0 mL, 6.0 mmol) was then added slowly, before the reaction was heated to 75 °C for 22 h. The reaction was then quenched with MeOH (5 mL), before solvent was removed in vacuo. The crude materials were redissolved in EtOAc (15 mL), washed with brine (10 mL), dried over $MgSO_4$, filtered, and concentrated in vacuo. Purification by flash column chromatography on silica gel (eluent = 10% DCM in pet. ether) gave the title compound **1x** as a colourless oil (62 mg, 0.23 mmol, 27%). **R$_f$** 0.29 (eluent = 10% DCM in pet. ether); **^{1}H NMR** (400 MHz, $CDCl_3$) δ_H = 7.39–7.31 (4H, m), 7.27–7.21 (1H, m), 6.88 (2H, s), 3.63–3.51 (2H, m), 3.47–3.29 (3H, m), 2.47–2.36 (1H, m), 2.31 (6H, s), 2.27 (3H, s), 2.24–2.12 (1H, m); **^{13}C NMR** (101 MHz, $CDCl_3$) δ_C = 143.8 (C), 142.4 (C), 138.2 (2×C), 134.8 (C), 129.3 (2×CH), 128.4 (2×CH), 127.3 (2×CH), 126.2 (CH), 57.6 (CH_2), 50.6 (CH_2), 45.5 (CH), 34.5 (CH_2), 20.8 (CH_3), 18.7 (2×CH_3); **HRMS** (ESI$^+$): calculated for $[C_{19}H_{24}N]^+$ (M+H)$^+$ m/z: 266.1909; found 266.1910.

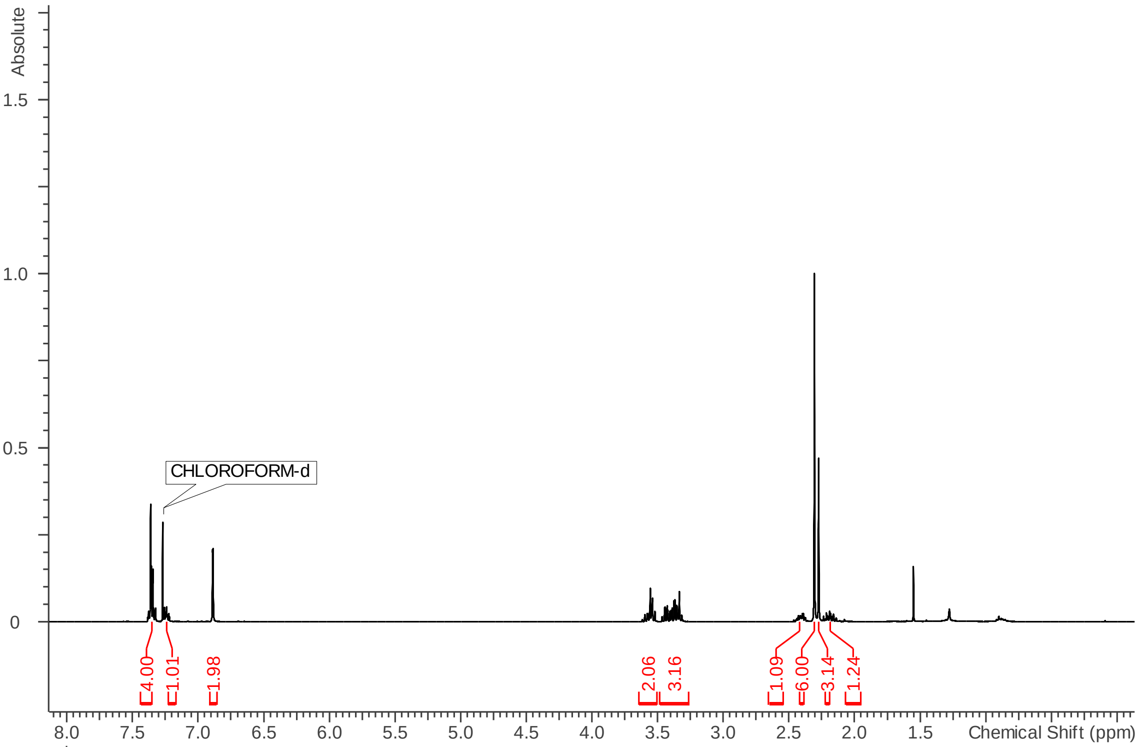

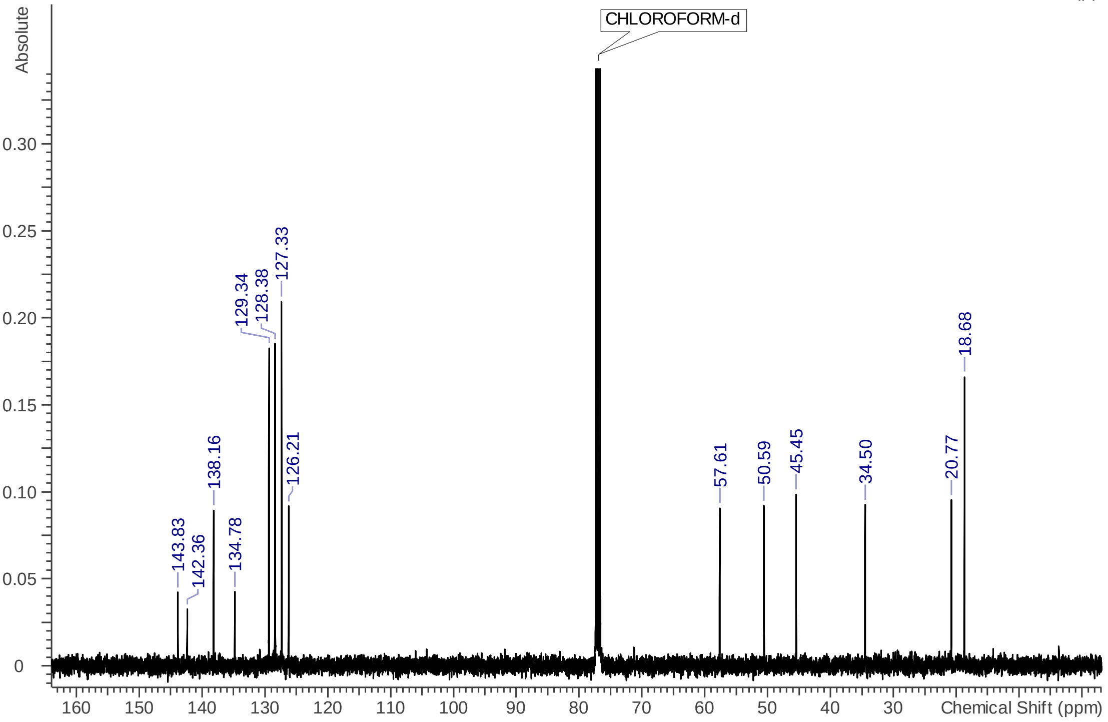

2-Methyl-1-(2,3,5,6-tetrafluorophenyl)pyrrolidine 1y

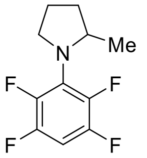

The title compound was prepared according to general procedure 2 using 2,3,5,6-tetrafluoroaniline (1.00 g, 6.05 mmol), NaH (290 mg, 12.1 mmol), 1,4-dibromopentane (0.84 mL, 6.66 mmol), and DMF (7 mL). Purification by flash column chromatography on silica gel (eluent = 5% DCM in pet. ether), gave the title compound **1y** as a colourless oil (233 mg, 1.00 mmol, 17%). **R$_f$** = 0.6 (eluent = 5% DCM in pet. ether); **^{1}H NMR** (400 MHz, $CDCl_3$) δ_H = 6.49 (1H, tt, J = 9.9, 7.0 Hz), 4.18–4.06 (1H, m), 3.82 (1H, app. tdt, J = 9.4, 6.6, 2.8 Hz), 3.29 (1H, ddd, J = 9.4, 8.1, 1.6 Hz), 2.23–2.13 (1H, m), 2.03-1.93 (1H, m), 1.92-1.78 (1H, m), 1.57-1.46 (1H, m), 1.11 (3H, d, J = 6.4 Hz); **^{13}C NMR** (101 MHz, $CDCl_3$) δ_C = 146.8 (2×C, dtd, $^1J_{C\text{-}F}$ = 243.6 Hz, $^2J_{C\text{-}F}$ = 13.5 Hz, $^4J_{C\text{-}F}$ = 3.4 Hz), 140.5 (2×C, dddd, $^1J_{C\text{-}F}$ = 243.4 Hz, $^2J_{C\text{-}F}$ = 14.9 Hz, $^3J_{C\text{-}F}$ = 7.0 Hz, $^4J_{C\text{-}F}$ = 3.6 Hz), 128.2–127.7 (C, m), 94.9 (CH, t, J = 23.6 Hz), 56.0 (CH, t, J = 4.7 Hz), 51.9 (CH_2, t, J = 4.8 Hz), 34.4 (CH_2), 25.0 (CH_2), 20.4 (CH_2); **^{19}F{^{1}H}NMR** (376 MHz, $CDCl_3$): δ_F = −141.69 (2F, dtd, J = 18.7, 10.0, 4.8 Hz), −153.17 ppm (2F, dd, J = 10.2, 9.2 Hz); **HRMS** (ESI$^+$) calculated for $[C_{11}H_{12}NF_4]^+$ $(M+H)^+$ m/z: 234.0906; found 234.0913.

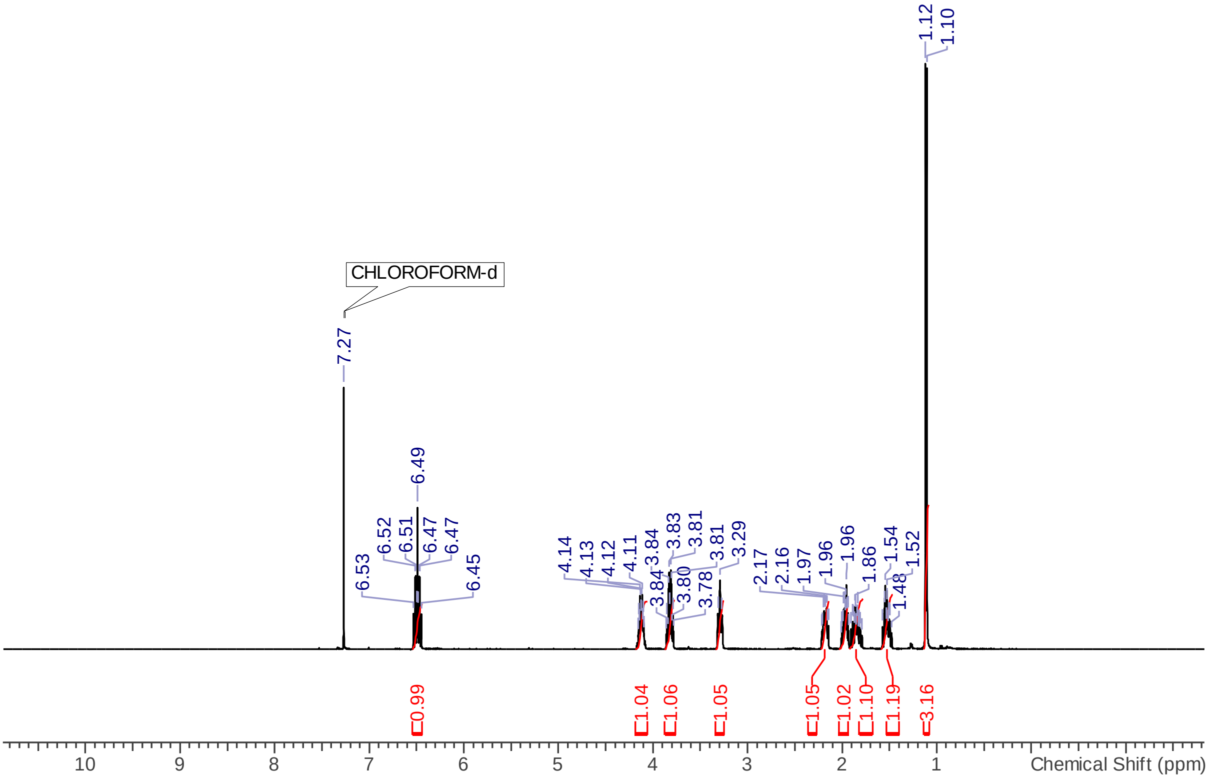

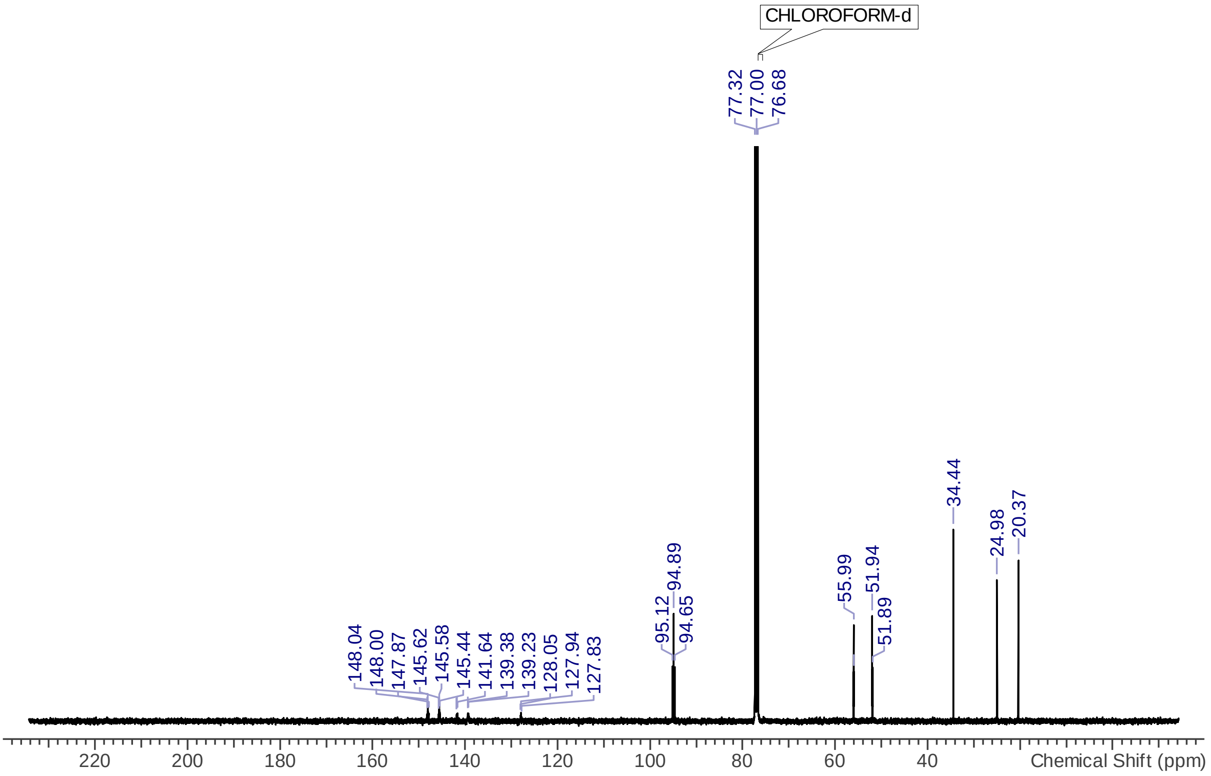

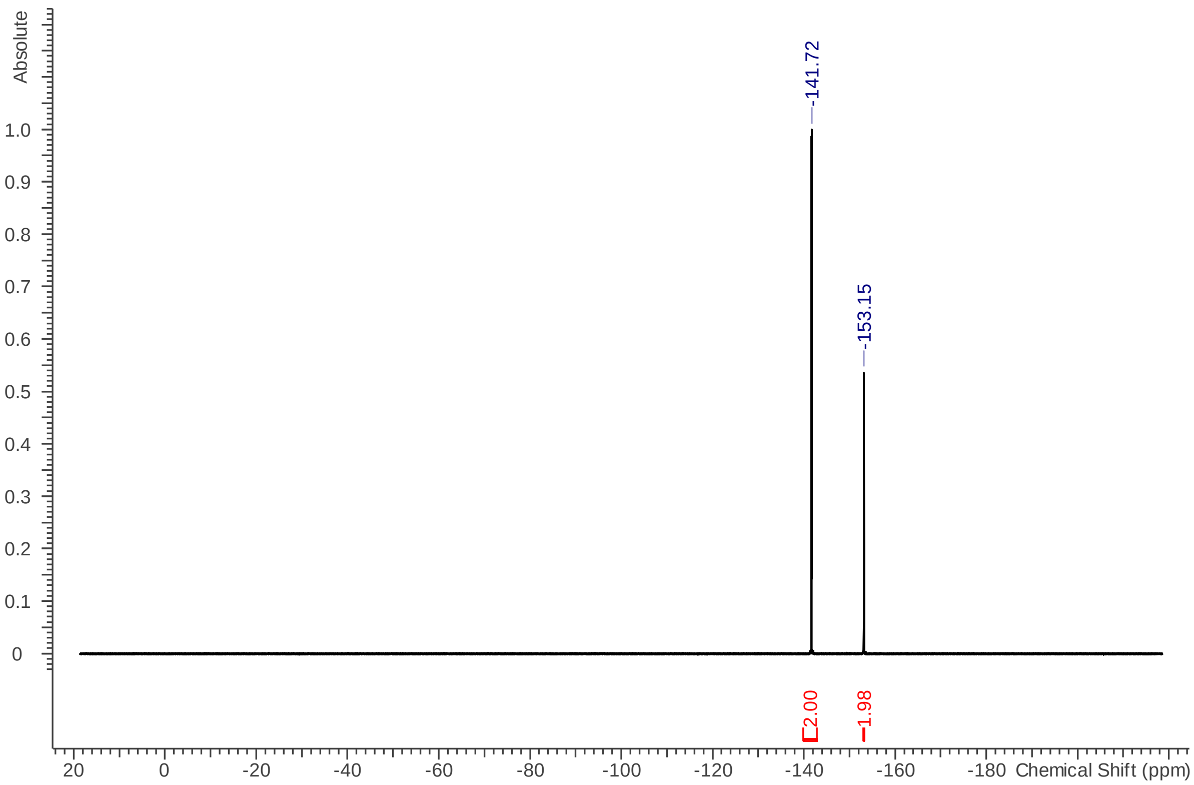

-141.72
-153.15
2.00
1.98
Absolute
1.0
0.9
0.8
0.7
0.6
0.5
0.4
0.3
0.2
0.1
0
20
0
-20
-40
-60
-80
-100
-120
-140
-160
-180
Chemical Shift (ppm)

1,2-Bis(4-(2-methylpyrrolidin-1-yl)phenyl)ethane 1z

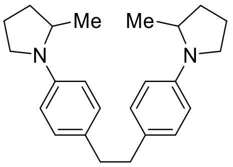

The title compound was prepared according to general procedure 2 using 3,3'-methylenedianiline (1.00 g, 4.71 mmol), NaH (338 mg, 14.1 mmol), 1,4-dibromopentane (1.48 mL, 11.8 mmol), and DMF (7 mL). Purification by flash column chromatography on silica gel (eluent = 10% EtOAc in pet. ether), gave the title compound **1z** as a white solid (920 mg, 2.64 mmol, 56%). $\mathbf{R_f}$ = 0.4 (eluent = 10% EtOAc in pet. ether); **^{1}H NMR** (400 MHz, $CDCl_3$) δ_H = 7.13–7.07 (4H, m), 6.57-6.52 (4H, m), 3.86 (2H, app. quind, *J* = 6.2, 1.8 Hz), 3.47-3.39 (2H, m), 3.21-3.10 (2H, m), 2.79 (4H, s), 2.15–1.92 (6H, m), 1.76-1.64 (2H, m), 1.18 (6H, d, *J* = 6.2 Hz); **^{13}C NMR** (101 MHz, $CDCl_3$) δ_C = 145.9 (2×C), 129.4 (4×CH, 2×C), 112.1 (4×CH), 54.0 (2×CH), 48.8 (2×CH_2), 37.9 (2×CH_2), 33.5 (2×CH_2), 23.7 (2×CH_2), 19.9 (2×CH_3); calculated for $C_{24}H_{33}N_2$ $(M+H)^+$ m/z: 349.2644; found 349.2644.

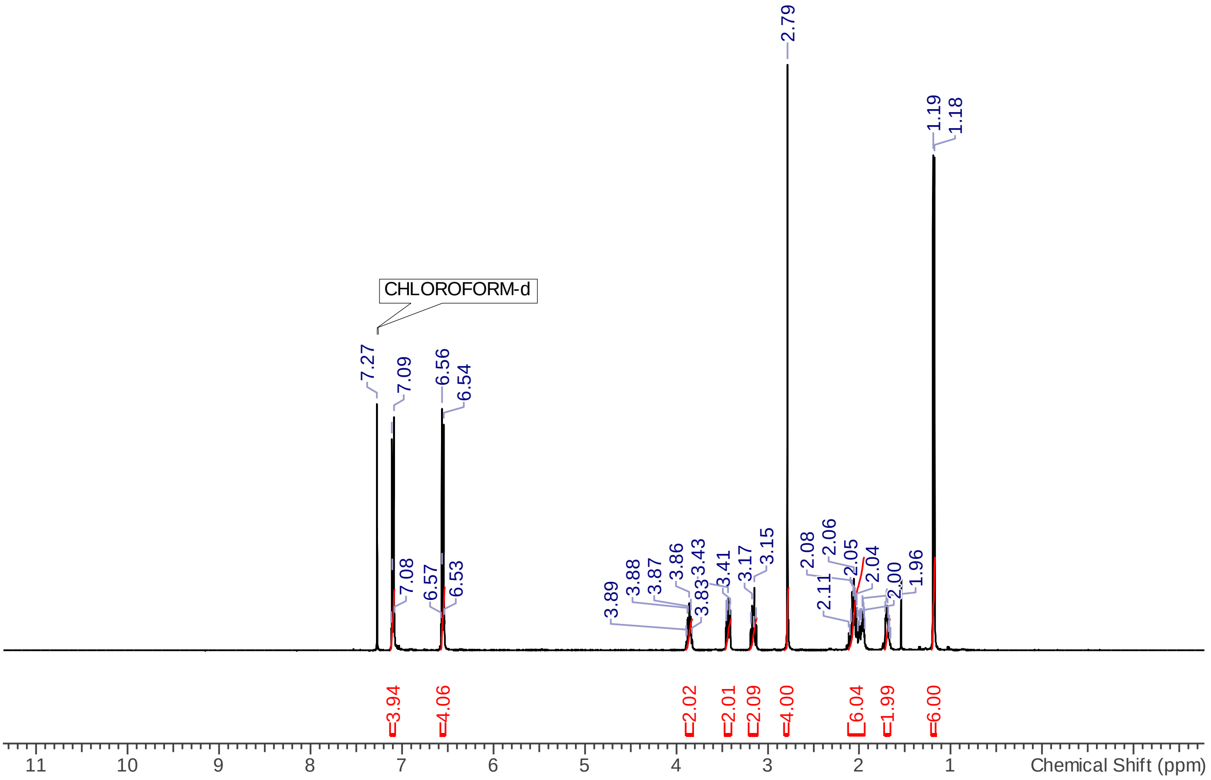

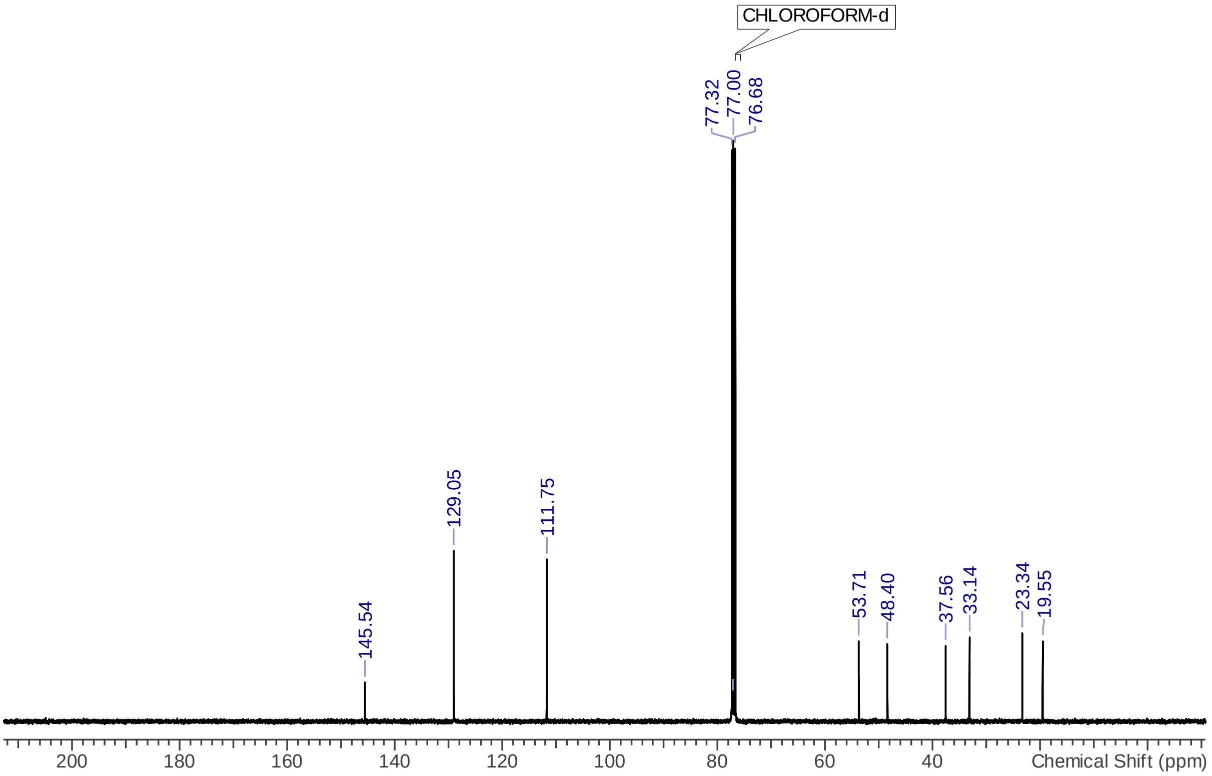

1-(4-Bromonaphthalen-1-yl)-2-methylpyrrolidine 1aa

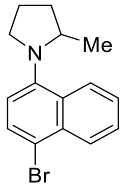

The title compound was prepared according to general procedure 2 using 4-bromonaphthalen-1-amine (800 mg, 3.60 mmol), K_2CO_3 (1.49 g, 10.8 mmol), 1,4-dibromopentane (0.45 mL, 3.28 mmol), and DMF (10 mL). Purification by flash column chromatography on silica gel (eluent = 20% DCM in pet. ether), gave the title compound **1aa** as an orange oil (254 mg, 0.87 mmol, 25%). **R$_f$** = 0.6 (eluent = 20% DCM in pet. ether); **^{1}H NMR** (400 MHz, $CDCl_3$) δ_H = 8.3 (1H, br. d, *J* = 8.3 Hz), 8.2 (1H, br. d, *J* = 8.2 Hz), 7.67 (1H, d, *J* = 8.2 Hz), 7.57 (1H, ddd, *J* = 8.2, 6.8, 1.3 Hz), 7.50 (1H, ddd, *J* = 8.3, 6.8, 1.3 Hz), 6.90 (1H, d, *J* = 8.2 Hz), 3.82 (1H, app. dt, *J* = 9.3, 7.1 Hz), 3.76 (1H, app. dquin, *J* = 8.1, 6.1 Hz), 2.91 (1H, ddd, *J* = 9.3, 8.5, 5.1 Hz), 2.24 (1H, dddd, *J* = 11.8, 8.5, 6.5, 3.4 Hz), 2.08–1.97 (1H, m), 1.93–1.83 (1H, m), 1.76–1.68 (1H, app. dq, *J* = 11.9, 8.8 Hz), 1.07 (3H, d, *J* = 6.1 Hz); **^{13}C NMR** (101 MHz, $CDCl_3$) δ_C = 147.1 (C), 132.8 (C), 131.37 (C), 129.7 (CH), 127.3 (CH), 127.0 (CH), 125.3 (CH), 125.1 (CH), 115.3 (C), 114.8 (CH), 55.8 (CH), 55.5 (CH_2), 33.6 (CH_2), 23.6 (CH_2), 18.6 (CH_3); **HRMS** (ESI$^+$) calculated for $[C_{15}H_{17}N^{79}Br]^+$ (M+H)$^+$ m/z: 290.0544; found 290.0536; calculated for $[C_{15}H_{17}N^{79}Br]^+$ (M+H)$^+$ m/z: 292.0524; found 292.0525.

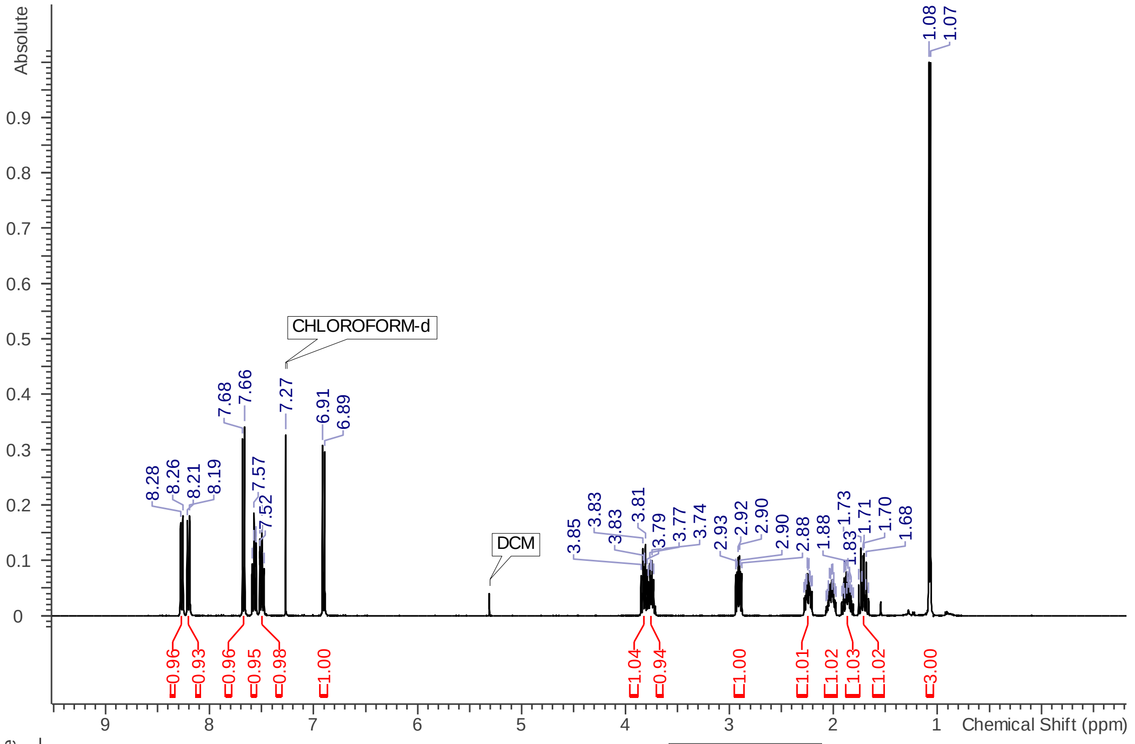

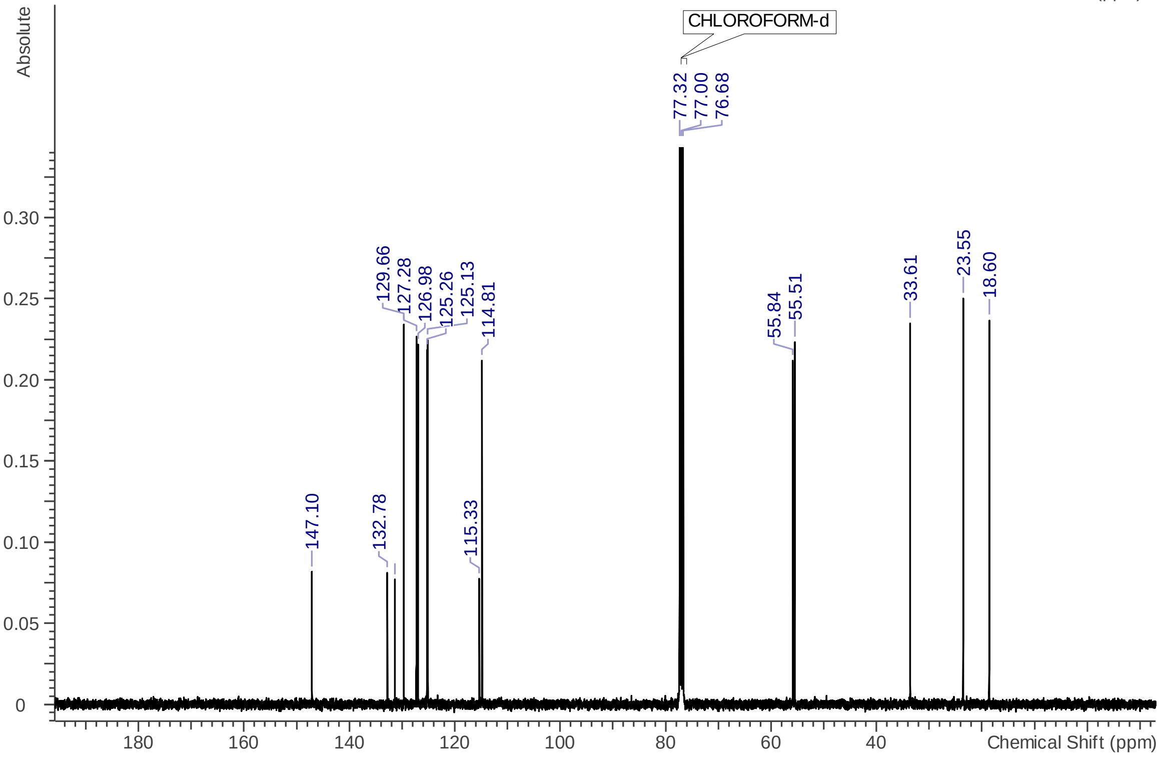

1-(*tert*-Butyl)pyrrolidine 1ab

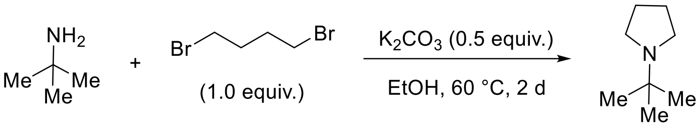

A 50 mL round bottomed flask equipped with stirrer bar and reflux condenser was charged with K_2CO_3 (1.28 g, 9.25 mmol), 1,4-dibromobutane (2.21 mL, 18.5 mmol) and EtOH (1.80 mL). *Tert*-butylamine (1.35 g, 18.5 mmol) was carefully added into the reaction mixture, before the reaction was stirred at 60 °C for 2 days. Upon completion, the mixture was distilled under reduced pressure to give the title compound **1ab** as a colourless liquid (1.50 g, 11.8 mmol, 64%). **^{1}H NMR** (400 MHz, $CDCl_3$) δ_H = 2.69–2.57 (4H, m), 1.81–1.68 (4H, m), 1.09 (9H, s); **^{13}C NMR** (101 MHz, $CDCl_3$) δ_C = 52.2 (C), 45.9 (2×CH_2), 26.0 (3×CH_3), 23.9 (2×CH_2); Spectroscopic data in accordance with that stated in the literature.[7]

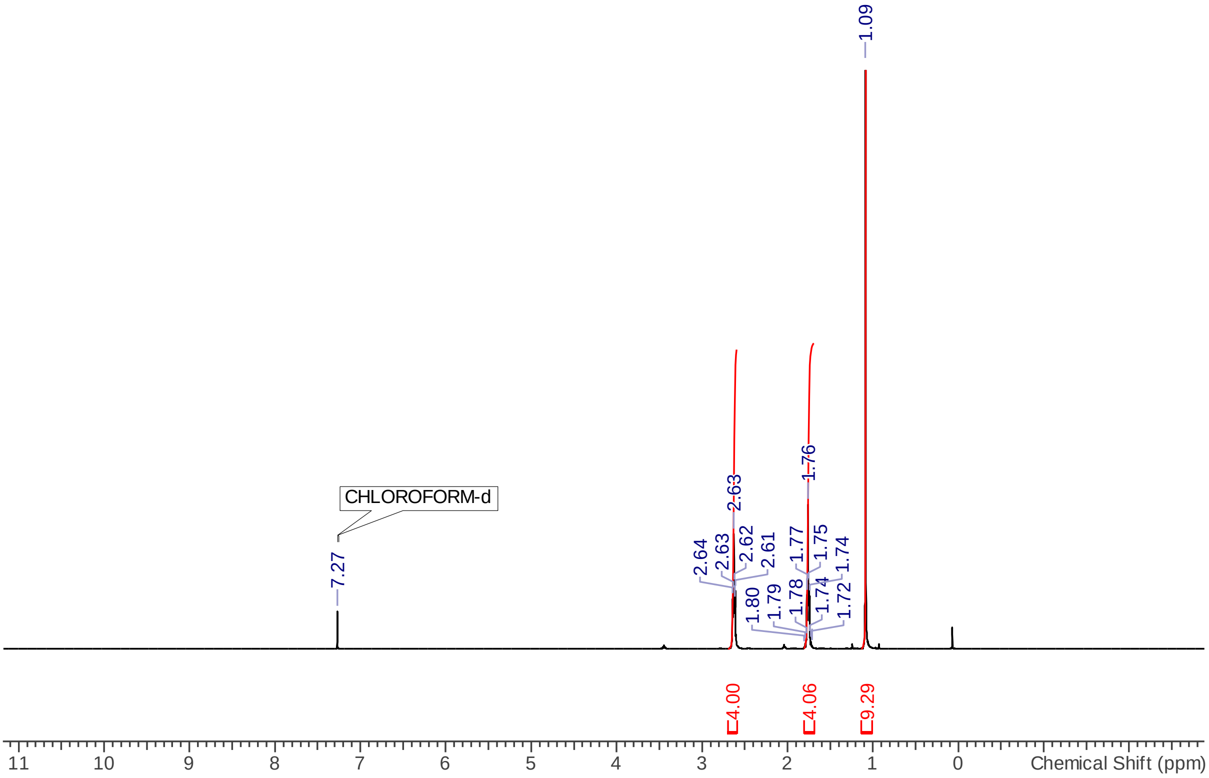

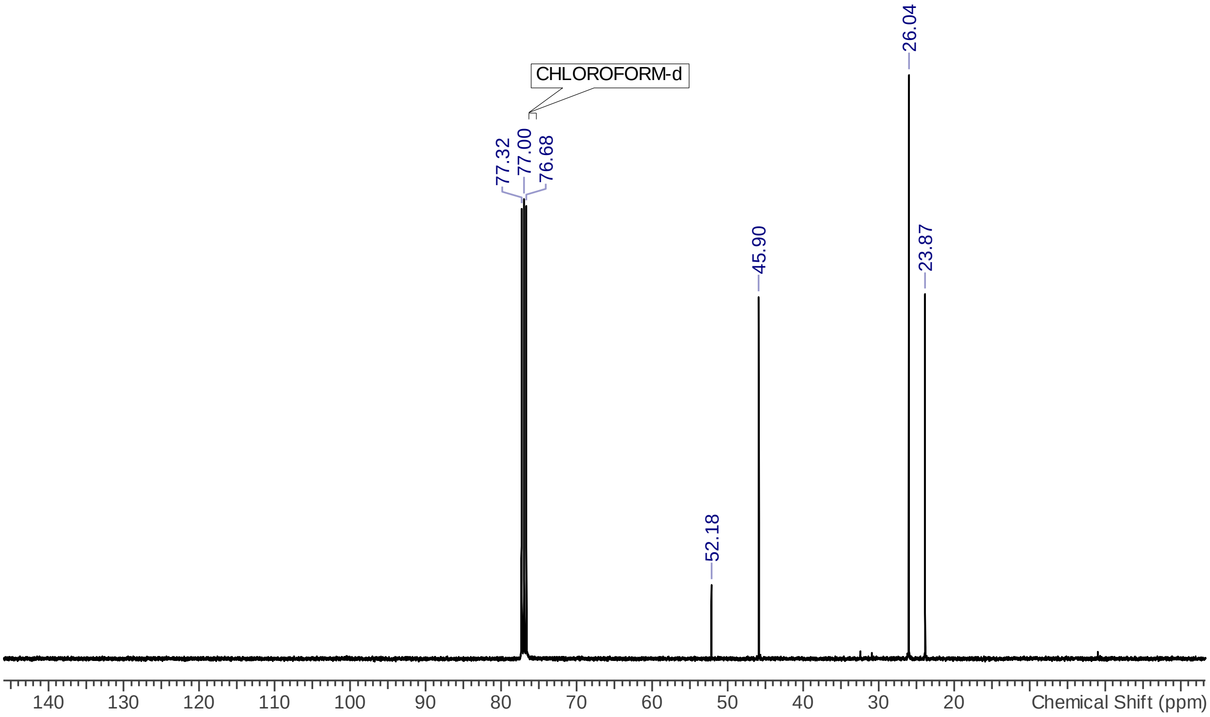

1-Benzyl-2,5-dimethylpyrrolidine 1ac

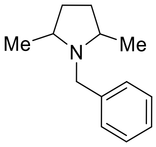

The title compound was prepared according to general procedure 3 using benzylamine (1.50 g, 14.0 mmol), AcOH (0.88 mL, 15.4 mmol), KOH (196 mg, 3.5 mmol), 2,5-hexadione (1.64 mL, 14.0 mmol) and $NaBH_3CN$ (1.06 g, 16.8 mmol). Purification by flash column chromatography on silica gel (eluent = 5% EtOAc in pet. ether), gave the title compound **1ac** as an orange oil (400 mg, 2.11 mmol, 15%, 61:39 mixture of diastereomers). $\mathbf{R_f}$ = 0.22 (eluent = 5% EtOAc in pet. ether); **^{1}H NMR** (400 MHz, $CDCl_3$) **Common signals:** δ_H = 1.43–1.33 (2H, m); **Major diastereomer:** δ_H = 7.33–7.28 (5H, m), 3.76 (2H, s), 2.61–2.56 (2H, m), 1.82–1.74 (2H, m), 1.06 (6H, d, *J* = 6.1 Hz); **Minor diastereomer: ^{1}H NMR** (400 MHz, $CDCl_3$) δ_H = 7.40–7.38 (2H, m), 7.25–7.22 (3H, m), 3.84 (1H, d, *J* = 13.8 Hz), 3.53 (1H, d, *J* = 13.8 Hz), 3.07–3.00 (2H, m), 2.05–1.98 (2H, m), 0.98 (d, *J* = 6.1 Hz, 6H, CH_3); **^{13}C NMR** (101 MHz, $CDCl_3$) δ_C = 140.8 (C)*, 139.3 (C), 129.3 (2×CH), 128.5 (2×CH)*, 128.0 (2×CH)*, 127.9 (2×CH), 126.6 (CH), 126.4 (CH)*, 59.5 (2×CH)*, 55.0 (2×CH), 54.9 (CH_2)*, 51.7 (CH_2), 31.2 (2×CH_2), 30.9 (2×CH_2)*, 20.6 (2×CH_3), 17.0 (2×CH_3)*. Spectroscopic data in accordance with that stated in the literature.[2] **Signals attributed to minor diastereomer.*

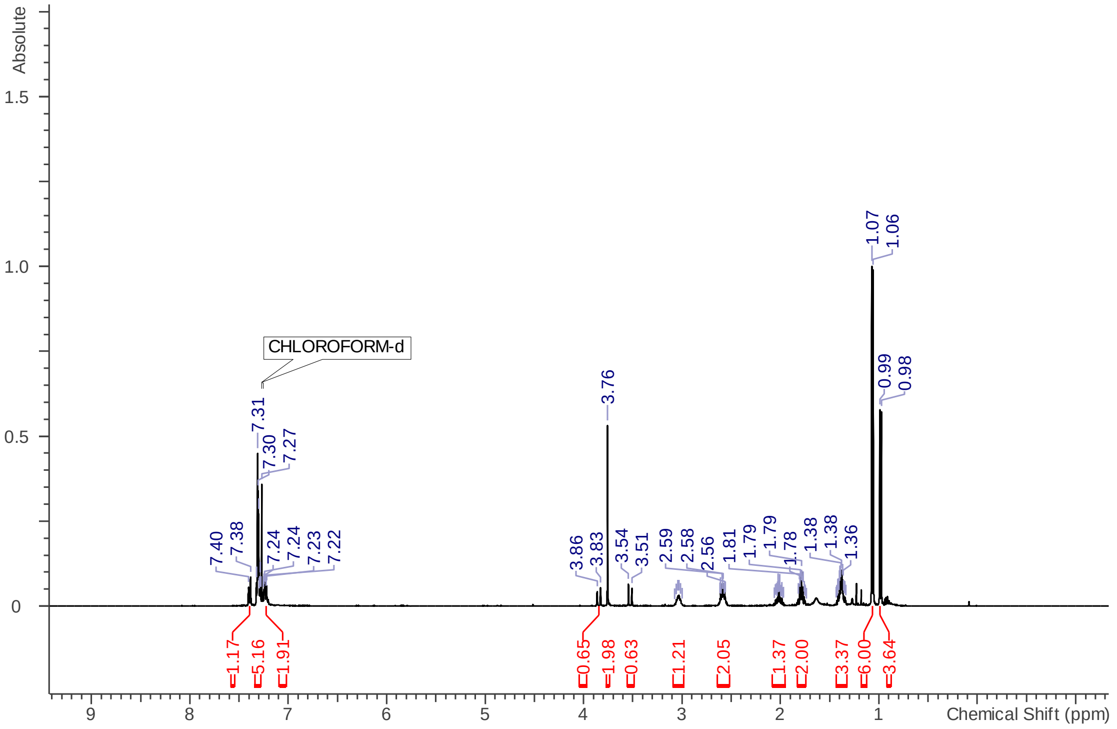

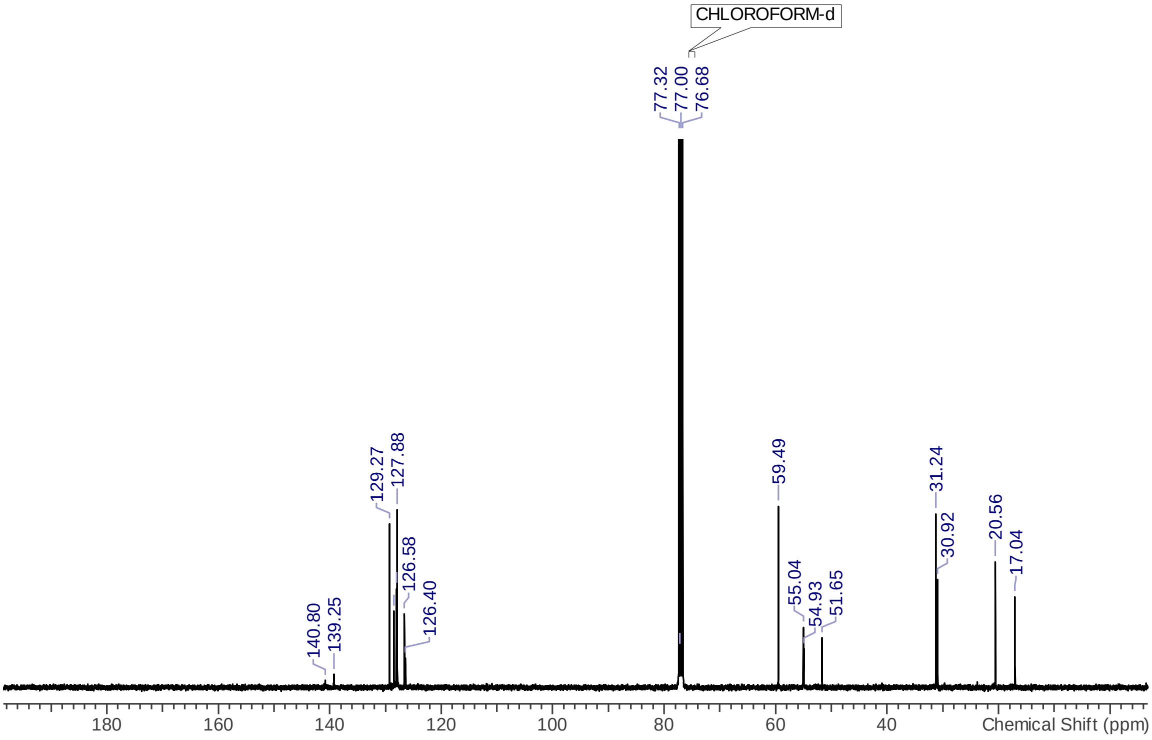

2,5-Dimethyl-1-(3-phenylpropyl)pyrrolidine 1ad

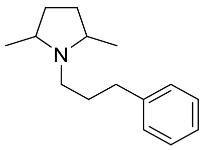

The title compound was prepared according to general procedure 3 using 3-phenylpropan-1-amine (1.00 g, 7.41 mmol), AcOH (0.47 mL, 8.14 mmol), KOH (26 mg, 0.46 mmol), 2,5-hexadione (0.87 mL, 7.4 mmol) and $NaBH_3CN$ (465 mg, 7.4 mmol). Purification by flash column chromatography on silica gel (eluent = 10% MeOH in 1:1 pet. ether:DCM), gave the title compound **1ad** as a brown oil (780 mg, 3.59 mmol, 48%). **R$_f$** = 0.34 (eluent = 10% MeOH in 1:1 pet. ether:DCM); **^{1}H NMR** (400 MHz, $CDCl_3$) δ_H = 7.32–7.26 (2H, m), 7.23–7.15 (3H, m), 2.68-2.54 (6H, m), 1.86–1.74 (4H, m), 1.40–1.30 (2H, m), 1.09 (6H, d, *J* = 6.1 Hz); **^{13}C NMR** (101 MHz, $CDCl_3$) δ_C = 142.4 (C), 128.3 (4×CH), 125.7 (CH), 59.9 (2×CH), 51.2 (CH_2), 34.1 (CH_2), 31.5 (2×CH_2), 28.7 (CH_2), 20.9 (2×CH_3); **HRMS** (ESI$^+$): calculated for $[C_{15}H_{24}N]^+$ (M+H)$^+$ m/z: 218.1909; found 218.1910.

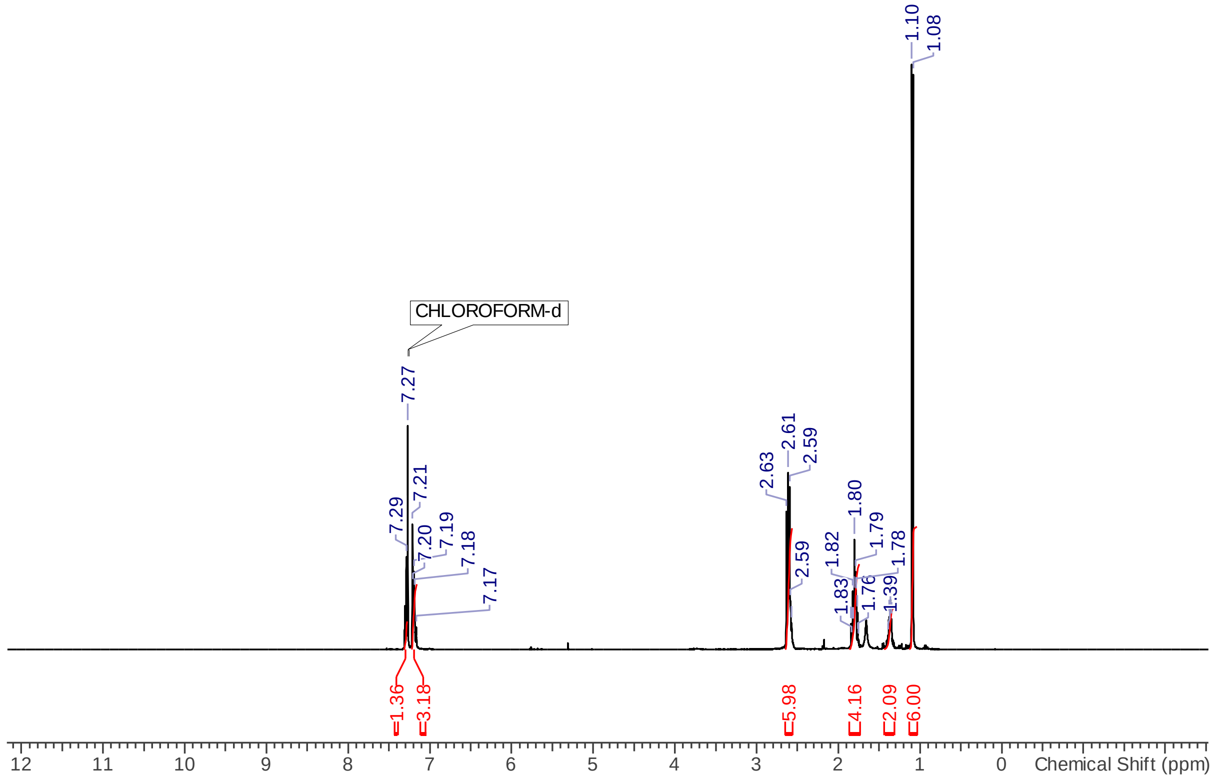

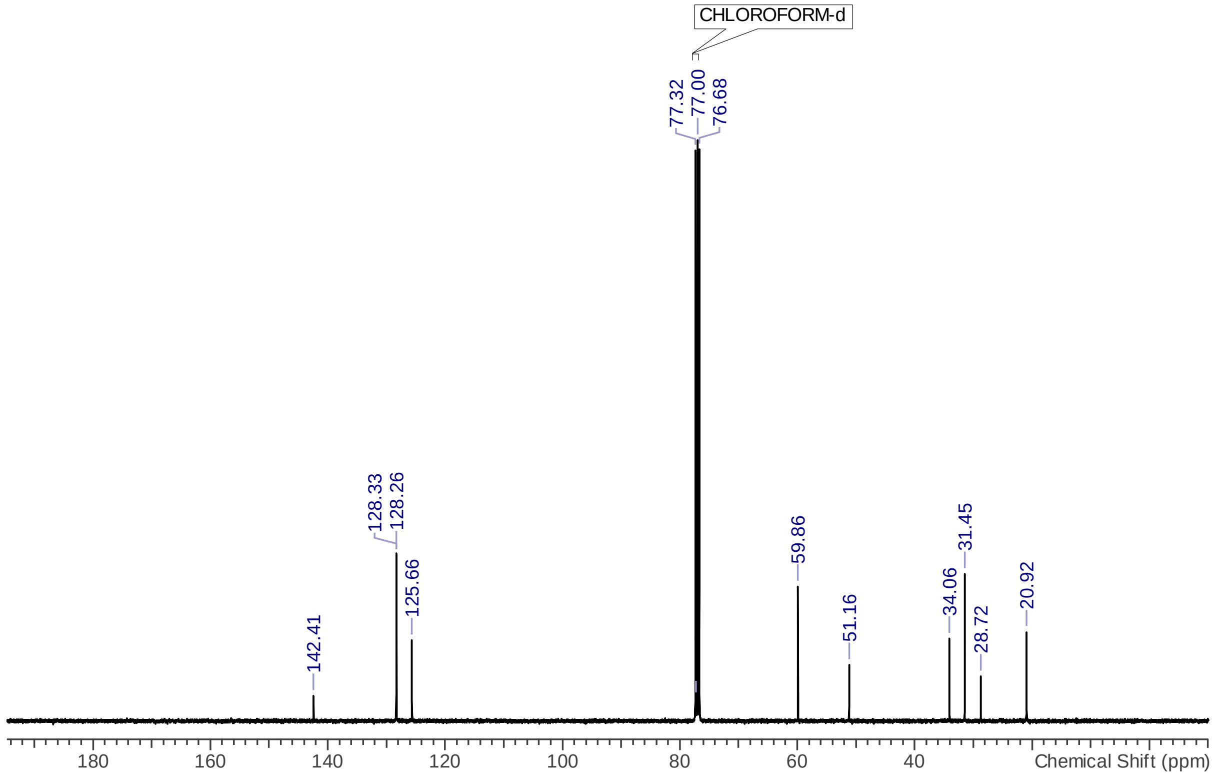

2,5-Dimethyl-1-(thiophen-2-ylmethyl)pyrrolidine 1ae

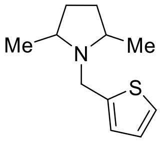

The title compound was prepared according to general procedure 3 using 2-thiophenemethylamine (1.00 g, 8.85 mmol), AcOH (0.57 mL, 9.73 mmol), 2,5-hexadione (1.03 mL, 8.85 mmol) and $NaBH_3CN$ (556 mg, 8.85 mmol). Purification by flash column chromatography on silica gel (eluent = 5% MeOH in 1:1 pet. ether:DCM), gave the title compound **1ae** as a brown oil (320 mg, 1.64 mmol, 19%, 56:44 mixture of diastereomers). **R_f** = 0.27 (eluent = 5% MeOH in 1:1 pet. ether:DCM); **^{1}H NMR** (400 MHz, $CDCl_3$) **Common signals:** δ_H =7.23–7.17 (1H, m), 1.44–1.32 (2H, m); **Major diastereomer:** δ_H = 6.97 (1H, dd, *J* = 5.1, 3.4 Hz), 6.87 (1H, d, *J* = 3.4 Hz), 4.05 (2H, s) 2.68–2.57 (2H, m), 1.81–1.72 (2H, m), 1.15 (6H, d, *J* = 6.1 Hz); **Minor diastereomer: ^{1}H NMR** δ_H = 6.95–6.91 (2H, m), 3.94 (1H, d, *J* = 14.2 Hz), 3.82 (1H, d, *J* = 14.2 Hz), 3.16–3.06 (2H, m) 2.08–1.95 (2H, m), 1.01 (6H, d, *J* = 6.3Hz); **^{13}C NMR** (101 MHz, $CDCl_3$) δ_C = 145.0 (C)*, 139.6 (C), 126.43 (CH)*, 126.40 (CH), 126.1 (CH)*, 124.5 (CH), 124.3 (CH)*, 124.1 (CH), 57.4 (2×CH), 55.1 (2×CH)*, 46.5 (CH_2), 46.4 (CH_2)*, 30.8 (2×CH_2)†, 19.7 (2×CH_3), 17.0 (2×CH_3)*; **HRMS** (ESI$^+$): calculated for $[C_{11}H_{18}NS]^+$ $(M+H)^+$ m/z: 196.1160; found 196.1163. **Signals attributed to minor diastereomer. †Signal attributed to both major and minor diastereomers.*

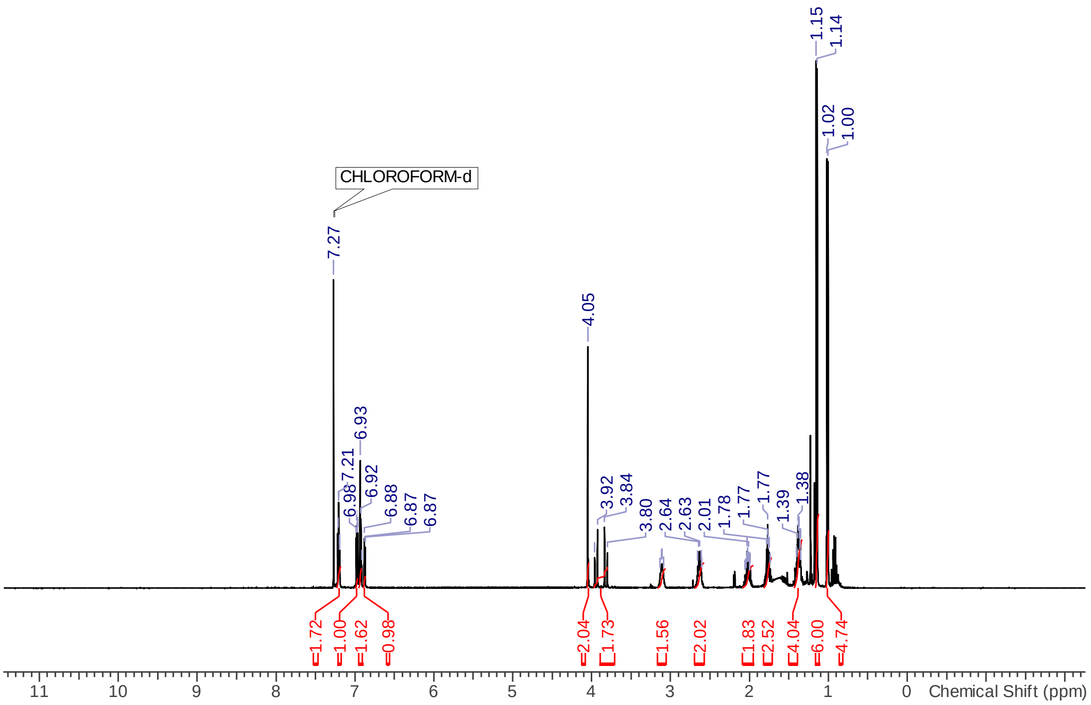

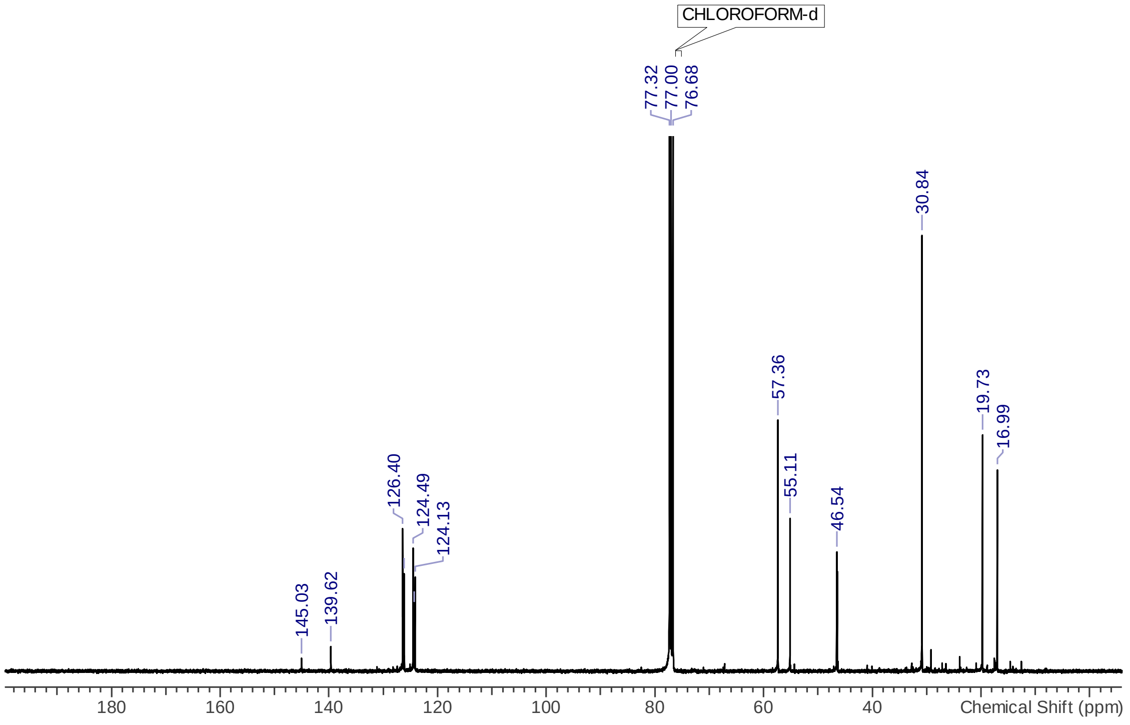

1-(1-Phenylcyclohexyl)pyrrolidine (rolicyclidine) 1af

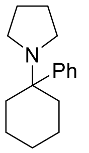

A 100 mL 3-neck round bottomed flask equipped with Dean-Stark apparatus and stirrer bar was charged with 1*H*-1,2,3-triazole (1.7 mL, 30 mmol), PhMe (25 mL), pyrrolidine (2.3 mL, 28 mmol), and cyclohexanone (2.6 mL, 25 mmol). The reaction was then heated to 135 °C for 8 h, before the reaction mixture was cooled to room temperature, and stored under a nitrogen atmosphere for 18 h. A 500 mL 3-neck round bottomed flask equipped with stirrer bar and reflux condenser was charged with magnesium turnings (3.26 g, 136 mmol) under a nitrogen atmosphere. Two crystals of iodine were added and stirred vigorously for 10 minutes to activate the magnesium, before THF (100 mL) was added. PhBr (10.5 mL, 100 mmol) was then added slowly, leading to heating and some reflux, before the reaction was further heated to reflux for 1.5 h. Upon cooling, the prepared solution of Grignard reagent was transferred to a 500 mL 2-neck round bottomed flask equipped with stirrer bar by cannula filtration. The prepared iminium solution in toluene was then added slowly to the Grignard reagent by syringe, before the reaction mixture was stirred for 1 h at ambient temperature. Subsequently, the reaction was quenched by the addition of NH_4Cl (aq., 20%, 60 mL), before the phases were separated. The aqueous phase was extracted with EtOAc (100 mL), before the combined organics were washed with $NaHCO_3$ (aq., 2 M, 50 mL) and water (50 mL), dried with $MgSO_4$, filtered and concentrated *in vacuo*. The crude materials were then purified by flash column chromatography on silica gel (eluent = 1.5% Et_3N in 4:1 pet. ether:EtOAc) to yield the desired product **1af** as a yellow solid (1.95 g, 8.5 mmol, 34%). **Rf** = 0.09 (eluent = 1% Et_3N in 4:1 pet. ether:EtOAc); **^{1}H NMR** (400 MHz, $CDCl_3$) δ_H = 7.41–7.32 (4H, m), 7.27–7.22 (1H, m), 2.47–2.40 (4H, m), 2.38–2.28 (2H, m), 1.89 (2H, ddd, *J* = 13.7, 11.0, 3.4 Hz), 1.72–1.60 (2H, m), 1.60–1.55 (4H, m), 1.51–1.23 (4H, m); **^{13}C NMR** (126 MHz, $CDCl_3$) δ_C = 138.0 (C), 127.9 (2×CH), 127.5 (2×CH), 126.1 (CH), 59.9 (C), 44.7 (2×CH_2), 34.9 (2×CH_2), 26.33 (CH_2), 22.8 (2×CH_2), 22.5 (2×CH_2). **HRMS** (ESI$^+$): calculated for $[C_{16}H_{24}N]^+$ $(M+H)^+$ m/z: 230.1909; found 239.1910.

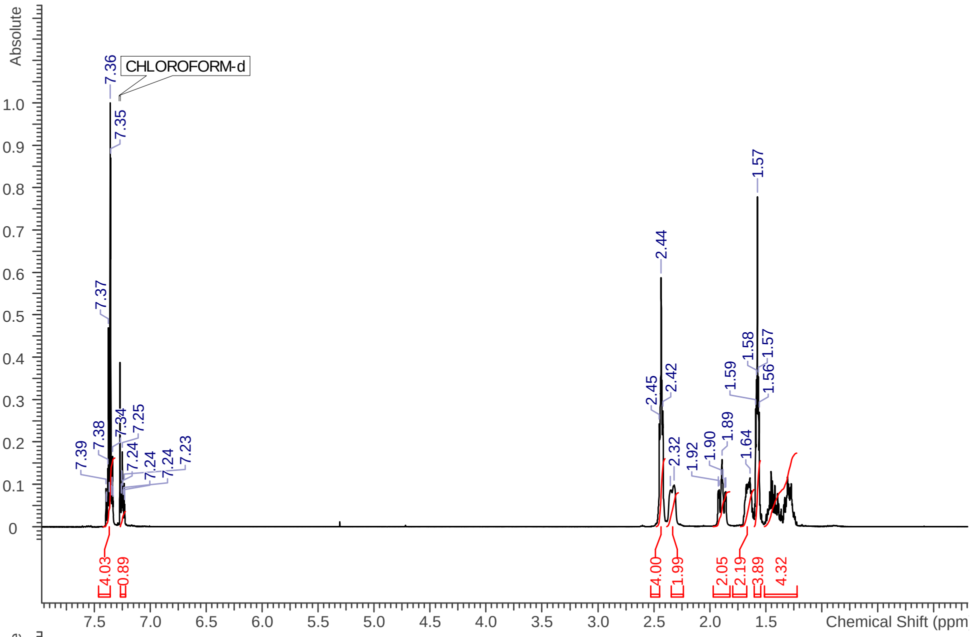

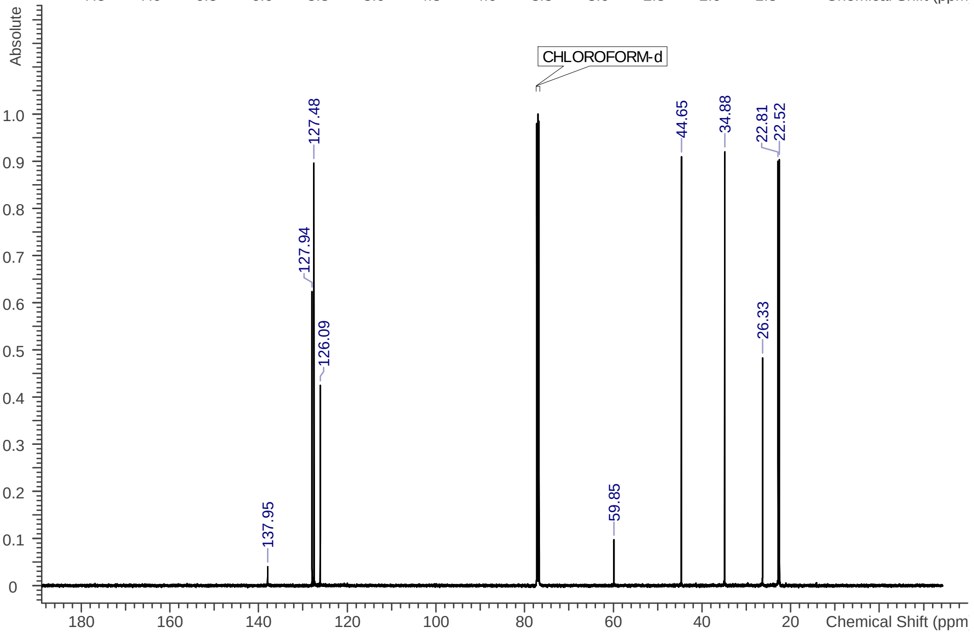

Estrone 3-methyl ether S5

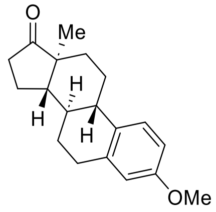

A 50 mL 2-neck round bottomed flask equipped with stirrer bar was charged with estrone (541 mg, 2.0 mmol) under a nitrogen atmosphere, before the addition of THF (15 mL). NaH (96 mg, 60% suspension in mineral oil, 2.4 mmol) was then added, with MeI (0.62 mL, 10 mmol) added upon cessation of effervescence. After stirring at ambient temperature for 18 h, more MeI (0.62 mL, 10 mmol) was added, before the reaction was allowed to stir at room temperature for a further 24 h. The reaction was then quenched by addition to brine (20 mL), before being extracted with EtOAc (3 × 30 mL). The combined organics were dried with $MgSO_4$, filtered, and concentrated *in vacuo* to yield the desired product **S5** as a yellow solid (580 mg, 2.0 mmol, quantitative). **^{1}H NMR** (400 MHz, $CDCl_3$) δ_H = 7.22 (1H, d, *J* = 8.6 Hz), 6.73 (1H, dd, *J* = 8.6, 2.7 Hz), 6.66 (1H, d, *J* = 2.7 Hz), 3.79 (3H, s), 2.95–2.87 (2H, m), 2.51 (1H, dd, *J* = 18.8, 8.5 Hz), 2.44–2.37 (1H, m), 2.31–2.22 (1H, m), 2.20–1.90 (4H, m), 1.71–1.40 (6H, m), 0.92 (3H, s); **^{13}C NMR** (126 MHz, $CDCl_3$) δ_C = 220.9 (C), 157.6 (C), 137.7 (C), 132.1 (C), 126.3 (CH), 113.9 (CH), 111.6 (CH), 55.2 (CH_3), 50.4 (CH), 48.0 (C), 44.0 (CH), 38.4 (CH), 35.9 (CH_2), 31.6 (CH_2), 29.7 (CH_2), 26.5 (CH_2), 25.9 (CH_2), 21.6 (CH_2), 13.8 (CH_3). Spectroscopic data in accordance with that stated in the literature.[28]

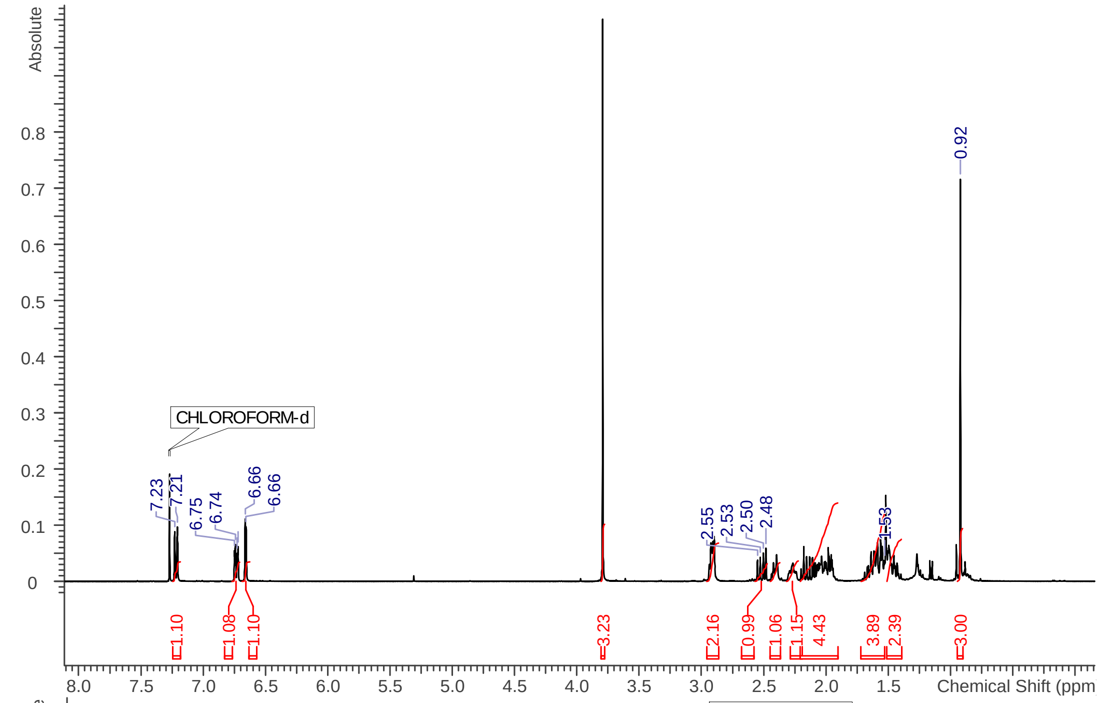

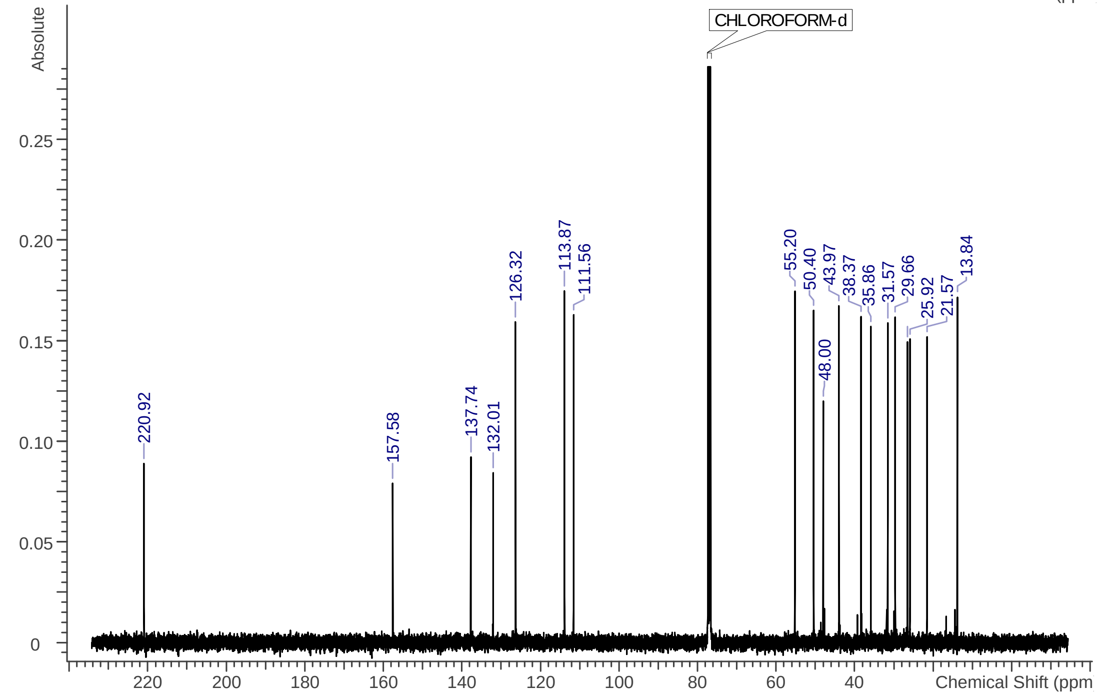

1-(3-Methoxy-14-methyl-7,8,9,11,12,13,14,15,16,17-decahydro-6H-cyclopenta[a]phenanthren-15-yl)pyrrolidine 1ag

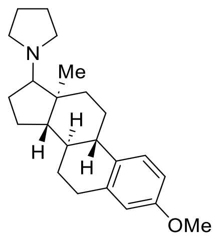

A 25 mL J. Youngs flask equipped with stirrer bar was charged with estrone 3-methyl ether **S5** (400 mg, 1.4 mmol), which was dissolved in formic acid (2.3 mL, 120 mmol). Pyrrolidine (3.4 mL, 82 mmol) was then added dropwise, leading to fuming. The reaction flask was then sealed, and heated to 145 °C for 18 h. Upon completion, the reaction was diluted with DCM (10 mL), then washed with water (3 × 10 mL), dried with Na_2SO_4, filtered, and concentrated *in vacuo*. The crude materials were then purified by flash column chromatography on silica gel (eluent = 1% Et_3N in 1:1 pet. ether:EtOAc) to yield the desired product **1ag** as a cream solid (302 mg, 0.89 mmol, 64%, 90:10 mixture of diastereomers). **Rf** = 0.25 (eluent = 1% Et_3N in 1:1 pet. ether:EtOAc); **^{1}H NMR** (500 MHz, $CDCl_3$) **Common signals:** δ_H = 7.21 (1H, d, *J* = 8.6 Hz), 6.72 (1H, dd, *J* = 8.6, 2.7 Hz), 6.64 (1H, d, *J* = 2.7 Hz), 3.78 (3H, s), 2.94–2.79 (2H, m), 2.67–2.46 (4H, m), 2.31–2.23 (1H, m), 2.19 (1H, app. td, *J* = 10.9, 4.2 Hz), 2.12 (1H, app. dt, *J* = 12.5, 3.2 Hz), 2.06–1.97 (1H, m), 1.96–1.84 (2H, m), 1.83–1.62 (6H, m), 1.53 (1H, app. qd, *J* = 13.0, 3.7 Hz), 1.47–1.25 (5H, m); **Major diastereomer:** δ_H = 0.86 (3H, s); **Minor diastereomer:** δ_H = 0.89 (3H, s); **^{13}C NMR** (126 MHz, $CDCl_3$) δ_C = 157.4 (C)†, 138.0 (CH)†, 132.7 (C)†, 126.3 (CH)†, 113.7 (CH)†, 111.4 (CH)†, 85.1 (CH)*, 77.4 (CH), 55.2 (CH_3)†, 54.4 (2×CH_2)*, 54.1 (2×CH_2), 52.6 (CH), 50.4 (CH)*, 45.8 (CH)*, 43.7 (CH), 43.6 (C)†, 39.6 (CH_2)*, 39.1 (CH_2), 38.9 (CH), 38.3 (CH)*, 34.5 (CH_2)*, 29.9 (CH_2), 29.4 (CH_2)*, 29.3 (CH_2), 27.6 (CH_2)*, 27.5 (CH_2), 26.7 (CH_2)†, 23.8 (CH_2)*, 23.5 (CH_2), 23.1 (2×CH_2), 23.0 (2×CH_2)*, 13.3 (CH_3)*, 12.3 (CH_3); **HRMS** (ESI$^+$): calculated for $[C_{23}H_{34}NO]^+$ (M+H)$^+$ m/z: 340.2640; found 340.2650. **Signals attributed to minor diastereomer. †Signals attributed to both major and minor diastereomers.*

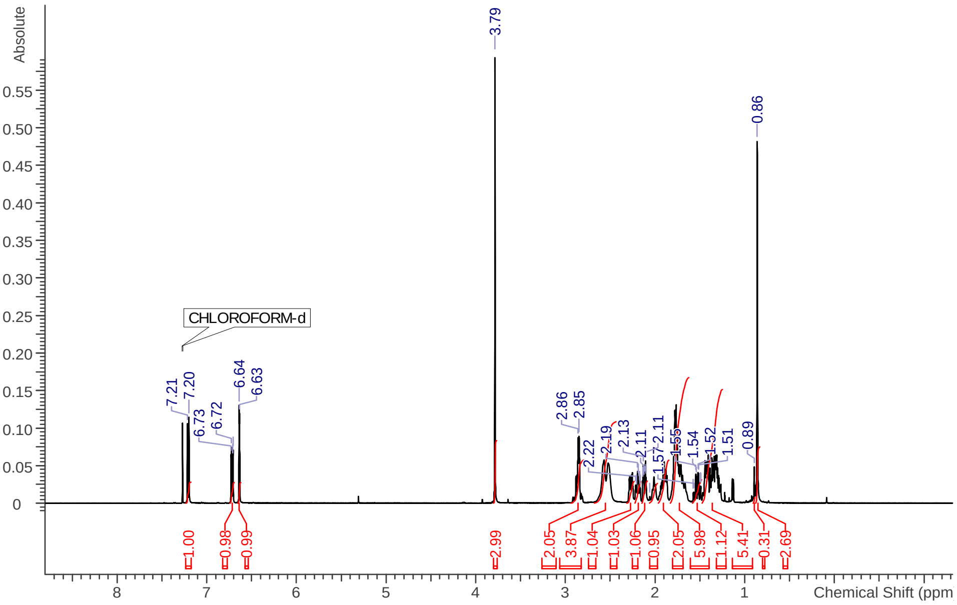

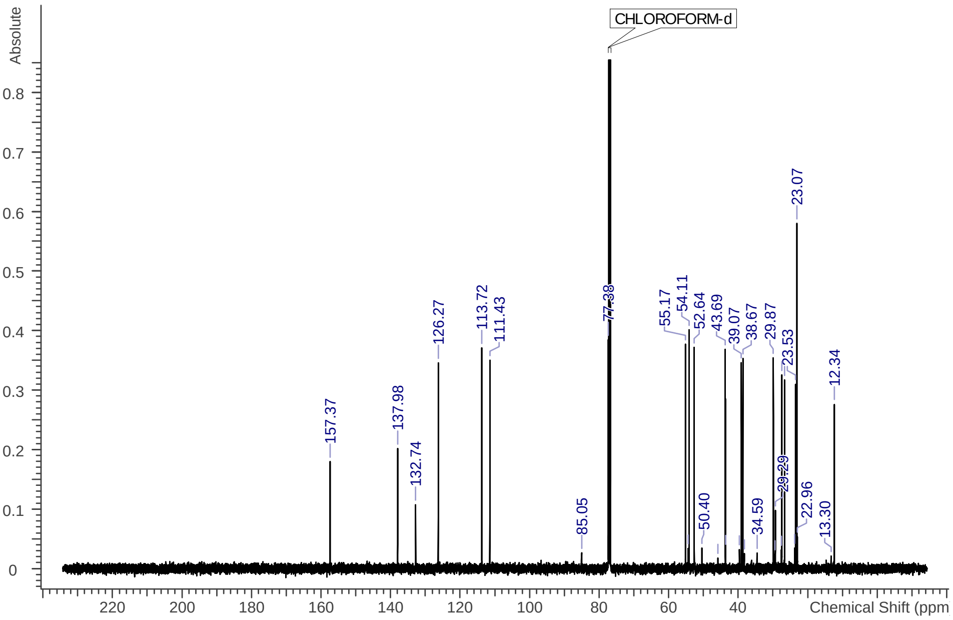

1-(4-(4-Chloro-3,5-dimethylphenoxy)phenyl)-2-methylpyrrolidine 1ah

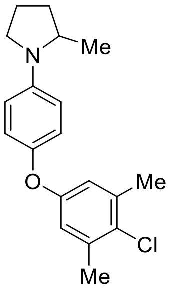

A 100 mL J. Youngs flask equipped with stirrer bar was charged with 1-(4-Bromophenyl)-2-methylpyrrolidine **1k** (285 mg, 1.19 mmol), 4-chloro-3,5-dimethylphenol (373 mg, 2.38 mmol), CuI (62 mg, 0.33 mmol), Cs_2CO_3 (465 mg, 1.43 mmol), and *N*,*N*-dimethylglycine (30.7 mg, 0.30 mmol) under a nitrogen atmosphere. 1,4-Dioxane (20 mL) was added, before the reaction flask was sealed and heated to 114 °C for 17 h. Upon cooling, monitoring by NMR revealed the reaction was incomplete, and so further 4-chloro-3,5-dimethylphenol (180 mg, 1.15 mmol), CuI (56 mg, 0.30 mmol), Cs_2CO_3 (433 mg, 1.32 mmol), and *N*,*N*-dimethylglycine (32 mg, 0.31 mmol) were added to the reaction flask. The flask was resealed, and heated to 125 °C for a further 20 h. Upon completion, the reaction was quenched by addition of water (50 mL), before being extracted with EtOAc (3 × 75 mL). The combined organics were washed with brine (70 mL), dried with $MgSO_4$, filtered, and concentrated *in vacuo*. The crude materials were purified by acid-base extraction: the materials were dissolved in HCl (4N, 15 mL), washed with Et_2O (20 mL), before the aqueous phase was basified by portionwise addition of K_2CO_3. The resulting suspension was extracted with Et_2O (3 × 30 mL), and the combined organics dried with $MgSO_4$, filtered, and concentrated in vacuo to yield the desired product **1ah** as a brown solid (262 mg, 0.83 mmol, 70%). **Rf** = 0.27 (eluent = 10% DCM in pet. ether); **^{1}H NMR** (500 MHz, $CDCl_3$) δ_H = 6.98–6.92 (2H, m), 6.69 (2H, s), 6.61–6.55 (2H, m), 3.88 (1H, app. quind, *J* = 6.3, 1.5 Hz), 3.48–3.42 (1H, m), 3.19 (1H, app. q, *J* = 8.3 Hz), 2.34 (6H, s), 2.16–1.96 (3H, m), 1.79–1.69 (1H, m), 1.22 (3H, d, *J* = 6.3 Hz); **^{13}C NMR** (126 MHz, $CDCl_3$) δ_C = 157.2 (C), 145.7 (C), 144.3 (C), 137.2 (2×C), 127.2 (C), 121.3 (2×CH), 116.7 (2×CH), 112.4 (2×CH), 54.0 (CH), 48.6 (CH_2), 33.2 (CH_2), 23.4 (CH_2) 20.8 (2×CH_3), 19.5 (CH_3); **HRMS** (ESI$^+$): calculated for $[C_{19}H_{23}NO^{35}Cl]^+$ $(M+H)^+$ m/z: 316.1468; found 316.1468; calculated for $[C_{19}H_{23}NO^{37}Cl]^+$ $(M+H)^+$ m/z: 318.1439; found 318.1444.

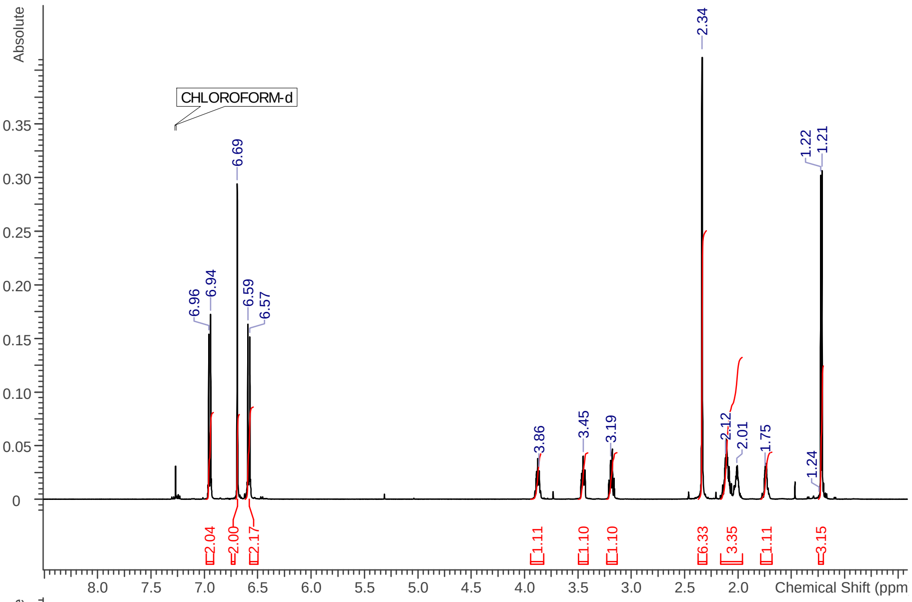

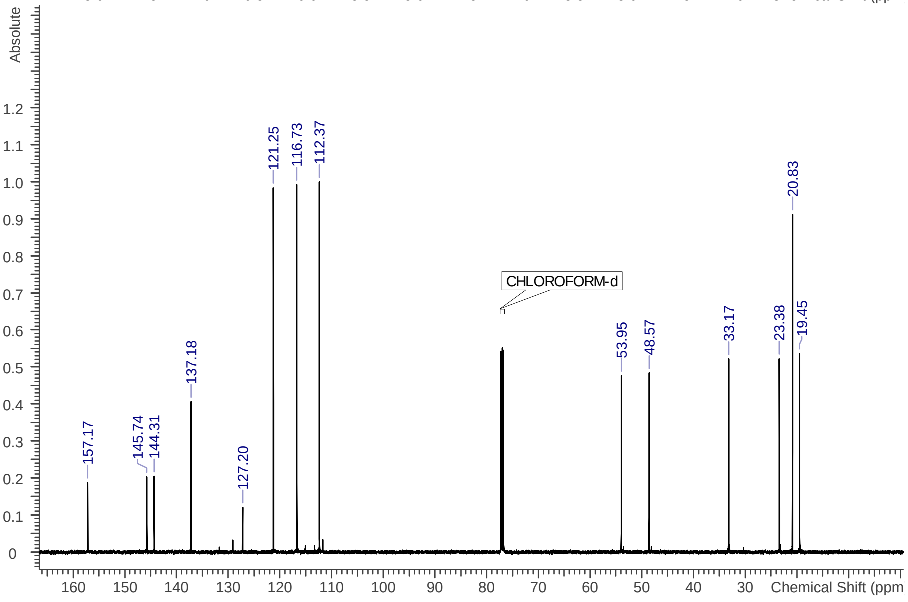

2.2 Synthesis of indolines

1-Methylindoline 4a

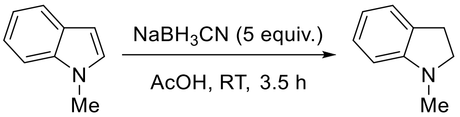

N-Methyl indole (0.25 mL, 2.0 mmol) was dissolved in acetic acid (10 mL) and cooled to 10 °C (water and ice bath). $NaBH_3CN$ (628 mg, 9.99 mmol) was added portionwise, before the reaction was allowed to stir at RT for 3.5 h. Upon completion, the reaction was quenched with water (5 mL), before the acetic acid was removed *in vacuo*. The resultant residue was neutralised with NaOH (aq., 5%), before being extracted with Et_2O (3 × 20 mL). The combined organics were washed with water (50 mL), dried with $MgSO_4$, filtered and concentrated *in vacuo*. The crude materials were then purified by flash column chromatography on silica gel (eluent = 5–10% EtOAc in pet. ether) to yield the desired product **4a** as a yellow oil (75 mg, 28%). **R**$_f$ (eluent = 10% EtOAc in pet. ether) = 0.40, **^{1}H NMR** (400 MHz, $CDCl_3$) δ_H = 7.14–7.05 (2H, m), 6.69 (1H, td, *J* = 7.4, 0.9 Hz), 6.54–6.48 (1H, m), 3.31 (2H, t, *J* = 8.1 Hz), 2.96 (2H, t, *J* = 8.1 Hz), 2.78 (3H, s).**^{13}C NMR** (101 MHz, $CDCl_3$) δ = 153.4 (C), 130.3 (C), 127.3 (CH), 124.2 (CH), 117.7 (CH), 107.2 (CH), 56.1 (CH_2), 36.3 (CH_3), 28.7 (CH_2). Spectroscopic data in accordance with that stated in the literature.[8]

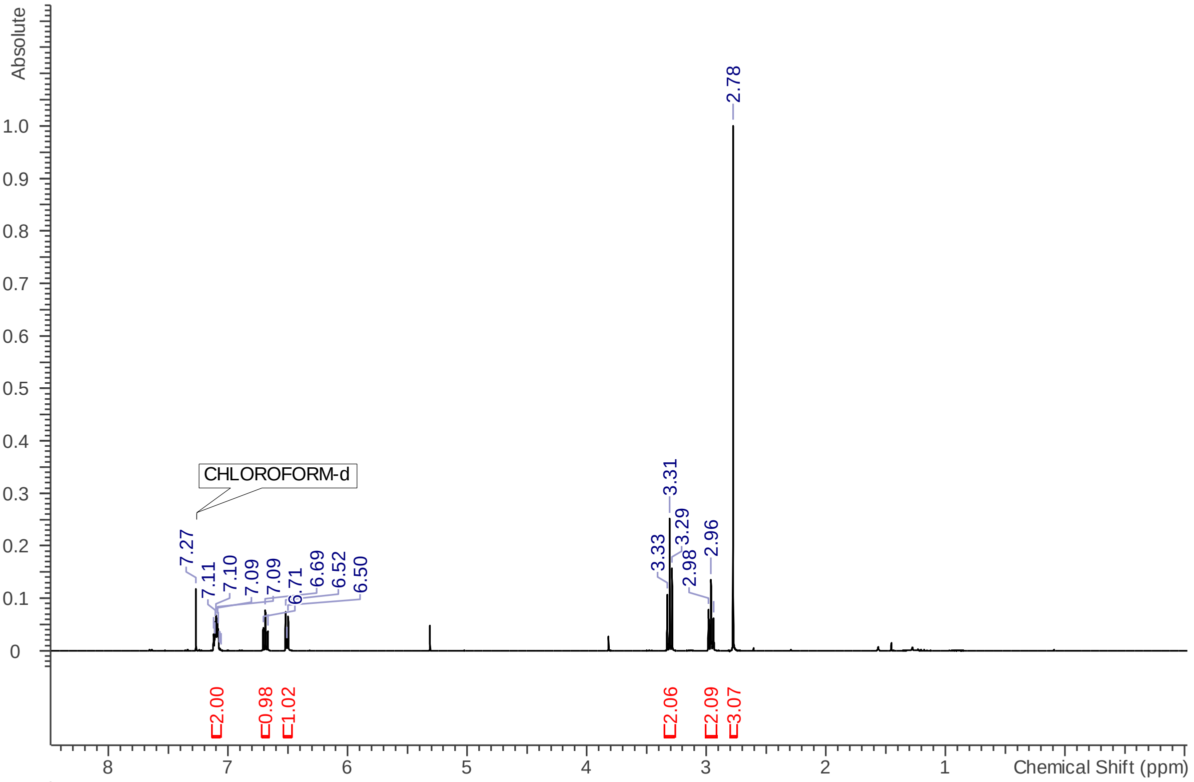

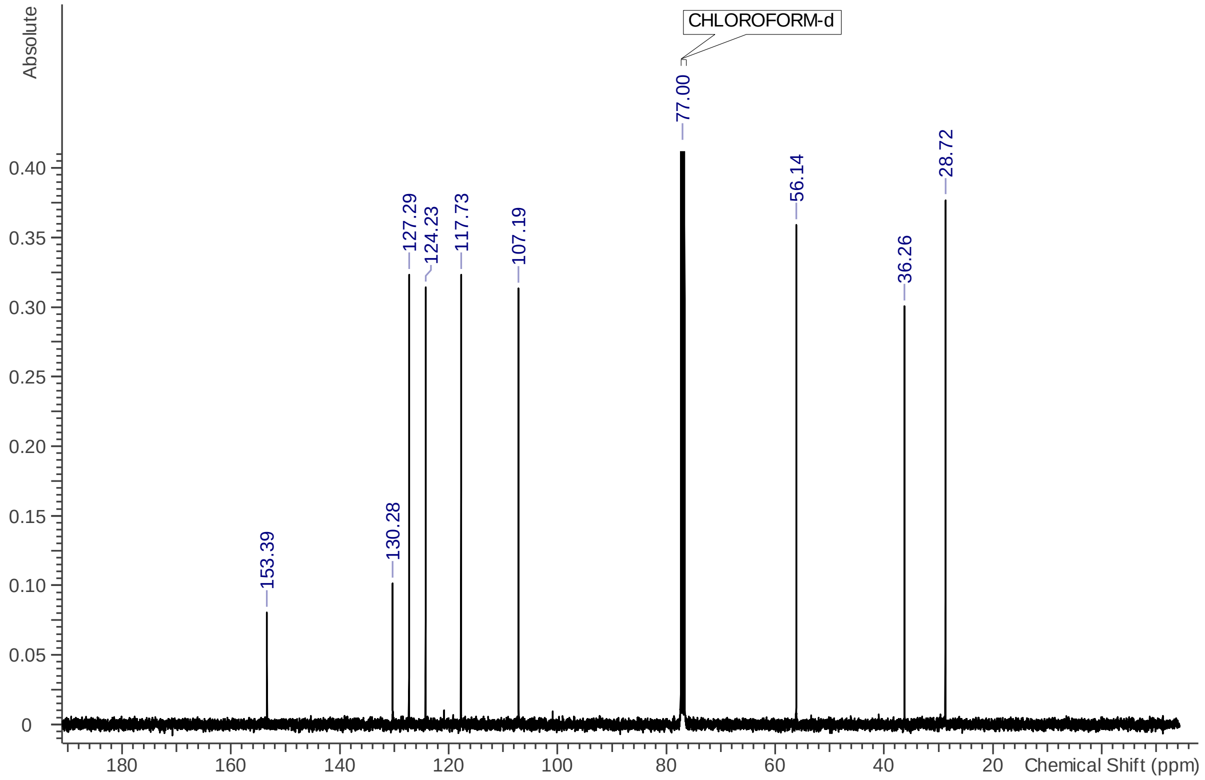

1,2-Dimethyl indoline 4b

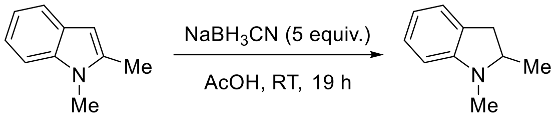

1,2-Dimethylindole (1.02 g, 7.02 mmol) was dissolved in glacial AcOH (25 mL), before $NaBH_3CN$ (2.17 g, 34.5 mmol) was added portionwise at 10 °C (using an ice/water bath). The reaction was allowed to stir at RT for 19 h, before water (15 mL) was added slowly to quench the reaction. The AcOH was removed *in vacuo*, before the resulting solution was basified with NaOH (aq., 15%). The mixture was extracted with Et_2O (3 x 30 mL). The combined organics were washed with water (40 mL) and brine (40 mL), before being dried with $MgSO_4$, filtered, and concentrated *in vacuo*. The crude materials were then purified by flash column chromatography on silica gel (eluent = 5% EtOAc in pet. ether) to yield the desired product **4b** as a yellow oil (180 mg, 1.22 mmol, 17%). **Rf** = 0.38 (eluent = 5% EtOAc in pet. Ether); **^{1}H NMR** (400 MHz, $CDCl_3$) δ_H = 7.14–7.01 (2H, m), 6.68 (1H, td, *J* = 7.3, 0.9 Hz), 6.47 (1H, d, *J* = 7.8 Hz), 3.42 (1H, ddq, *J* = 10.3, 8.2, 6.1 Hz), 3.10 (1H, dd, *J* = 15.3, 8.2 Hz), 2.73 (3H, s), 2.62 (1H, dd, *J* = 15.3, 10.3 Hz), 1.35 (3H, d, *J* = 6.1 Hz); **^{13}C NMR** (101 MHz, $CDCl_3$) δ_C = 153.5 (C), 129.2 (C), 127.3 (CH), 124.0 (CH), 117.8 (CH), 107.1 (CH), 62.8 (CH), 37.4 (CH_3), 33.7 (CH_2), 18.8 (CH_3). Spectroscopic data in accordance with that stated in the literature.[8]

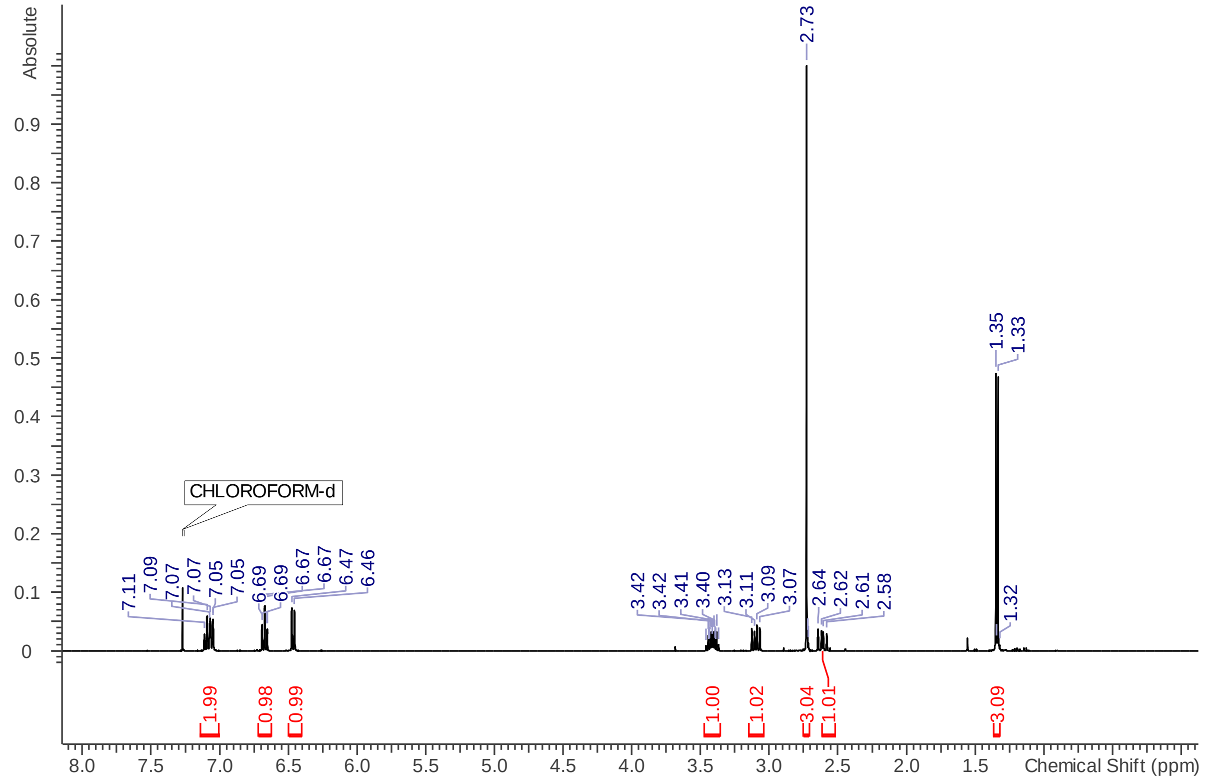

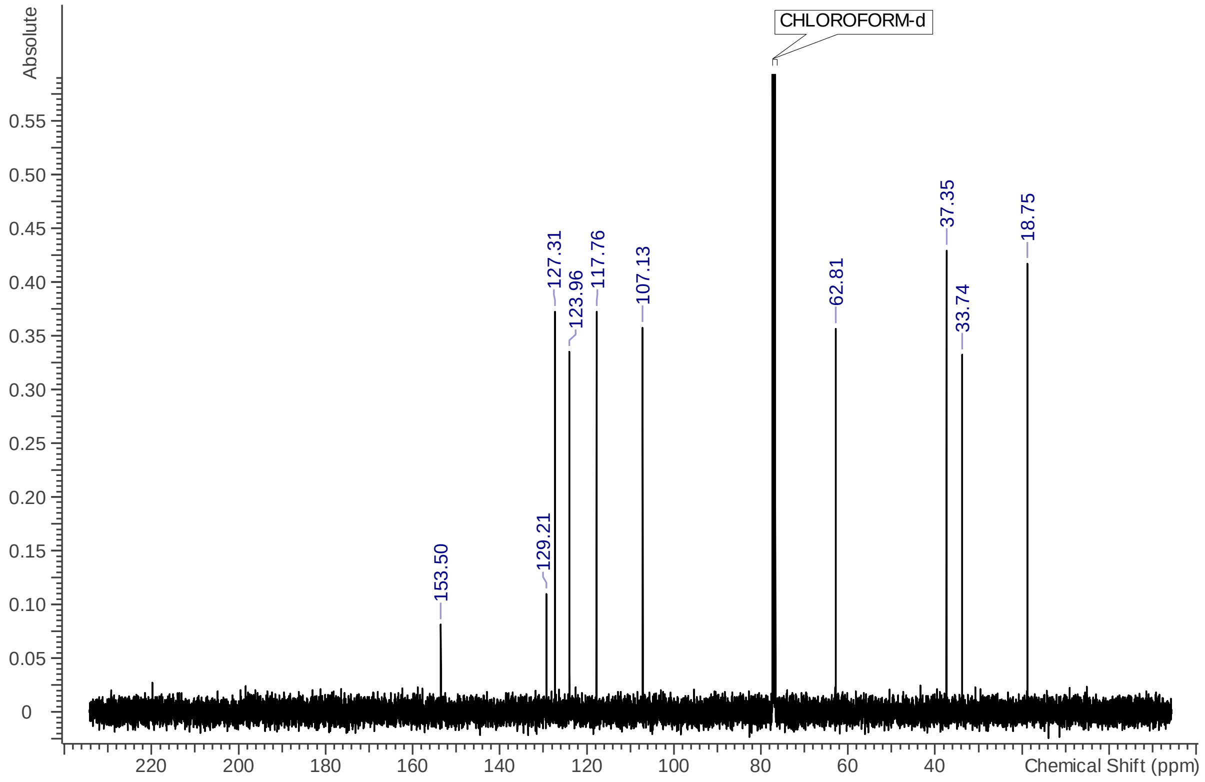

3. Optimization

Table S1 - optimization of acceptors

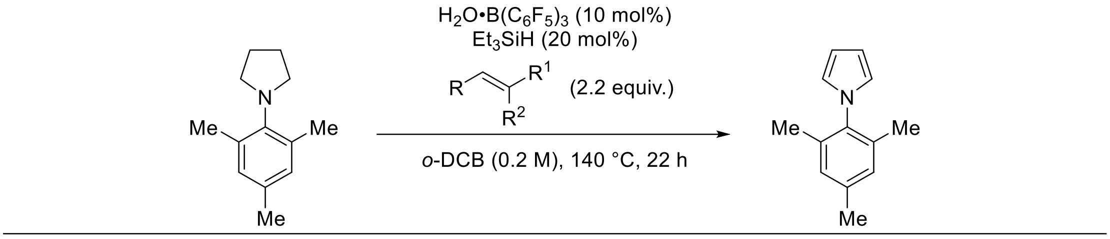

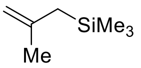

2a

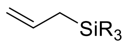

S5, R = Me
S7, R = ^{i}Pr
S8, R = Ph
S9, R = (OEt)

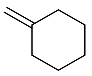

S10

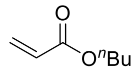

2c

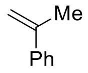

S11

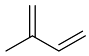

2b

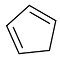

S12

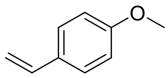

S13

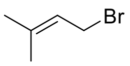

S14

Entry[a]	Acceptor	Yield (%)[b]
1	None	34
2	2a	79
3	S6	29
4	S7	41
5	S8	34
6	S9	0
7	S10	37
8	2c	47
9	S11	0
10	2b	72
11	S12	17
12	S13	0
13	S14	0

[a] Reactions performed using 0.2 mmol of *N*-Mesitylpyrrollidine, following general procedure 5. [b] Yields were determined by ^{1}H NMR analysis of the crude reaction mixture with nitromethane as the internal standard.

4. Experimental procedures for dehydrogenation

General procedure 4: Preparation of a solution of water-free $B(C_6F_5)_3$ [in situ preparation of water-free $B(C_6F_5)_3$]

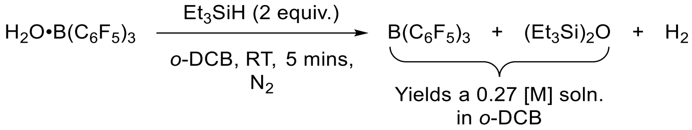

Preparation of a solution of $B(C_6F_5)_3$: To an oven-dried 5 mL J. Youngs ampoule equipped with stirrer bar was added $H_2O{\cdot}B(C_6F_5)_3$ (1 equiv.), which was suspended in *o*-DCB (3.75 mL/mmol) under a nitrogen atmosphere. Et_3SiH (2 equiv.) was added dropwise, leading to effervescence. The reaction mixture was stirred for 5 minutes. If required, gentle heating can be used to ensure all $B(C_6F_5)_3$ is in solution prior to usage in .

General procedure 5: Dehydrogenation of *N*-heterocycles with silane drying technique.

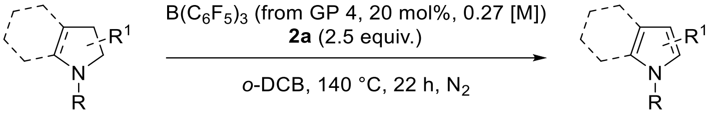

To an oven-dried 5 mL J. Youngs ampoule equipped with stirrer bar under a nitrogen atmosphere was added the solution of $B(C_6F_5)_3$ (0.15 mL, 20 mol%, prepared according to general procedure 4). Subsequently, a solution of pyrrolidine **1** (0.2 mmol) in *o*-DCB (0.6 mL, solution prepared in a sample vial under a nitrogen atmosphere) was added to the mixture, before methallyltrimethylsilane **2a** (0.5 mmol) was added. The ampoule was sealed, and stirred at 140 °C (oil bath) for 22 h. Upon completion, the reaction mixture was allowed to cool to ambient temperature, before the reaction was quenched with $NaHCO_3$ (aq., sat., 0.15 mL). The product was extracted with DCM (3 × 2 mL), before the organics were dried over an $MgSO_4$ plug. The combined organics were then concentrated in vacuo, before $MeNO_2$ (21.5 µL, 0.4 mmol) was added as an internal standard, and the spectroscopic yield calculated. Th crude material was purified via flash column chromatography on silica gel with a suitable eluent to give pure pyrroles.

General procedure 6: Preparation and isolation of water-free $B(C_6F_5)_3$.

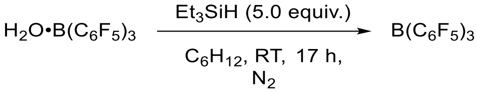

To an oven-dried Schlenk flask equipped with stirrer bar under a nitrogen atmosphere was added $H_2O{\cdot}B(C_6F_5)_3$ (1.0 equiv.), which was suspended in anhydrous hexane (3.5 mL/mmol). Et_3SiH (5.0 equiv.) was then added slowly, leading to effervescence. The reaction was allowed to stir at ambient temperature for 17 h, before the solvent was removed in vacuo at 60°C for 5 h. After this time, the resulting white solid was mobilised in pentane (roughly 3 mL/mmol) under an argon atmosphere, before being stored in the freezer overnight (−30 °C). The following day, the mixture was cooled to −30 °C (acetone/N_2 bath) and the solids were removed by cannula filtration at −30 °C. The solids were further washed with pentane (2 × 3 mL/mmol) at −30 °C. The resulting solid was dried in vacuo for a minimum of 30 minutes, before being stored in the glovebox.

General procedure 7: Dehydrogenation of pyrrolidines with isolated water-free $B(C_6F_5)_3$.

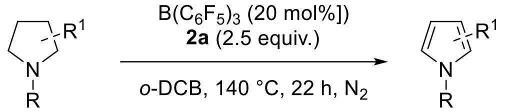

To an oven-dried 5 mL J. Youngs ampoule equipped with stirrer bar under an argon atmosphere (inside a glovebox) was added the $B(C_6F_5)_3$ (20 mol%, prepared according to General Procedure 6). This vial was removed from the glovebox, before being dissolved in *o*-DCB under a nitrogen atmosphere. A solution of pyrrolidine **1** (0.2 mmol) in *o*-DCB (0.6 mL, solution prepared in a sample vial under a nitrogen atmosphere) was added to the mixture, before methallyltrimethylsilane **2a** (stored over mol. sieves, 0.5 mmol) was added. The ampoule was sealed, and stirred at 140 °C (oil bath) for 22 h. Upon completion, the reaction mixture was allowed to cool to ambient temperature, before the reaction was quenched with $NaHCO_3$ (aq., sat., 0.15 mL). The product was extracted with DCM (3 × 2 mL), before the organics were dried over an $MgSO_4$ plug. The combined organics were then concentrated in vacuo, before $MeNO_2$ (21.5 µL, 0.4 mmol) was added as an internal standard, and the spectroscopic yield calculated.

1-Mesityl-1*H*-pyrrole 3a

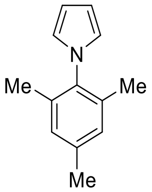

The title compound was prepared according to general procedure 5 using 1-mesitylpyrrolidine **1a** (37.8 mg, 0.20 mmol) with an NMR yield of 79%. Purification by flash column chromatography on silica gel (eluent = 20% DCM in pet. ether) gave the title compound **3a** as an orange oil (25 mg, 0.13 mmol, 67%).

Using 2a as the drying agent and acceptor to form 3a:

Following a modified general procedure 5, using 1-mesitylpyrrolidine **1a** (37.8 mg, 0.20 mmol), **2a** (102 µL, 2.9 equiv.), in the absence of Et_3SiH, **3a** was formed in 72% NMR yield.

Presumably, $H_2O{\cdot}B(C_6F_5)_3$ protonates **2a** which subsequently forms isobutylene and formally Et_3Si^+, which in turn reacts to form Et_3SiOH or $(Et_3Si)O$, as shown in scheme below.

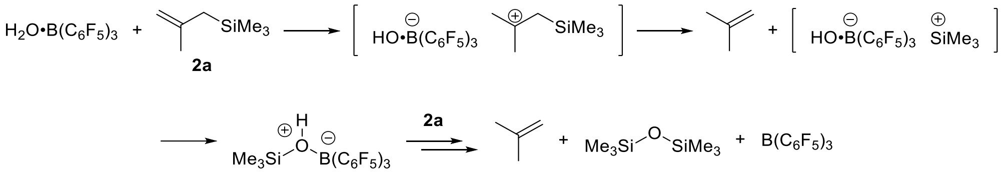

Scheme S1. Proposed pathway for the formation of water-free $B(C_6F_5)_3$ from $H_2O{\cdot}B(C_6F_5)_3$ and methylallyl trimethylsilane **2a** in the absence of Et_3SiH.

Preparative scale reaction to form 3a:

To a 25 mL J. Youngs ampoule equipped with stirrer bar was added $H_2O{\cdot}B(C_6F_5)_3$ (318 mg, 0.6 mmol) under a nitrogen atmosphere. *o*-DCB (4 mL) was added, before Et_3SiH (0.19 mL, 1.2 mmol) was added slowly with stirring, and the mixture allowed to stir for 10 minutes. In a separate vial, *N*-mesitylpyrrolidine **1a** (567.9 mg, 3 mmol) was dissolved in *o*-DCB (3 mL) under a nitrogen atmosphere, before being slowly added to the $B(C_6F_5)_3$ solution. Subsequently, methallyltrimethylsilane (1.32 mL, 7.5 mmol) was added, before the reaction was sealed and heated to 140 °C for 22 h. Upon completion, the reaction was quenched with $NaHCO_3$ (aq., sat., 10 mL) and extracted with DCM (3×20 mL). The combined organics were dried with $MgSO_4$ and concentrated in vacuo. Purification by flash column chromatography on silica gel (eluent = 5% DCM in pet. ether), gave the title compound **3a** as a colourless oil (375 mg, 2.03 mmol, 68%). $\mathbf{R_f}$ = 0.4 (eluent = 20% DCM in pet. ether); **^{1}H NMR** (400 MHz, $CDCl_3$) δ_H = 6.95 (s, ArH, 2H), 6.61 (2H, app. t, *J* = 2.1), 6.33 (2H, app. t, *J* = 2.1 Hz), 2.35 (3H, s), 2.02 (6H, s); **^{13}C NMR** (101 MHz, $CDCl_3$) δ_C = 137.7 (C), 137.4 (C) 135.9 (2×C), 128.6 (2×CH), 121.6 (2×CH), 108.4 (2×CH), 21.0 (CH_3), 17.2 (2×CH_3). Spectroscopic data in accordance with that stated in the literature.[9]

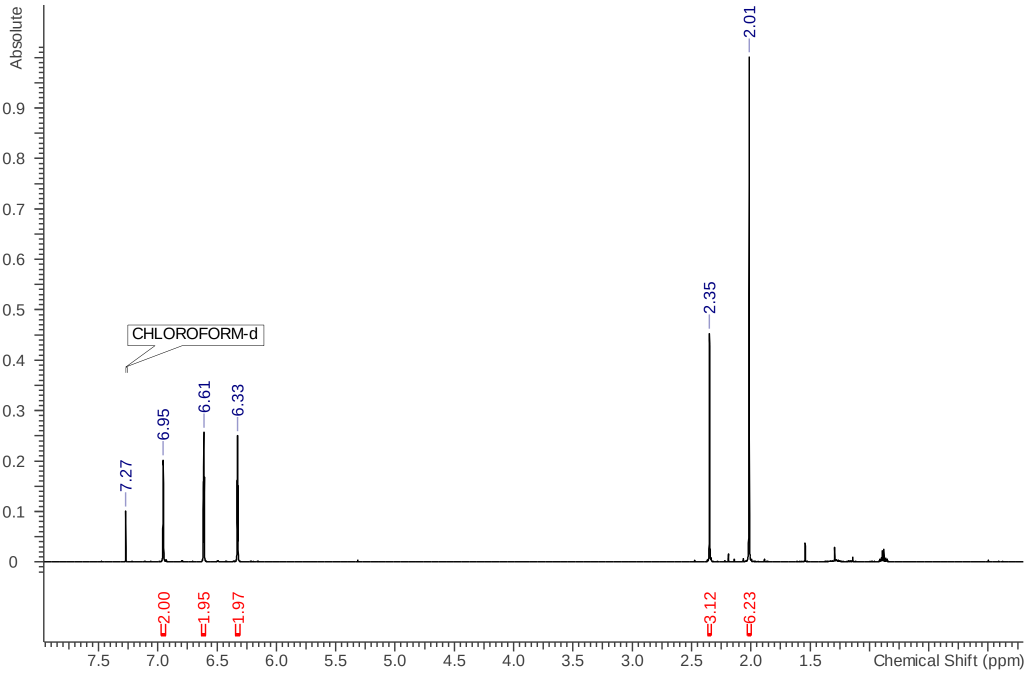

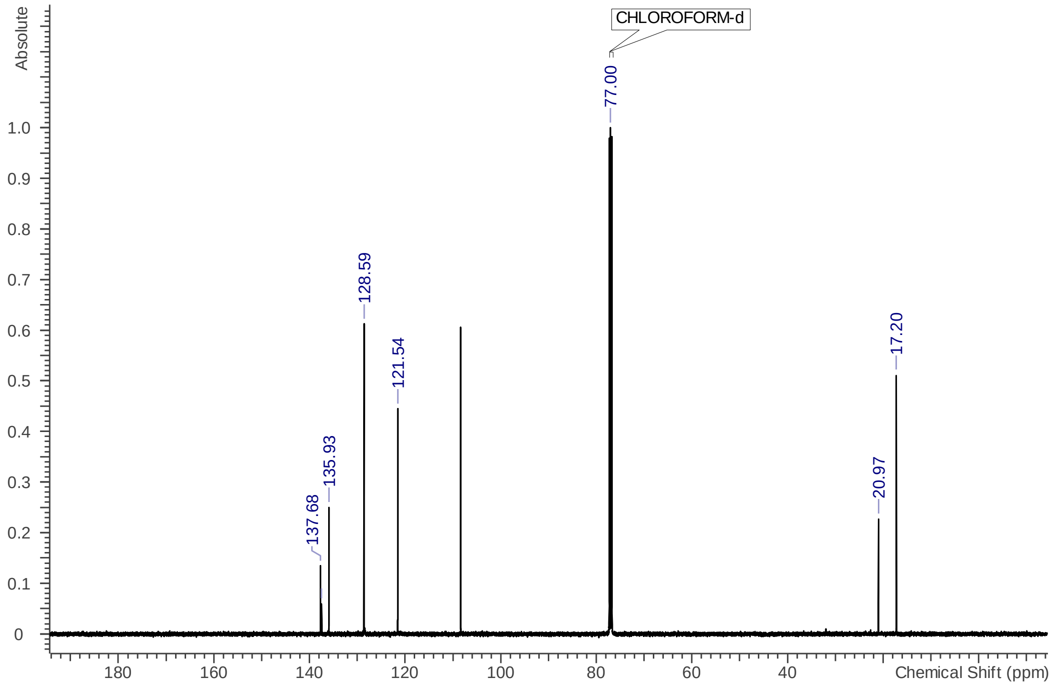

CHLOROFORM-d
77.00
128.59
121.54
135.93
137.68
20.97
17.20
Absolute
1.0
0.9
0.8
0.7
0.6
0.5
0.4
0.3
0.2
0.1
0
180
160
140
120
100
80
60
40
Chemical Shift (ppm)

2,5-Dimethyl-1-phenyl-1*H*-pyrrole 3b

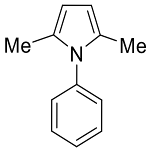

The title compound was prepared according to general procedure 5 using 2,5-dimethyl-1-phenylpyrrolidine **1b** (35.0 mg, 0.20 mmol) with an NMR yield of 62%. Purification by flash column chromatography on silica gel (eluent = 40% DCM in pet. ether) gave the title compound **3b** as a white solid (20.3 mg, 0.12 mmol, 59%). **R$_f$** = 0.4 (eluent = 40% DCM in pet. ether); **^{1}H NMR** (400 MHz, $CDCl_3$) δ_H = 7.51–7.38 (3H, m), 7.26–7.20 (2H, m), 5.92 (2H, s), 2.05 (6H, s); **^{13}C NMR** (101 MHz, $CDCl_3$) δ_C = 139.0 (C), 129.0 (2×CH), 128.8 (C), 128.2 (2×CH), 127.6 (CH), 105.6 (2×CH), 13.0 (CH_3); Spectroscopic data in accordance with that stated in the literature.[10]

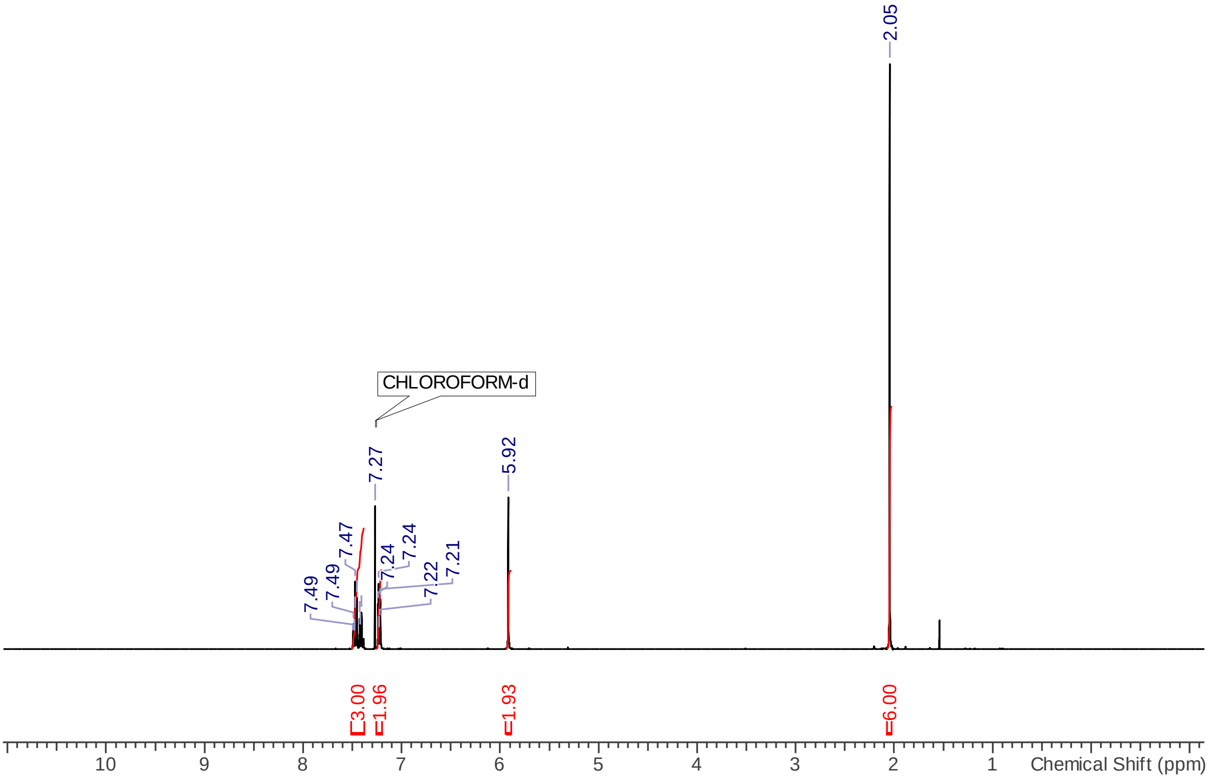

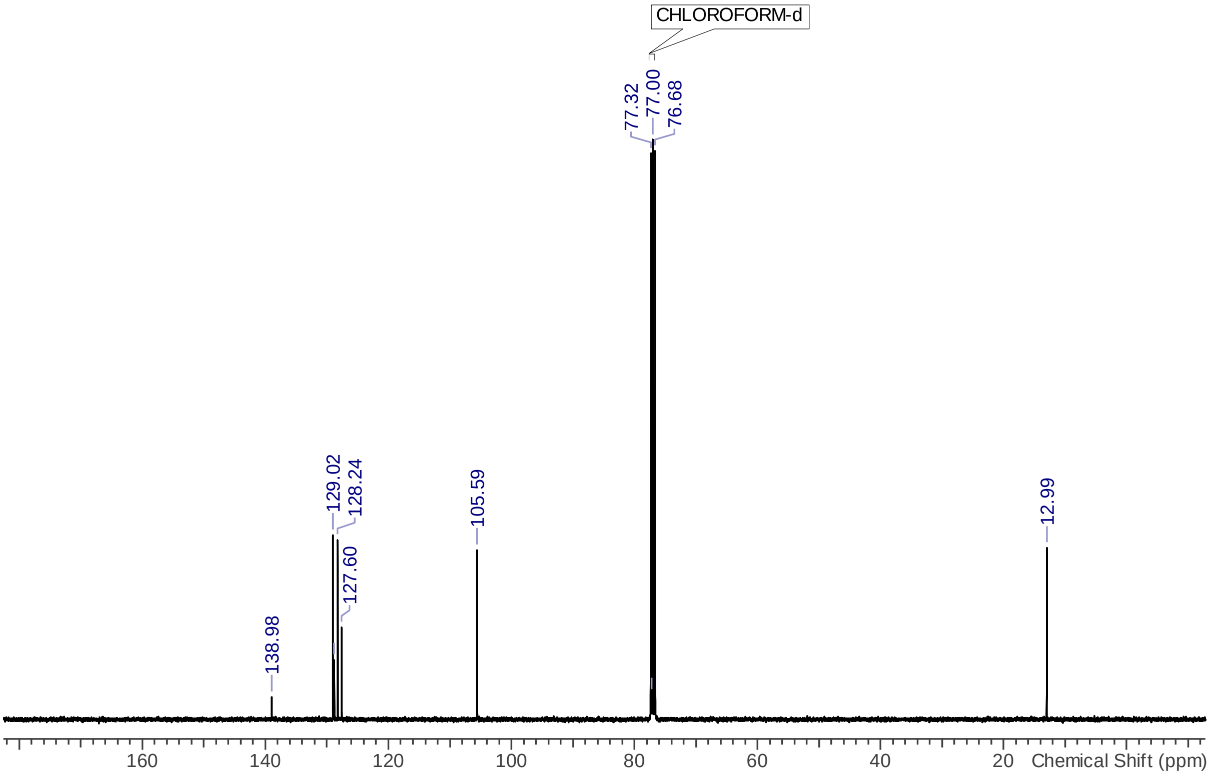

1-(2-Chlorophenyl)pyrrole 3c

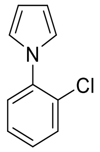

The title compound was prepared according to general procedure 5 using 1-(2-chlorophenyl)pyrrolidine **1c** (36.2 mg, 0.20 mmol) with an NMR yield of 60%. Purification by flash column chromatography on silica gel (eluent = 20% DCM in pet. ether) gave the title compound **3c** as an orange oil (20 mg, 0.11 mmol, 56%). **R$_f$** = 0.5 (eluent = 20% DCM in pet. ether); **^{1}H NMR** (400 MHz, $CDCl_3$) δ_H = 7.55–7.50 (1H, m), 7.37–7.28 (3H, m), 6.93 (2H, app. t, *J* = 2.1 Hz), 6.36 (2H, app. t, *J* = 2.1 Hz); **^{13}C NMR** (101 MHz, $CDCl_3$) δ_C = 138.8 (C), 130.7 (CH), 129.6 (C), 128.2 (CH), 127.8 (CH), 127.5 (CH), 122.2 (2×CH), 109.3 (2×CH). Spectroscopic data in accordance with that stated in the literature.[11]

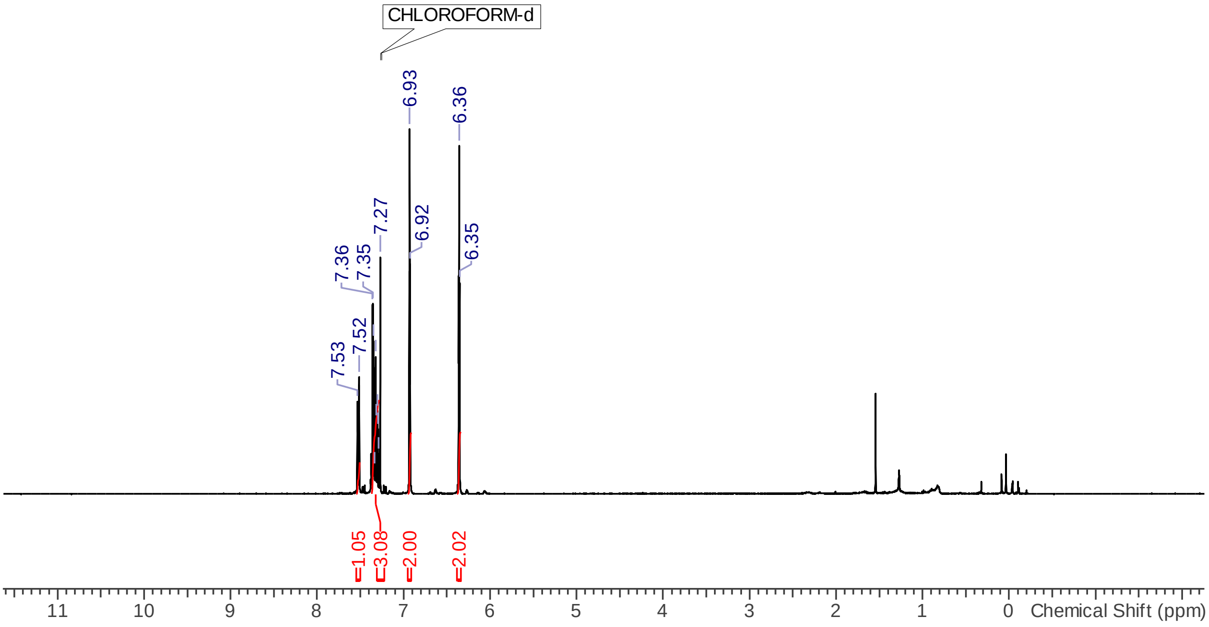

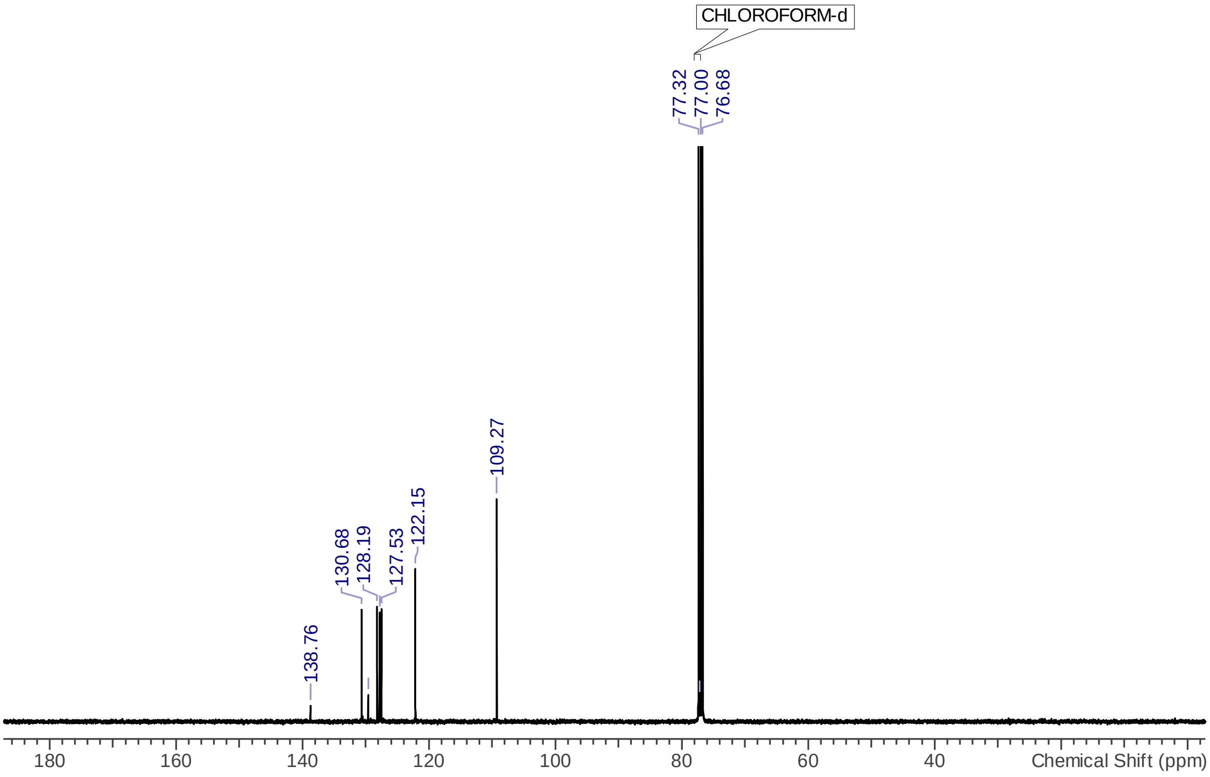

1-(2-Bromophenyl)-3-methyl-1H-pyrrole 3d

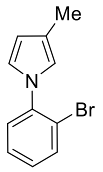

The title compound was prepared according to general procedure 5 using 1-(2-bromophenyl)-3-methylpyrrolidine **1d** (48.4 mg, 0.20 mmol) with an NMR yield of 50%. Purification by flash column chromatography on silica gel (eluent = 20% $CHCl_3$ in pet. ether) gave the title compound **3d** as a brown oil (14.1 mg, 0.06 mmol, 30%). **R_f** (25% $CHCl_3$/petroleum ether) 0.49. **^{1}H NMR** (400 MHz, $CDCl_3$) δ_H = 7.69 (1H, dd, *J* = 8.0, 1.3 Hz), 7.37 (1H, ddd, *J* = 7.9, 7.2, 1.3 Hz), 7.31 (1H, dd, *J* = 7.9, 1.8 Hz), 7.21 (1H, ddd, *J* = 8.0, 7.2, 1.8 Hz), 6.81 (1H, app. t, *J* = 2.5 Hz), 6.69–6.65 (1H, m), 6.20–6.16 (1H, m), 2.19 (3H, m); **^{13}C NMR** (101 MHz, $CDCl_3$) δ = 140.5 (C), 133.8 (CH), 128.3 (CH), 128.2 (CH), 128.1 (CH), 122.1 (CH), 120.0 (CH), 119.8 (C), 119.5 (C), 110.8 (CH), 11.9 (CH_3). **HRMS** (ESI): calculated for $[C_{11}H_{11}{}^{79}BrN]^+$ (M+H)$^+$ m/z: 236.0075; found 236.0074; calculated for $[C_{11}H_{11}{}^{81}BrN]^+$ (M+H)$^+$ m/z: 238.0054; found 238.0059.

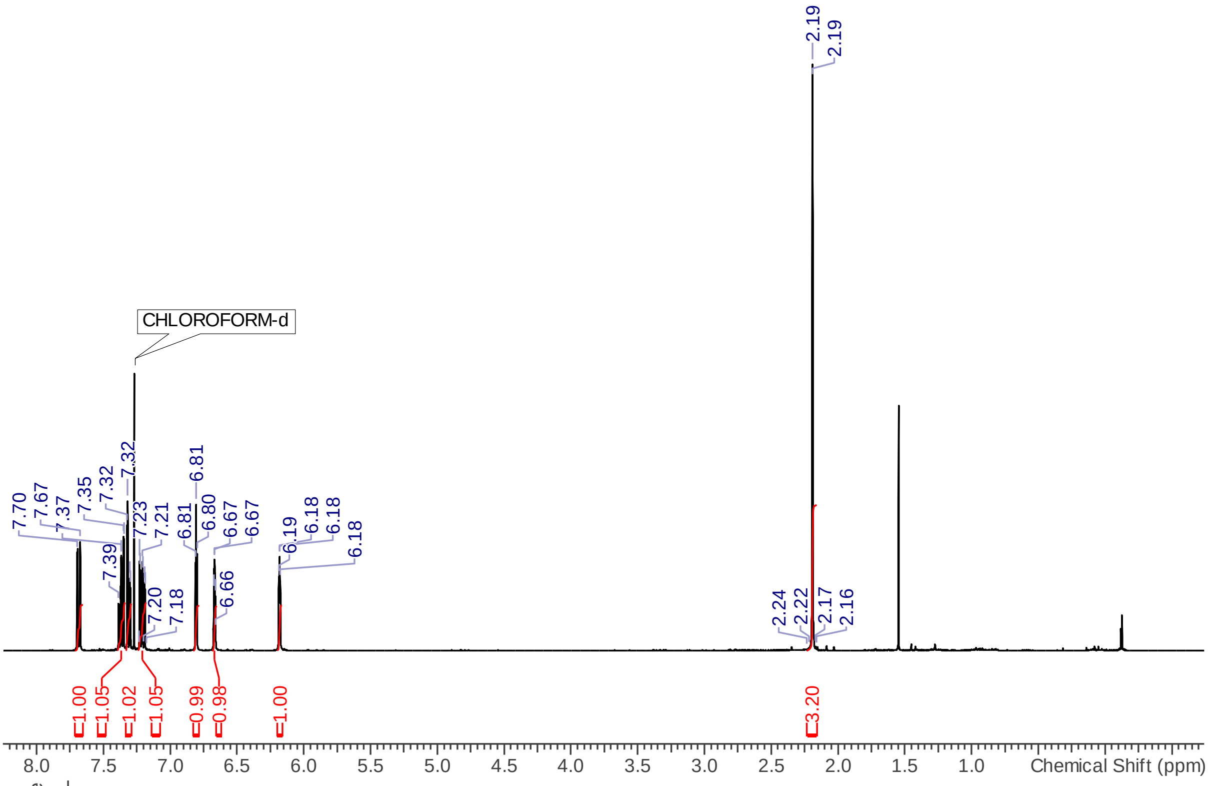

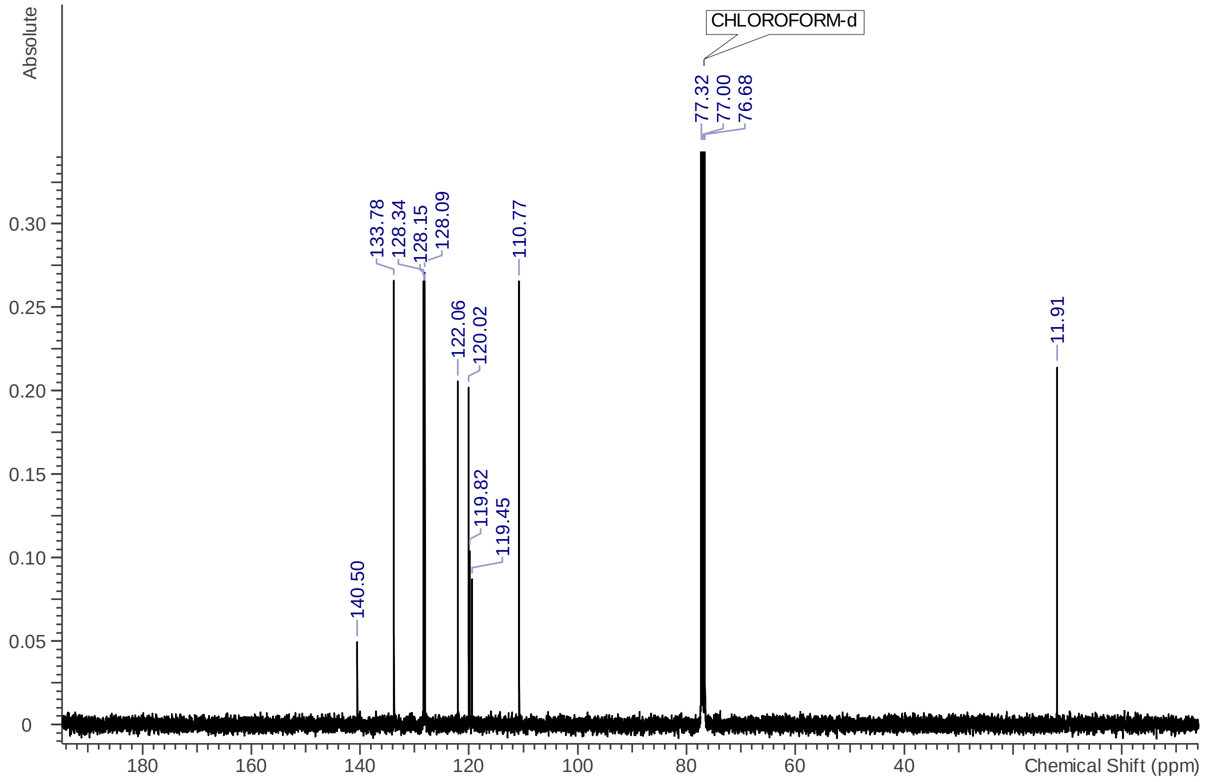

2-Methyl-1-phenyl-pyrrole 3e

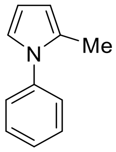

The title compound was prepared according to general procedure 5 using 2-methyl-1-phenyl-pyrrolidine **1e** (32.2 mg, 0.20 mmol) with an NMR yield of 60%. Purification by flash column chromatography on silica gel (eluent = 10% DCM, 0.3% Et_3N in pet. ether) gave the title compound **3e** as a colourless oil (12.1 mg, 0.08 mmol, 39%). **R$_f$** = 0.25 (eluent = 10% DCM in pet. ether); **^{1}H NMR** (400 MHz, $CDCl_3$) δ_H = 7.49–7.42 (2H, m), 7.40–7.30 (3H, m), 6.79 (1H, dd, *J* = 2.8, 1.9 Hz), 6.22 (1H, dd, *J* = 3.3, 2.8 Hz), 6.06 (1H, ddq, *J* = 3.3, 1.9, 0.9 Hz), 2.23 (3H, d, *J* = 0.9 Hz); **^{13}C NMR** (101 MHz, $CDCl_3$) δ_C = 140.4 (C), 129.0 (C, 2×CH), 126.8 (CH), 125.7 (2×CH), 121.4 (CH), 108.1 (CH), 108.0 (CH), 12.9 (CH_3). Spectroscopic data in accordance with that stated in the literature.[12]

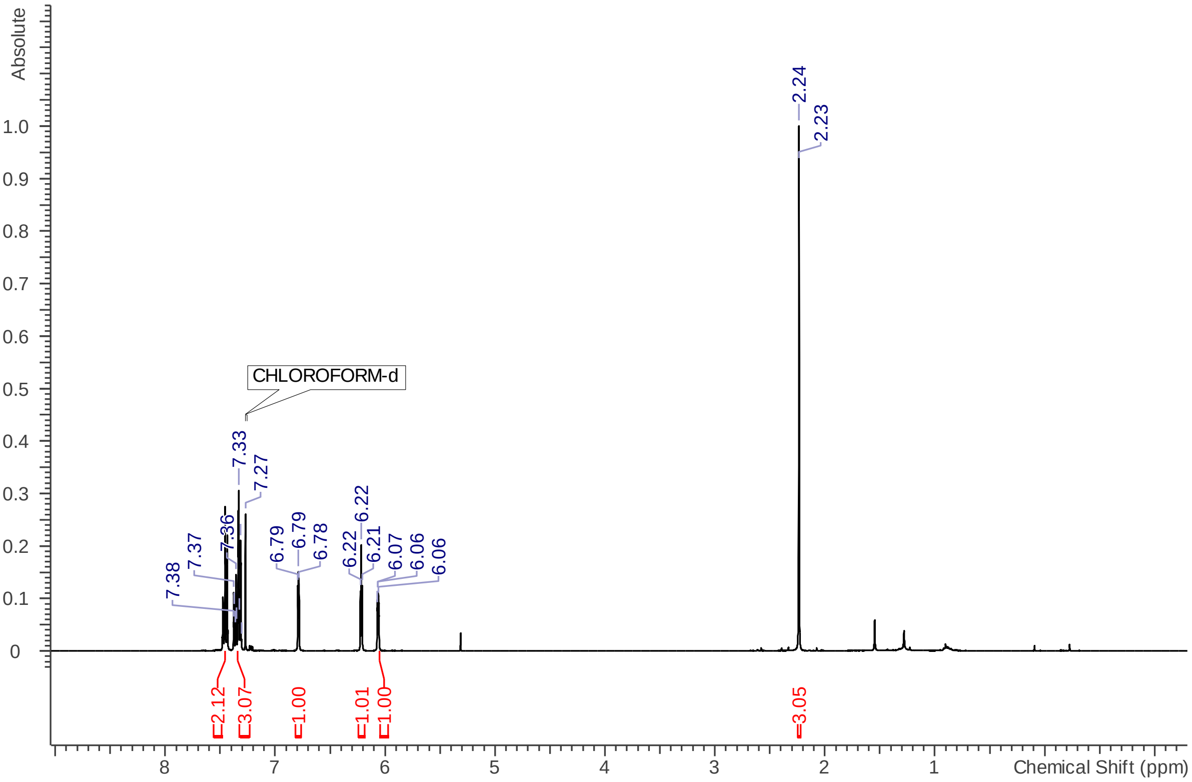

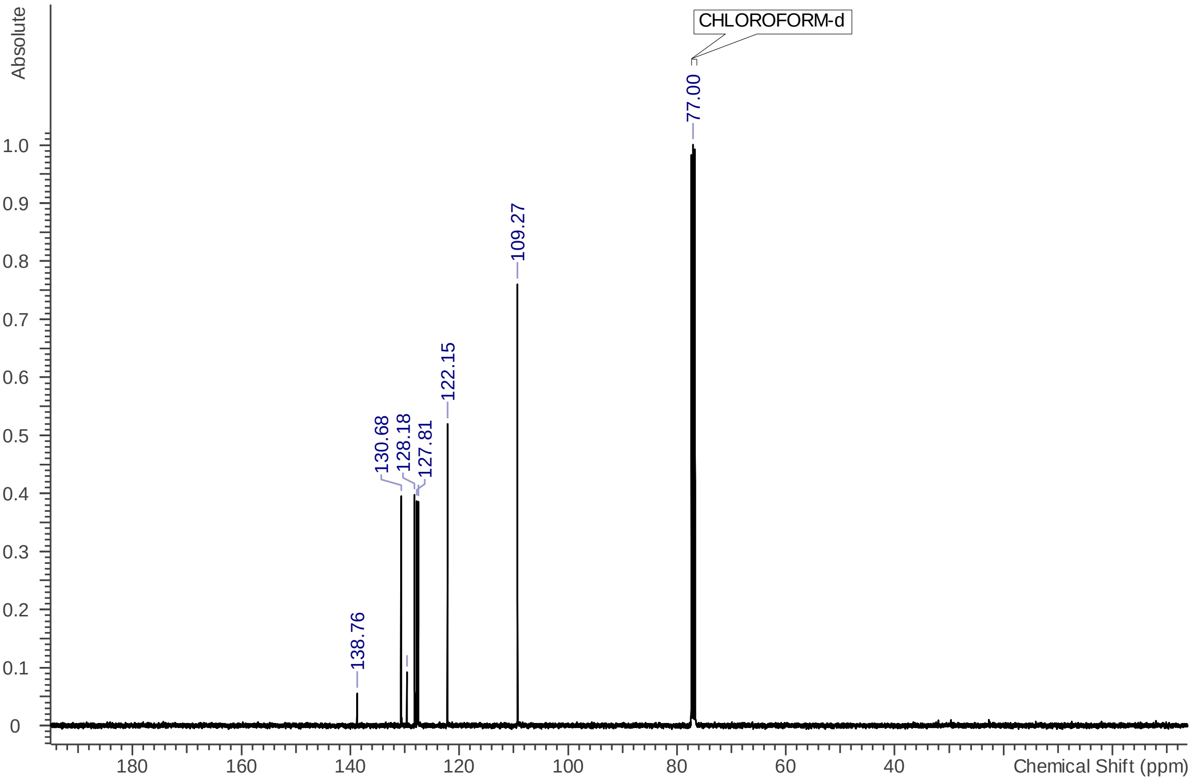

1-(2-Chlorophenyl)-2-methylpyrrole 3f

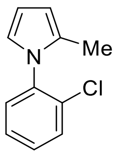

The title compound was prepared according to general procedure 5 using 1-(2-chlorophenyl)-2-methylpyrrolidine **1f** (39.0 mg, 0.20 mmol) with an NMR yield of 70%. Purification by automated flash column chromatography on silica gel (eluent = 5–25% DCM in pet. ether) gave the title compound **3f** as a yellow oil (5.8 mg, 0.03 mmol, 15%). **R_f** (10% DCM/petroleum ether) 0.31. **^{1}H NMR** (400 MHz, $CDCl_3$) δ_H = 7.56–7.49 (1H, m), 7.41–7.32 (3H, m), 6.65 (1H, dd, *J* = 2.8, 1.8 Hz), 6.24 (1H, dd, *J* = 3.2, 2.8 Hz), 6.06 (1H, ddq, *J* = 3.2, 1.8, 0.7 Hz), 2.06 (3H, d, *J* = 0.7 Hz); **^{13}C NMR** (101 MHz, $CDCl_3$) δ = 138.0 (C), 132.9 (C), 130.2 (CH), 130.1 (C), 129.8 (CH), 129.3 (CH), 127.3 (CH), 121.3 (CH), 108.1 (CH), 107.0 (CH), 12.1 (CH_3). Spectroscopic data in accordance with that stated in the literature.[13]

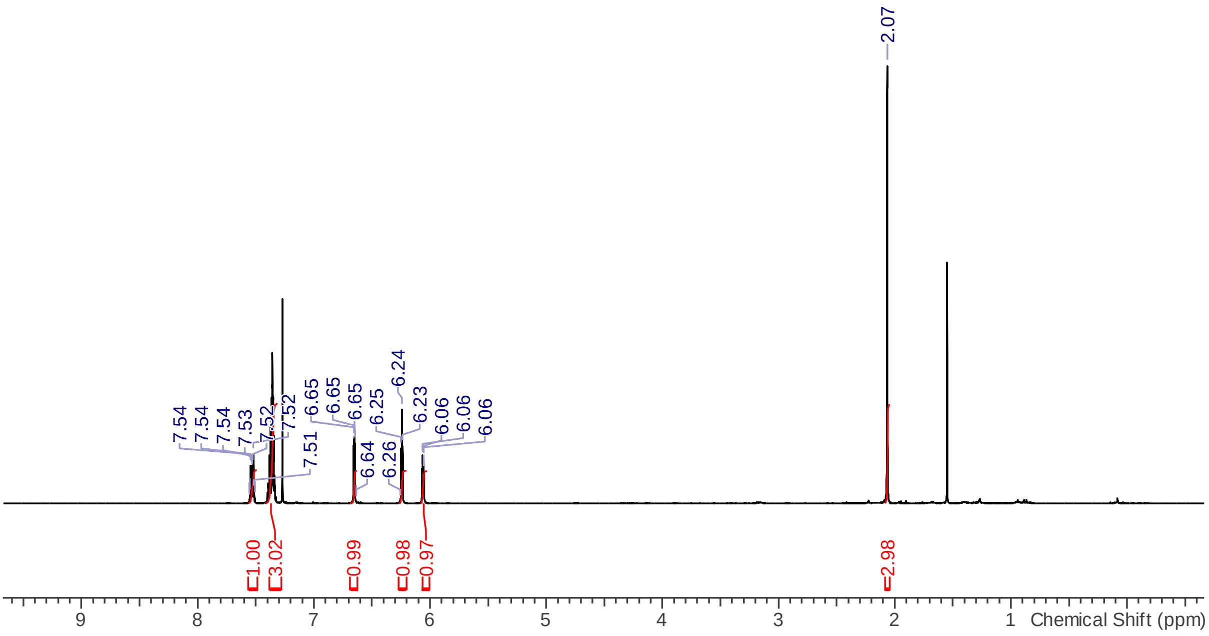

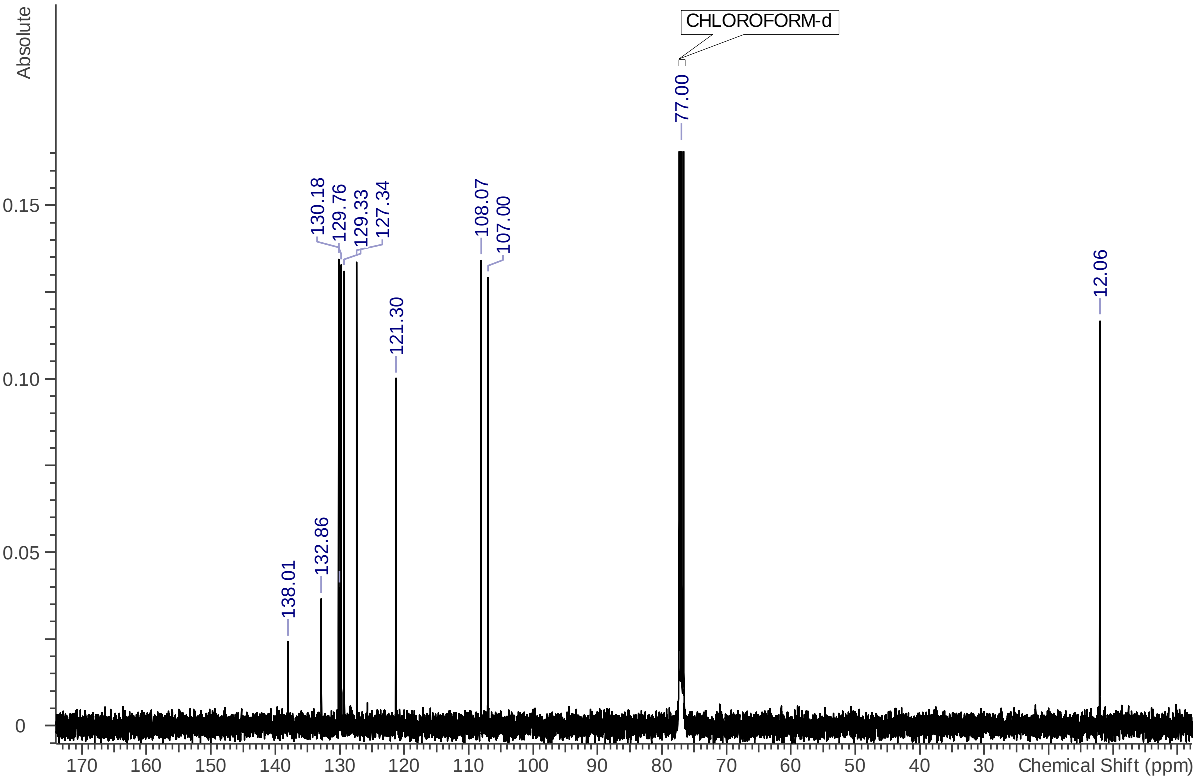

1-(2-Bromophenyl)-2-methyl-1*H*-pyrrole 3g

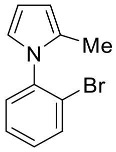

The title compound was prepared according to general procedure 5 using 1-(2-bromophenyl)-2-methylpyrrolidine **1g** (47.8 mg, 0.20 mmol) with an NMR yield of 80%. Purification by flash column chromatography on silica gel (eluent = 20% DCM in pet. ether) gave the title compound **3g** as a colourless oil (35 mg, 0.12 mmol, 61%). **R$_f$** = 0.2 (eluent = 20% DCM in pet. ether); **^{1}H NMR** (400 MHz, $CDCl_3$) δ_H = 7.75–7.66 (1H, m), 7.45–7.39 (1H, m), 7.38–7.28 (2H, m), 6.68–6.60 (1H, m), 6.26–6.24 (1H, m), 6.09–6.03 (1H, m), 2.07 (3H, br. s); **^{13}C NMR** (101 MHz, $CDCl_3$) δ_C = 139.6 (C), 133.3 (CH), 129.8 (C, CH), 129.6 (CH), 128.0 (CH), 123.2 (C), 121.1 (CH), 108.0 (CH), 107.0 (CH), 12.1 (CH_3); Spectroscopic data in accordance with that stated in the literature.[13]

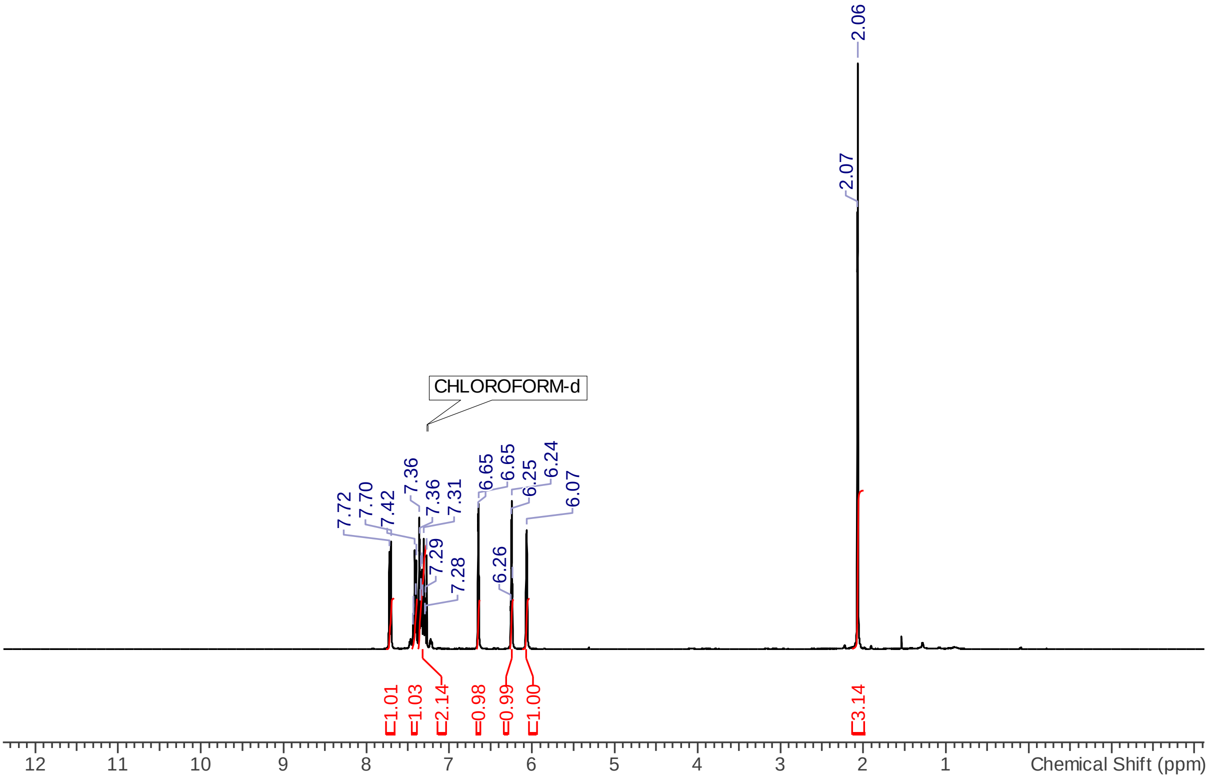

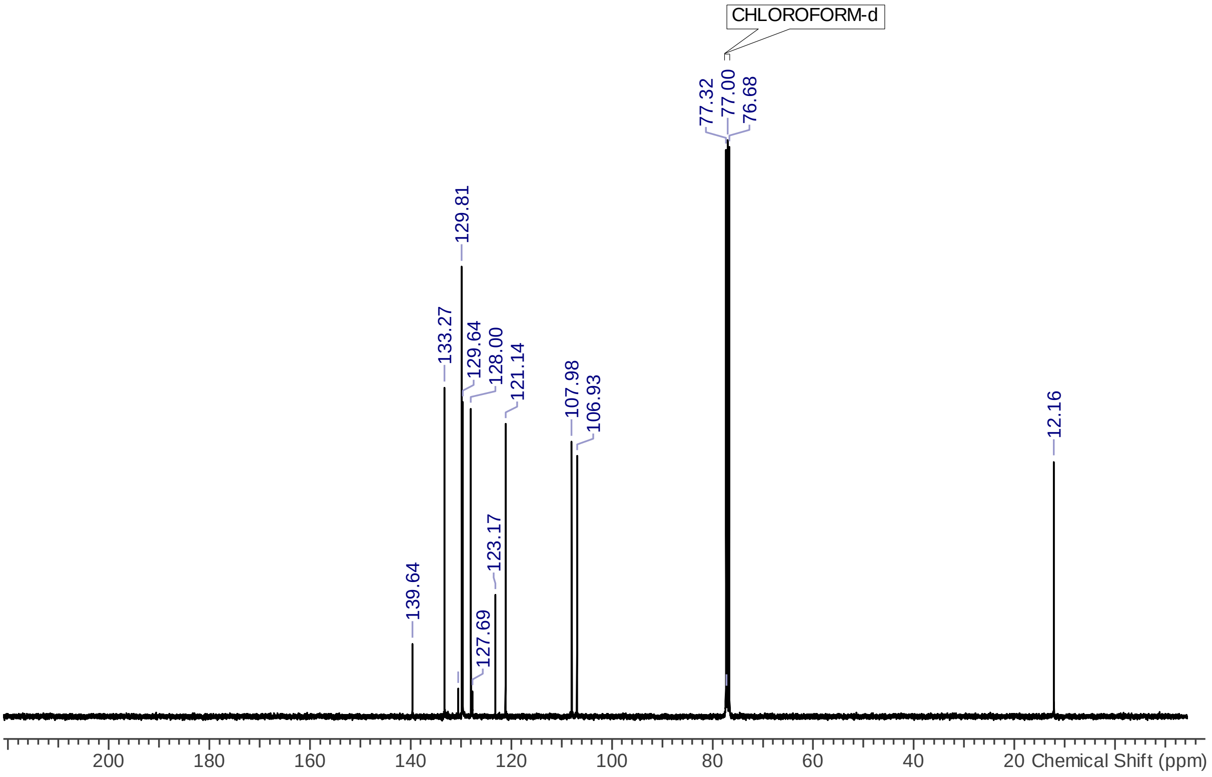

1-(2-Methoxyphenyl)-2-methyl-1*H*-pyrrole 3h

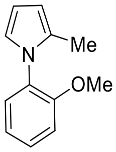

The title compound was prepared according to general procedure 5 using 1-(2-methoxyphenyl)-2-methylpyrrolidine **1h** (38.1 mg, 0.20 mmol) with an NMR yield of 40%. Purification by flash column chromatography on silica gel (eluent = 40% DCM in pet. ether) gave the title compound **3h** as an orange oil (14.2 mg, 0.08 mmol, 38%). **R**$_f$ = 0.4 (eluent = 40% DCM in pet. ether); ^{1}**H NMR** (400 MHz, $CDCl_3$) δ_H = 7.37 (1H, app. td, *J* = 7.9, 1.7 Hz), 7.24 (1H, dd, *J* = 8.1, 1.7 Hz), 7.06–6.98 (2H, m), 6.67 (1H, dd, *J* = 2.7, 1.9 Hz), 6.22 (1H, dd, *J* = 3.4, 2.7 Hz), 6.05-6.01 (1H, m), 3.80 (3H, s), 2.08 (3H, br. d, *J* = 0.6 Hz); ^{13}**C NMR** (101 MHz, $CDCl_3$) δ_C = 154.8 (C), 130.2 (C), 129.0 (C, 2×CH), 121.7 (CH), 120.5 (CH), 111.9 (CH), 107.6 (CH), 106.6 (CH), 55.7 (CH_3), 12.1 (CH_3); **HRMS** (ESI$^+$): calculated for $[C_{12}H_{14}NO]^+$ $(M+H)^+$ m/z: 188.1075; found 188.1076.

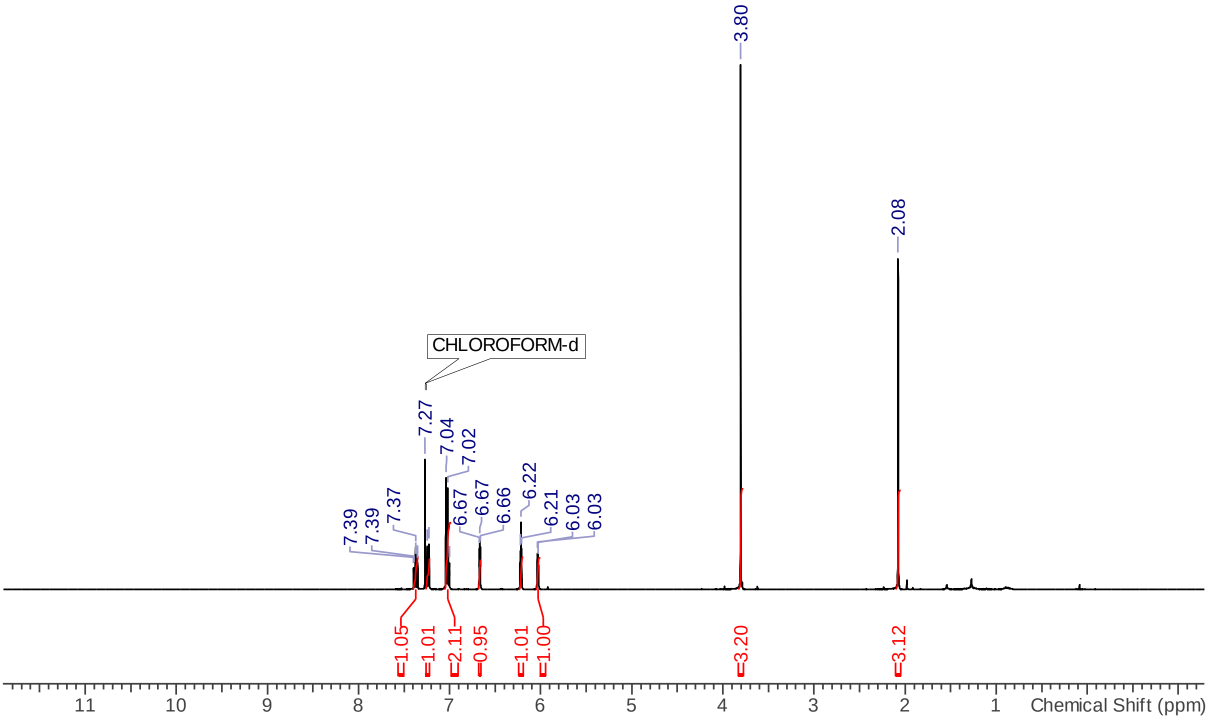

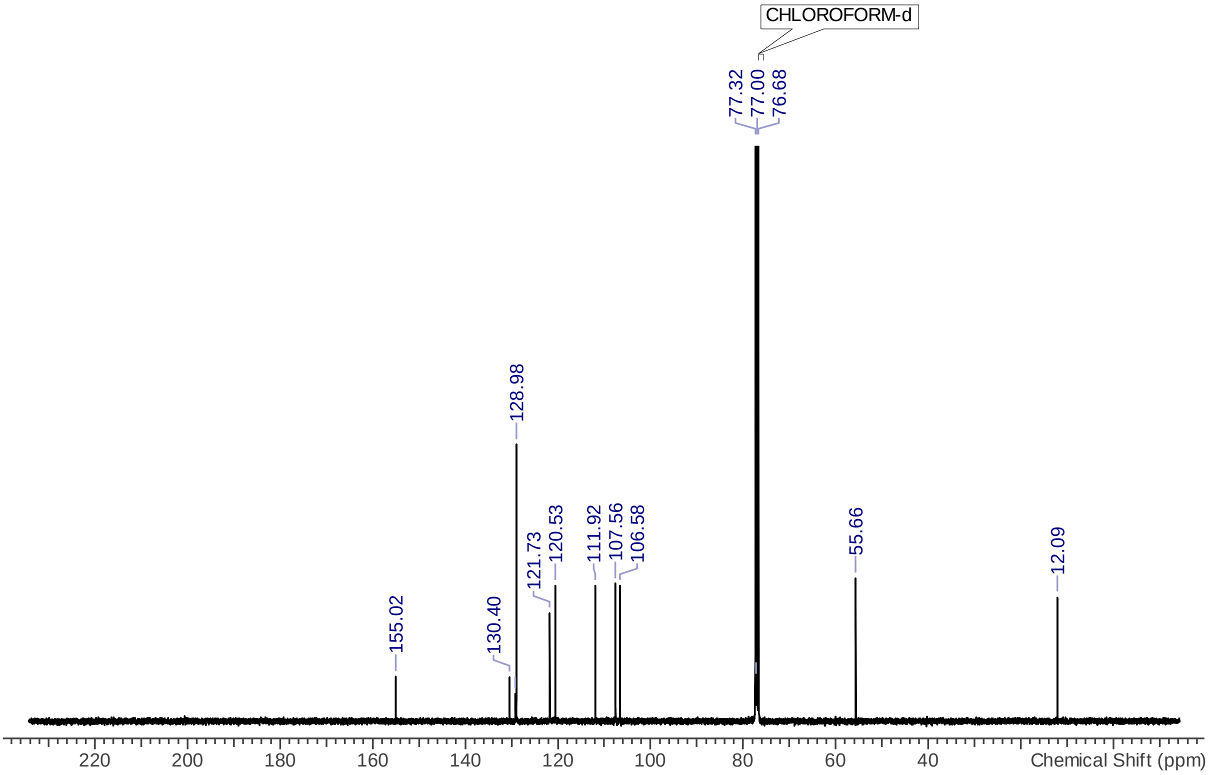

2-Methyl-1-(2-phenoxyphenyl)-1*H*-pyrrole 3i

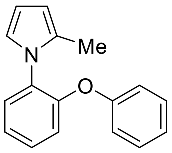

The title compound was prepared according to general procedure 5 (reaction done using DCE, heated to 85 °C) using 2-methyl-1-(2-phenoxyphenyl)pyrrolidine **1i** (50.6 mg, 0.20 mmol) with an NMR yield of 88%. Purification by flash column chromatography on silica gel (eluent = 20% DCM in pet. ether) gave the title compound **3i** as a light brown oil (42.5 mg, 0.17 mmol, 84%). $\mathbf{R_f}$ = 0.3 (eluent = 20% DCM in pet. ether); **^{1}H NMR** (400 MHz, $CDCl_3$) δ_H = 7.37–7.25 (4H, m), 7.22–7.16 (1H, m), 7.11–7.01 (2H, m), 6.96-6.89 (2H, m), 6.70–6.66 (1H, m), 6.15–6.11 (1H, m), 5.97–5.94 (1H, m), 2.17 (3H, br. s); **^{13}C NMR** (101 MHz, $CDCl_3$) δ_C = 156.6 (C), 152.7 (C), 131.6 (C), 130.1 (C), 129.6 (2×CH), 129.5 (CH), 128.8 (CH), 123.5 (2×CH), 121.8 (CH), 119.7 (CH), 118.7 (2×CH), 107.8 (CH), 107.0 (CH), 12.3 (CH_3); **HRMS** (ESI$^+$): calculated for $[C_{17}H_{16}NO]^+$ (M+H)$^+$ m/z: 250.1232; found 250.1233.

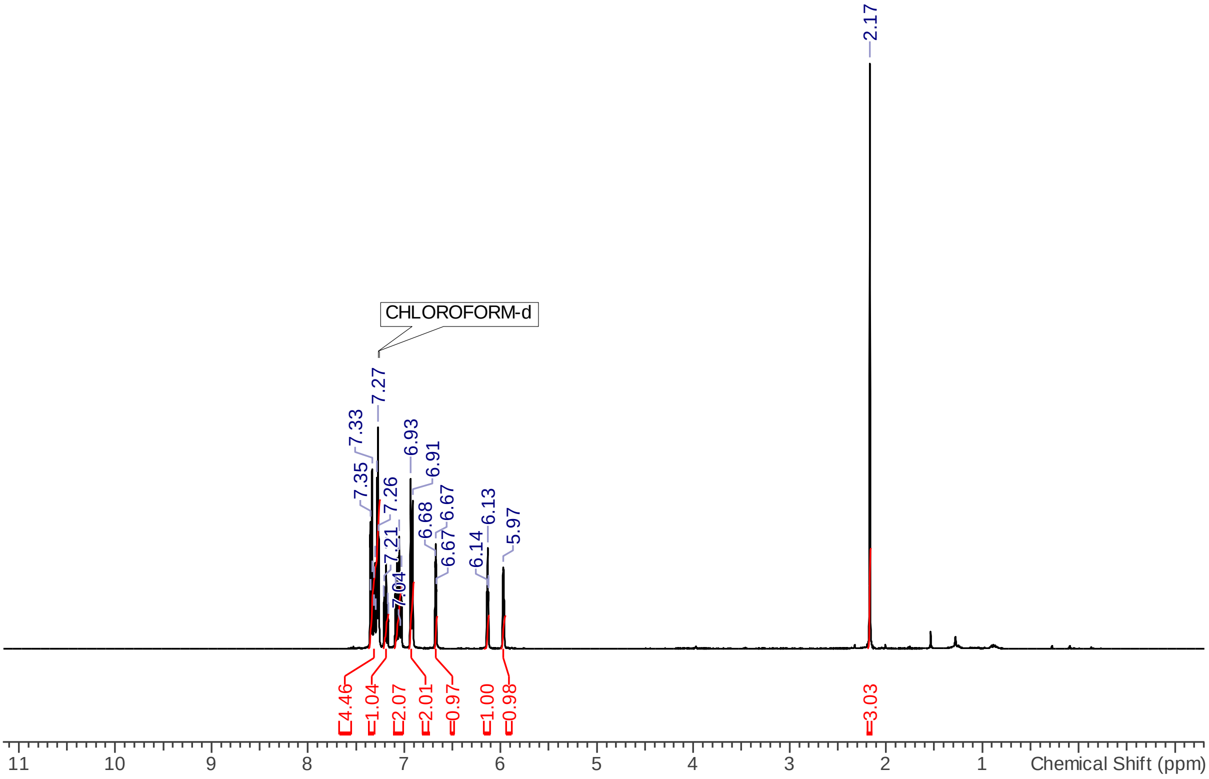

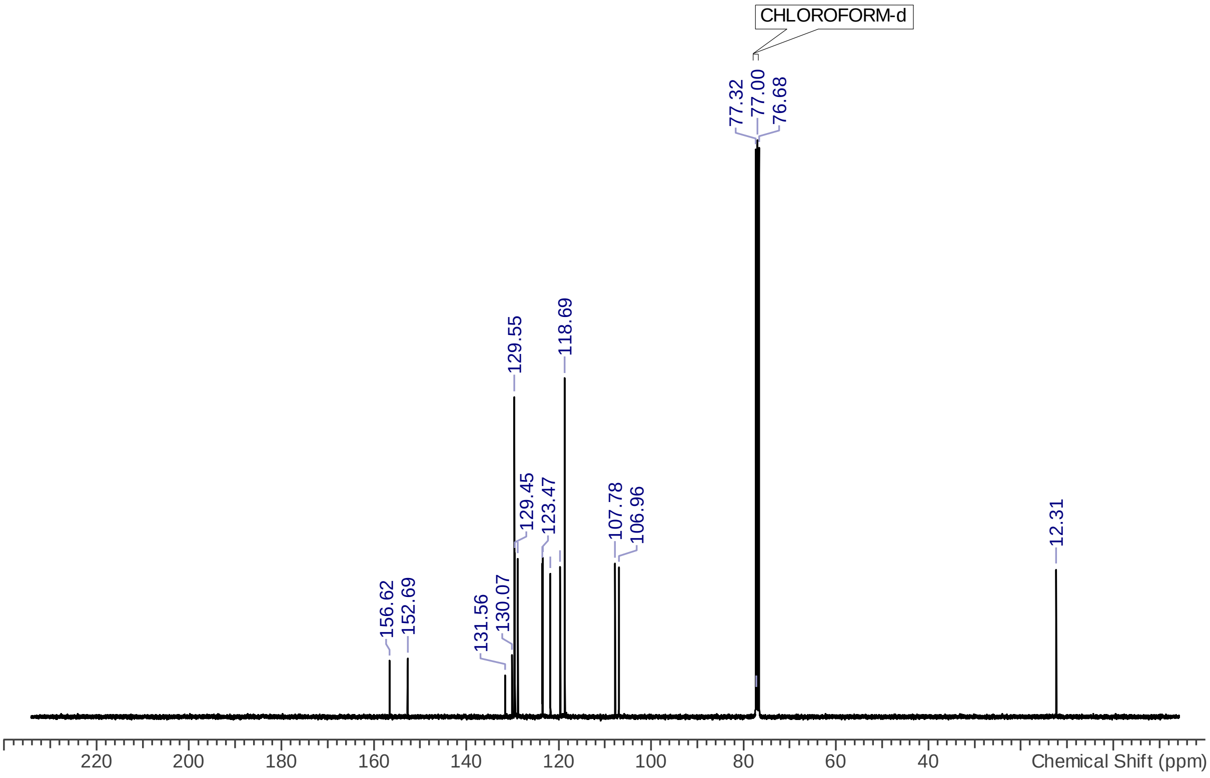

1-(4-Chlorophenyl)-2-methyl-*1H*-pyrrole 3j

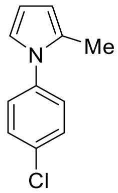

The title compound was prepared according to general procedure 5 using 1-(4-chlorophenyl)-2-methylpyrrolidine **1j** (39 mg, 0.20 mmol) with an NMR yield of 64%. Purification by automated flash column chromatography on silica gel (eluent = 10–25% $CHCl_3$ in pet. ether) gave the title compound **3j** as a brown oil (3.5 mg, 0.02 mmol, 9%). $\mathbf{R_f}$ = 0.22 (eluent = 14% $CHCl_3$ in pet. ether); **^{1}H NMR** (400 MHz, $CDCl_3$) δ_H = 7.45–7.38 (2H, m), 7.27–7.22 (2H, m), 6.74 (1H, dd, *J* = 2.9, 1.8 Hz), 6.21 (1H, dd, *J* = 3.1, 2.9 Hz), 6.05 (1H, ddq, *J* = 3.1, 1.8, 0.8 Hz), 2.21 (3H, d, *J* = 0.8 Hz); **^{13}C NMR** (101 MHz, $CDCl_3$) δ_C = 138.9 (C), 132.6 (C), 129.2 (2×CH), 129.0 (C), 126.9 (2×CH), 121.3 (CH), 108.5 (CH), 108.4 (CH), 12.9 (CH_3); Spectroscopic data in accordance with that stated in the literature.[14]

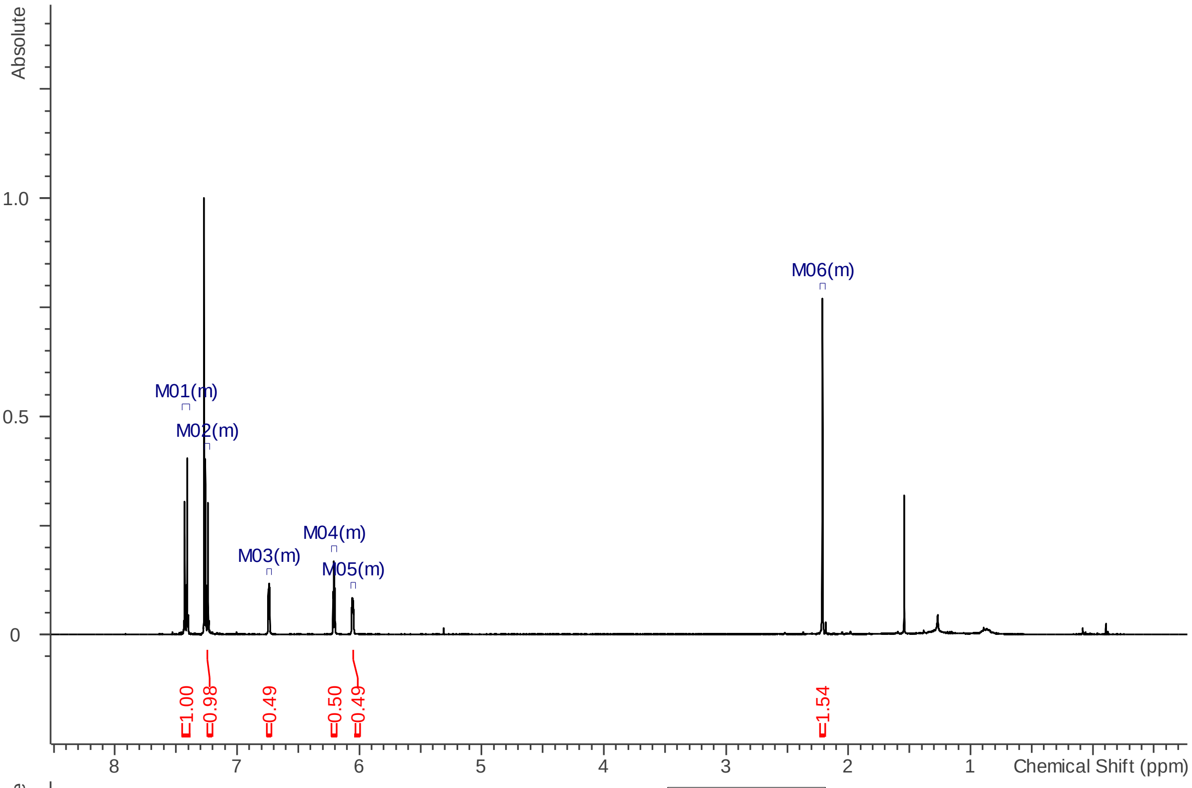

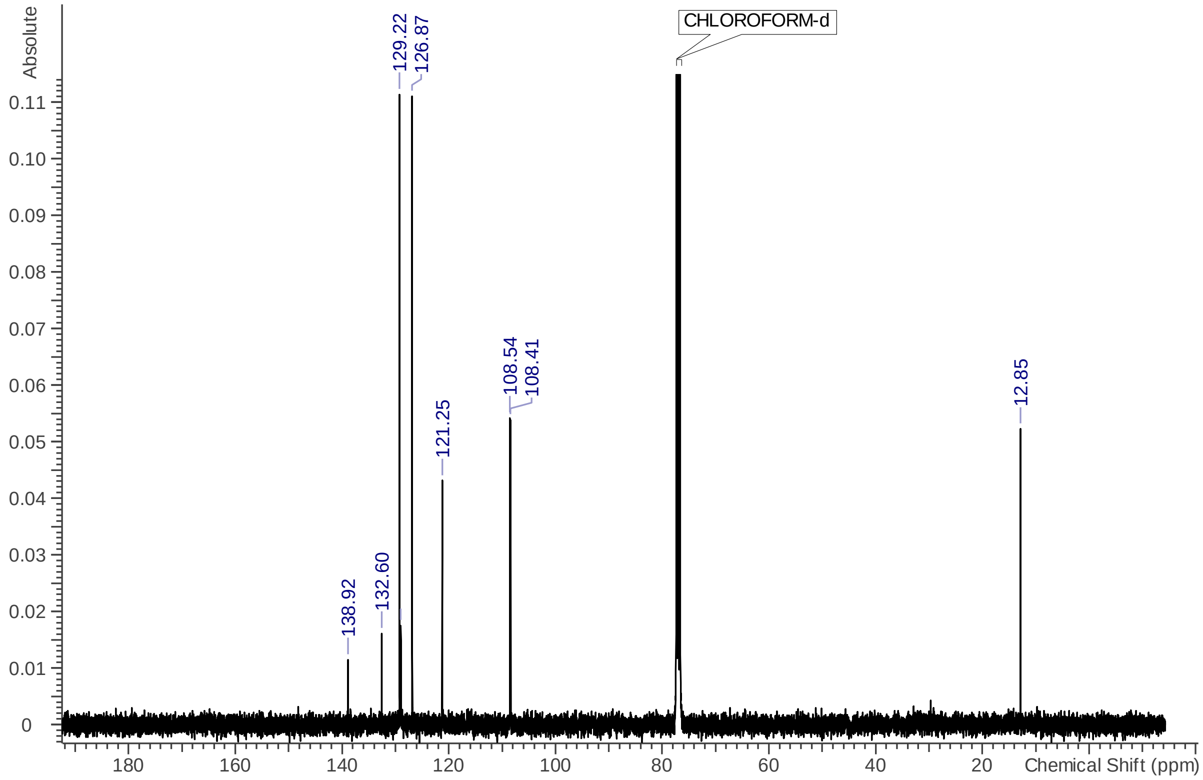

1-(4-Bromophenyl)-2-methyl-1*H*-pyrrole 3k

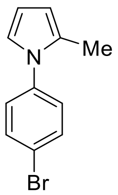

The title compound was prepared according to general procedure 5 using 1-(4-bromophenyl)-2-methylpyrrolidine **1k** (47.8 mg, 0.20 mmol) with an NMR yield of 68%. Purification by flash column chromatography on silica gel (eluent = 10% DCM in pet. ether) gave the title compound **3k** as a yellow oil (22 mg, 0.10 mmol, 49%). **R$_f$** = 0.29 (eluent = 10% DCM in pet. ether); **^{1}H NMR** (400 MHz, $CDCl_3$) δ_H = 7.61–7.55 (2H, m), 7.23–7.17 (2H, m), 6.74 (1H, dd, *J* = 2.8, 1.9 Hz), 6.21 (1H, dd, *J* = 3.3, 2.8 Hz), 6.06 (1H, ddq, *J* = 3.3, 1.9, 0.6 Hz), 2.22 (3H, d, *J* = 0.6 Hz); **^{13}C NMR** (101 MHz, $CDCl_3$) δ_C = 139.4 (C), 132.2 (2×CH), 128.9 (C), 127.2 (2×CH), 121.2 (CH), 120.5 (C), 108.6 (CH), 108.5 (CH), 12.9 (CH_3); **HRMS** (ESI$^+$): calculated for $[C_{11}H_{10}N^{79}Br]^+$ (M+H)$^+$ m/z: 236.0075; found 236.0075; calculated for $[C_{11}H_{10}N^{81}Br]^+$ (M+H)$^+$ m/z: 238.0054; found 238.0056.

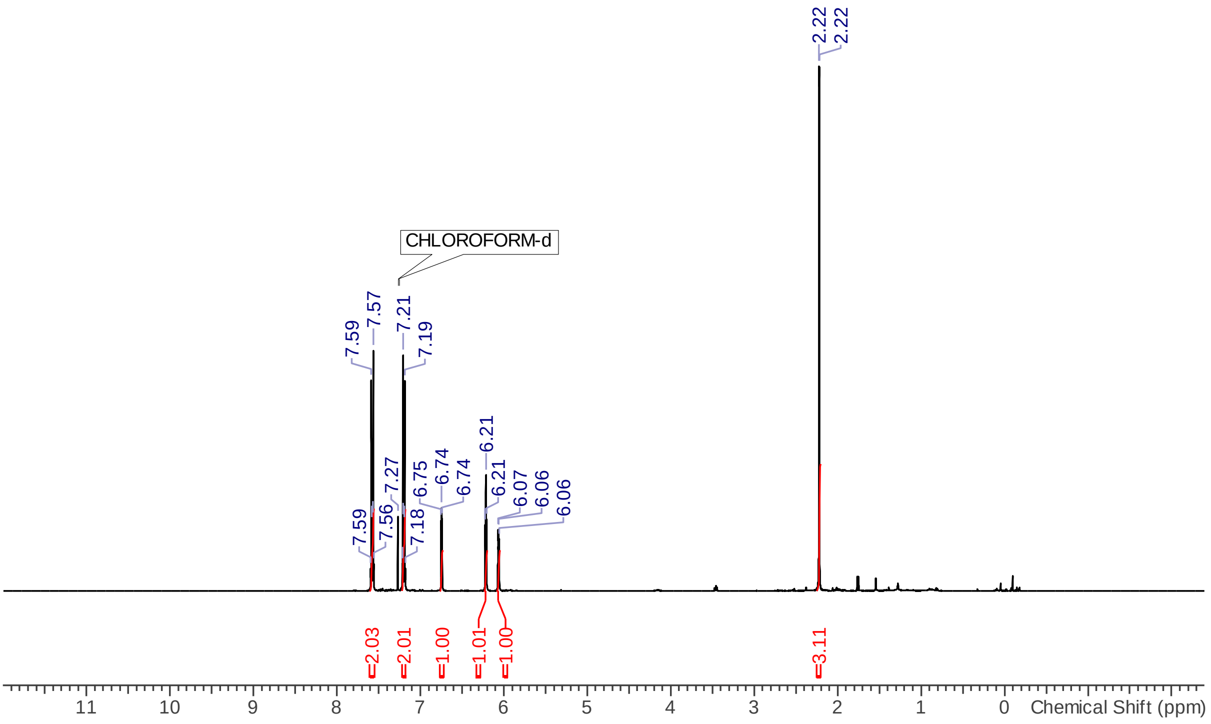

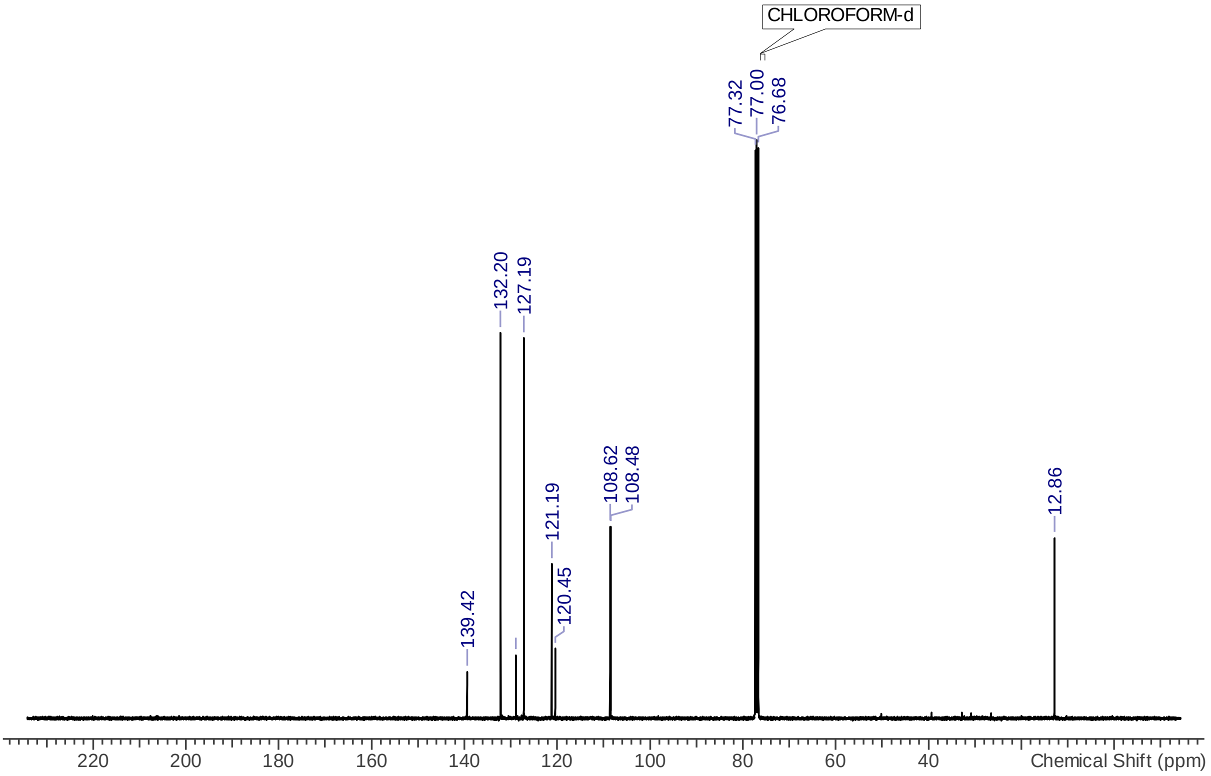

2-Methyl-1-(*p*-tolyl)-1*H*-pyrrole 3l

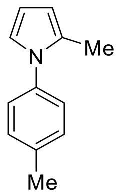

The title compound was prepared according to general procedure 5 using 1-(4-methylphenyl)-2-methylpyrrolidine **1l** (35.1 mg, 0.20 mmol) with an NMR yield of 56%. Purification by flash column chromatography on silica gel (eluent = 30% DCM in pet. ether) gave the title compound **3l** as a colourless oil (16 mg, 0.09 mmol, 45%). **R$_f$** = 0.3 (eluent = 30% DCM in pet. ether); **^{1}H NMR** (400 MHz, $CDCl_3$) δ_H = 7.26–7.22 (2H, m), 7.22–7.17 (2H, m), 6.75 (1H, dd, *J* = 2.7, 1.9 Hz), 6.19 (1H, dd, *J* = 3.3, 2.7 Hz), 6.04 (1H, ddq, *J* = 3.3, 1.9, 0.7 Hz), 2.42 (3H, s), 2.21 (3H, d, *J* = 0.7 Hz); **^{13}C NMR** (101 MHz, $CDCl_3$) δ_C = 137.9 (C), 136.7 (C), 129.6 (2×CH), 129.1 (C), 125.6 (2×CH), 121.4 (CH), 107.8 (CH), 107.7 (CH), 21.0 (CH_3), 12.8 (CH_3); **HRMS** (ESI$^+$): calculated for $[C_{12}H_{14}N]^+$ $(M+H)^+$ m/z: 172.1126; found 172.1132.

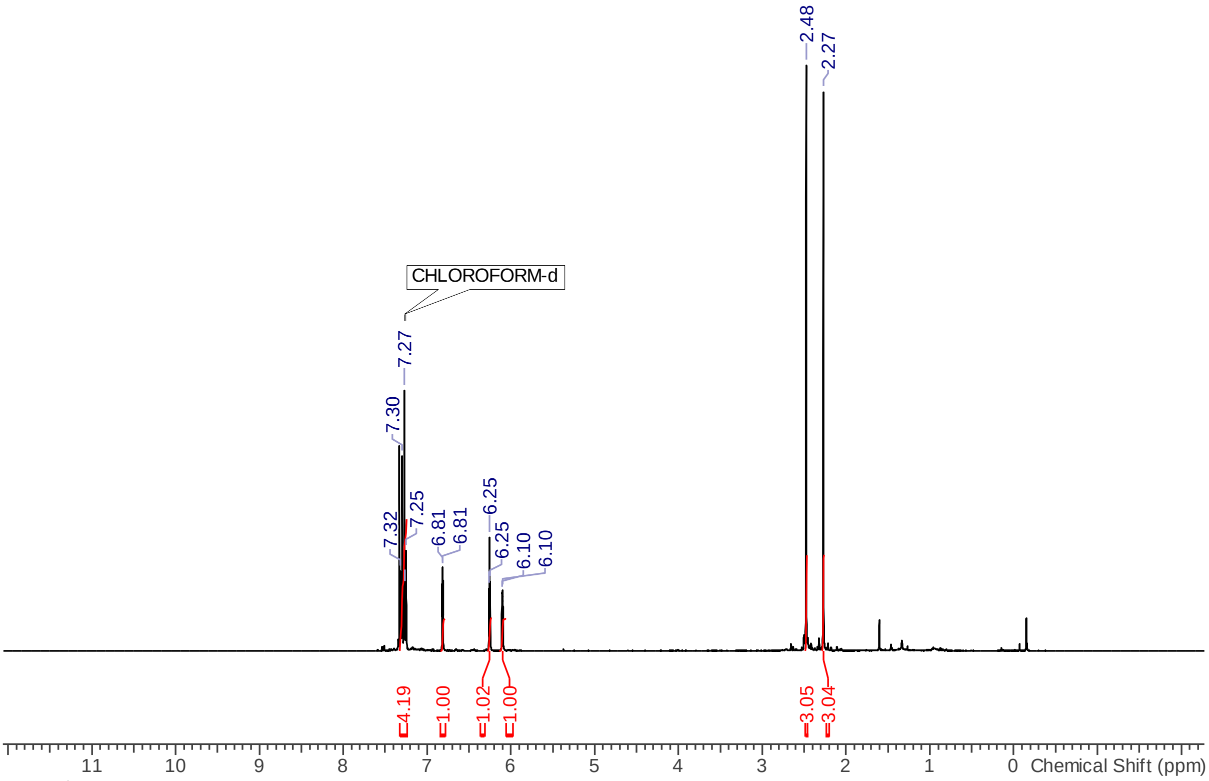

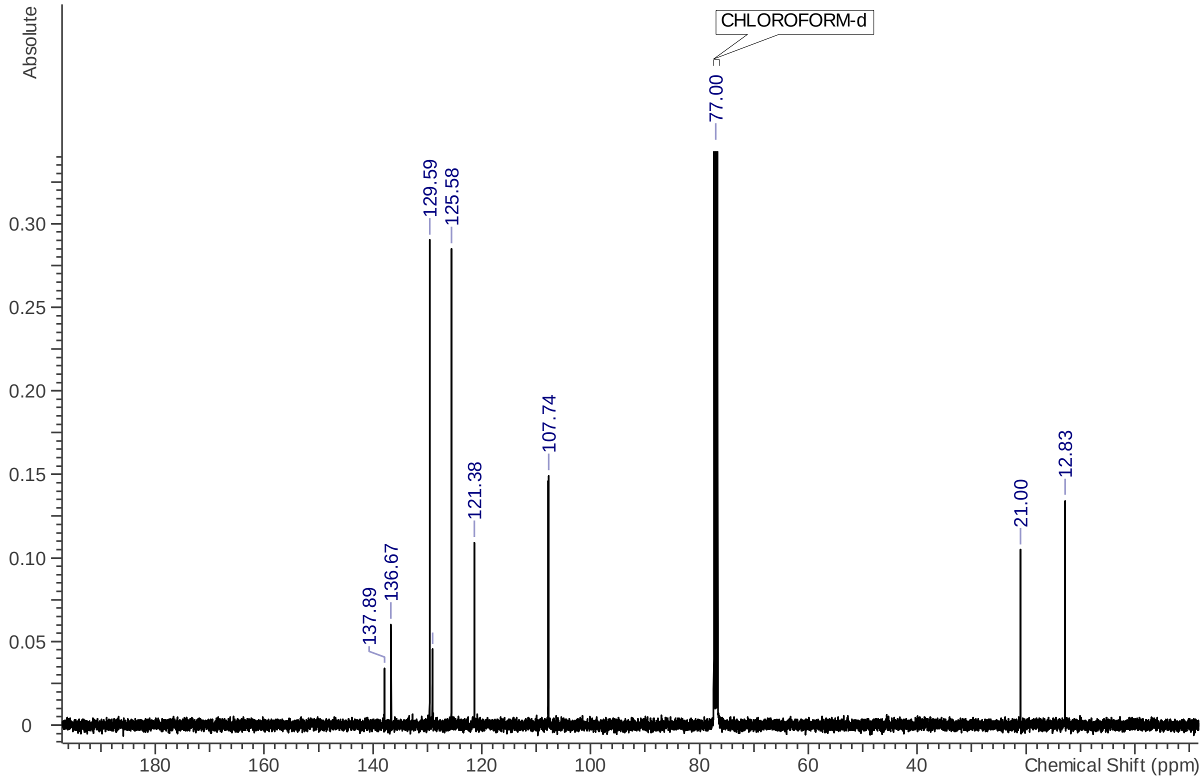

1-(4-Methoxyphenyl)-2-methyl-1*H*-pyrrole 3m

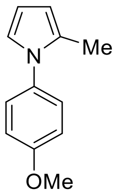

The title compound was prepared according to general procedure 5 using 1-(4-methoxyphenyl)-2-methylpyrrolidine **1m** (38.2 mg, 0.20 mmol) with an NMR yield of 64%. Purification by flash column chromatography on silica gel (eluent = 20% DCM in pet. ether) gave the title compound **3m** as a colourless oil (23.1 mg, 0.12 mmol, 62%). **R**$_f$ = 0.2 (eluent = 20% DCM in pet. ether); **^{1}H NMR** (400 MHz, $CDCl_3$) δ_H = 7.26–7.19 (2H, m), 6.99-6.93 (2H, m), 6.73 (1H, dd, *J* = 2.8, 2.0 Hz), 6.18 (1H, dd, *J* = 3.3, 2.8 Hz), 6.05–6.00 (1H, m), 3.86 (3H, s), 2.18 (3H, br. s); **^{13}C NMR** (101 MHz, $CDCl_3$) δ_C = 158.4 (C), 133.5 (C), 129.3 (C), 127.1 (2×CH), 121.5 (CH), 114.1 (2×CH), 107.6 (CH), 107.5 (CH), 55.5 (CH_3), 12.7 (CH_3); **HRMS** (ESI$^+$): calculated for $[C_{12}H_{14}NO]^+$ $(M+H)^+$ m/z: 188.1075; found 188.1075.

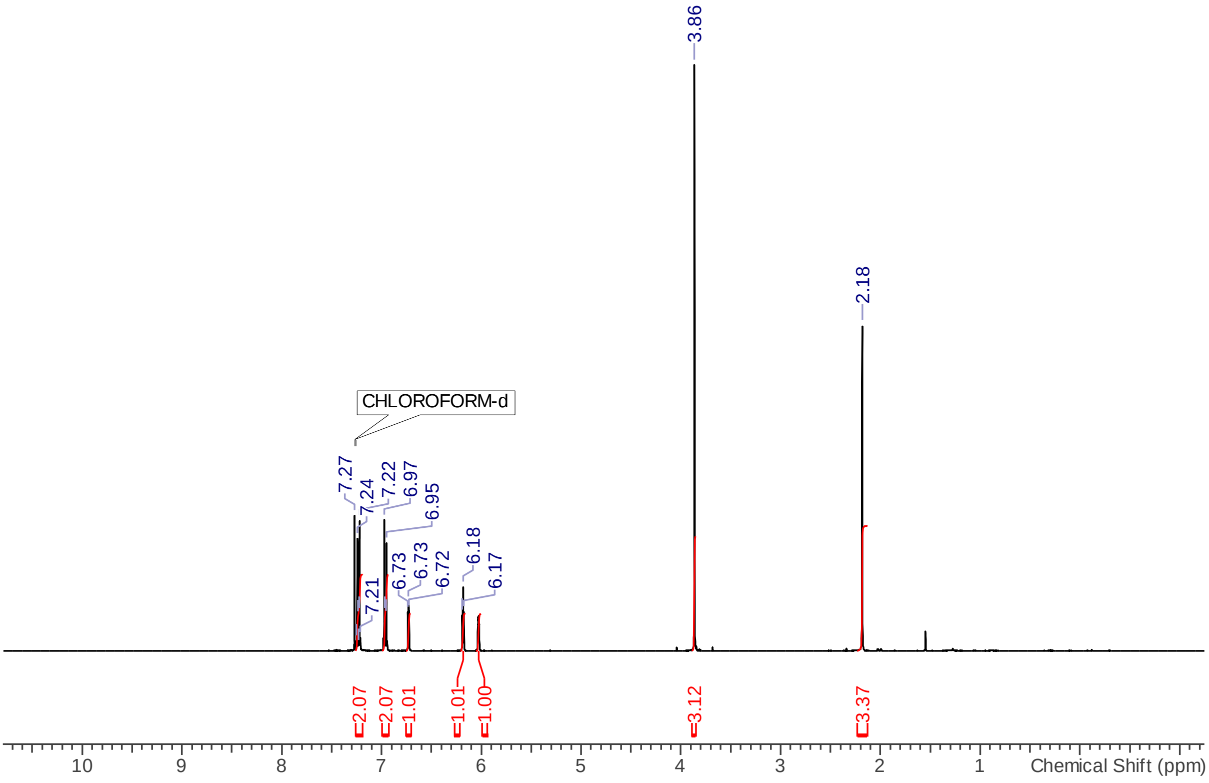

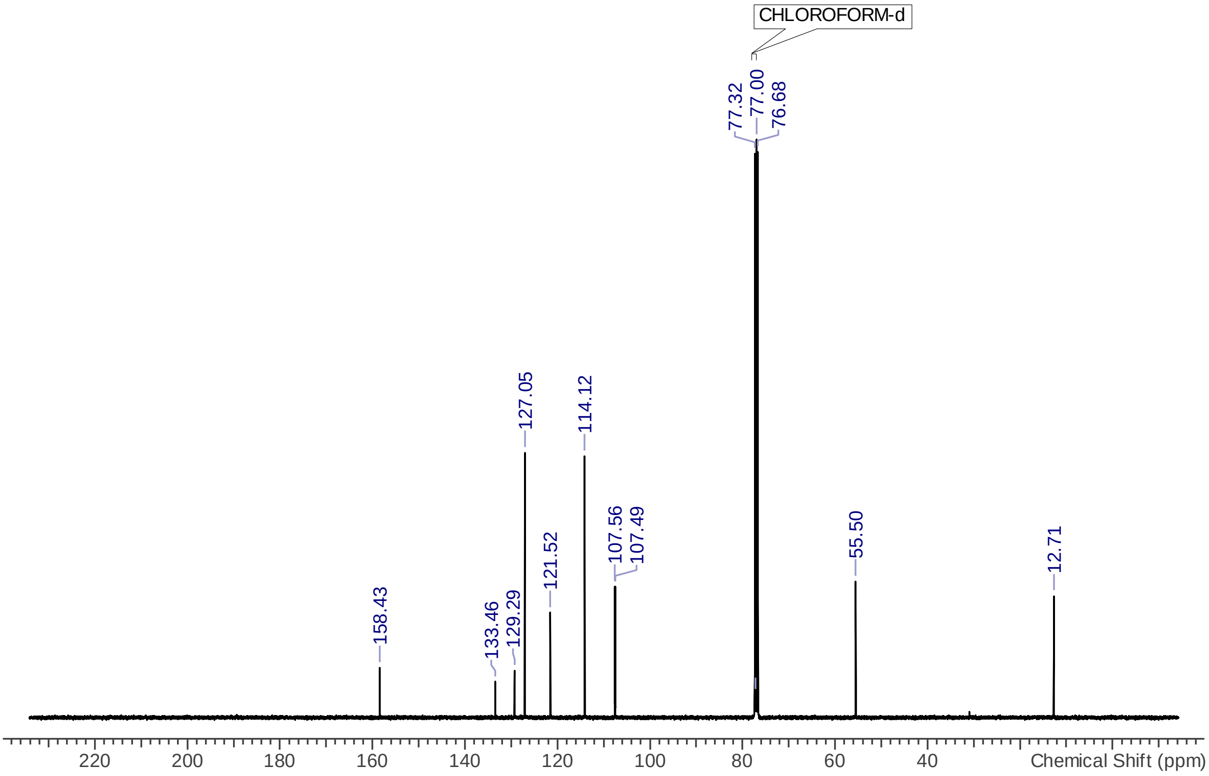

3-(2,5-Dimethyl-1H-pyrrol-1-yl)benzoic acid 3n

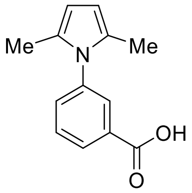

The title compound was prepared according to general procedure 7 using 3-(2,5-dimethylpyrrolidin-1-yl)benzoic acid **1n** (43.8 mg, 0.20 mmol) with an NMR yield of 14%. Purification by flash column chromatography on silica gel (eluent = 10% MeOH in 1:1 DCM: pet. ether) gave the title compound **3n** as a brown oil (3.8 mg, 0.02 mmol, 9%). **R$_f$** = 0.27 (eluent = 10% MeOH in 1:1 DCM: pet. ether); **^{1}H NMR** (400 MHz, $CDCl_3$) δ_H = 8.15 (1H, ddd, J = 7.7, 1.8, 1.2 Hz), 7.99 (1H, dd, *J* = 2.1, 1.8 Hz), 7.60 (1H, dd, *J* = 7.9, 7.7 Hz), 7.49 (1H, ddd, *J* = 7.9, 2.1, 1.2 Hz), 5.94 (2H, s), 2.06 (6H, s); **^{13}C NMR** (101 MHz, $CDCl_3$) δ_C = 170.2 (C=O), 139.4 (C), 133.5 (CH), 130.4 (C), 129.9 (CH), 129.4 (CH), 129.3 (CH), 128.7 (2×C), 106.3 (2×CH), 13.0 (2×CH_3); Spectroscopic data in accordance with that stated in the literature.[15]

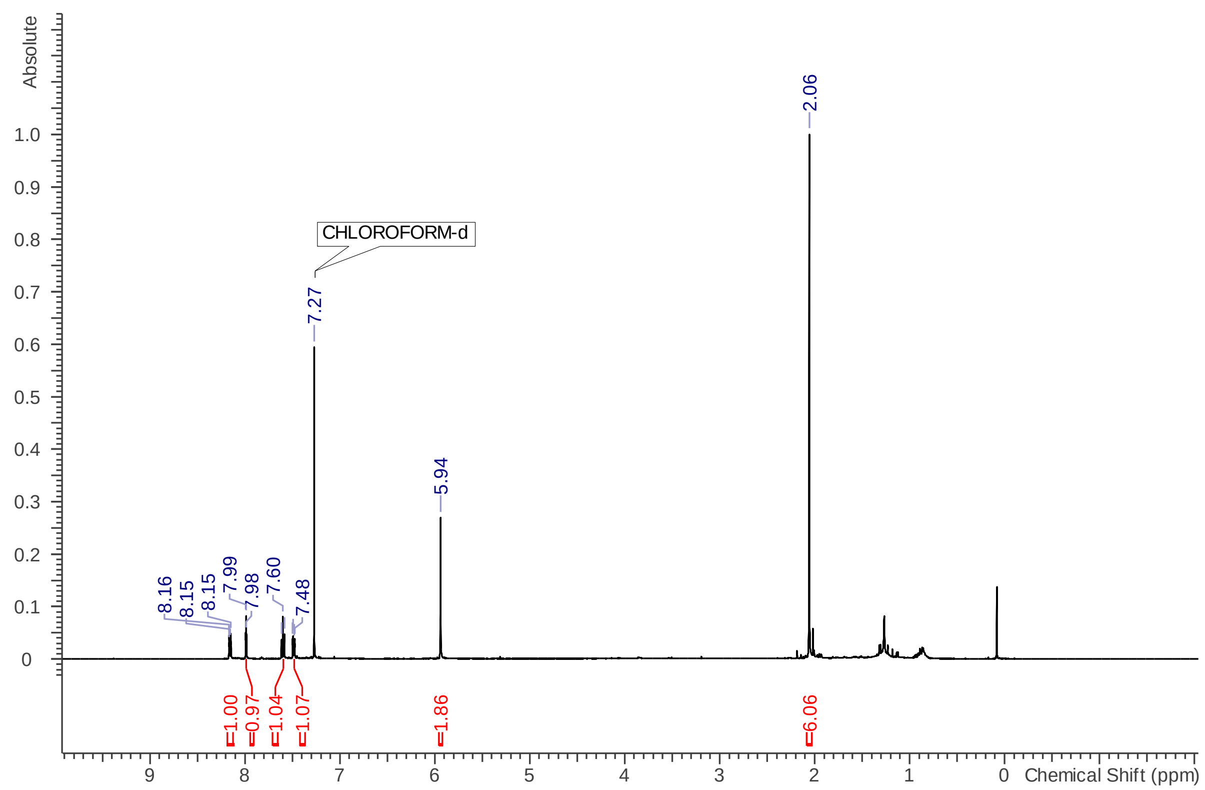

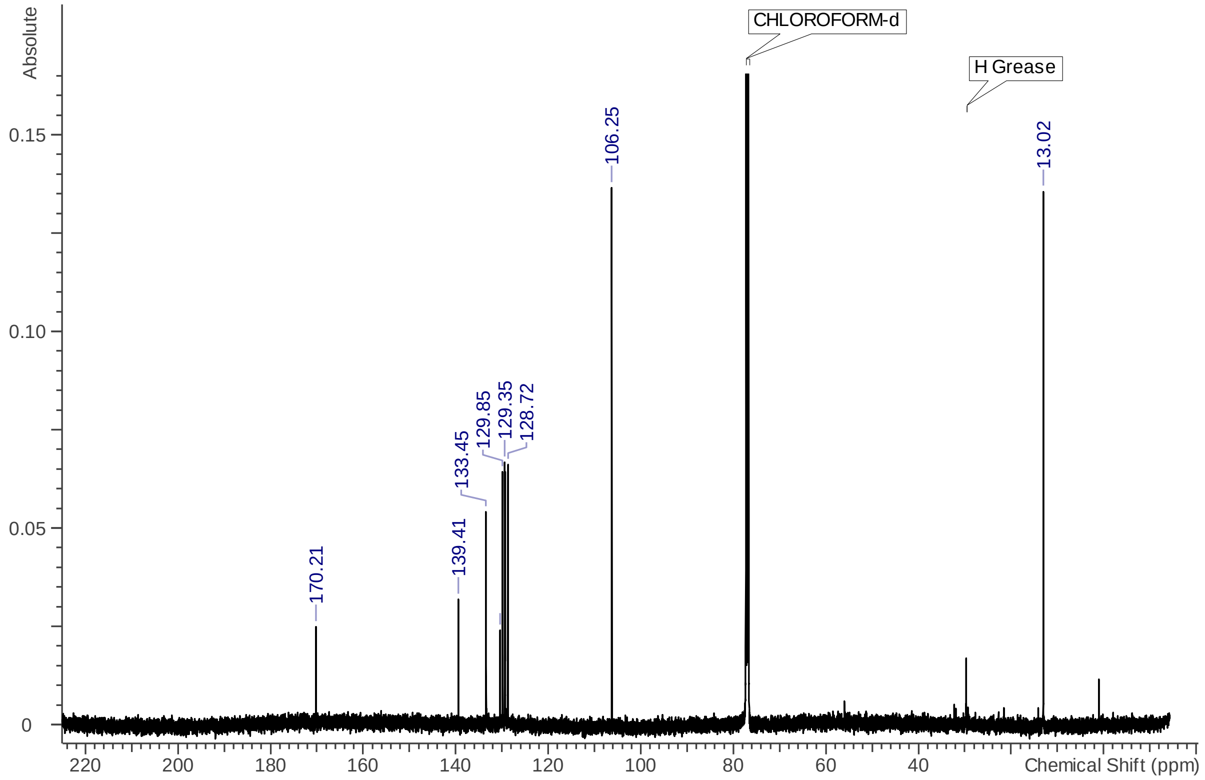

1-(3-(((*tert*-Butyldimethylsilyl)oxy)methyl)phenyl)-2,5-dimethyl-1*H*-pyrrole 3o

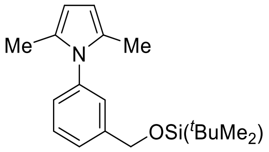

The title compound was prepared according to general procedure 5 using 1-(3-(((tert-butyldimethylsilyl)oxy)methyl)phenyl)-2,5-dimethylpyrrolidine **1o** (63.9 mg, 0.20 mmol) with an NMR yield of 52%. Purification by flash column chromatography on silica gel (eluent = 20% MeOH in 1:1DCM: pet. ether) gave the title compound **3o** as a colourless oil (27.3 mg, 0.09 mmol, 43%); **R$_f$** = 0.5 (eluent = 20% DCM in pet. ether). ^{1}**H NMR** (400 MHz, $CDCl_3$) δ_H = 7.42 (1H, app. t, *J* = 7.7 Hz), 7.38–7.33 (1H, m), 7.21–7.18 (1H, m), 7.12–7.07 (1H, m), 5.91 (2H, s), 4.79 (2H, s), 2.04 (6H, s), 0.94 (9H, s), 0.11 (6H, s); ^{13}**C NMR** (101 MHz, $CDCl_3$) δ_C = 142.7 (C), 138.9 (C), 128.8 (2×C), 126.7 (CH), 125.8 (CH), 125.2 (CH), 105.5 (2×CH), 64.5 (CH_2), 25.9 (3×CH_3), 18.4 (C), 13.0 (2×CH_3), −5.3 (2×CH_3); **HRMS** (ESI$^+$): calculated for $[C_{19}H_{29}NOSi]^+$ $(M+H)^+$ m/z: 316.2097; found 316.2083.

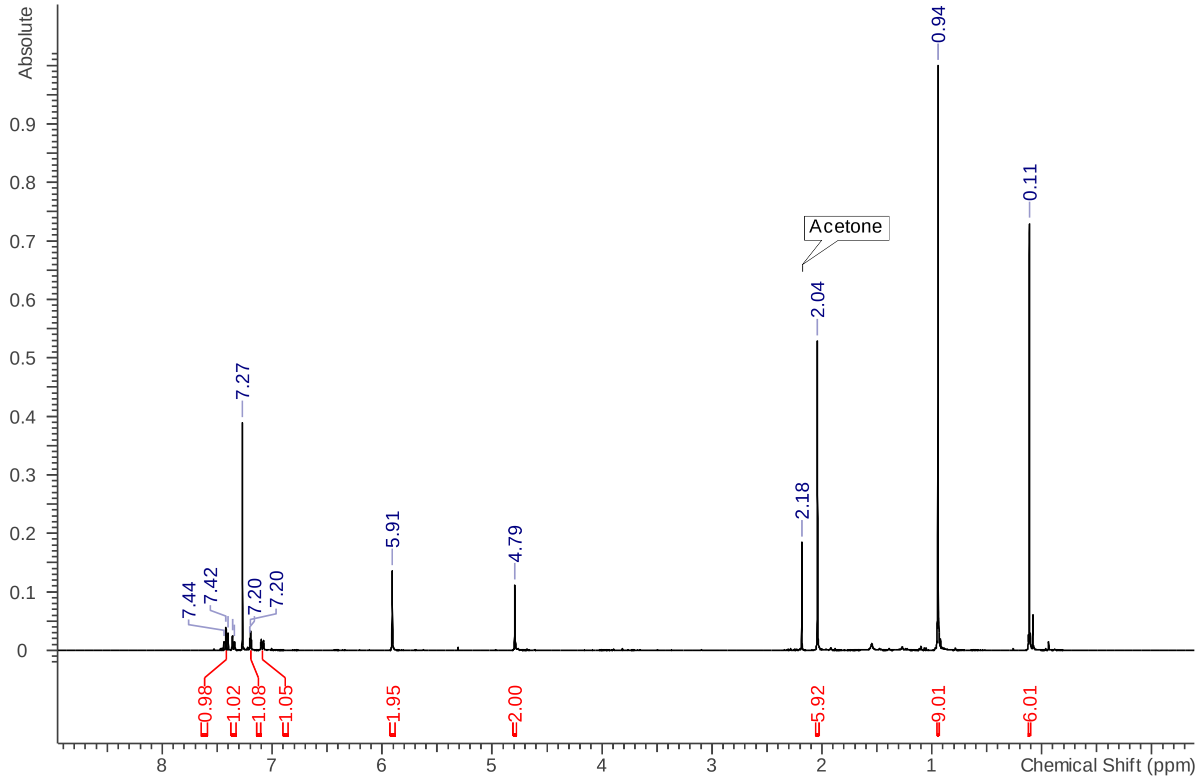

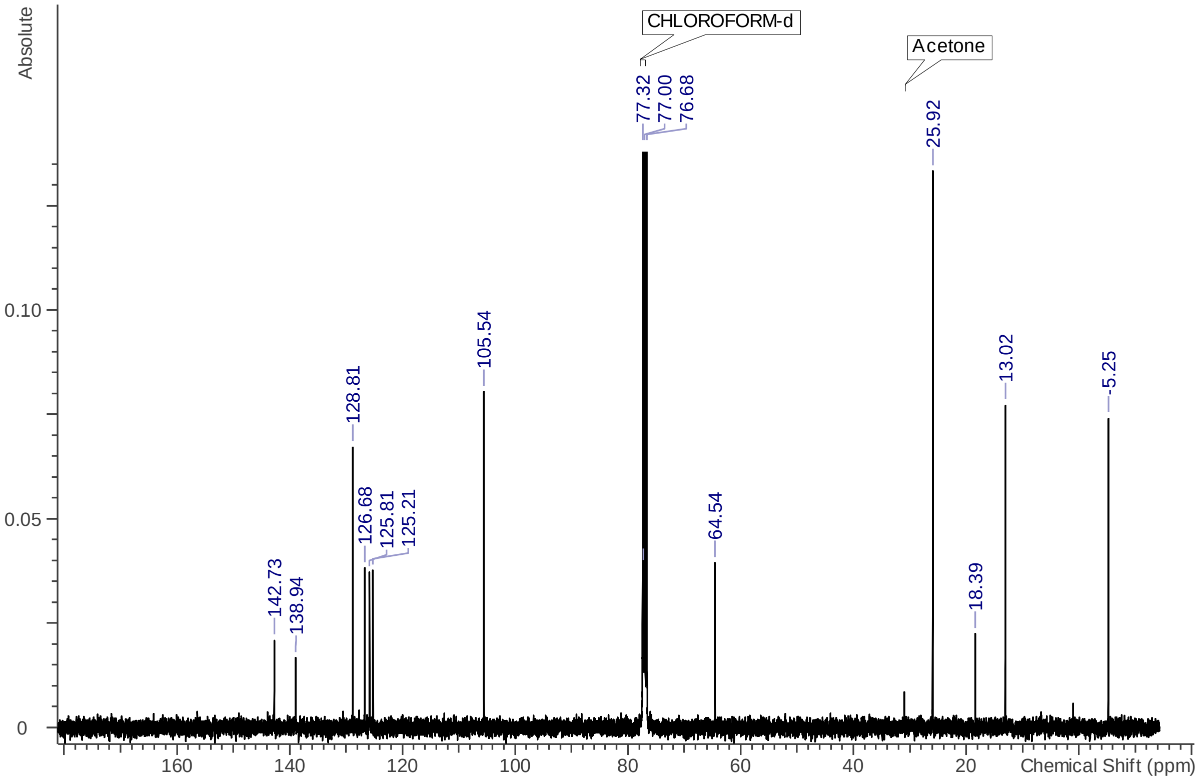

1-(4-Bromo-2-(*tert*-butyl)phenyl)-1*H*-pyrrole 3p

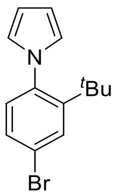

The title compound was prepared according to general procedure 5 using 1-(4-bromo-2-(*tert*-butyl)phenyl)pyrrolidine **1p** (56.2 mg, 0.20 mmol) with an NMR yield of 80%. Purification by flash column chromatography on silica gel (eluent = 10% DCM in pet. ether) gave the title compound **3p** as a colourless oil (36.4 mg, 0.13 mmol, 66%). **R$_f$** = 0.4 (eluent = 10% DCM in pet. ether); **^{1}H NMR** (400 MHz, $CDCl_3$) δ_H = 7.66 (1H, d, *J* = 2.3 Hz), 7.34 (1H, dd, *J* = 8.3, 2.3 Hz), 6.96 (1H, d, *J* = 8.3 Hz), 6.72 (2H, app. t, *J* = 2.3 Hz), 6.25 (2H, app. t, *J* = 2.3 Hz), 1.19 (9H, s); **^{13}C NMR** (101 MHz, $CDCl_3$) δ_C = 149.4 (C), 139.2 (C), 132.6 (CH), 131.0 (CH), 129.3 (CH), 124.4 (2×CH), 122.5 (C), 108.3 (2×CH), 36.0 (C), 31.4 (3×CH_3); **HRMS** (ESI$^+$): calculated for $[C_{14}H_{17}N^{79}Br]^+$ (M+H)$^+$ m/z: 278.0544; found 278.0544; calculated for $[C_{14}H_{17}N^{81}Br]^+$ (M+H)$^+$ m/z: 280.0524; found 280.0521.

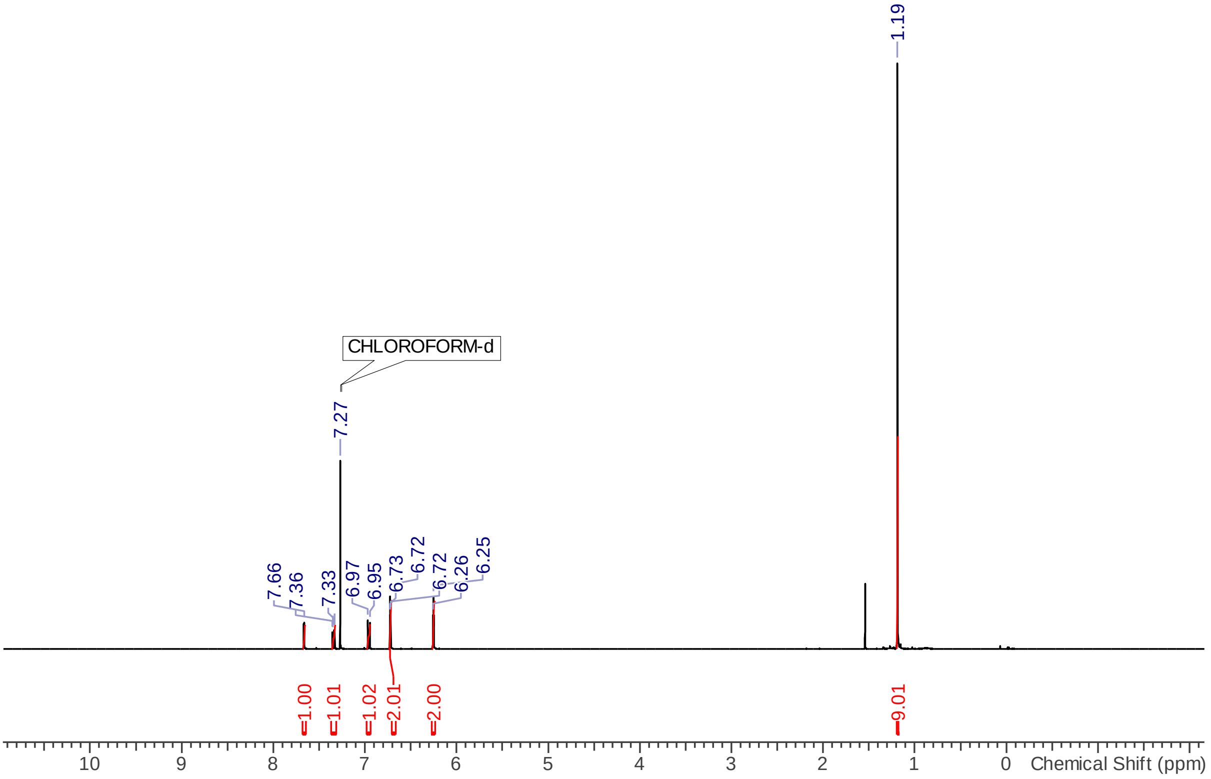

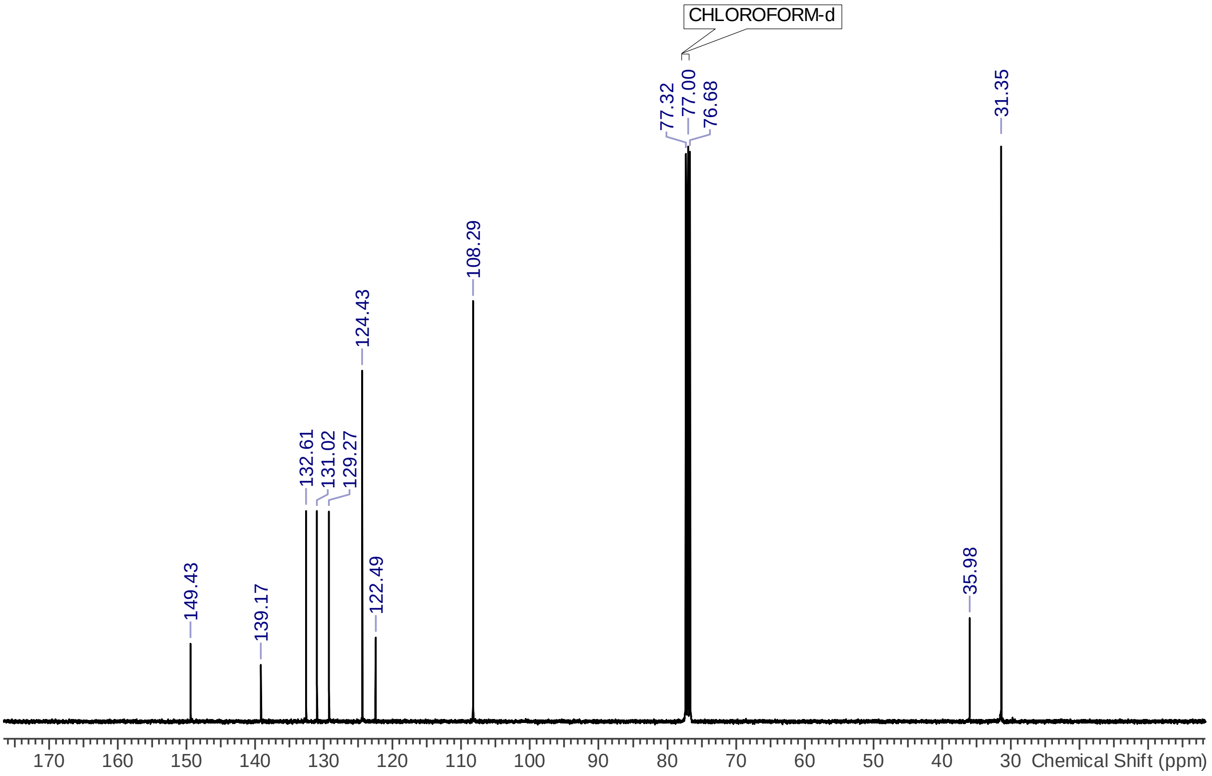

1-(2-(*tert*-Butyl)-4-(pinacolboryl)phenyl)pyrrole 3q

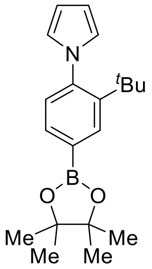

The title compound was prepared according to general procedure 5 using 1-(2-(*tert*-butyl)-4-(pinacolboryl)phenyl)pyrrolidine **1q** (65.8 mg, 0.20 mmol) with an NMR yield of 50%. Purification by flash column chromatography on silica gel (eluent = 0-5% EtOAc in 1:1 $CHCl_3$:pet. ether) gave the title compound **3q** as a colourless oil (5.6 mg, 0.02 mmol, 9%). **R$_f$** (50% DCM/petroleum ether) 0.30. **^{1}H NMR** (400 MHz, $CDCl_3$) δ_H = 8.02 (1H, d, *J* = 1.4 Hz), 7.67 (1H, dd, *J* = 7.7, 1.4 Hz), 7.10 (1H, d, *J* = 7.7 Hz), 6.75 (2H, app. t, *J* = 2.1 Hz), 6.25 (2H, app. t, *J* = 2.1 Hz), 1.37 (12H, s), 1.23 (9H, s). **^{11}B NMR (**128 MHz, $CDCl_3$) δ_B = 31.0. **^{13}C NMR** (101 MHz, $CDCl_3$) δ_C = 146.2 (C), 142.7 (C), 134.3 (CH), 132.7 (CH), 130.5 (CH), 127.7 (C) 124.4 (2×CH), 107.9 (2×CH), 83.9 (2×C), 35.8 (C), 31.6 (3×CH_3), 24.9 (4×CH_3). **HRMS** (ESI): calculated for $[C_{20}H_{29}{}^{11}BNO_2]^+$ $(M+H)^+$ m/z: 326.2291; found 326.2303.

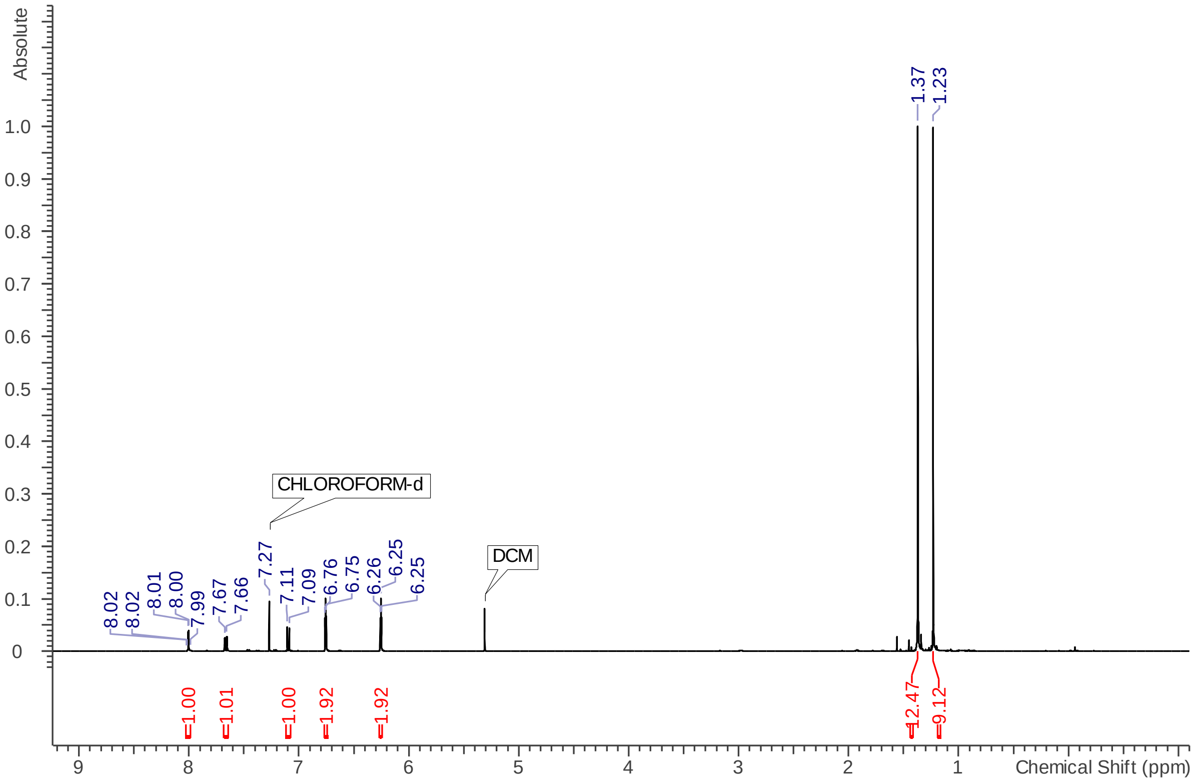

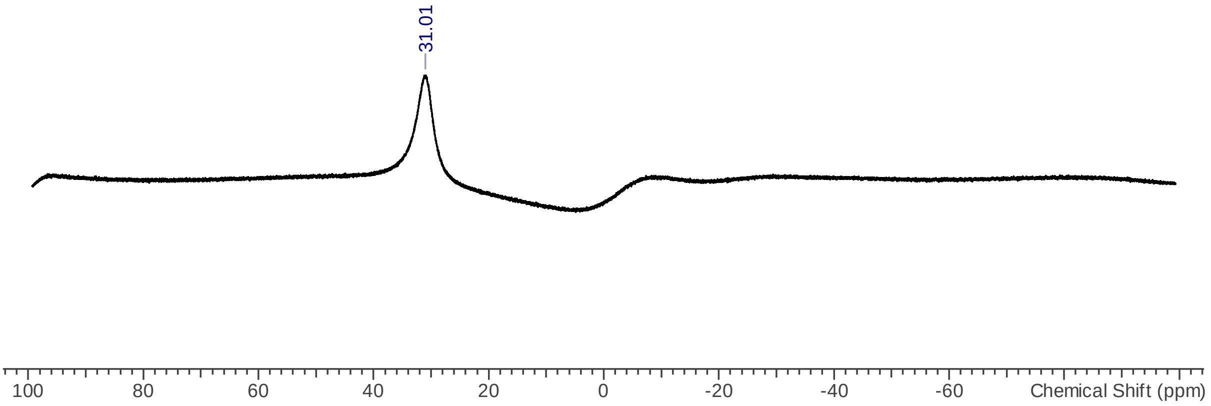

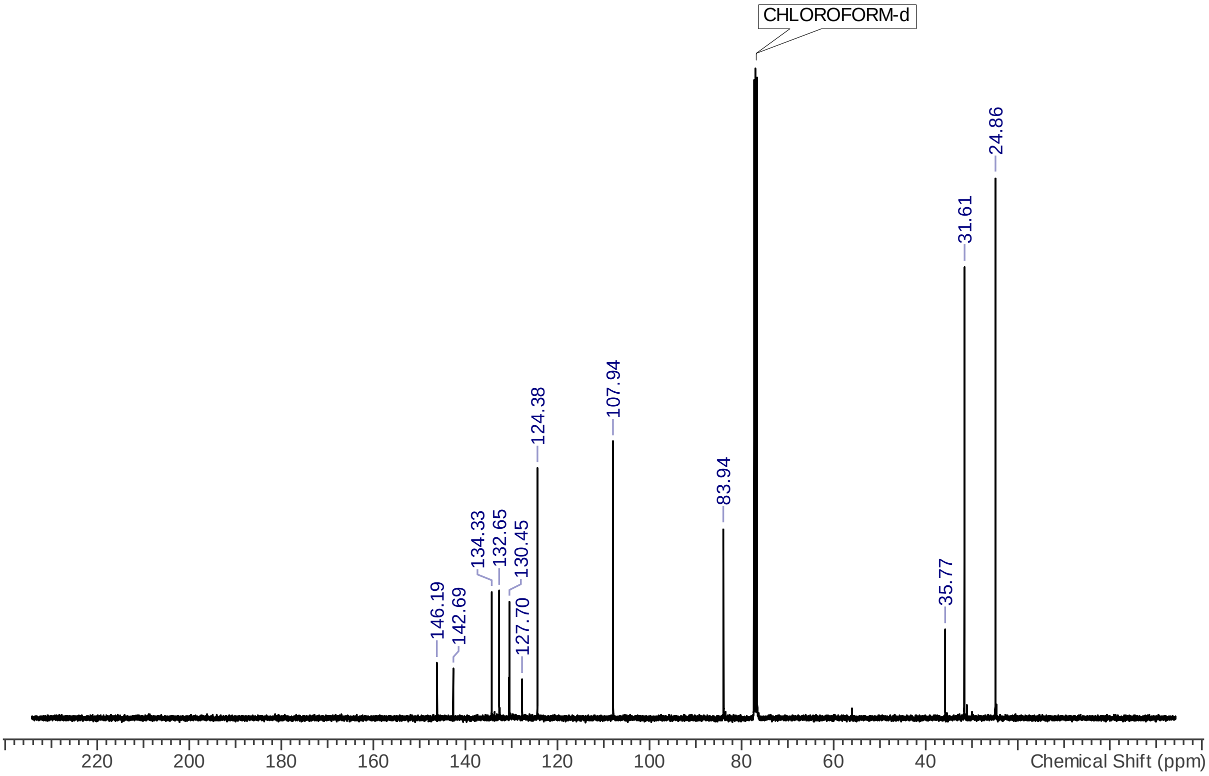
CHLOROFORM-d
146.19
142.69
134.33
132.65
130.45
127.70
124.38
107.94
83.94
35.77
31.61
24.86
220
200
180
160
140
120
100
80
60
40
Chemical Shift (ppm)

1-(2,4-Dichlorophenyl)-2-methyl-1*H*-pyrrole 3r

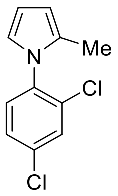

The title compound was prepared according to general procedure 5 using 1-(2,4-dichlorophenyl)-2-methylpyrrolidine **1r** (45.8 mg, 0.20 mmol) with an NMR yield of 76%. Purification by flash column chromatography on silica gel (eluent = 10% DCM in pet. ether) gave the title compound **3r** as a colourless oil (29.3 mg, 0.14 mmol, 69%). $\mathbf{R_f}$ = 0.4 (eluent = 10% DCM in pet. ether); **^{1}H NMR** (400 MHz, $CDCl_3$) δ_H = 7.56 (1H, d, *J* = 2.2 Hz), 7.35 (1H, dd, *J* = 8.4, 2.2 Hz), 7.29 (1H, d, *J* = 8.4 Hz), 6.62 (1H, dd, *J* = 2.9, 1.8 Hz), 6.25 (1H, dd, *J* = 3.3, 2.9 Hz), 6.07 (1H, ddq, *J* = 3.3, 1.8, 0.6 Hz), 2.06 (3H, d, *J* = 0.6 Hz); **^{13}C NMR** (101 MHz, $CDCl_3$) δ_C = 136.7 (C), 134.6 (C), 133.7 (C), 130.4 (CH), 130.0 (C, CH), 127.7 (CH), 121.3 (CH), 108.5 (CH), 107.4 (CH), 12.0 (CH_3); **HRMS** (ESI$^+$): calculated for $[C_{11}H_{10}N^{35}Cl_2]^+$ $(M+H)^+$ m/z: 226.0190; found 226.0184; calculated for $[C_{11}H_{10}N^{35}Cl^{37}]^+$ $(M+H)^+$ m/z: 238.0161; found 238.0155; calculated for $[C_{11}H_{10}N^{37}Cl_2]^+$ $(M+H)^+$ m/z: 230.0131; found 230.0140.

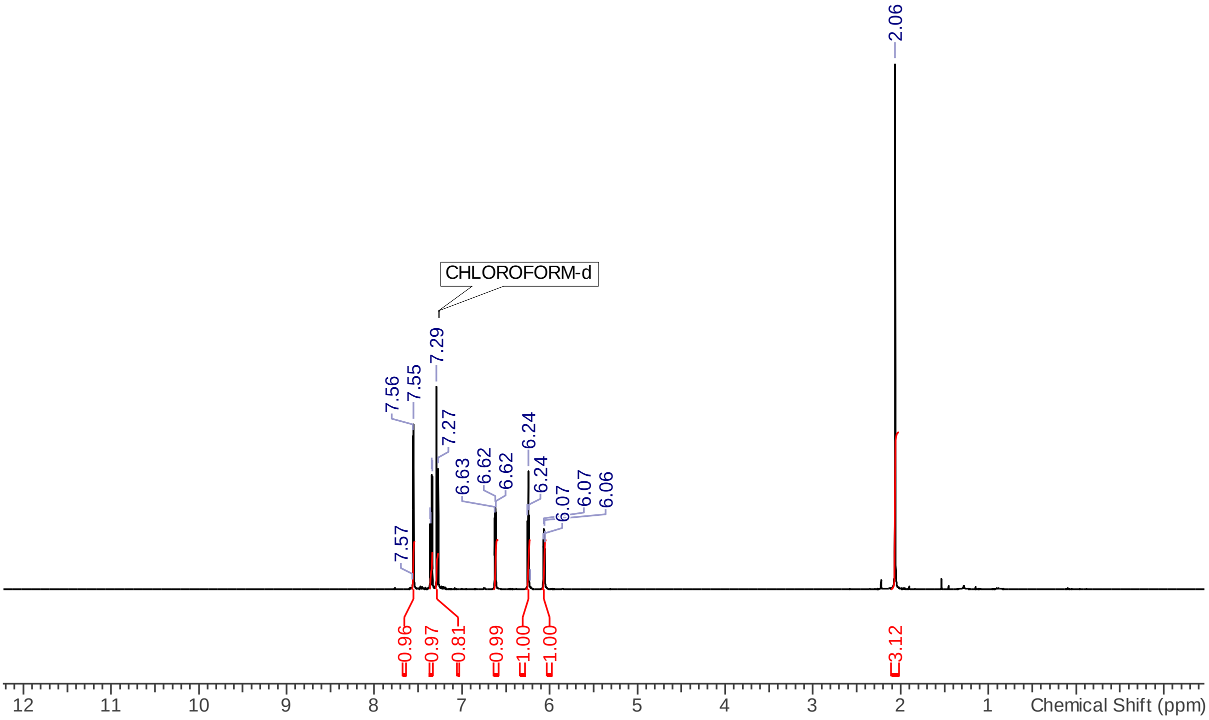

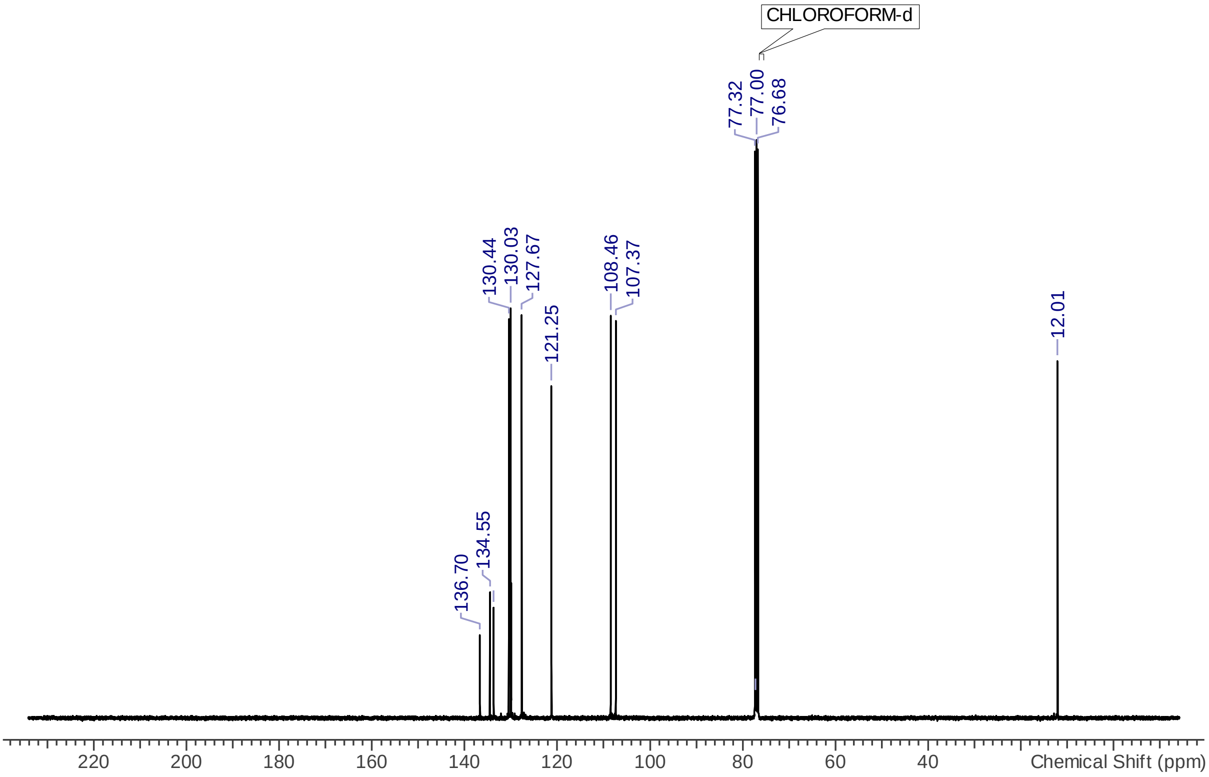

1-(5-Chloro-2-methoxyphenyl)-2-methyl-1H-pyrrole 3s

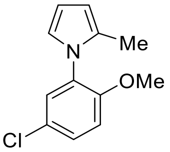

The title compound was prepared according to general procedure 5 (reaction done using DCE, heated to 85 °C) using 1-(5-chloro-2-methoxyphenyl)-2-methylpyrrolidine **1s** (45.02 mg, 0.2 mmol) with an NMR yield of 66%. Purification by flash column chromatography on silica gel (eluent = 20% DCM in pet. ether) gave the title compound **3s** as a colourless oil (28.8 mg, 0.13 mmol, 65%). **R_f** = 0.30 (eluent = 20% DCM in pet. ether); **^{1}H NMR** (400 MHz, $CDCl_3$) δ_H = 7.33 (1H, dd, *J* = 8.8, 2.7 Hz), 7.24 (1H, d, *J* = 2.7 Hz), 6.95 (1H, d, *J* = 8.8 Hz), 6.64 (1H, dd, *J* = 2.8, 1.8 Hz), 6.21 (1H, dd, *J* = 3.4, 2.8 Hz), 6.03 (1H, ddq, *J* = 3.4, 1.8, 0.8 Hz), 3.79 (3H, s), 2.08 (3H, d, *J* = 0.8 Hz); **^{13}C NMR** (101 MHz, $CDCl_3$) δ_C = 153.8 (C), 130.4 (C), 130.1 (C), 129.0 (CH), 128.7 (CH), 125.2 (C), 121.6 (CH), 113.0 (CH), 108.1 (CH), 107.1 (CH), 56.0 (CH_3), 12.1 (CH_3); **HRMS** (ESI$^+$): calculated for $[C_{12}H_{13}NO^{35}Cl]^+$ $(M+H)^+$ m/z: 222.0686; found 222.0683; calculated for $[C_{12}H_{13}NO^{37}Cl]^+$ $(M+H)^+$ m/z: 224.0656; found 224.0655.

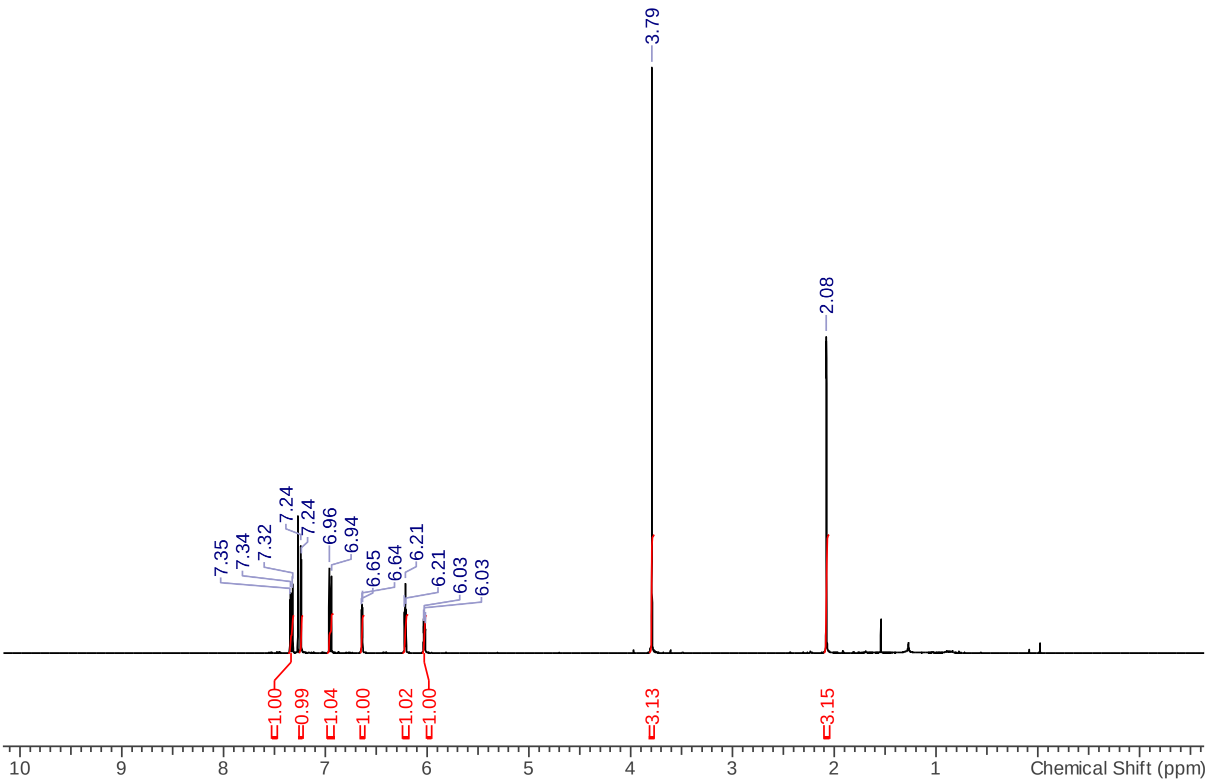

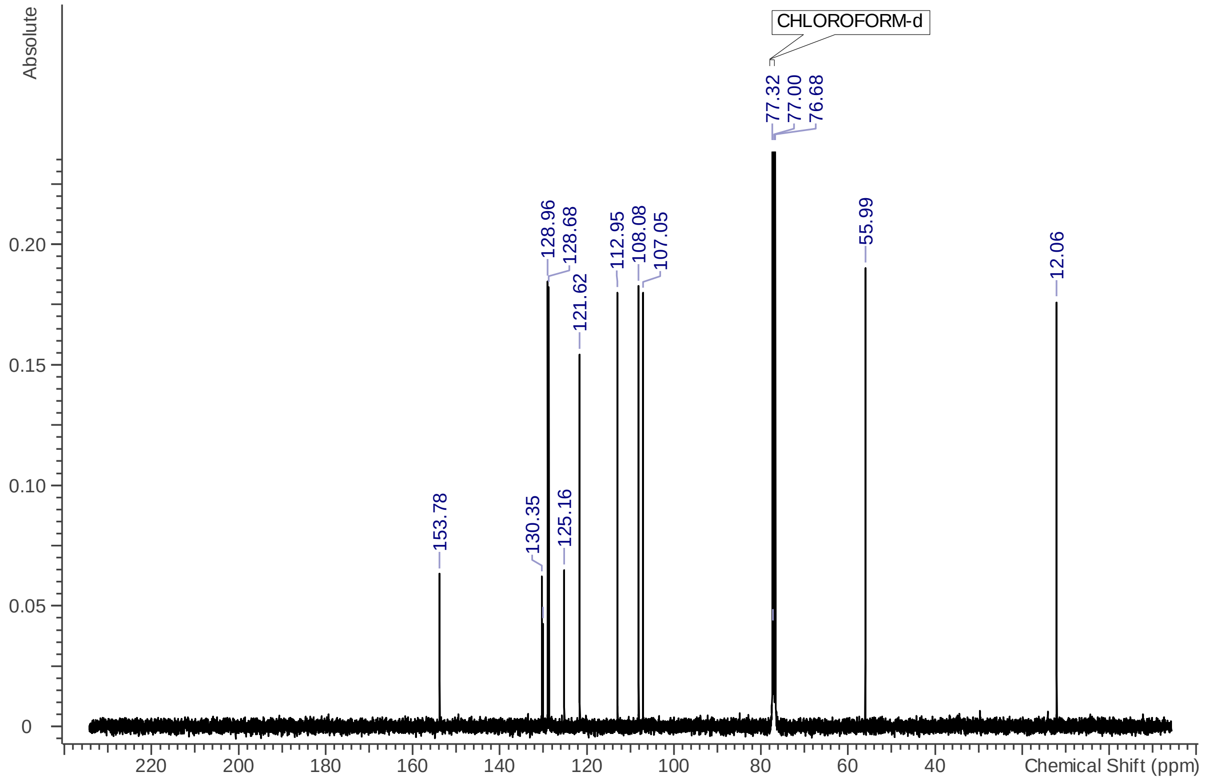

1-(2-Chloro-5-(trifluoromethyl)phenyl)-2-methyl-1*H*-pyrrole 3t

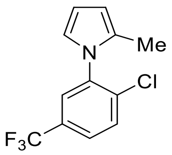

The title compound was prepared according to general procedure 5 (reaction done using *p*-xylene, heated to 120 °C) using 1-(2-chloro-5-(trifluoromethyl)phenyl)-2-methylpyrrolidine **1t** (52.6 mg, 0.20 mmol) with an NMR yield of 74%. Purification by flash column chromatography on silica gel (eluent = 10% DCM in pet. ether) gave the title compound **3t** as a colourless oil (20.8 mg, 0.08 mmol, 40%). **R**$_f$ = 0.3 (eluent = 10% DCM in pet. ether); **^{1}H NMR** (400 MHz, $CDCl_3$) δ_H = 7.71–7.62 (3H, m), 6.65 (1H, dd, *J* = 2.9, 1.8 Hz), 6.27 (1H, dd, *J* = 3.4, 2.9 Hz), 6.08 (1H, ddq, *J* = 3.4, 1.8, 0.8 Hz), 2.07 (3H, d, *J* = 0.8 Hz); **^{13}C NMR** (101 MHz, $CDCl_3$) δ_C = 138.7 (C), 136.9, (C), 130.9 (CH) 130.1 (C, q, $^2J_{C\text{-}F}$ = 33.6 Hz), 130.0 (C), 126.8 (CH, q, $^3J_{C\text{-}F}$ = 3.6 Hz), 126.1 (CH, q, $^3J_{C\text{-}F}$ = 3.6 Hz), 123.2 (C, q, $^1J_{C\text{-}F}$ = 272 Hz), 121.2 (CH), 108.9 (CH), 107.8 (CH), 12.0 (CH_3); **^{19}F NMR** (376 MHz, $CDCl_3$): δ_F = −62.60 ppm (3F, s); **HRMS** (ESI^+): calculated for $[C_{12}H_{10}NF_3{}^{35}Cl]^+$ $(M+H)^+$ m/z: 260.0454; found 260.0452; calculated for $[C_{12}H_{10}NF_3{}^{37}Cl]^+$ $(M+H)^+$ m/z: 262.0424; found 262.0426.

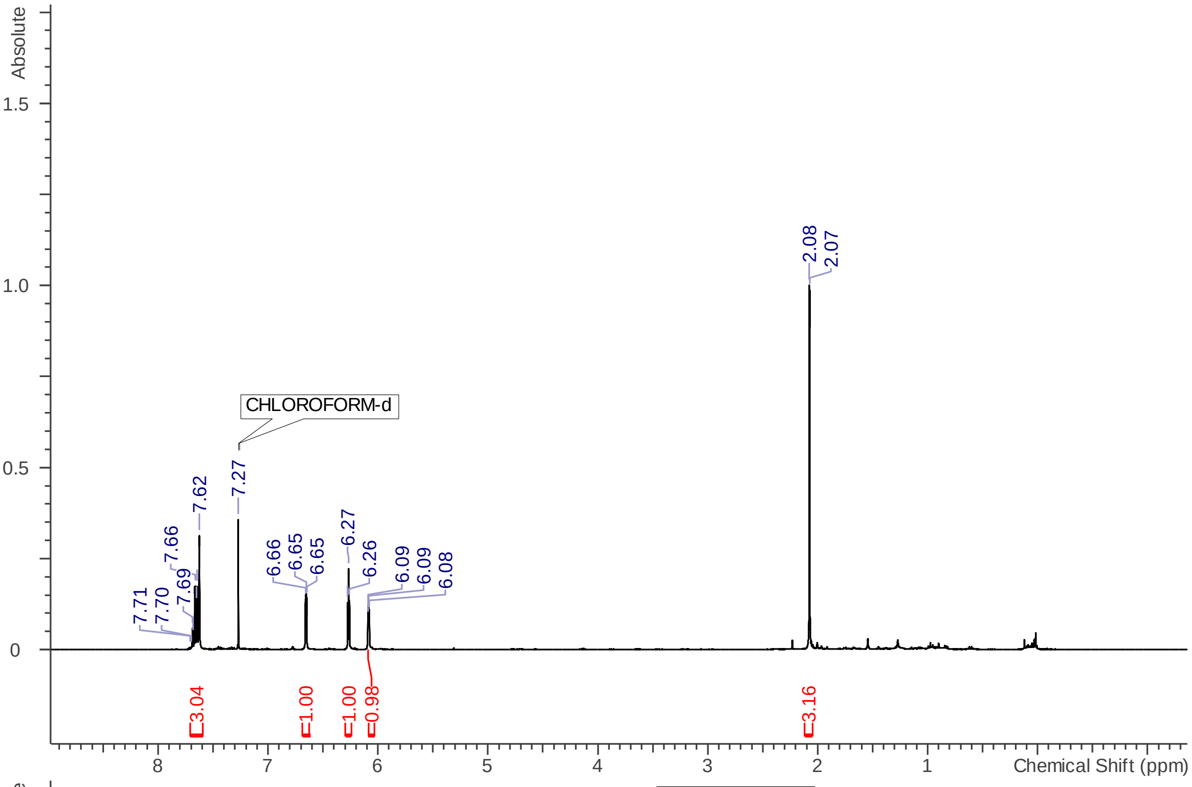

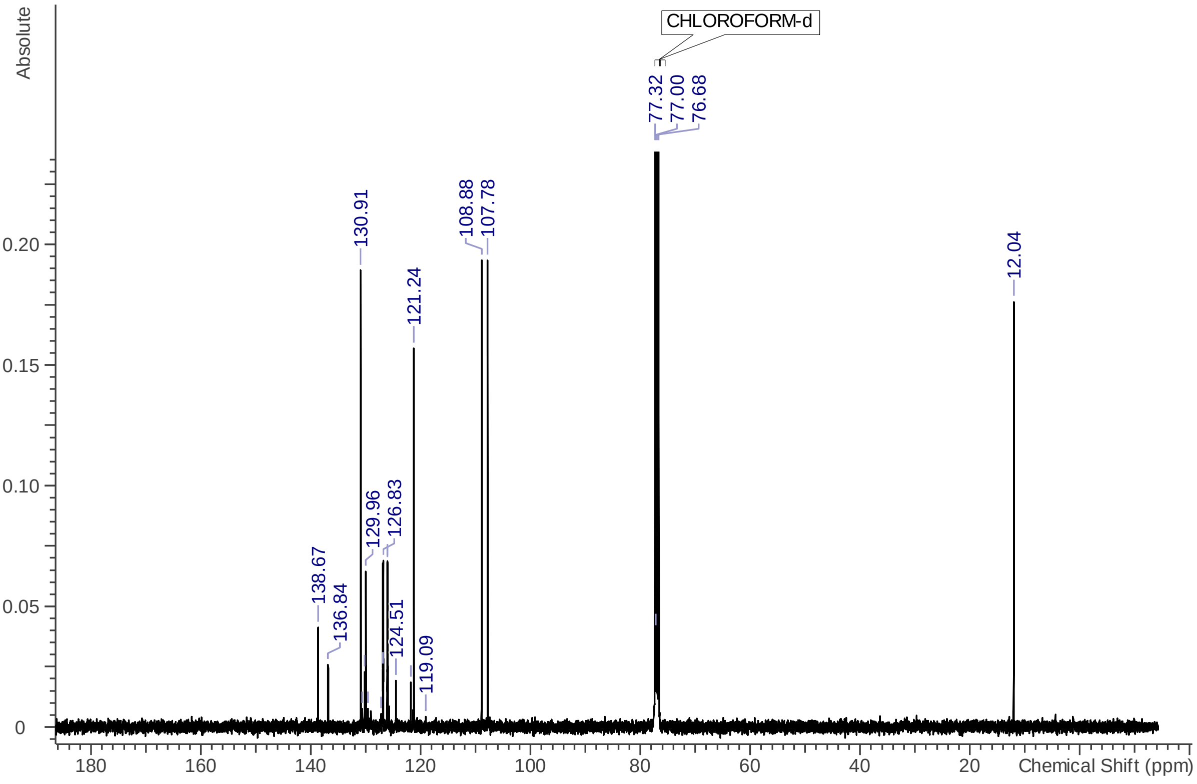

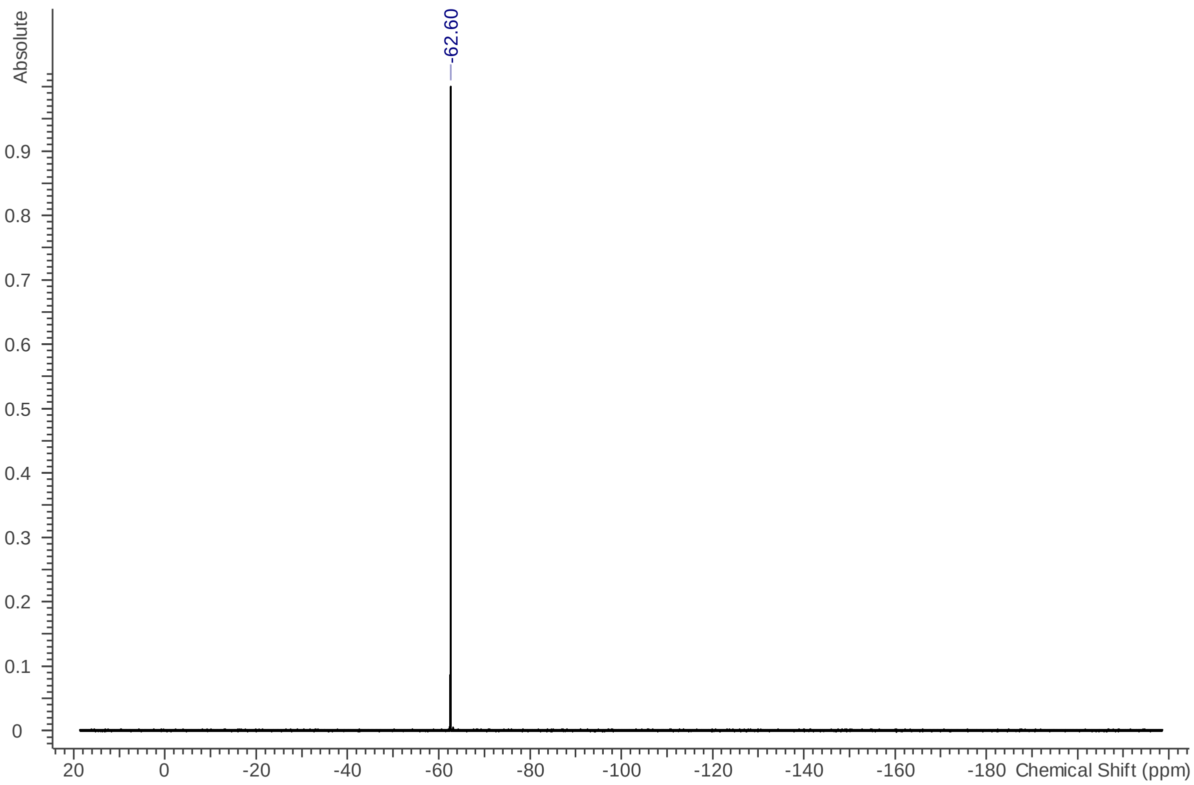
-62.60
Absolute
0.9
0.8
0.7
0.6
0.5
0.4
0.3
0.2
0.1
0
20
0
-20
-40
-60
-80
-100
-120
-140
-160
-180
Chemical Shift (ppm)

1-(3-Chloro-4-methylphenyl)-2,5-dimethyl-1*H*-pyrrole 3u

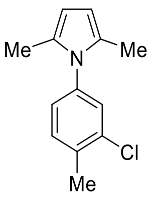

The title compound was prepared according to general procedure 5 using 1-(3-Chloro-4-methylphenyl)-2,5-dimethyl-1*H*-pyrrolidine **1u** (44.6 mg, 0.20 mmol) with an NMR yield of 65%. Purification by flash column chromatography on silica gel (10% DCM in pet. ether) gave the title compound **3u** as a yellow oil (26.2 mg, 0.12 mmol, 59%). $\mathbf{R_f}$ = 0.3 (eluent = 10% DCM in pet. ether); **^{1}H NMR** (400 MHz, $CDCl_3$) δ_H = 7.32 (1H, d, *J* = 8.0 Hz), 7.23 (1H, d, *J* = 1.9 Hz), 7.03 (1H, dd, *J* = 8.0, 1.9 Hz), 5.90 (2H, s), 2.45 (3H, s), 2.05 (6H, s); **^{13}C NMR** (101 MHz, $CDCl_3$) δ_C = 137.7 (C), 135.7 (C), 134.5 (C), 131.1 (CH), 128.8 (2×C) 128.7 (CH), 126.5 (CH), 105.9 (2×CH), 19.8 (CH_3), 13.0 (2×CH_3); **HRMS** (ESI$^+$): calculated for $[C_{13}H_{15}N^{35}Cl]^+$ (M+H)$^+$ m/z: 220.0893; found 220.0892; calculated for $[C_{13}H_{15}N^{37}Cl]^+$ (M+H)$^+$ m/z: 222.0864; found 222.0860.

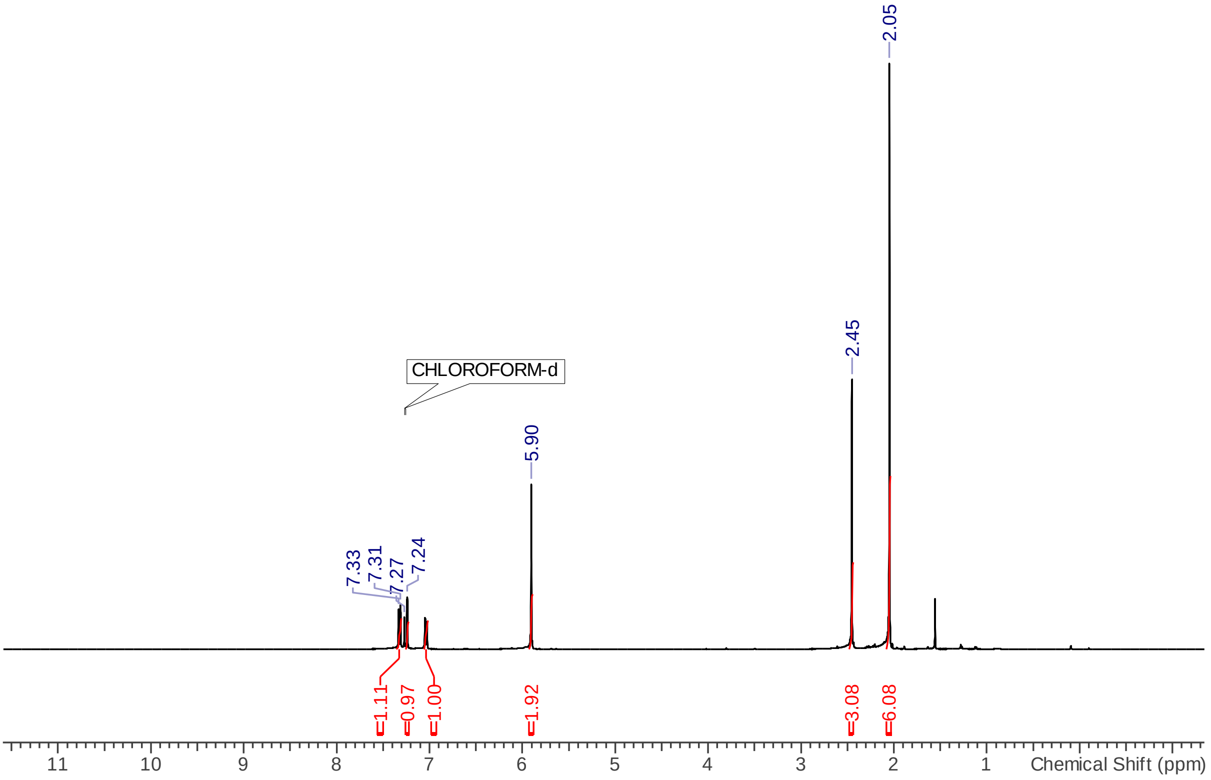

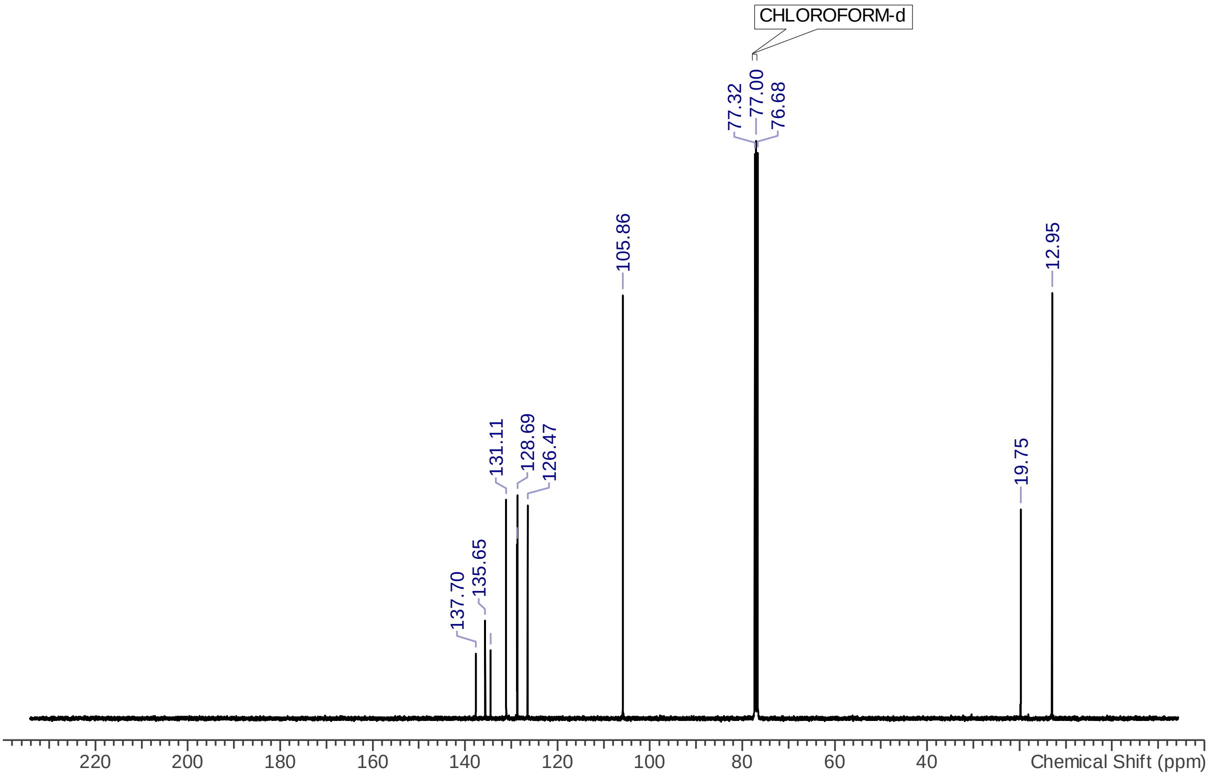

1-(3-Chloro-4-methylphenyl)-2,5-dimethyl-1*H*-pyrrole 3v

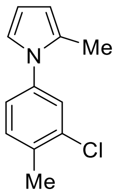

The title compound was prepared according to general procedure 5 using 1-(3-chloro-4-methylphenyl)-2-methylpyrrolidine **1v** (41.8 mg, 0.20 mmol) with an NMR yield of 72%. Purification by flash column chromatography on silica gel (eluent = 10% DCM in pet. ether) gave the title compound **3v** as a colourless oil (13.8 mg, 0.07 mmol, 34%). **R$_f$** = 0.44 (eluent = 10% DCM in pet. ether); **^{1}H NMR** (400 MHz, $CDCl_3$) δ_H = 7.33 (1H, d, *J* = 2.2 Hz), 7.30 (1H, d, *J* = 8.1 Hz), 7.12 (1H, dd, *J* = 8.1, 2.2 Hz), 6.74 (1H, dd, *J* = 2.8, 1.8 Hz), 6.2 (1H, dd, *J* = 3.3, 2.8 Hz), 6.05 (1H, ddq, *J* = 3.3, 1.8, 0.7 Hz), 2.45 (s, 3H, CH_3), 2.05 (3H, d, *J* = 0.7 Hz); **^{13}C NMR** (101 MHz, $CDCl_3$) δ_C = 139.1 (C), 134.7 (C), 134.5 (C), 131.1 (CH), 129.0 (C), 126.2 (CH), 123.9 (CH), 121.3 (CH), 108.4 (CH), 108.3 (CH), 19.6 (CH_3), 12.8 (CH_3). **HRMS** (ESI$^+$): calculated for $[C_{12}H_{13}NO^{35}Cl]^+$ $(M+H)^+$ m/z: 206.0737; found 206.0735; calculated for $[C_{12}H_{13}N^{37}Cl]^+$ $(M+H)^+$ m/z: 208.0707; found 208.0722.

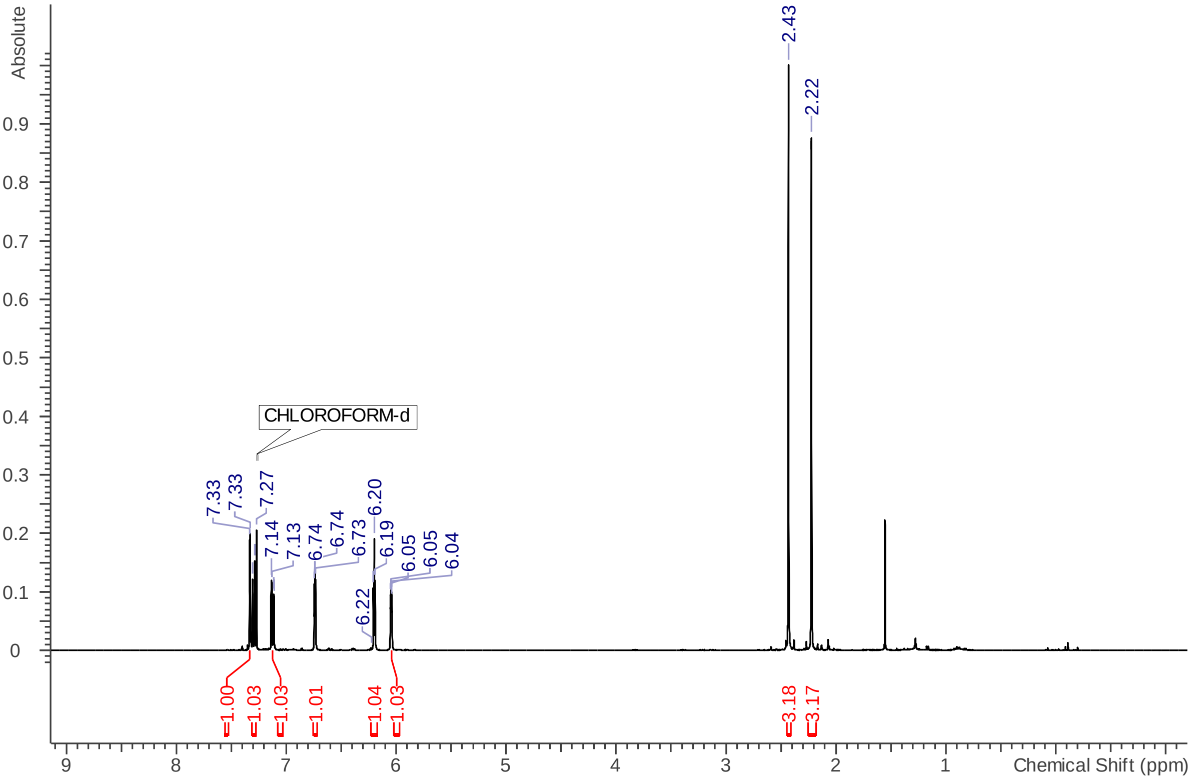

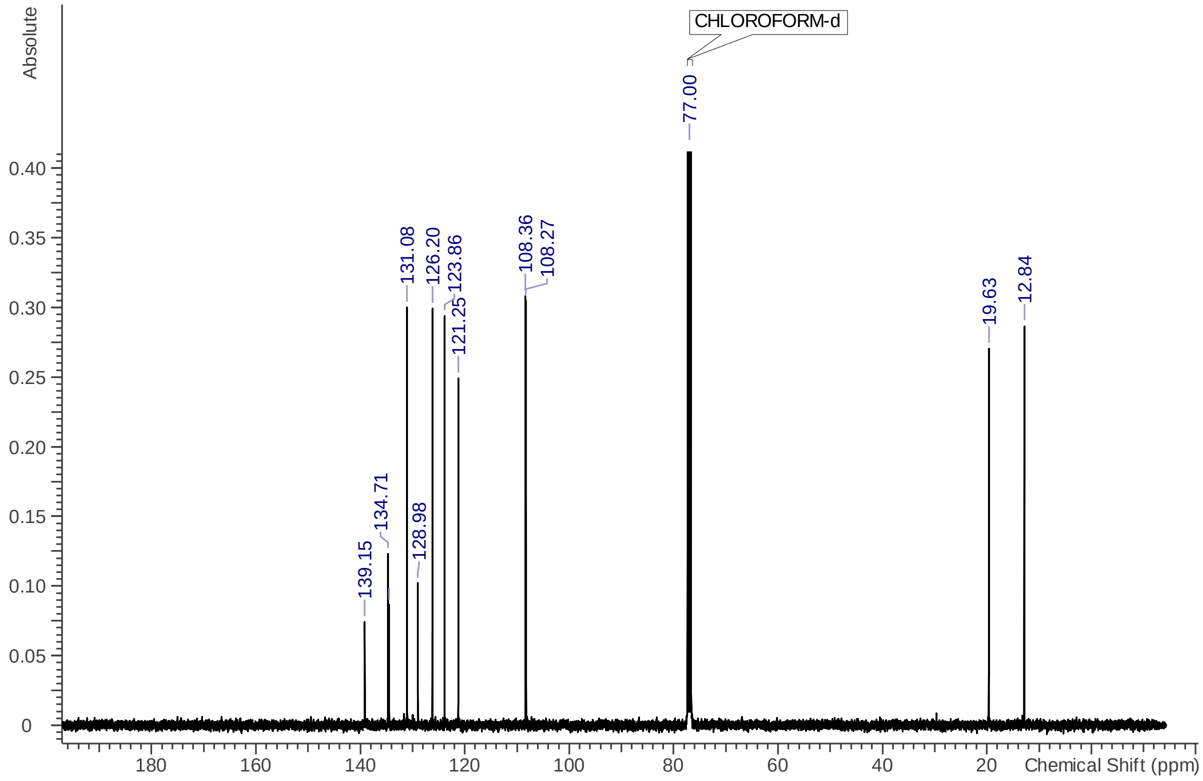

1-(4-Methoxy-2,6-dimethylphenyl)-1*H*-pyrrole 3w

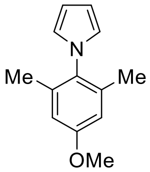

The title compound was prepared according to general procedure 5 using 1-(4-methoxy-2,6-dimethylphenyl)pyrrolidine **1w** (41.0 mg, 0.20 mmol) with an NMR yield of 71%. Purification by flash column chromatography on silica gel (eluent = 40% DCM in pet. ether) gave the title compound **3w** as a yellow oil (21.4 mg, 0.11 mmol, 53%). **R$_f$** = 0.4 (eluent = 40% DCM in pet. ether); **^{1}H NMR** (400 MHz, $CDCl_3$) δ_H = 6.66 (2H, s), 6.59 (2H, app. t, J = 2.0 Hz), 6.31 (2H, app. t, J = 2.0 Hz), 3.82 (3H, s), 2.02 (6H, s); **^{13}C NMR** (101 MHz, $CDCl_3$) δ_C = 158.7 (C), 137.5 (2×C), 133.1 (C), 121.8 (2×CH), 112.9 (CH), 108.4 (2×CH), 55.4 (CH_3), 11.6 (CH_3); **HRMS** (ESI$^+$): calculated for $[C_{13}H_{16}NO]^+$ $(M+H)^+$ m/z: 202.1232; found 202.1240.

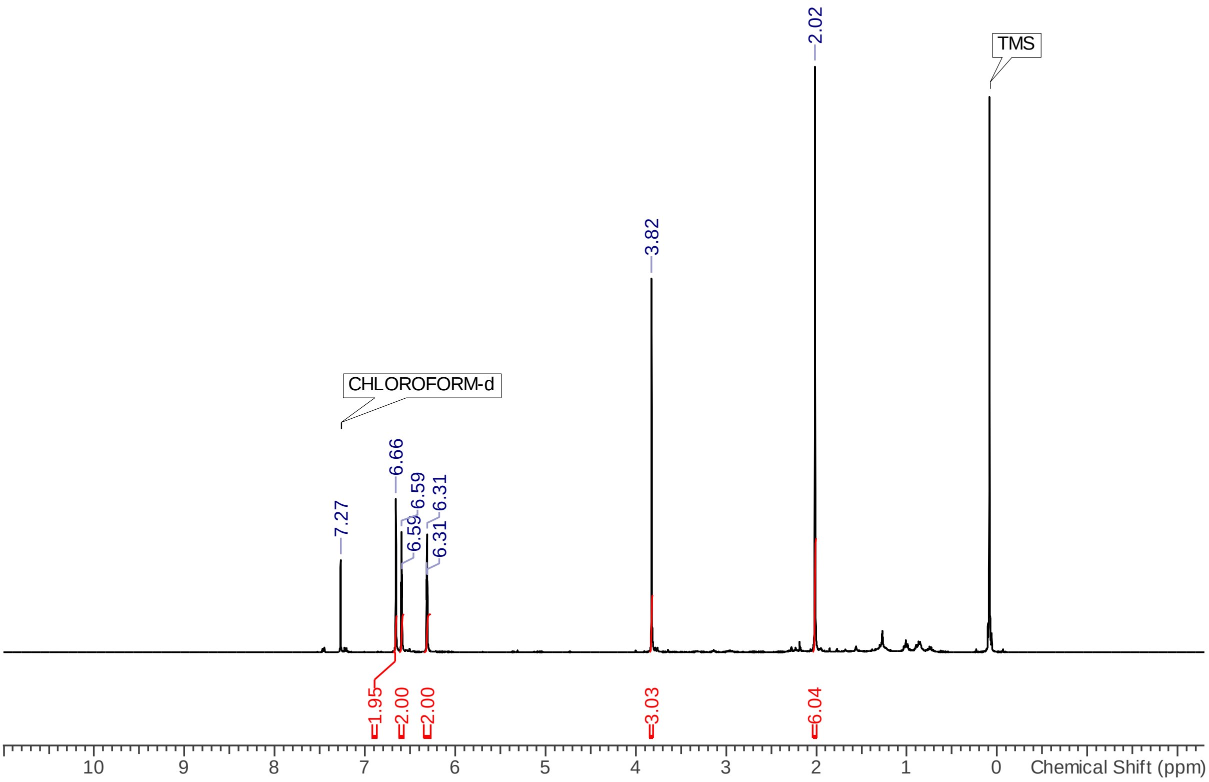

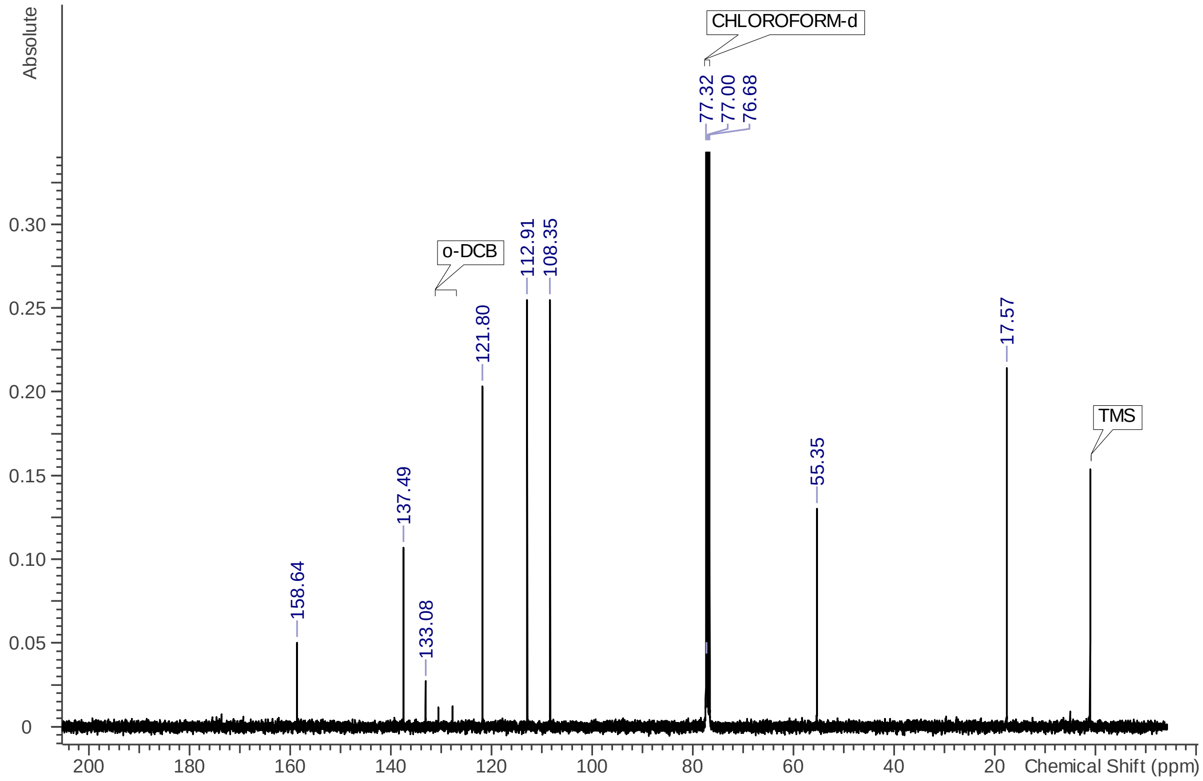

1-Mesityl-3-phenyl-1H-pyrrole 3x

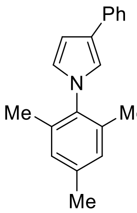

The title compound was prepared according to general procedure 5 using 1-mesityl-3-phenyl-pyrrolidine **1x** (53.0 mg, 0.20 mmol) with an NMR yield of 69%. Purification by flash column chromatography on silica gel (eluent = 20% DCM in pet. ether) gave the title compound **3x** as a pale yellow oil (32.0 mg, 0.13 mmol, 61%). **R**$_f$ = 0.35 (eluent = 20% DCM in pet. ether); **^{1}H NMR** (400 MHz, $CDCl_3$) δ_H = 7.60–7.55 (2H, m), 7.38–7.32 (2H, m), 7.20–7.15 (1H, m), 6.97 (2H, s), 6.93 (1H, app. t, *J* = 2.0 Hz), 6.65 (1H, dd, *J* = 2.8, 2.0 Hz), 6.63 (1H, dd, *J* = 2.8, 2.0 Hz), 2.36 (3H, s), 2.07 (6H, s); **^{13}C NMR** (101 MHz, $CDCl_3$) δ = 137.9 (C), 137.2 (C), 135.8 (2×C), 135.8 (C), 128.7 (2×CH), 128.6 (2×CH), 125.3 (CH), 125.0 (C), 124.9 (2×CH), 122.7 (CH), 118.4 (CH), 106.6 (CH), 21.0 (CH_3), 17.3 (2×CH_3); **HRMS** (ESI): calculated for $[C_{19}H_{20}N]^+$ $(M+H)^+$ m/z: 262.1596; found 262.1596.

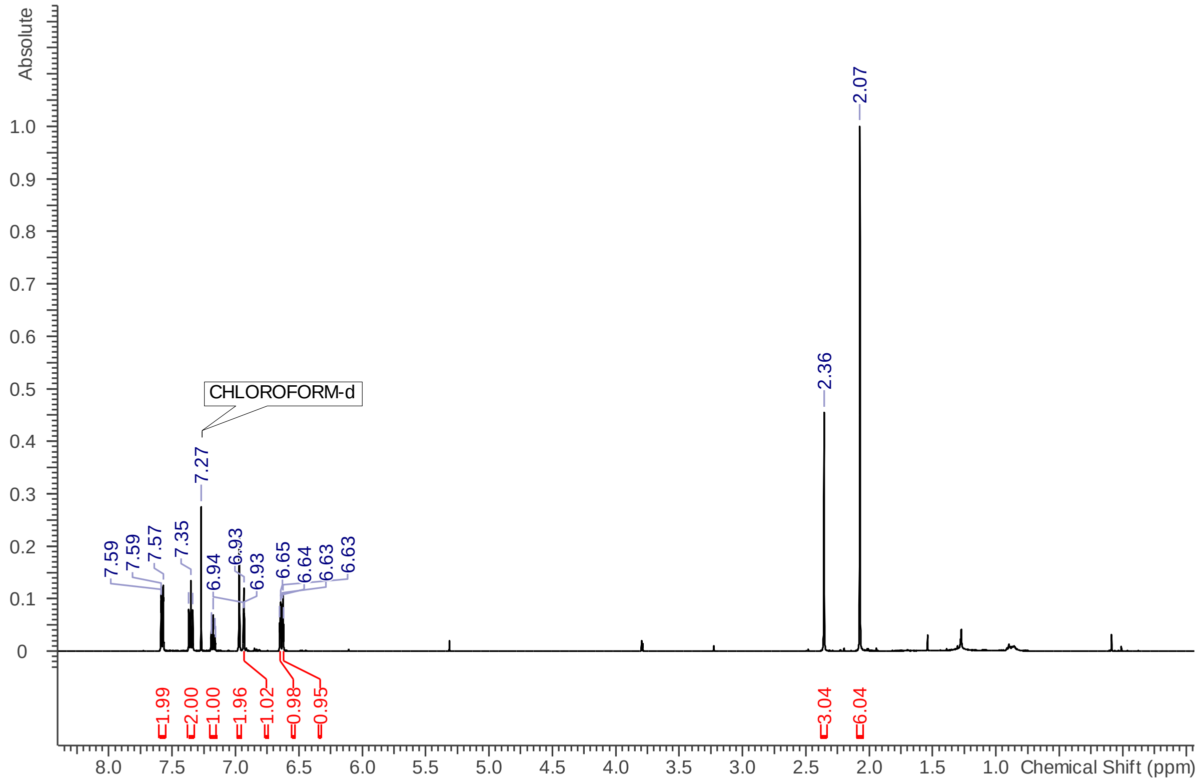

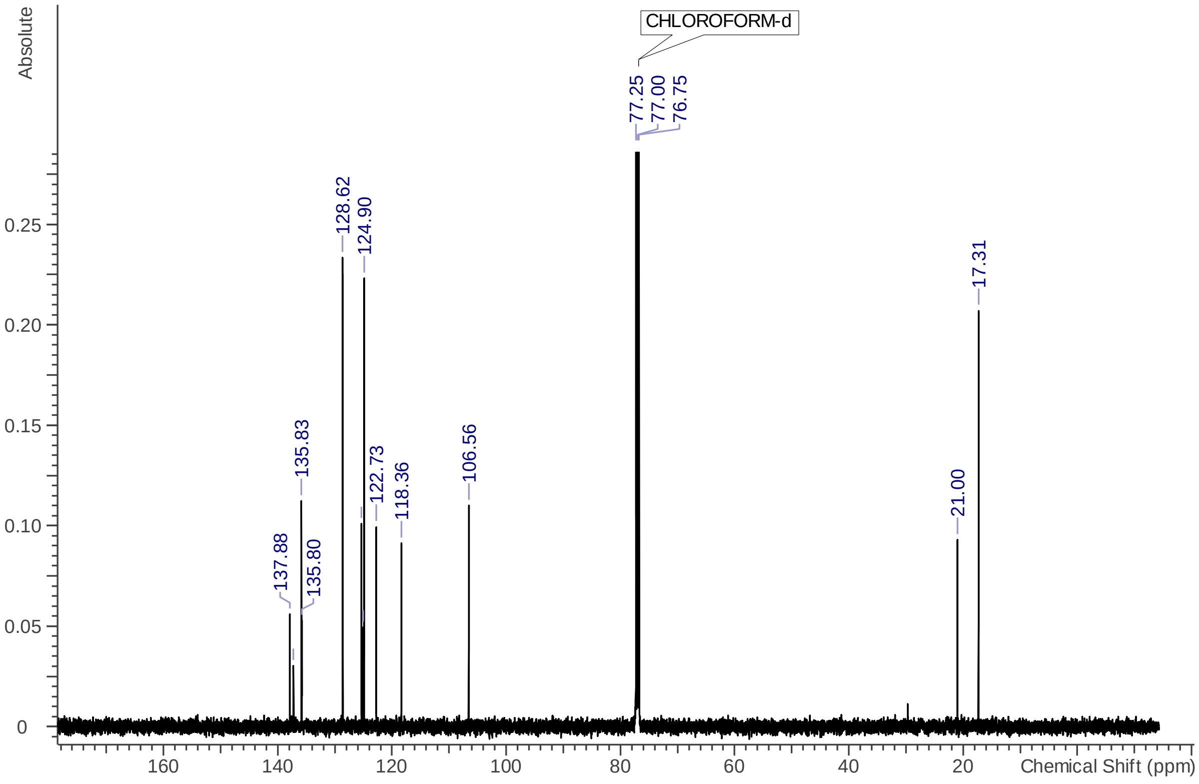

2-Methyl-1-(2,3,5,6-tetrafluorophenyl)pyrrole 3y

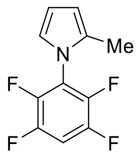

The title compound was prepared according to general procedure 5 using 2-methyl-1-(2,3,5,6-tetrafluorophenyl)pyrrolidine **1y** (47.6 mg, 0.20 mmol) with an NMR yield of 68%. Purification by flash column chromatography on silica gel (eluent = 5% DCM in pet. ether) gave the title compound **3y** as a colourless oil (2.5 mg, 0.01 mmol, 6%). **R$_f$** (5% DCM/petroleum ether) 0.27. **^{1}H NMR** (400 MHz, $CDCl_3$) δ_H = 7.07 (1H, app. tt, $^3J_{H\text{-}F}$ = 9.7 Hz, $^4J_{H\text{-}F}$ = 7.2 Hz), 6.59–6.52 (1H, m), 6.20 (1H, app. t, J = 3.2 Hz), 6.04–5.98 (1H, m), 2.02 (3H, s). **^{13}C NMR** (201 MHz, $CDCl_3$) δ = 146.1 (2×C, dddd, $^1J_{C\text{-}F}$ = 250.6 Hz, $^2J_{C\text{-}F}$ = 13.7 Hz, $^3J_{C\text{-}F}$ = 11.8 Hz, $^4J_{C\text{-}F}$ = 5.0 Hz), 143.2 (2×C, ddd, $^1J_{C\text{-}F}$ = 251.2 Hz, $^2J_{C\text{-}F}$ = 14.3, $^4J_{C\text{-}F}$ = 4.3 Hz), 130.3 (C), 121.8 (CH), 120.4 (C, app. t, $^2J_{C\text{-}F}$ = 14.6), 110.0 (CH), 108.6 (CH), 105.5 (CH, app. t, $^2J_{C\text{-}F}$ = 22.6 Hz), 11.6 (CH_3). **^{19}F NMR** (376 MHz, $CDCl_3$) δ = −137.94-−138.94 (2F, m), −146.78 −−146.95 (2F, m). **HRMS** (ESI): calculated for $[C_{11}H_8F_4N]^+$ $(M+H)^+$ m/z: 230.0587; found 230.0583.

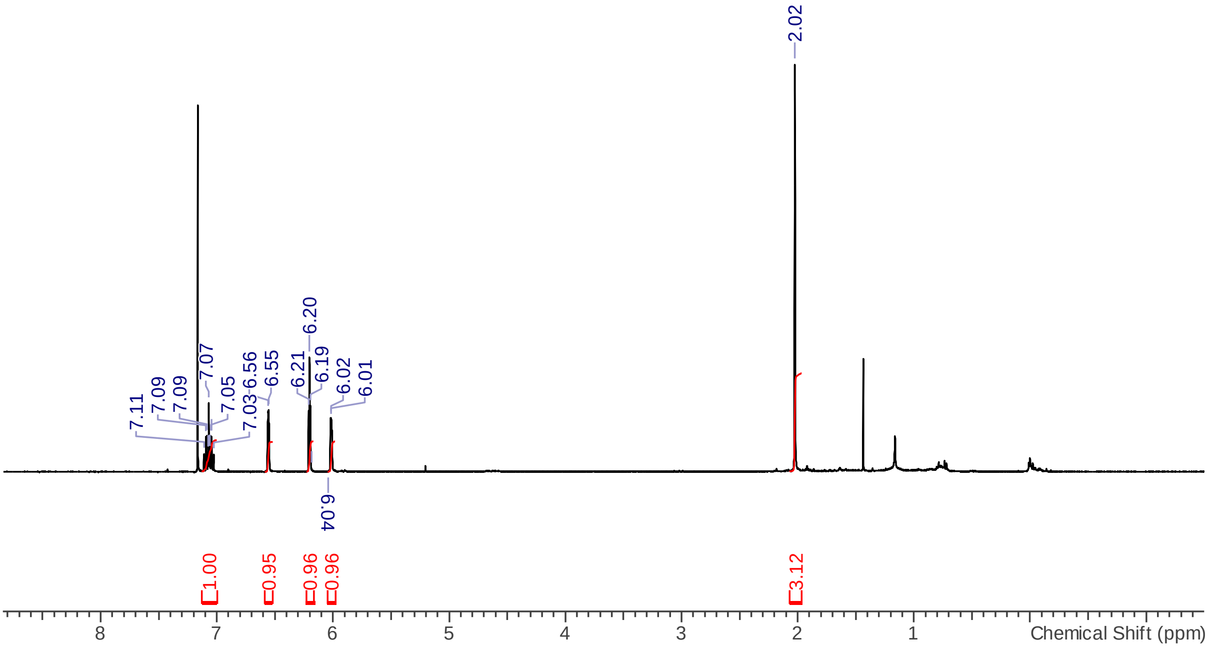

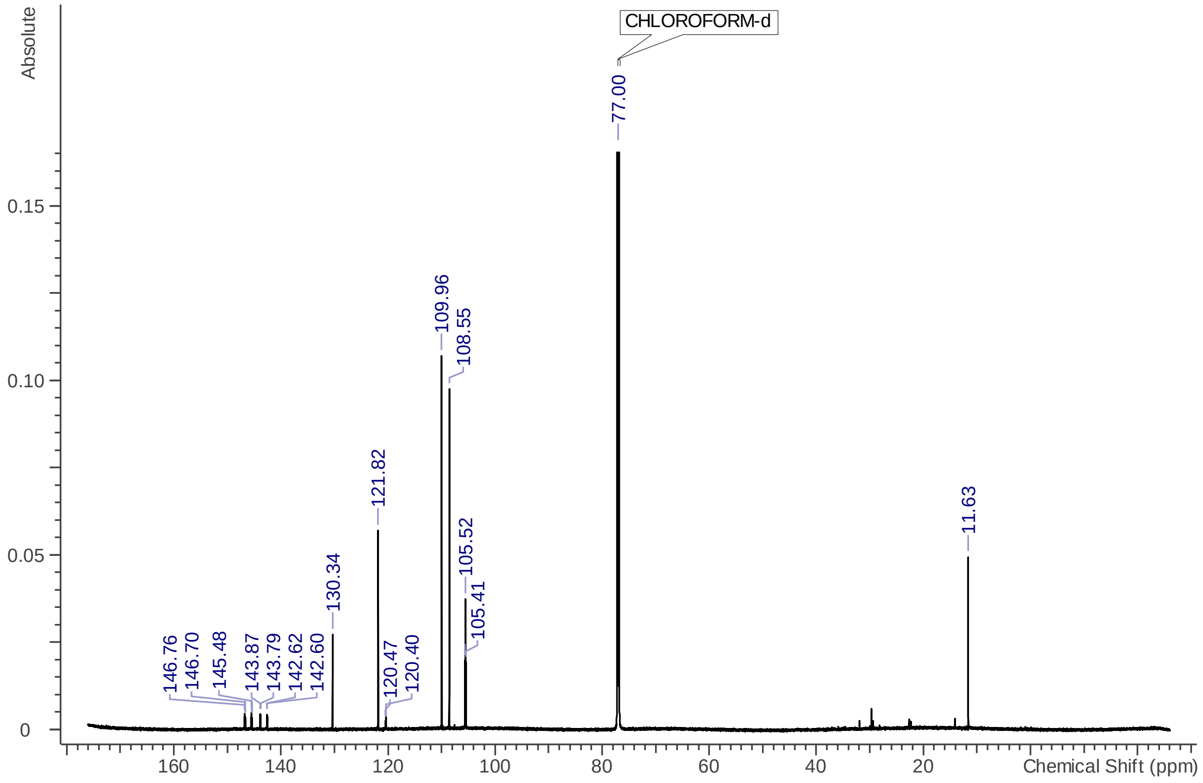

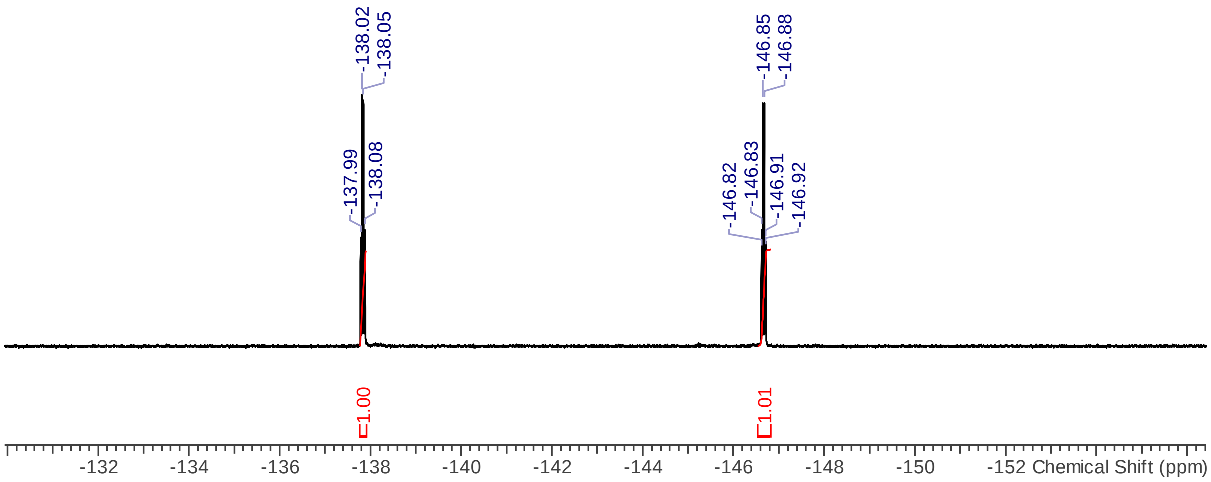
-138.02
-138.05
-137.99
-138.08
-146.85
-146.88
-146.82
-146.83
-146.91
-146.92
1.00
1.01
-132
-134
-136
-138
-140
-142
-144
-146
-148
-150
-152 Chemical Shift (ppm)

1,2-Bis(4-(2-methyl-1*H*-pyrrol-1-yl)phenyl)ethane 3z

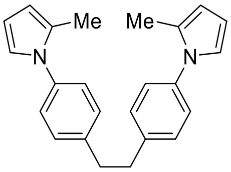

The title compound was prepared according to general procedure 5 (reaction done 5 equiv. of methallyltrimethylsilane **2a** (0.18 mL, 1.0 mmol)) using 2-methyl-1-(3-(4-(2-methylpyrrolidin-1-yl)benzyl)phenyl)pyrrolidine **1z** (69.7 g, 0.20 mmol) with an NMR yield of 52%. Purification by flash column chromatography on silica gel (eluent = 30% DCM in pet. ether) gave the title compound **3z** as a colourless oil (34.8 mg, 0.10 mmol, 51%). **R**$_f$ = 0.4 (eluent = 30% DCM in pet. ether); **^{1}H NMR** (400 MHz, CDCl$_3$) δ_H = 7.31–7.24 (8H, m), 6.82–6.75 (2H, m), 6.23 (2H, app. t, *J* = 3.1 Hz), 6.10–6.04 (2H, m), 3.04 (4H, s), 2.25 (6H, br. s); **^{13}C NMR** (101 MHz, CDCl$_3$) δ_C = 140.2 (2×C), 138.5 (2×C), 129.0 (4×CH), 129.0 (2×C), 125.7 (4×CH), 121.3 (2×CH), 108.0 (2×CH), 107.9 (2×CH), 37.3 (2×CH$_2$), 12.9 (2×CH$_3$); **HRMS** (ESI$^+$): calculated for $[C_{24}H_{25}N_2]^+$ (M+H)$^+$ m/z: 341.2018; found 341.2014.

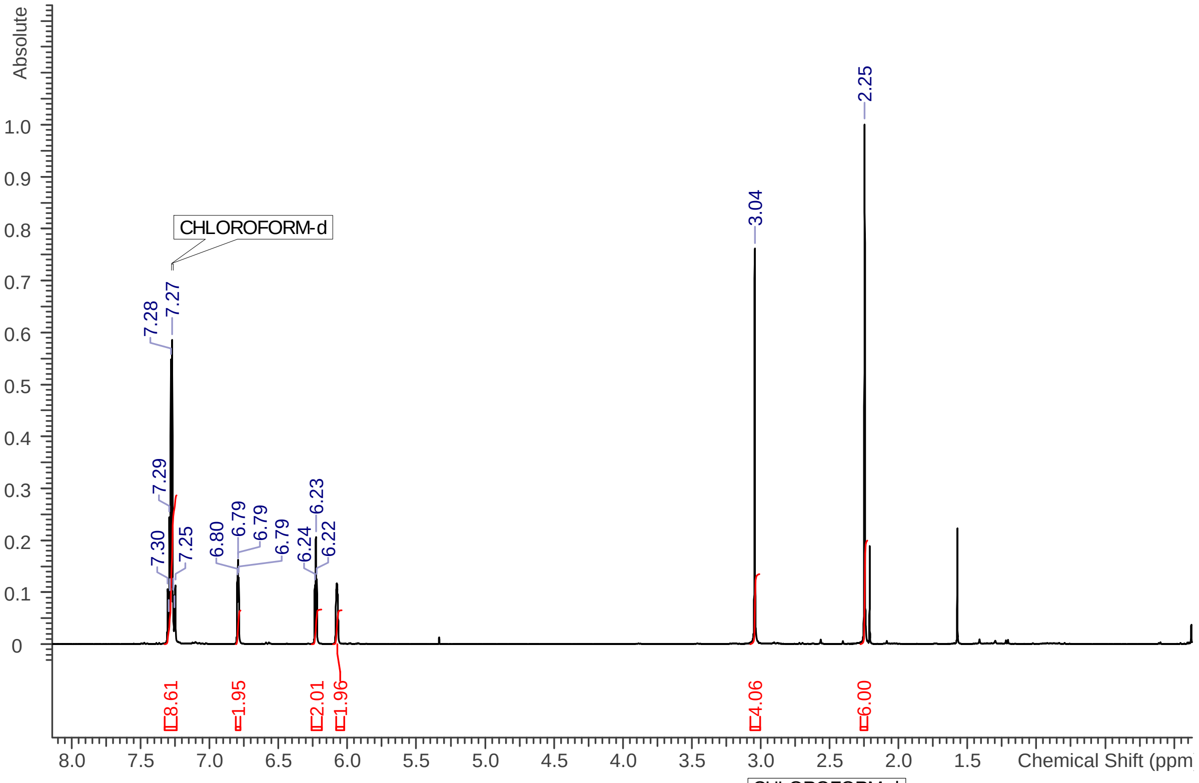

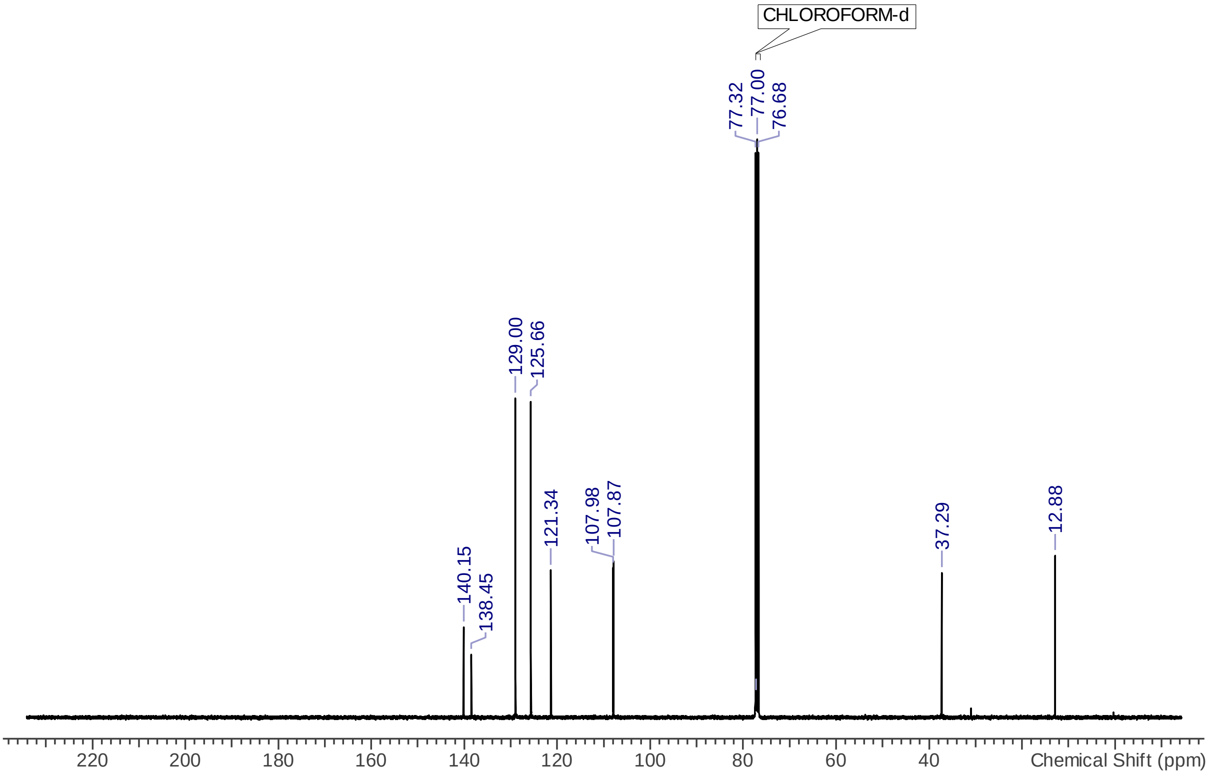

1-(4-Bromonaphthalen-1-yl)-2-methyl-1*H*-pyrrole 3aa

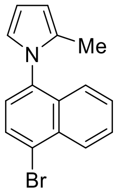

The title compound was prepared according to general procedure 5 using 1-(4-bromonaphthalen-1-yl)-2-methylpyrrolidine **1aa** (57.8 mg, 0.20 mmol) with an NMR yield of 74%. Purification by flash column chromatography on silica gel (eluent = 10% DCM in pet. ether) gave the title compound **3aa** as a light orange oil (40.8 mg, 0.14 mmol, 74%). **R_f** = 0.4 (eluent = 10% DCM in pet. ether); **^{1}H NMR** (400 MHz, CDCl3) δH = 8.3 (1H, app. dt, J = 8.4, 1.0 Hz), 7.86 (1H, d, J = 7.8 Hz), 7.65 (1H, ddd, J = 8.4, 7.0, 1.2), 7.53 (1H, ddd, J = 8.3, 7.0, 1.2 Hz), 7.33–7.28 (2H, m), 6.77 (1H, dd, J = 2.8, 1.8 Hz), 6.30 (1H, dd, J = 3.1, 2.8 Hz), 6.13 (1H, ddq, J = 3.1, 1.8, 0.8 Hz), 1.98 (3H, d, J = 0.8 Hz); **^{13}C NMR** (101 MHz, $CDCl_3$) δC = 137.0 (C), 132.5 (C), 132.4 (C), 130.8 (C), 129.2 (CH), 128.0 (CH), 127.9 (CH), 127.5 (CH), 125.5 (CH), 124.0 (C), 122.9 (C), 122.7 (CH), 108.0 (CH), 107.1 (CH), 12.2 (CH_3); **HRMS** (ESI$^+$): calculated for $[C_{15}H_{13}N_{79}Br]^+$ (M+H)$^+$ m/z: 286.0231; found 286.0237; calculated for $[C_{11}H_{10}N_{81}Br]^+$ (M+H)$^+$ m/z: 288.0211; found 288.0220.

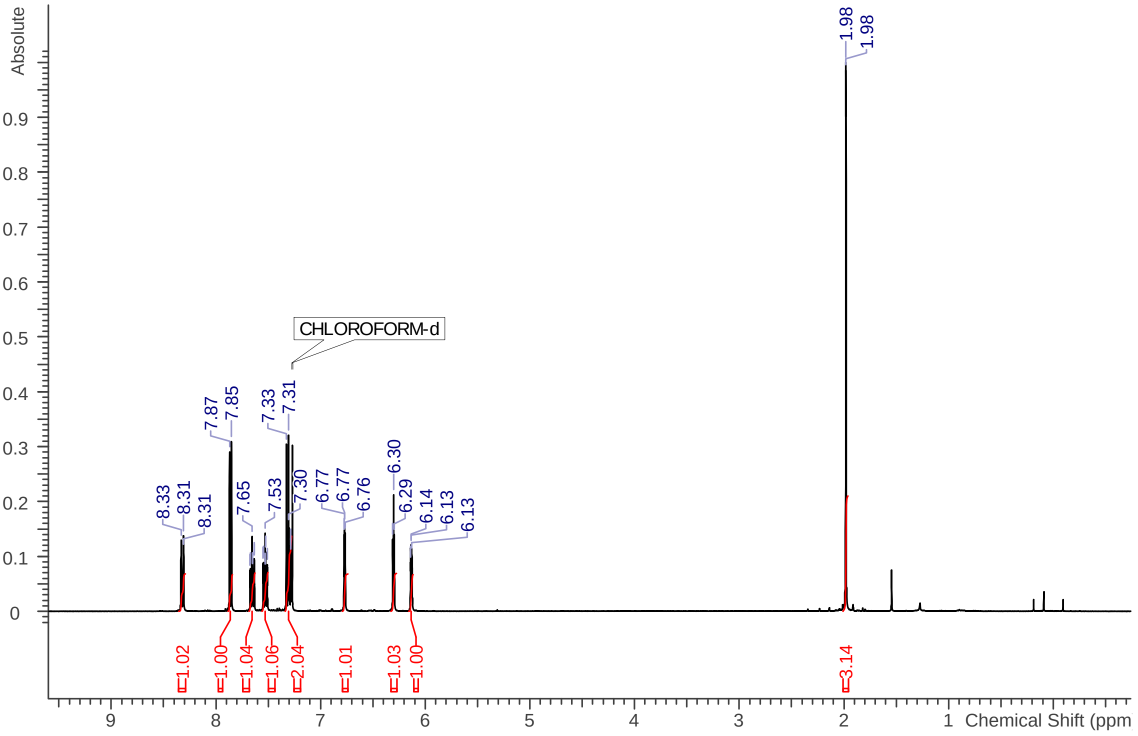

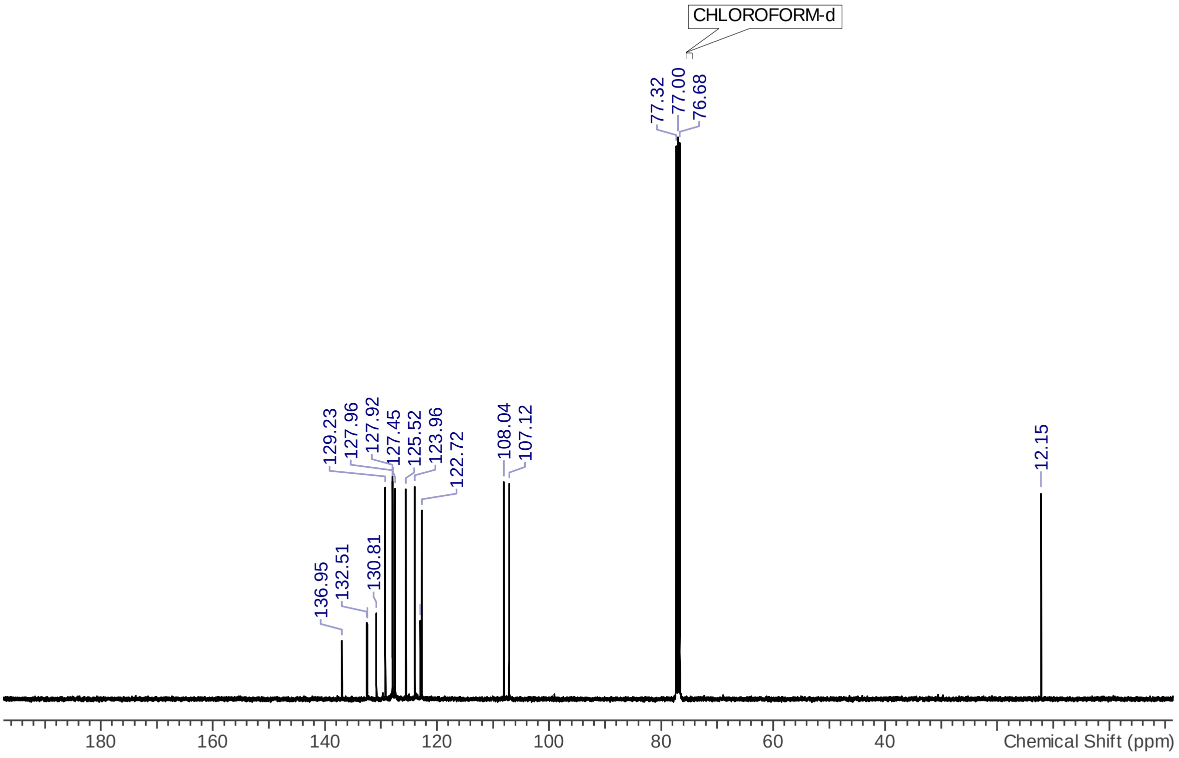

***N-tert*-Butylpyrrole 3ab**

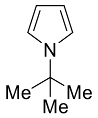

The title compound was prepared according to general procedure 5 (reaction heated to 140 °C for 48 hours rather than 22 hours) using *N-tert*-butylpyrrolidine, **1ab** (41.0 mg, 0.20 mmol), with an NMR yield of 45%. It was unable to be isolated from the reaction mixture due to volatility, so was instead synthesised independently to prove the data matched (see next page).

^{1}H NMR of crude reaction mixture forming **3ab**:

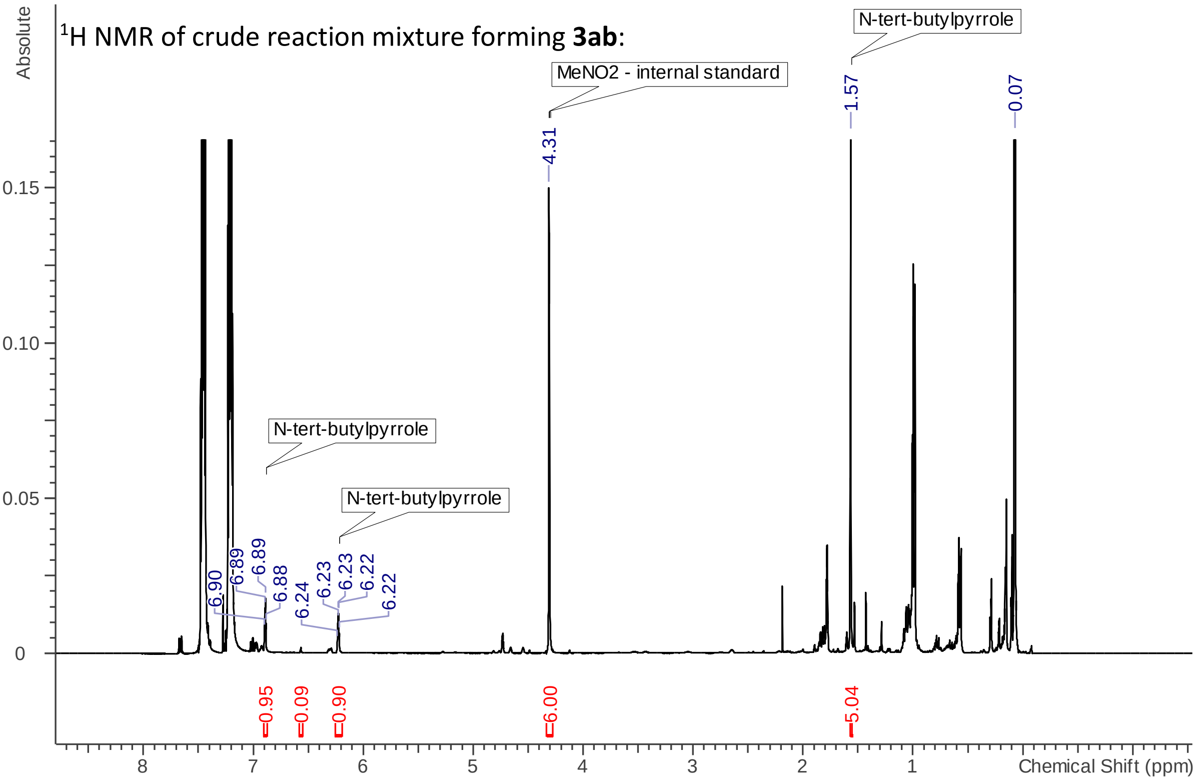

***N-tert*-Butylpyrrole, 3ab**

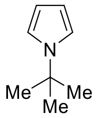

Under N_2, to a solution of 2,5-dimethoxytetrahydrofuran (3.7 mL, 29 mmol) in AcOH (15 mL) was added slowly *t*-butylamine (6.0 mL, 57 mmol). Upon cessation of effervescence, the reaction mixture was heated to 80 °C for 64 h, before being diluted with Et_2O (25 mL), washed with NaOH (aq., 2M, 2×25 mL), H_2O (25 mL) and brine (25 mL), before being dried over $MgSO_4$. Filtration and evaporation gave a crude brown oil (1.5 g). A small amount of the title compound **3ab** (252 mg, 7.2%) was isolated by Kugel Rohr distillation (55 °C, 500 mbar), before other methods of purification were attempted on the rest of the crude to try to find a way to purify a 'real' sample. **^{1}H NMR** (400 MHz, $CDCl_3$) δ_H = 6.86 (2H, t, *J* = 2.2 Hz), 6.18 (2H, t, *J* = 2.2 Hz), 1.56 (9H, s). **^{13}C NMR** (101 MHz, $CDCl_3$) δ = 117.5 (2×CH), 107.4 (2×CH), 54.6 (C), 30.8 (3×CH_3). Spectroscopic data in accordance with that stated in the literature.[16]

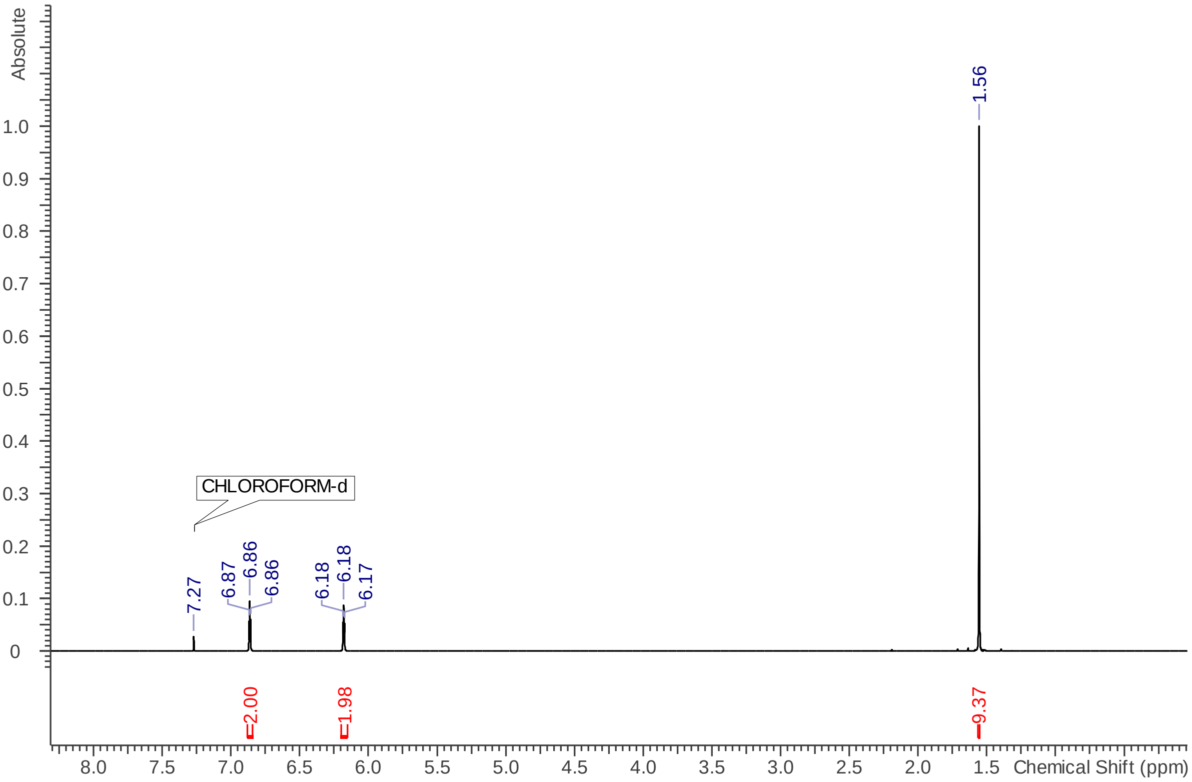

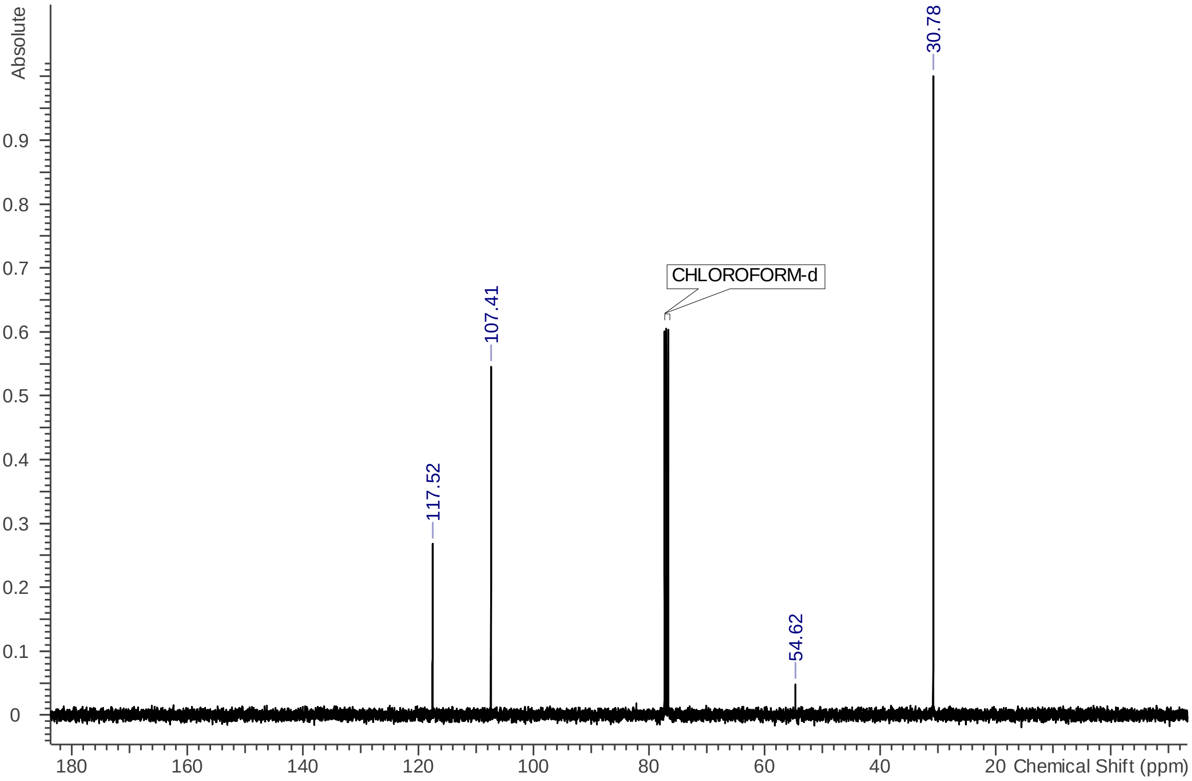

1-Benzyl-2,5-dimethyl-1*H*-pyrrole 3ac

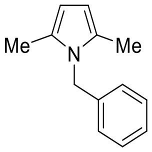

The title compound was prepared according to general procedure 5 using 1-benzyl-2,5-dimethylpyrrolidine **1ac** (37.4 mg, 0.20 mmol) with an NMR yield of 77%. Purification by flash column chromatography on silica gel (20% DCM in pet. ether) gave the title compound **3ac** as a colourless oil (27.6 mg, 0.15 mmol, 75%). **R$_f$** = 0.3 (eluent = 20% DCM in pet. ether); **^{1}H NMR** (400 MHz, $CDCl_3$) δ_H = 7.34–7.29 (2H, m), 7.27–7.21 (1H, m), 6.93–6.88 (2H, m), 5.87 (2H, s), 5.03 (2H, s), 2.16 (6H, s); **^{13}C NMR** (101 MHz, $CDCl_3$) δ_C = 138.5 (C), 128.7 (2×CH), 128.0 (C), 127.0 (CH), 125.6 (2×CH), 105.4 (2×CH), 46.7 (CH_2), 12.4 (2×CH_3); Spectroscopic data in accordance with that stated in the literature.[10]

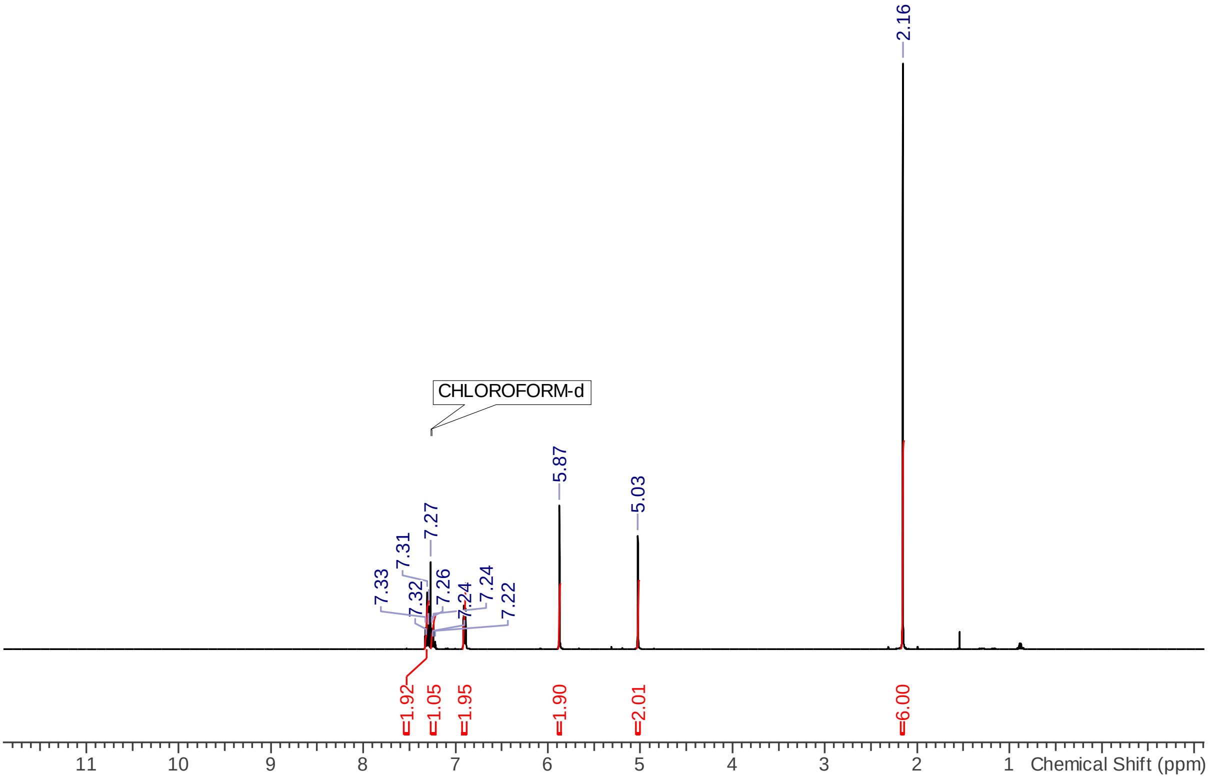

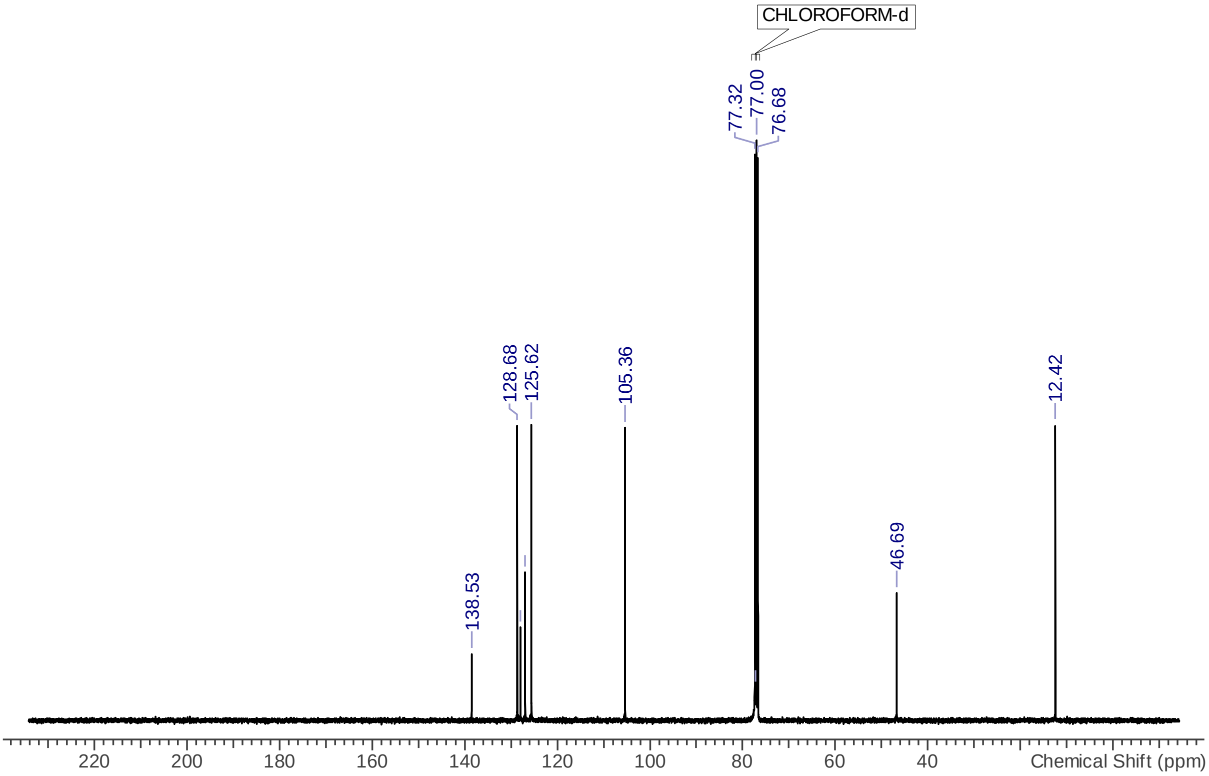

2-Methyl-1-(3-phenylpropyl)-1*H*-pyrrole 3ad

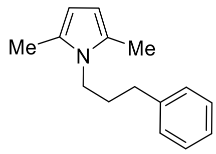

The title compound was prepared according to general procedure 7 using 2,5-dimethyl-1-(3-phenylpropyl)pyrrolidine **1ad** (45.0 mg, 0.20 mmol) with an NMR yield of 57%. Purification by flash column chromatography on silica gel (20% DCM in pet. ether) gave the title compound **3ad** as an orange oil (10.6 mg, 0.05 mmol, 25%). **R**$_f$ = 0.3 (eluent = 20% DCM in pet. ether); ^{1}**H NMR** (400 MHz, $CDCl_3$) δ_H = 7.35–7.28 (2H, m), 7.26–7.19 (3H, m), 5.78 (2H, s), 3.81–3.72 (2H, m), 2.69 (2H, t, *J* = 7.7 Hz), 2.19 (6H, s), 2.05–1.92 (2H, m); ^{13}**C NMR** (101 MHz, $CDCl_3$) δ_C = 141.1 (C), 128.5 (2×CH), 128.2 (2×CH), 127.3 (C), 126.1 (CH), 105.1 (2×CH), 43.1 (CH_2), 33.1 (CH_2), 32.2 (CH_2), 12.4 (2×CH_3); **HRMS** (ESI$^+$): calculated for $[C_{15}H_{20}N]^+$ $(M+H)^+$ m/z: 214.1596; found 214.1595.

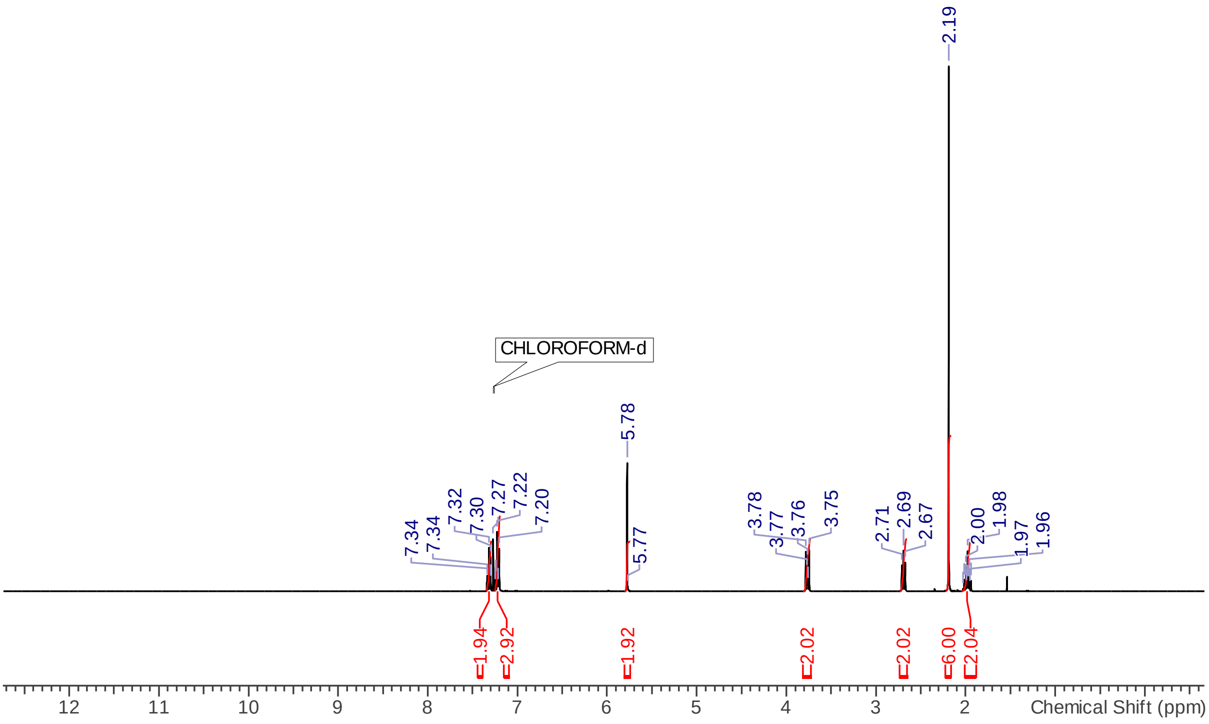

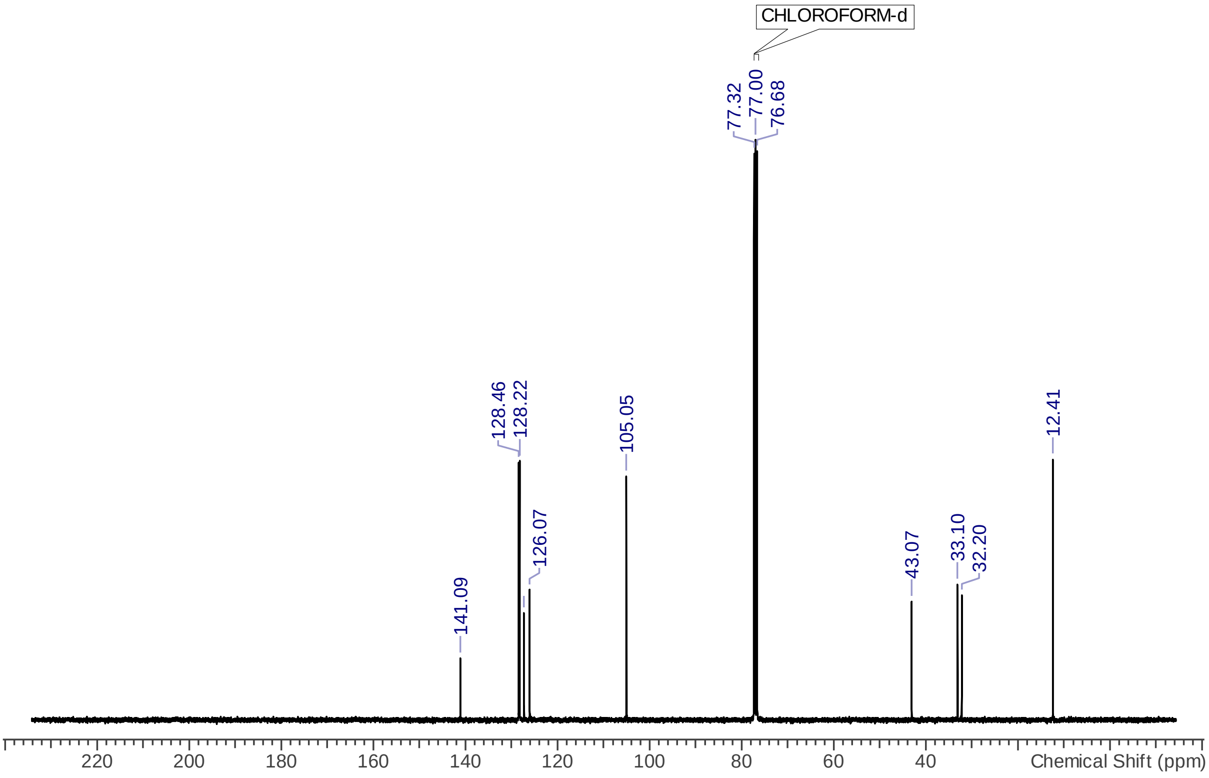

2,5-Dimethyl-1-(thiophen-2-ylmethyl)-1*H*-pyrrole 3ae

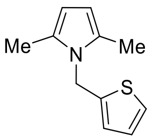

The title compound was prepared according to general procedure 5 using 2,5-dimethyl-1-(3-phenylpropyl)pyrrolidine **1ae** (38.2 mg, 0.20 mmol) with an NMR yield of 56%. Purification by flash column chromatography on silica gel (20% 4CM in pet. ether) gave the title compound **3ae** as a colourless oil (19.9 mg, 0.11 mmol, 52%). **R$_f$** = 0.3 (eluent = 40% DCM in pet. ether); **^{1}H NMR** (400 MHz, $CDCl_3$) δ_H = 7.19 (1H, dd, *J* = 5.1, 1.2 Hz), 6.92 (1H, dd, *J* = 5.1, 3.5 Hz), 6.70 (1H, ddt, *J* = 3.5, 1.2, 0.9 Hz), 5.83 (2H, s), 5.12 (2H, d, *J* = 0.9 Hz), 2.24 (6H, s); **^{13}C NMR** (101 MHz, $CDCl_3$) δ_C = 141.7 (C), 127.6 (C), 126.9 (CH), 124.6 (CH), 124.2 (CH), 105.7 (2×CH), 42.5 (CH_2), 12.4 (2×CH_3); **HRMS** (ESI$^+$): calculated for $[C_{11}H_{14}NS]^+$ (M+H)$^+$ m/z: 192.0847; found 192.0844.

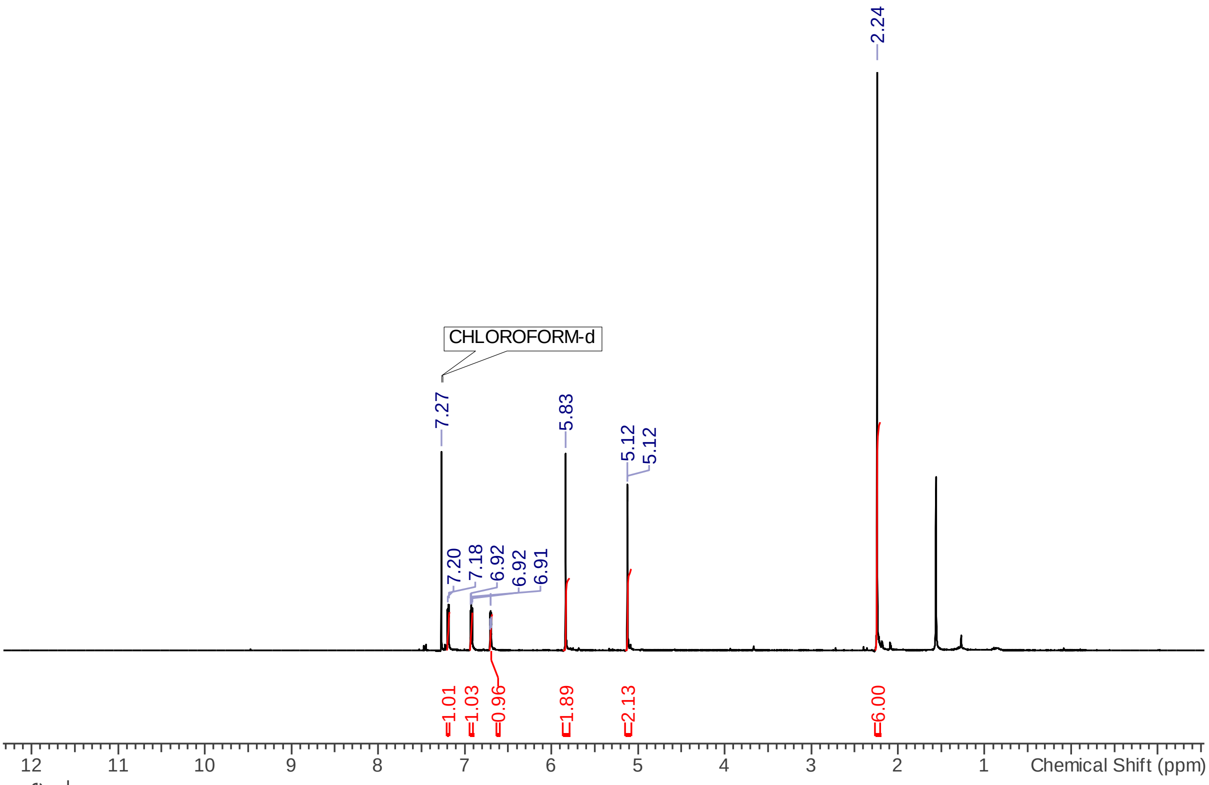

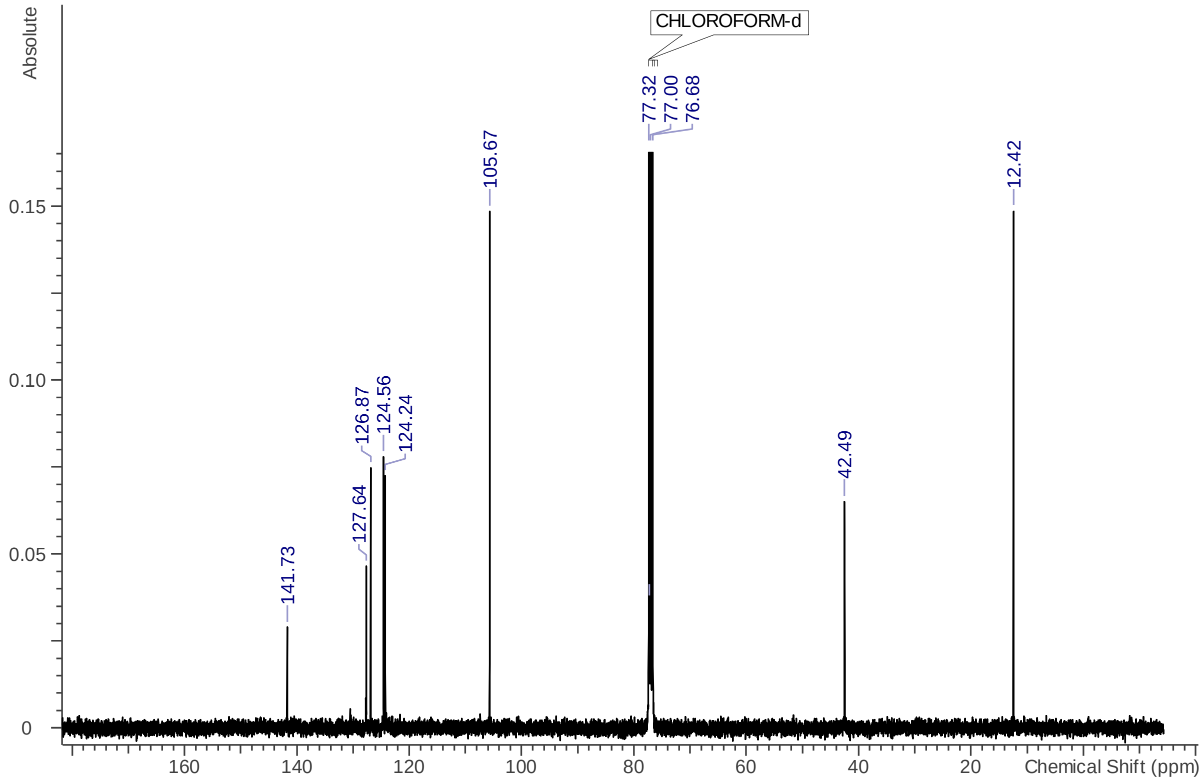

1-(1-Phenylcyclohexyl)pyrrole (tetradehydro-rolicyclidine) 3af

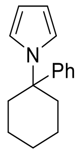

The title compound was prepared according to general procedure 4b using **1af** (45.9 mg, 0.20 mmol), $H_2O.B(C_6F_5)_3$ (42.8 mg, 0.08 mmol, 40 mol%), Et_3SiH (25.6 µL, 0.16mmol, 80 mol%), and methallyltrimethylsilane (0.18 mL, 1.0 mmol) with an NMR yield of 58%. Purification by flash column chromatography on silica gel (eluent = 5% DCM in pet. ether) gave the title compound **3af** as a white solid (17.9 mg, 0.08 mmol, 40%). **Rf** = 0.19 (eluent = 10% DCM in pet. ether); **^{1}H NMR** (400 MHz, $CDCl_3$) δ_H = 7.28–7.23 (2H, m), 7.21–7.16 (1H, m), 6.88 (2H, t, *J* = 2.2 Hz), 6.86–6.82 (2H, m), 6.26 (2H, t, *J* = 2.2 Hz), 2.46 (2H, br. d, *J* = 14.3 Hz), 2.20 (2H, ddd, *J* = 14.3, 11.7, 3.6 Hz), 1.75–1.56 (5H, m), 1.50–1.37 (1H, m); **^{13}C NMR** (101 MHz, $CDCl_3$) δ_C = 148.5 (C), 128.4 (2×CH), 126.8 (CH), 124.9 (2×CH), 119.2 (2×CH), 107.9 (2×CH), 63.2 (C), 37.5 (2×CH_2), 25.2 (CH_2), 22.1 (2×CH_2); **HRMS** (ESI$^+$): calculated for $[C_{16}H_{20}N]^+$ (M+H)$^+$ m/z: 226.1596; found 226.1600.

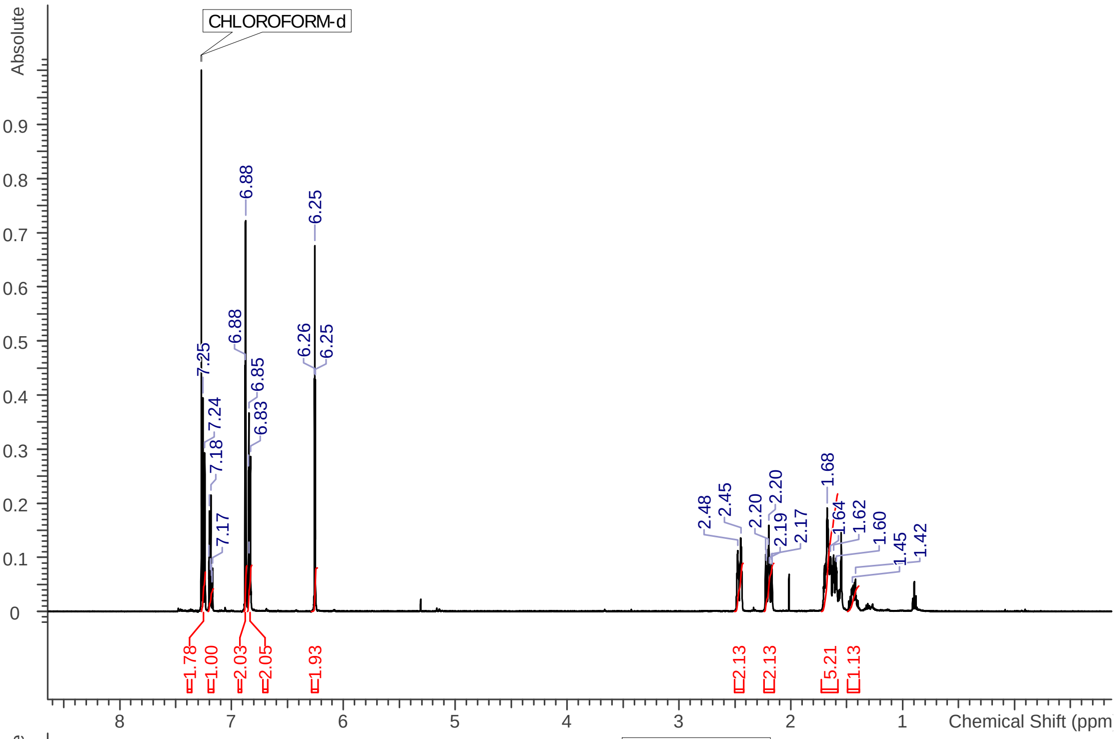

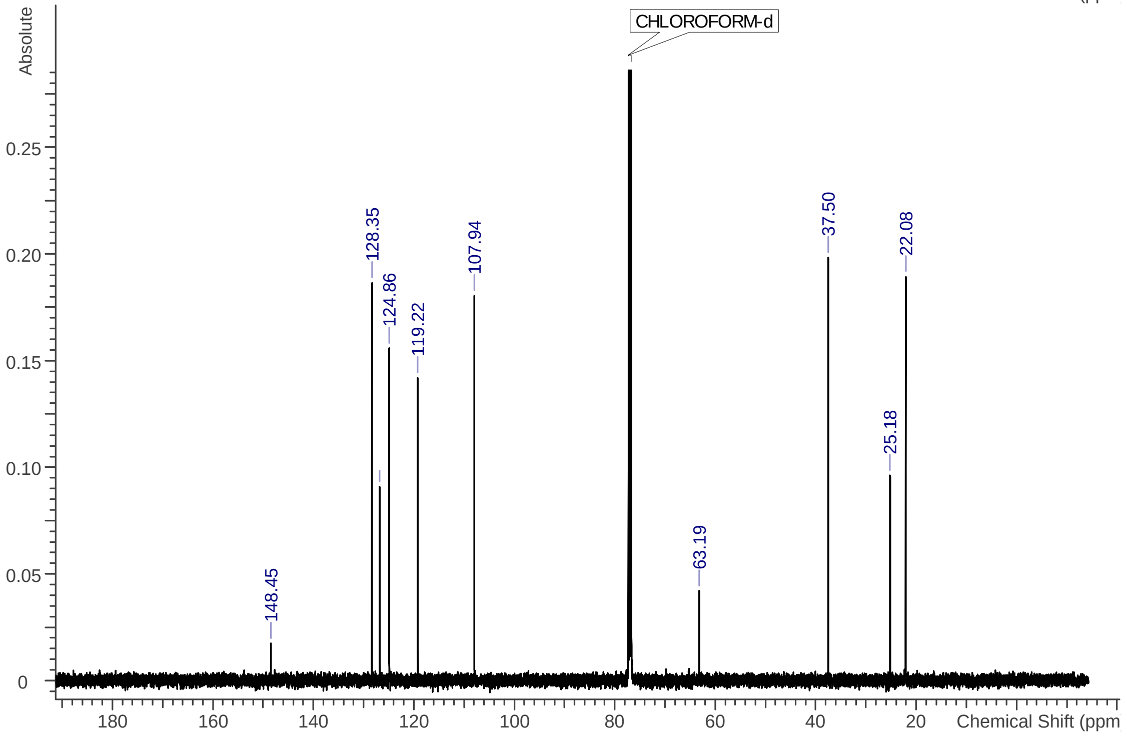

1-(3-Methoxy-14-methyl-7,8,9,11,12,13,14,15,16,17-decahydro-6H-cyclopenta[a]phenanthren-15-yl)pyrrole 3ag

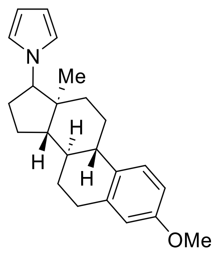

The title compound was prepared according to general procedure 4b using 1-(3-methoxy-14-methyl-7,8,9,11,12,13,14,15,16,17-decahydro-6*H*-cyclopenta[a]phenanthren-15-yl)pyrrolidine **1ag** (67.9 mg, 0.20 mmol), $H_2O.B(C_6F_5)_3$ (42.4 mg, 0.08 mmol), Et_3SiH (25.4 µL, 0.16mmol) and methallyltrimethylsilane (0.18 mL, 1.0 mmol) with an NMR yield of 71% (89:11 mixture of diastereomers). Purification by flash column chromatography on silica gel (eluent = 30% DCM in pet. ether) gave the title compound **X** as a white solid (24.4 mg, 0.07 mmol, 36%, 91:9 mixture of diastereomers). **Rf** = 0.21 (eluent = 30% DCM in pet. ether); **^{1}H NMR** (500 MHz, $CDCl_3$) **Common signals:** δ_H = 7.22 (1H, d, *J* = 8.6 Hz), 6.73 (1H, dd, *J* = 8.6, 2.8 Hz), 6.56 (1H, br. d, *J* = 2.8 Hz), 6.20–6.15 (2H, m), 3.80 (3H, s), 2.97–2.83 (2H, m), 2.40–2.18 (4H, m), 1.99–1.81 (3H, m), 1.56–1.36 (6H, m); **Major diastereomer:** δ_H = 6.79 (2H, t, *J* = 2.1 Hz), 4.02 (1H, t, *J* = 9.8 Hz), 0.60 (3H, s); **Minor diastereomer:** δ_H = 6.76 (2H, t, *J* = 2.1 Hz), 3.53 (1H, d, *J* = 9.5 Hz), 0.65 (3H, s); **^{13}C NMR** (126 MHz, $CDCl_3$) δ_C = 157.5 (C)†, 137.8 (C)†, 132.44 (C)*, 132.40 (C), 126.3 (CH), 126.2 (CH)*, 120.2 (2×CH)*, 120.1 (2×CH) 113.8 (CH)†, 111.5 (CH)†, 107.1 (2×CH)†, 78.4 (CH)*, 69.9 (CH), 55.2 (CH_3)†, 51.7 (CH), 50.1 (CH)*, 45.6 (C)*, 44.5 (C), 44.02 (CH)*, 43.95 (CH), 38.9 (CH), 38.7 (CH)*, 37.2 (CH_2), 33.7 (CH_2)*, 31.9 (CH_2)*, 29.8 (CH_2), 27.34 (CH_2), 27.31 (CH_2)*, 26.4 (CH_2), 26.2 (CH_2)*, 26.0 (CH_2)†, 23.0 (CH_2), 20.5 (CH_2)*, 12.9 (CH_3)*, 12.2 (CH_3); **HRMS** (ESI$^+$): calculated for $[C_{23}H_{30}NO]^+$ (M+H)$^+$ m/z: 336.2327; found 336.2325. **Signals attributed to minor diastereomer. †Signals attributed to both major and minor diastereomers.*

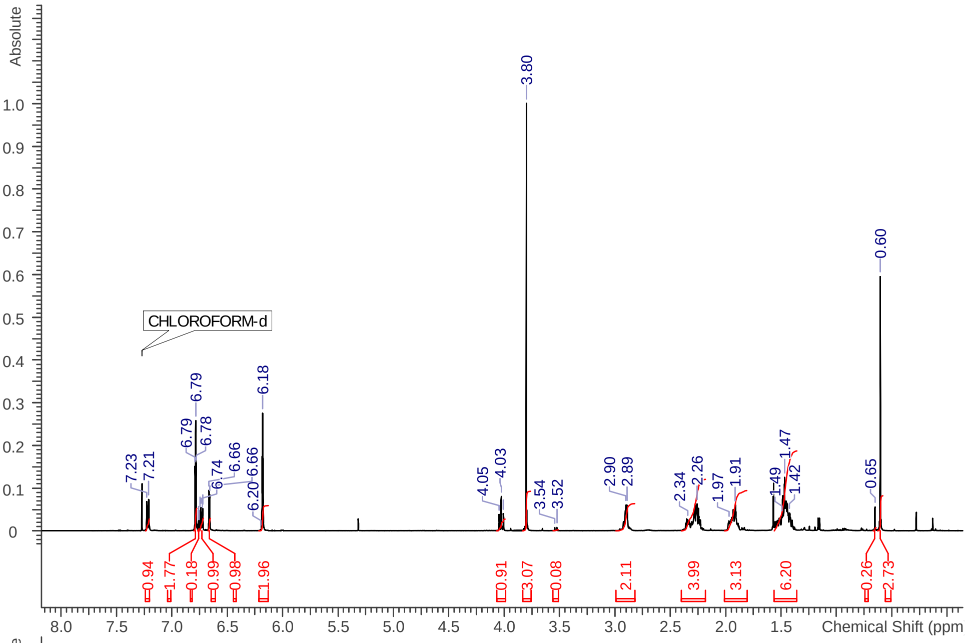

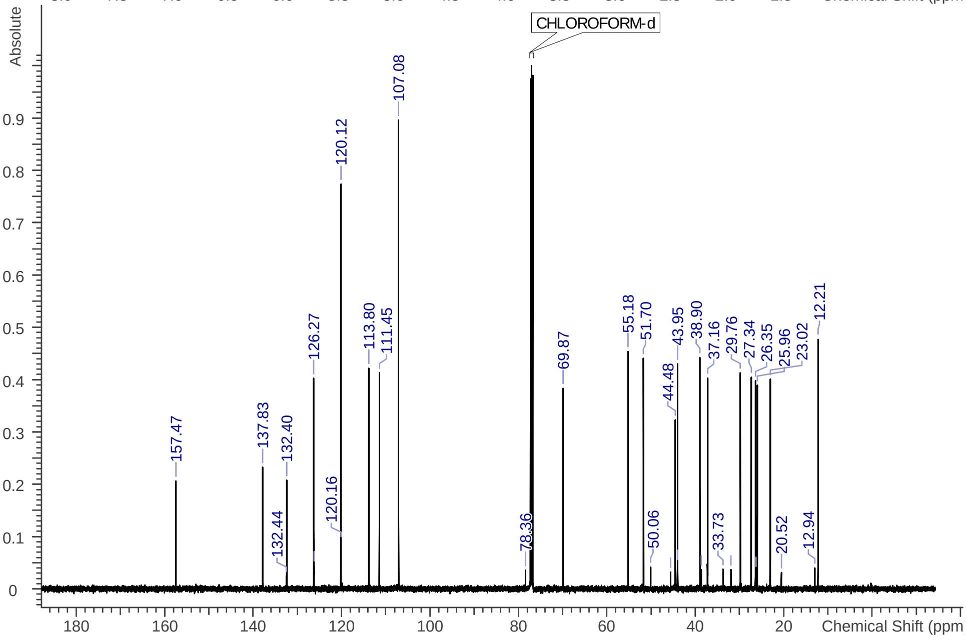

1-(4-(4-Chloro-3,5-dimethylphenoxy)phenyl)-2-methylpyrrole 3ah

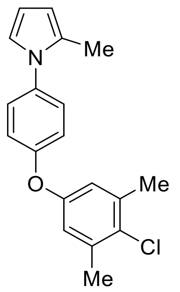

The title compound was prepared according to general procedure 4b using 1-(4-(4-chloro-3,5-dimethylphenoxy)phenyl)-2-methylpyrrolidine **1ah** (63.2 mg, 0.20 mmol), $H_2O.B(C_6F_5)_3$ (21.2 mg, 0.04 mmol), and Et_3SiH (12.7 µL, 0.08mmol) with an NMR yield of 70%. Purification by flash column chromatography on silica gel (eluent = 7.5% DCM in pet. ether) gave the title compound **3ah** as a colourless oil (25.9 mg, 0.08 mmol, 42%). **Rf** = 0.36 (eluent = 10% DCM in pet. ether); **^{1}H NMR** (500 MHz, $CDCl_3$) δ_H = 7.28–7.23 (2H, m), 7.05–7.01 (2H, m), 6.82 (2H, br. s), 6.76 (1H, dd, *J* = 2.8, 2.0 Hz), 6.20 (1H, dd, *J* = 3.3, 2.8 Hz), 6.05 (1H, ddq, *J* = 3.3, 2.0, 0.9 Hz), 2.39 (6H, br.s), 2.22 (3H, d, *J* = 0.9 Hz); **^{13}C NMR** (126 MHz, $CDCl_3$) δ_C = 156.3 (C), 154.3 (C), 137.9 (2×C), 135.5 (C), 129.5 (C), 129.2 (C), 127.1 (2×CH), 121.4 (CH), 119.2 (2×CH), 118.6 (2×CH), 107.91 (CH), 107.90 (CH), 20.9 (2×CH_3), 12.8 (CH_3); **HRMS** (ESI$^+$): calculated for $[C_{19}H_{19}NO^{35}Cl]^+$ (M+H)$^+$ m/z: 312.1155; found 312.1158; calculated for $[C_{19}H_{19}NO^{37}Cl]^+$ (M+H)$^+$ m/z: 314.1126; found 314.1132.

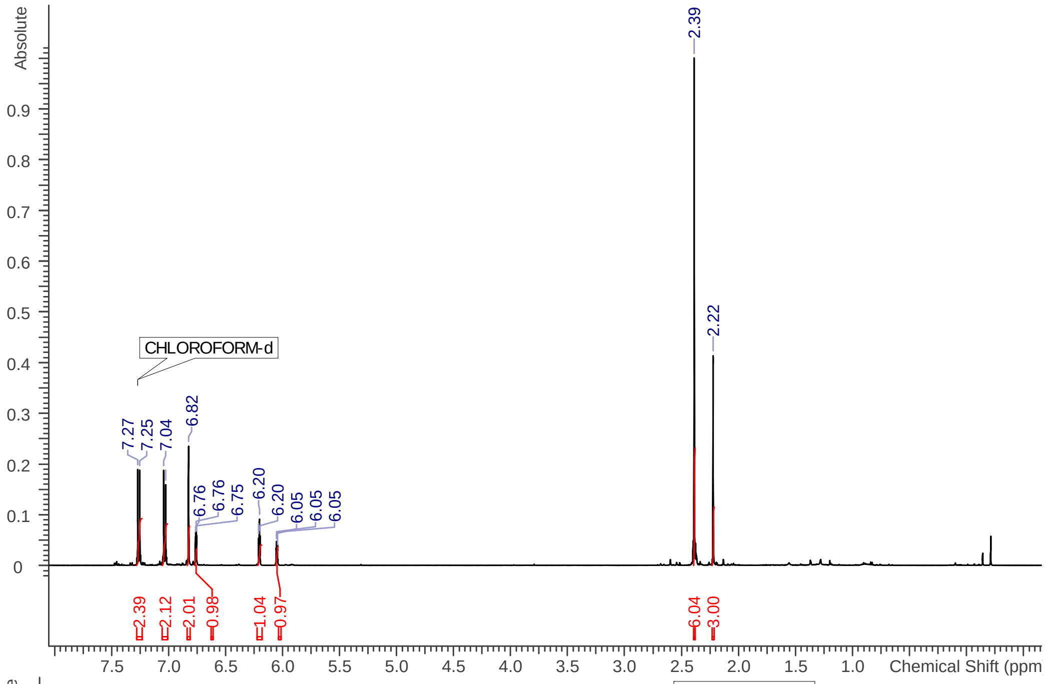

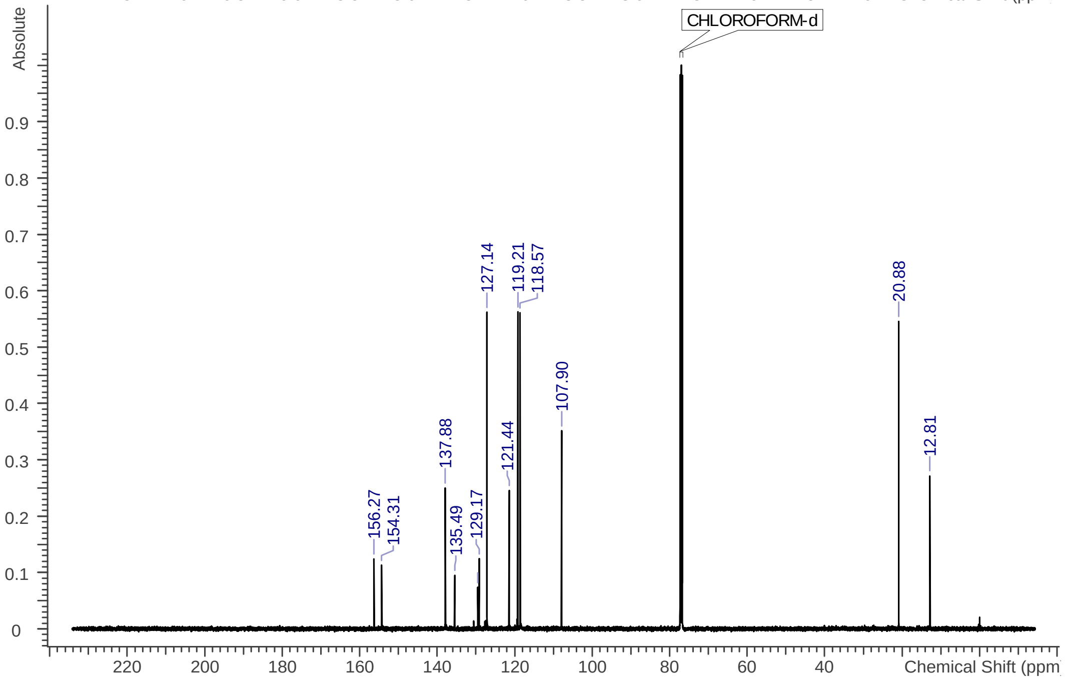

Optimization of 3af, 3ag and 3ah

Table S2. Optimization of **3af.**

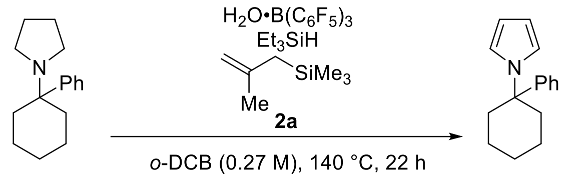

1af **3af**

Entry[a]	$B(C_6F_5)_3$•H_2O (mol%)	Et_3SiH (mol%)	**2a** (equiv.)	Yield **3af** (%)[b]
1	20	40	2.5	19
2	40	80	2.5	29
3	40	80	5	58
4	40	40	2.5	41
5	40	60	2.5	52

[a] Reactions performed using 0.2 mmol of **1af**, following general procedure 5. [b] Yields were determined by ^{1}H NMR analysis of the crude reaction mixture with nitromethane as the internal standard.

Table S3. Optimization of **3ag**.

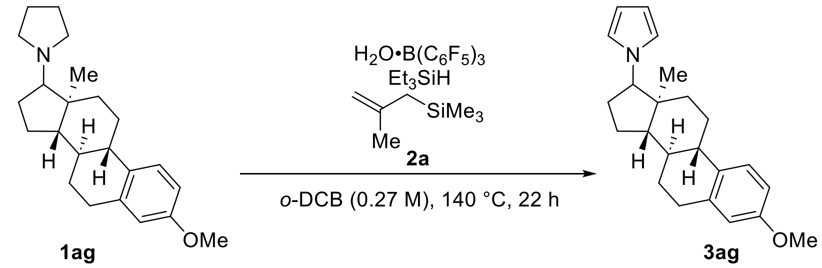

1ag **3ag**

Entry[a]	$B(C_6F_5)_3$•H_2O (mol%)	Et_3SiH (mol%)	**2a** (equiv.)	Yield **3ag** (%)[b]
1	20	40	2.5	31
2	40	80	2.5	58
3	40	80	5	71

[a] Reactions performed using 0.2 mmol of **1ag**, following general procedure 5. [b] Yields were determined by ^{1}H NMR analysis of the crude reaction mixture with nitromethane as the internal standard.

Table S4. Optimization of **3ah**

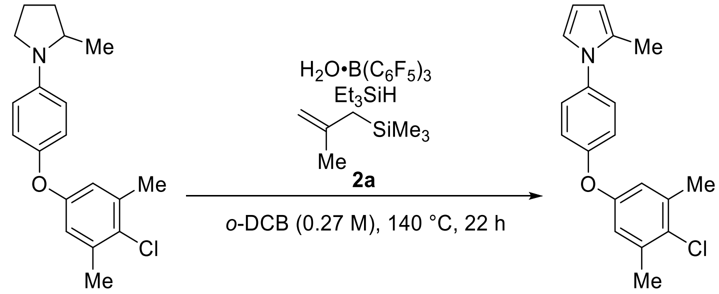

1ah **3ah**

Entry[a]	$B(C_6F_5)_3$•H_2O (mol%)	Et_3SiH (mol%)	**2a** (equiv.)	Yield **3ah** (%)[b]
1	20	40	2.5	70
2	40	80	2.5	41
3	40	80	5	23

[a] Reactions performed using 0.2 mmol of **1ah**, following general procedure 5. [b] Yields were determined by ^{1}H NMR analysis of the crude reaction mixture with nitromethane as the internal standard.

Dehydrogenation of indolines
1-Methyl-1*H*-indole 5a

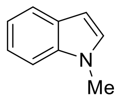

The title compound was prepared according to general procedure 5 (reaction using 5 mol% $B(C_6F_5)_3$, 10 mol% Et_3SiH heated to 40°C) using 1-methylindoline **4a** (26.6 mg, 0.20 mmol) with an NMR yield of 84%. Purification by flash column chromatography on silica gel (10% DCM in pet. ether) gave the title compound **5a** as a colourless oil (20.6 mg, 0.16 mmol, 79%). **R$_f$** = 0.47 (eluent = 20% DCM in pet. ether); **^{1}H NMR** (400 MHz, $CDCl_3$) δ_H = 7.66 (1H, d, *J* = 7.9 Hz), 7.35 (d, *J* = 8.2 Hz, 1H, ArH), 7.27–7.23 (1H, m), 7.16–7.10 (1H, m), 7.07 (1H, d, *J* = 3.1 Hz), 6.51 (1H, d, *J* = 3.1 Hz), 3.82 (3H, s); **^{13}C NMR** (101 MHz, $CDCl_3$) δ_C = 136.7 (C), 128.7 (CH), 128.4 (C), 121.4 (CH), 120.8 (CH), 119.2 (CH), 109.1 (CH), 100.9 (CH), 32.8 (CH_3). Spectroscopic data in accordance with that stated in the literature.[15]

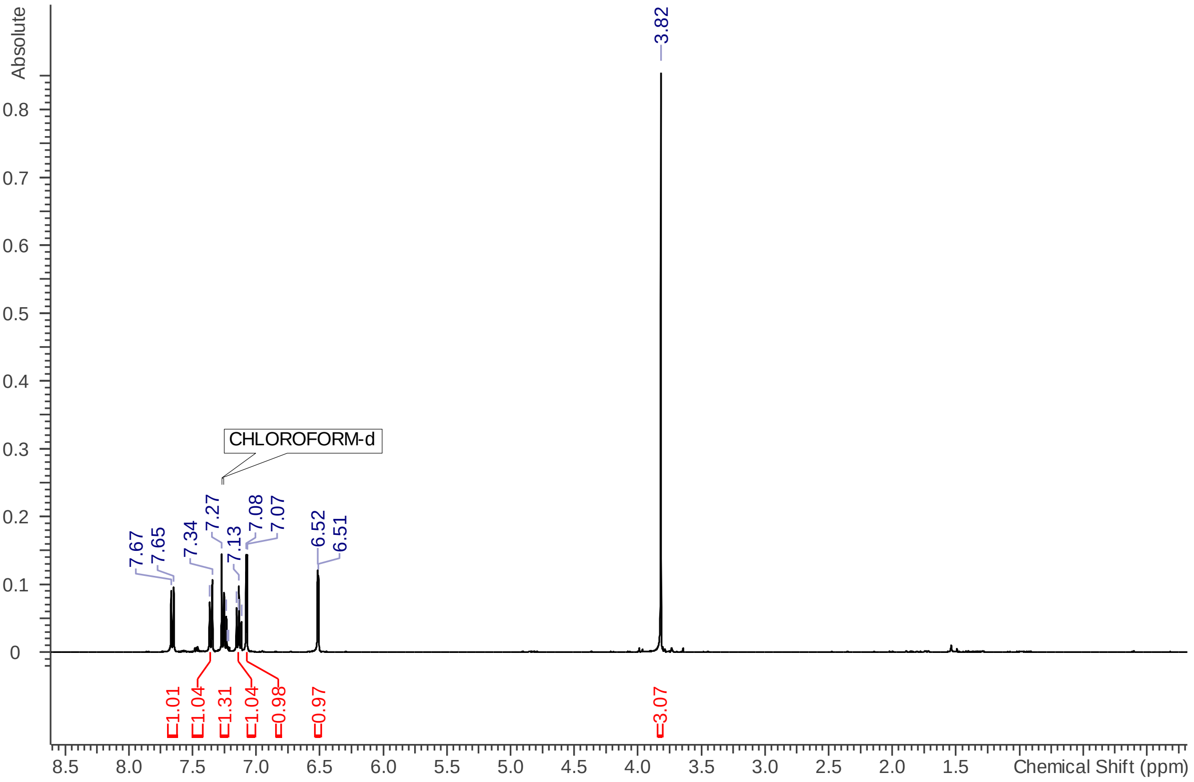

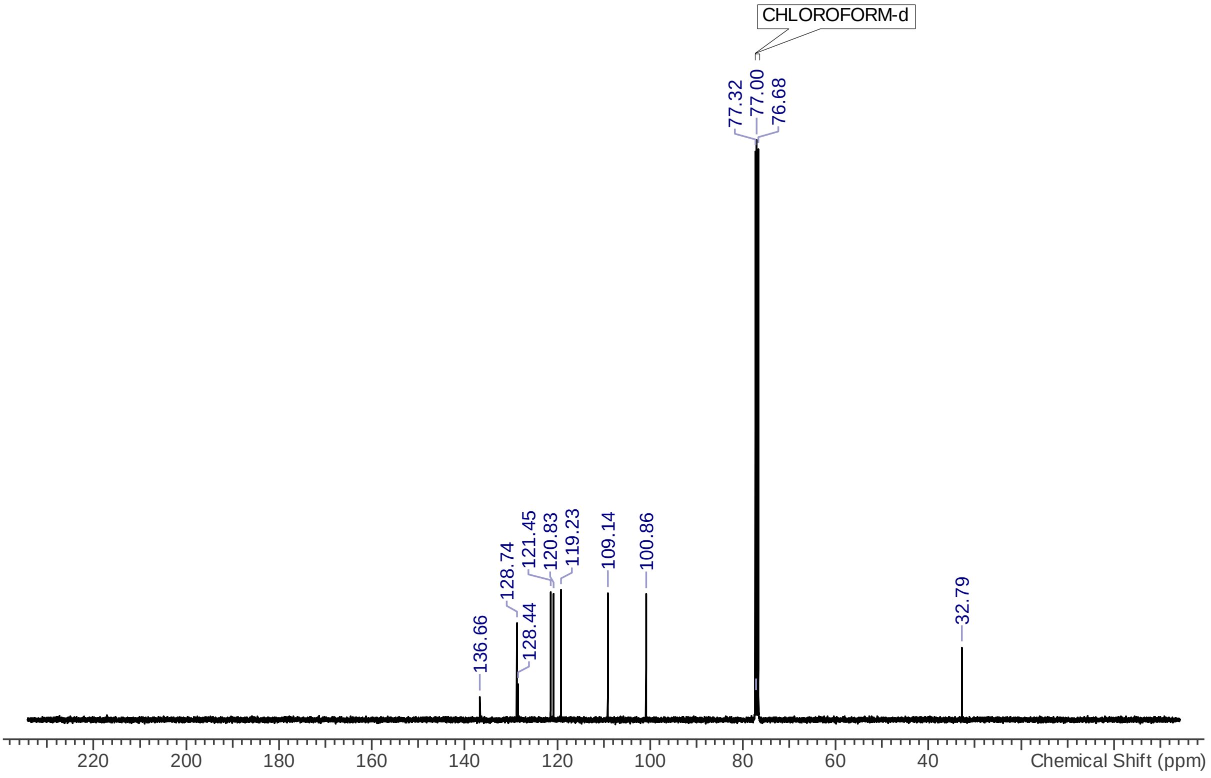

1,2-Dimethyl-1*H*-indole 5b

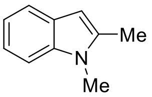

The title compound was prepared according to general procedure 5 (reaction using 5 mol% $B(C_6F_5)_3$, 10 mol% Et_3SiH heated to 40°C) using 1,2-dimethylindoline **4b** (29.4 mg, 0.20 mmol) with an NMR yield of 71%. Purification by flash column chromatography on silica gel (11% DCM in pet. ether) gave the title compound **4b** as a white amorphous solid (15.0 mg, 0.10 mmol, 48%). $\mathbf{R_f}$ = 0.39 (eluent = 11% DCM in pet. ether); **^{1}H NMR** (400 MHz, $CDCl_3$) δ_H = 7.58 (1H, d, *J* = 7.8 Hz), 7.30 (1H, dd, *J* = 8.1, 0.9 Hz), 7.21 (1H, dd, *J* = 8.1, 7.1 Hz), 7.12 (1H, dd, *J* = 7.8, 7.1 Hz, 1H), 6.30 (1H, app. quin, *J* = 0.9 Hz), 3.69 (3H, s), 2.47 (3H, d, *J* = 0.9 Hz); **^{13}C NMR** (101 MHz, $CDCl_3$) δ_C = 137.3 (C), 136.7 (C), 127.9 (C), 120.4 (CH), 119.6 (CH), 119.2 (CH), 108.7 (CH), 99.5 (CH), 29.3 (CH_3), 12.7 (CH_3). Spectroscopic data in accordance with that stated in the literature.[15]

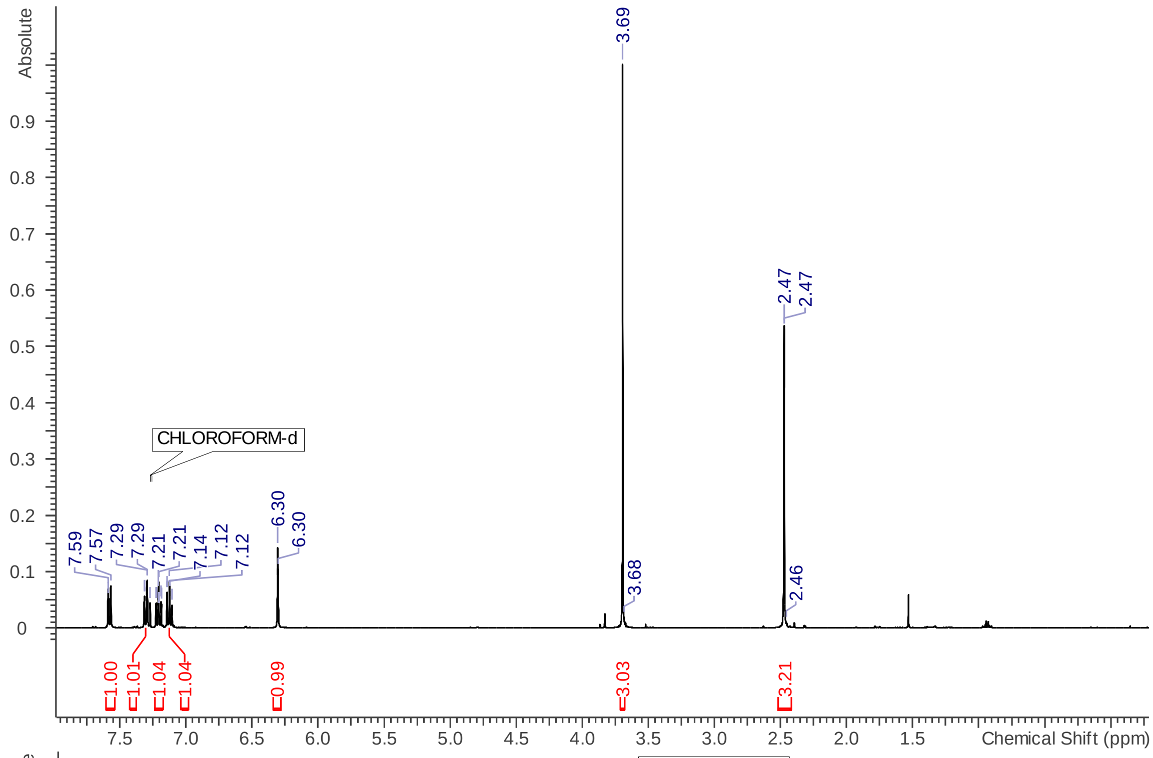

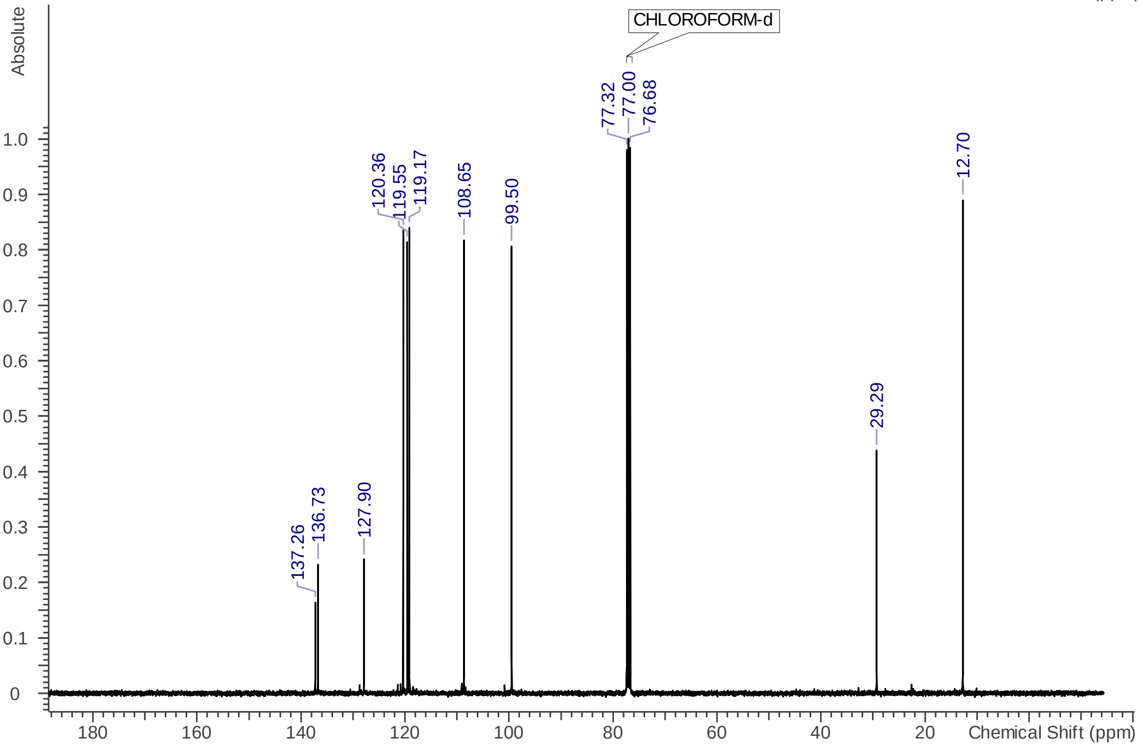

5. Mechanistic studies

5.1 Identification of reaction intermediates

Stoichiometric reaction of N-mesityl pyrrolidine and $B(C_6F_5)_3$

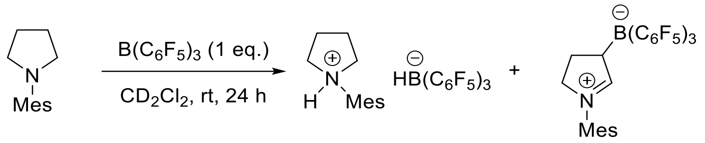

1a (1 eq.) **6a** **7a**

In a glovebox (Ar), *N*-Mesityl pyrrolidine **1a** (20.8 mg, 0.11 mmol) and $B(C_6F_5)_3$ (prepared according to general procedure 6, 56.3 mg, 0.11 mmol) were dissolved in DCM-d$_2$ (0.7 mL) and added to a J. Youngs NMR tube. Cyclohexane (Int. standard, 12 µL, 0.11 mmol) was added, before the NMR tube was sealed and periodically shaken for 24 h, until equilibrium was reached. **6a** and **7a** were found to be present in a 1:1 mixture.

6a (50%): ^{1}**H NMR** (400 MHz, CD_2Cl_2) δ_H = 7.58 (1H, br. s), 7.05 (2H, br. s), 4.23–4.08 (2H, m), 3.86–3.68 (2H, m), 2.62–2.47 (2H, m), 2.43 (6H, br. s), 2.38–2.33 (2H, m), 2.29 (3H, br. s); ^{13}**C NMR** (101 MHz, CD_2Cl_2) δ_C = 148.8 (2×C, d, $^{1}J_{C\text{-}F}$ = 239 Hz), 142.1 (C), 138.7 (C, m), 136.3 (C, m), 133.4 (C), 133.2 (2×CH), 130.0 (2×C), 59.1 (2×CH_2), 26.0 (2×CH_2), 21.2 (CH_3), 18.6 (2×CH_3); ^{11}**B NMR** (160 MHz, CD_2Cl_2) δ_B = −25.26 (br. s)*; ^{19}**F NMR** (376 MHz, CD_2Cl_2) δ_F = −133.72–−134.75 (6F, m), −163.4–−164.2 (3F, m), −166.3–−168.0 (6F, m). **VT NMR at −266K resolved this peak to: −25.11 (br. d, $^{1}J_{B\text{-}H}$ = 90.8 Hz).*

7a (50%): ^{1}**H NMR** (400 MHz, CD_2Cl_2) δ_H = 8.24 (1H, s), 7.00 (1H, s), 6.99 (1H, s), 4.93–4.73 (1H, m), 4.07–3.96 (2H, m), 2.88–2.71 (1H, m), 2.31 (3H, br. s), 2.18 (3H, br. s), 2.12–2.06 (1H, m), 2.00 (3H, br. s); ^{13}**C NMR** (101 MHz, CD_2Cl_2) δ_C = 195.6 (CH), 142.3 (C), 134.1 (C), 132.7 (C), 132.6 (C), 130.8 (CH), 130.6 (CH), 62.4 (CH_2), 49.4 (CH)†, 25.8 (CH_2), 20.9 (CH_3), 17.9 (CH_3)*, 16.8 (CH_3)*; ^{11}**B NMR** (160 MHz, CD_2Cl_2) δ_B = -13.33 (s, Ar_3BC) ppm. ^{19}**F** (376 MHz, CD_2Cl_2) δ_F = −131.83–−132.66 (6F, m), −160.54 (3F, app. t, $J_{F\text{-}F}$ = 20.4 Hz), −165.0 (6F, app. t, J = 20.4 Hz). *In ^{13}C NMR -$B(C_6F_5)_3$ moeity signals either consumed in broad signals for 6a, or not seen due to being too broad. *Methyl groups of mesityl believed to be inequivalent due to restricted rotation. †Peak at 49.4 confirmed by HSQC, likely broad as CH next to B.*

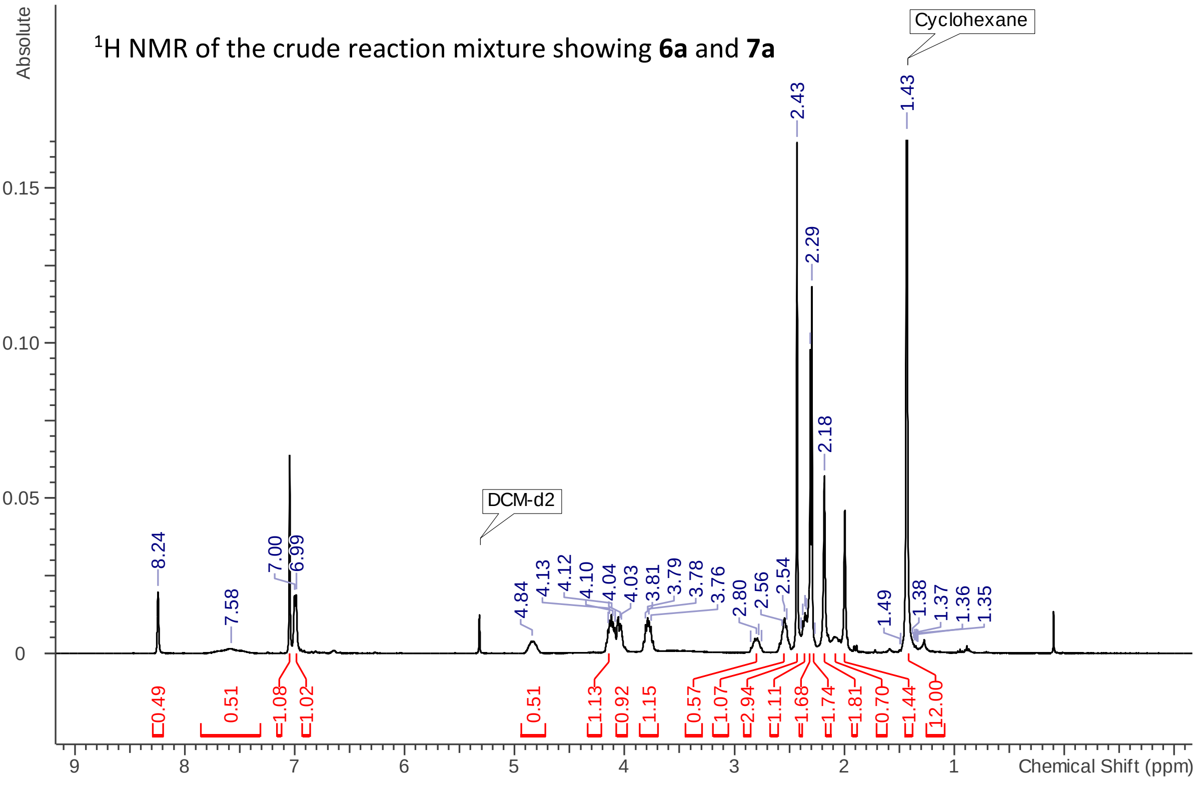

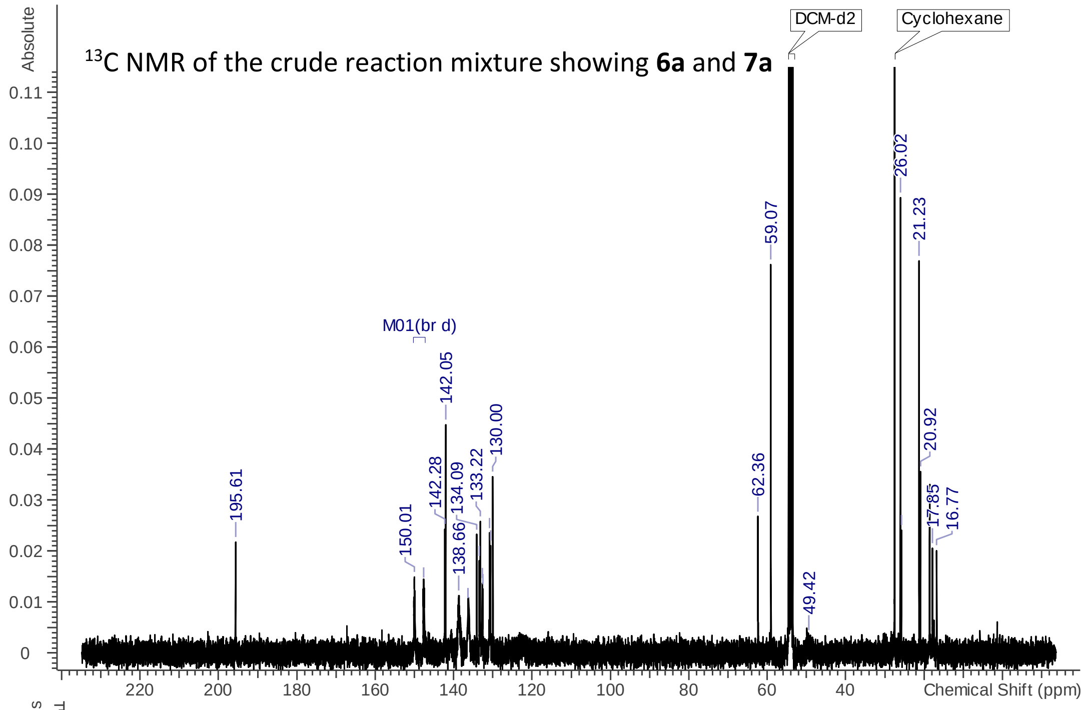

^{13}C NMR of the crude reaction mixture showing **6a** and **7a**
DCM-d2
Cyclohexane
M01(br d)
195.61
150.01
142.28
142.05
138.66
134.09
133.22
130.00
62.36
59.07
49.42
26.02
21.23
20.92
17.85
16.77
Absolute
Chemical Shift (ppm)

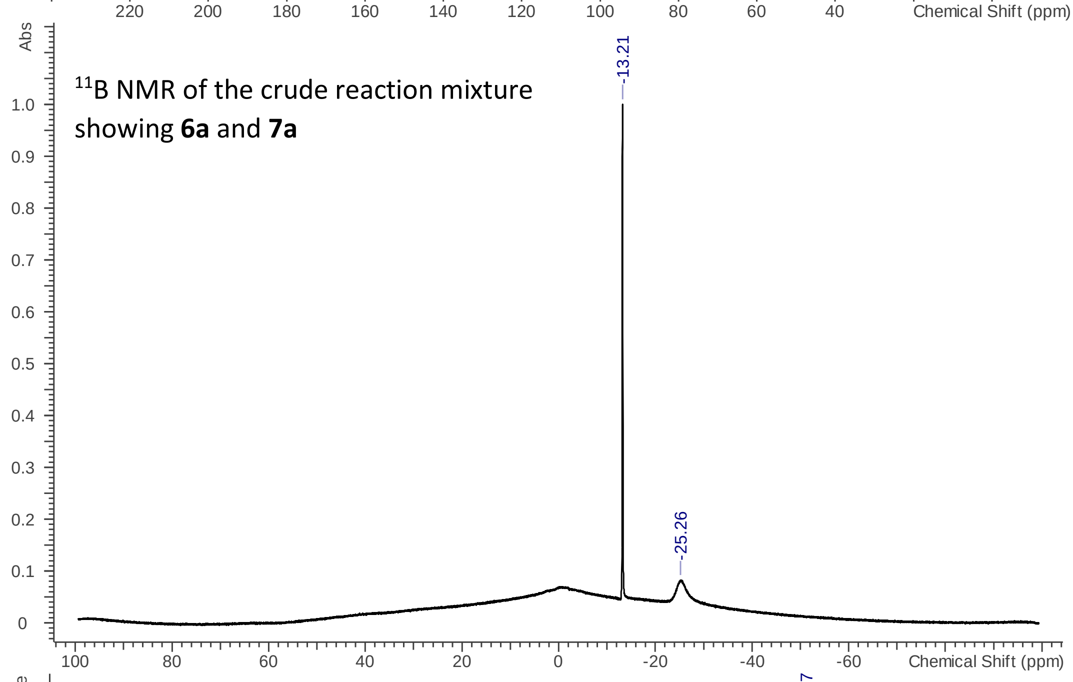

^{11}B NMR of the crude reaction mixture showing **6a** and **7a**
-13.21
-25.26
Abs
Chemical Shift (ppm)

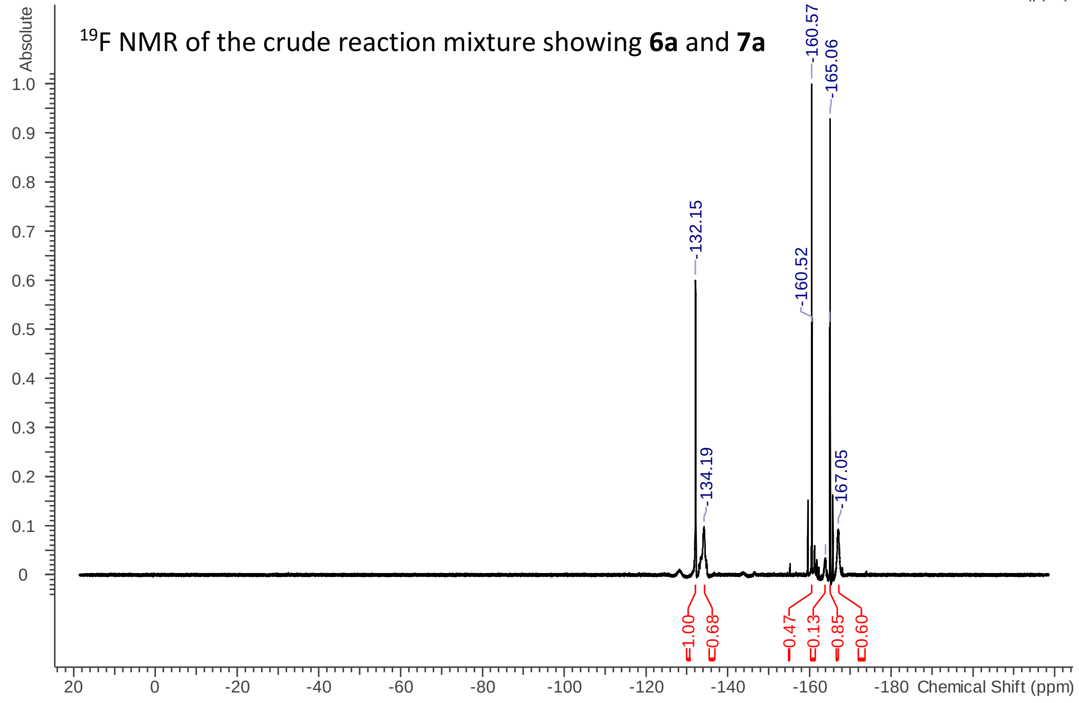

^{19}F NMR of the crude reaction mixture showing **6a** and **7a**
-132.15
-134.19
-160.52
-160.57
-165.06
-167.05
1.00
0.68
0.47
0.13
0.85
0.60
Absolute
Chemical Shift (ppm)

Synthesis of ammonium borohydrides:

General procedure 8: Synthesis of ammonium borohydrides

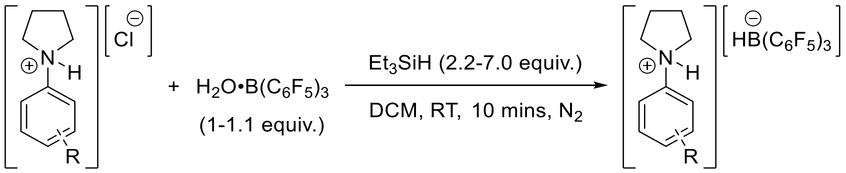

According to a modified literature procedure,[17] a 25 mL J. Youngs ampoule equipped with stirrer bar was charged with $H_2O{\cdot}B(C_6F_5)_3$ (1-1.1 equiv.) under a nitrogen atmosphere, before being suspended in DCM (2 mL/mmol). Et_3SiH (2.2-7.0 equiv.) was added slowly, and the mixture allowed to stir at ambient temperature for 10 minutes. In a separate ampoule, pyrrolidine HCl salt (1.0 equiv.) was dissolved in DCM (3 mL), before being added slowly to the $B(C_6F_5)_3$ solution, before further Et_3SiH (1.1 equiv.) was added slowly and allowed to stir at ambient temperature for 20 minutes. After this time, the DCM was removed in vacuo to about 1/5 it's original volume, before dry pentane (2 mL) was added, and the suspension stirred overnight. The pentane was then decanted with a syringe, before the resulting solid was washed with further dry pentane (2 × 2 mL). The white solid that was left behind was dried in vacuo for at least 2 h, before being taken into the glovebox for analysis.

1-Mesitylpyrrolidin-1-ium trispentafluorophenylhydridoborate 6a

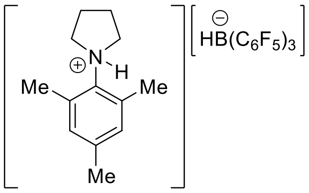

The title compound was prepared according to general procedure 8 using 1-mesitylpyrrolidin-1-ium chloride (226 mg, 1.0 mmol), $H_2O{\cdot}B(C_6F_5)_3$ (530 mg, 1.0 mmol), and Et_3SiH (0.53 mL, 3.3 mmol), which gave the title compound **6a** as a white solid (384 mg, 0.55 mmol, 55%). **^{1}H NMR** (400 MHz, CD_2Cl_2) δ_H = 7.61 (1H, br. s), 7.03 (2H, br. d, J = 0.5 Hz), 4.19–4.07 (2H, m), 3.85–3.70 (2H, m), 3.54 (1H, br. q, $^1J_{H\text{-}B}$ = 88.9 Hz), 2.59–2.47 (2H, m), 2.42 (6H, s), 2.38–2.31 (2H, m), 2.30 (3H, br. s); **^{13}C NMR** (101 MHz, CD_2Cl_2) δ_C = 148.7 (6×C, br. d, $^1J_{C\text{-}F}$ = 238.8 Hz), 142.2 (C), 138.5 (3×C, br. d, $^1J_{C\text{-}F}$ = 241.5 Hz), 135.9 (6×C, br. d, $^1J_{C\text{-}F}$ = 248.5 Hz), 133.5 (C), 133.2 (2×CH), 130.0 (2×C), 125.1 (3×C, v. br. s), 59.0 (2×CH_2), 26.0 (2×CH_2), 20.9 (CH_3), 18.5 (2×CH_3); **^{11}B NMR** (160 MHz, CD_2Cl_2) δ_B = −24.98 (br. d, $1J_{B\text{-}H}$ = 88.9 Hz); **^{19}F NMR** (376 MHz, CD_2Cl_2) δ_F = −134.31 (6F, br d, J = 22.5 Hz), −163.78 (3F, app. t, J = 20.3 Hz), −166.95–−167.15 (6F, m).

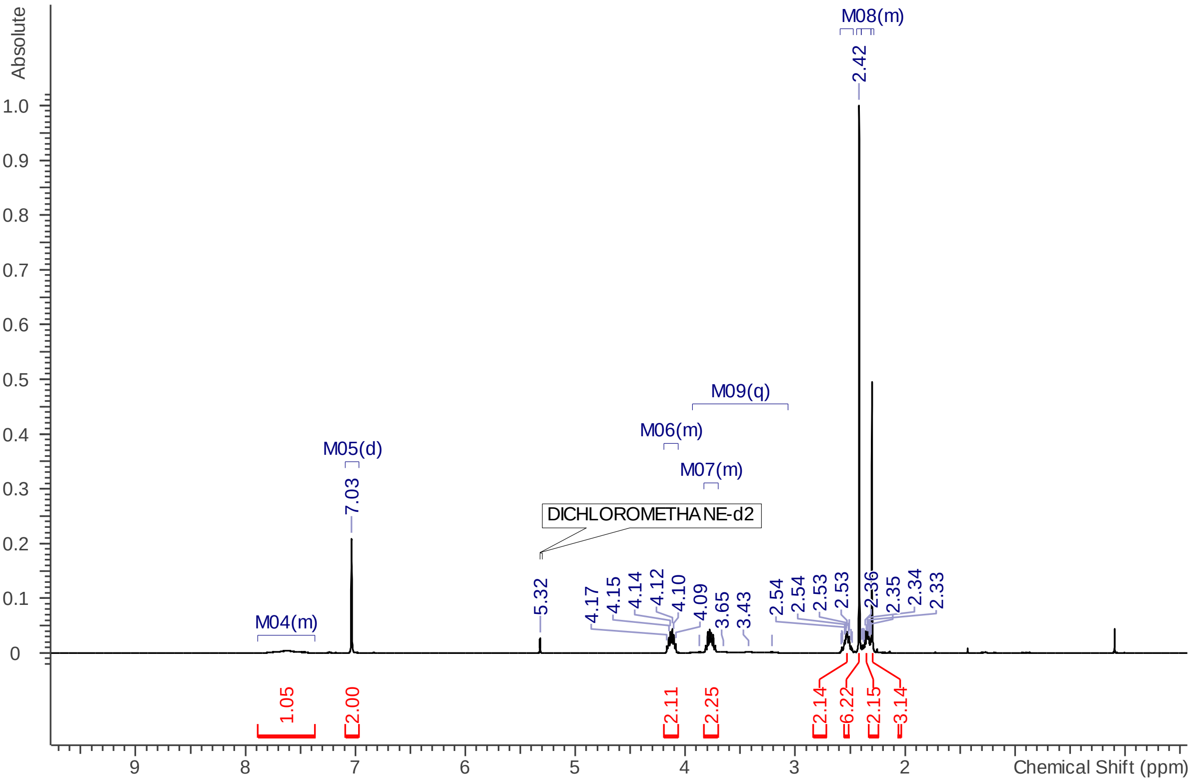

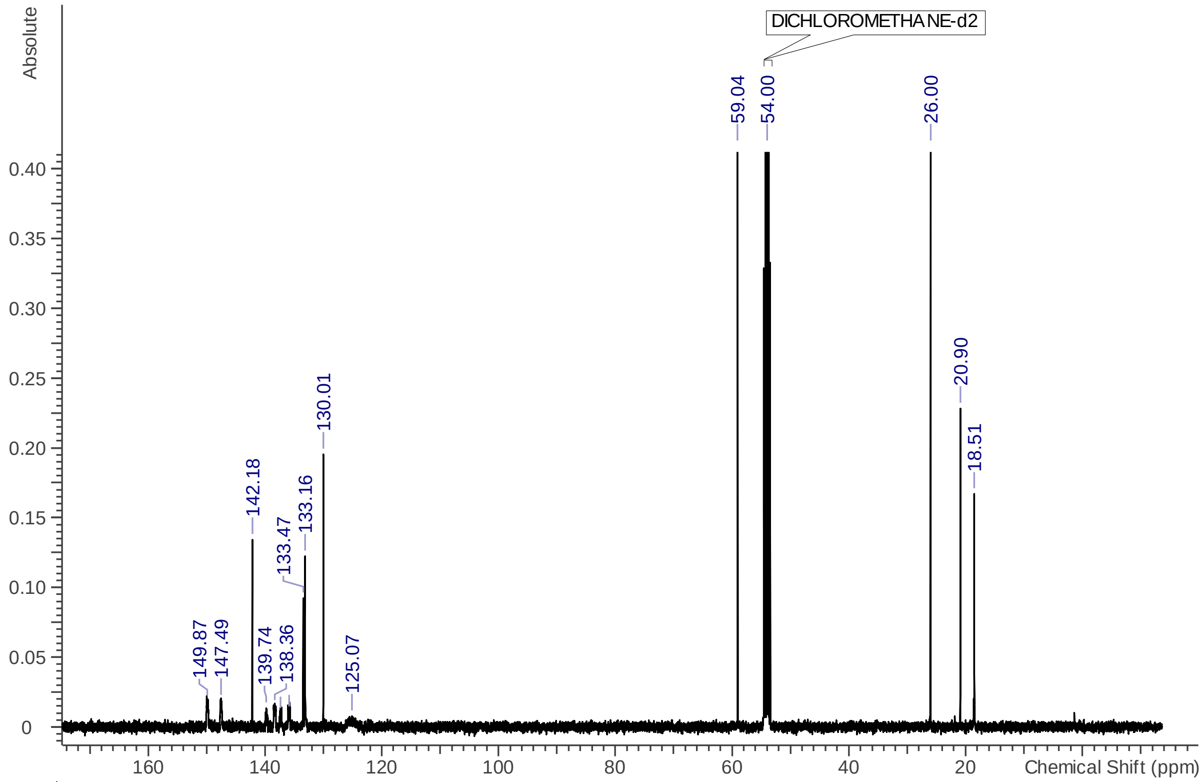
DICHLOROMETHANE-d2
59.04
54.00
26.00
20.90
18.51
149.87
147.49
142.18
139.74
138.36
133.47
133.16
130.01
125.07
Absolute
Chemical Shift (ppm)

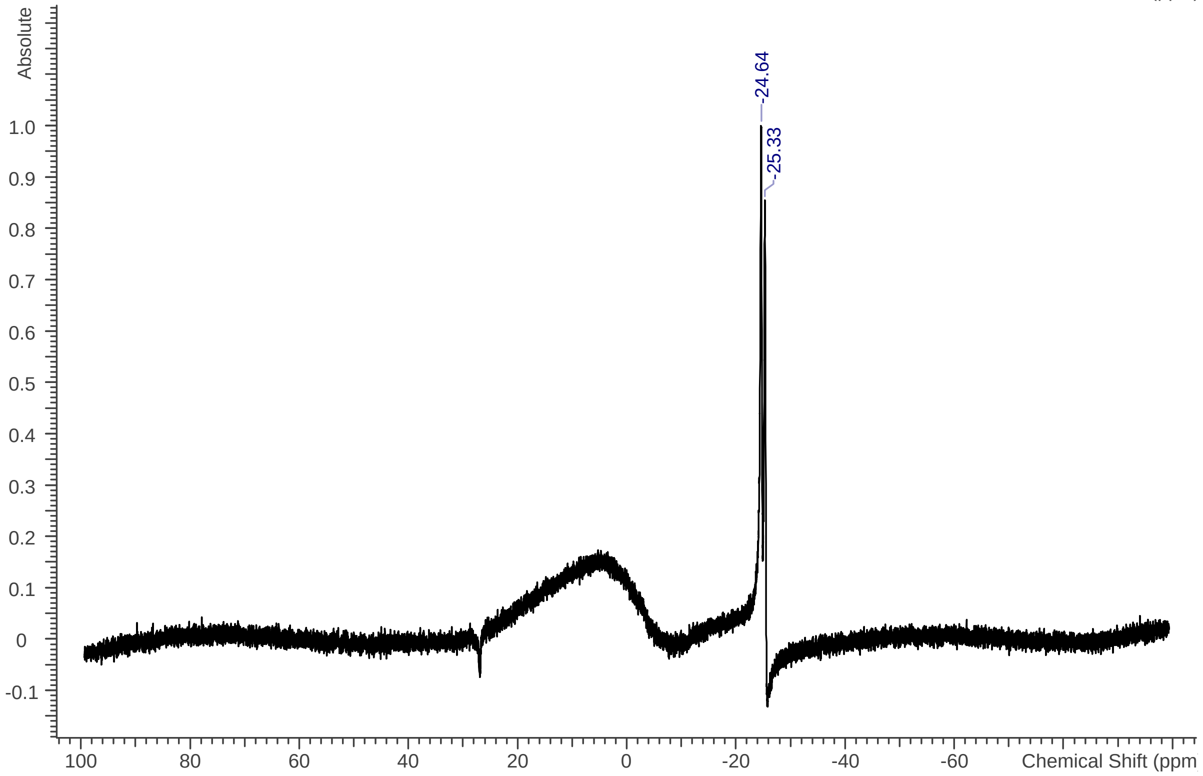
-24.64
-25.33
Absolute
Chemical Shift (ppm

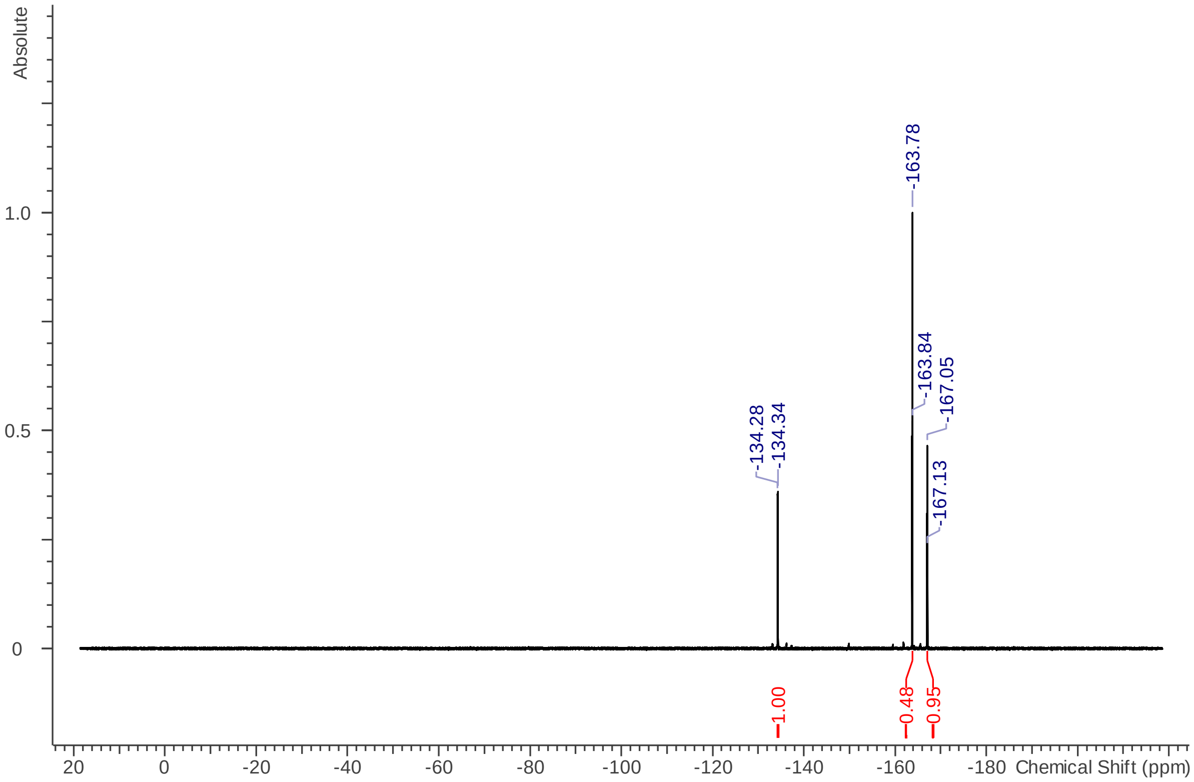

Absolute
1.0
0.5
0
-134.28
-134.34
-163.78
-163.84
-167.05
-167.13
1.00
0.48
0.95
20
0
-20
-40
-60
-80
-100
-120
-140
-160
-180
Chemical Shift (ppm)

1-(2-Chlorophenyl)pyrrolidin-1-ium trispentafluorophenylhydridoborate 6c

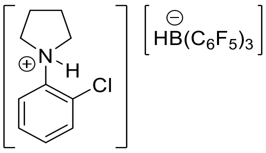

The title compound was prepared according to general procedure 8 using 1-(2-chlorophenyl)pyrrolidin-1-ium chloride (109 mg, 0.5 mmol), $H_2O{\cdot}B(C_6F_5)_3$ (293 mg, 0.55 mmol), and Et_3SiH (0.66 mL, 4.1 mmol), which gave the title compound **6c** as a white solid (240 mg, 0.35 mmol, 69%). **^{1}H NMR** (400 MHz, C_6D_6) δ_H = 7.86 (1H, s), 6.79 (1H, dd, *J* = 8.0, 1.3 Hz), 6.72 (1H, ddd, *J* = 8.2, 7.9, 1.3 Hz), 6.61 (1H, ddd, *J* = 8.0, 7.9, 1.2 Hz), 6.42 (1H, dd, *J* = 8.2, 1.2 Hz), 4.07–3.40 (1H, m), 2.86–2.74 (4H, m), 1.35–1.30 (2H, m); **^{11}B NMR** (160 MHz, C_6D_6) δ_B = −24.26 (br. d, $^{1}J_{B\text{-}H}$ = 89.5 Hz); **^{19}F NMR** (376 MHz, C_6D_6) δ_F = −133.42 (6F, br d, *J* = 22.5 Hz), −162.43 (3F, app. t, *J* = 20.6 Hz), −165.82–−166.20 (6F, m).

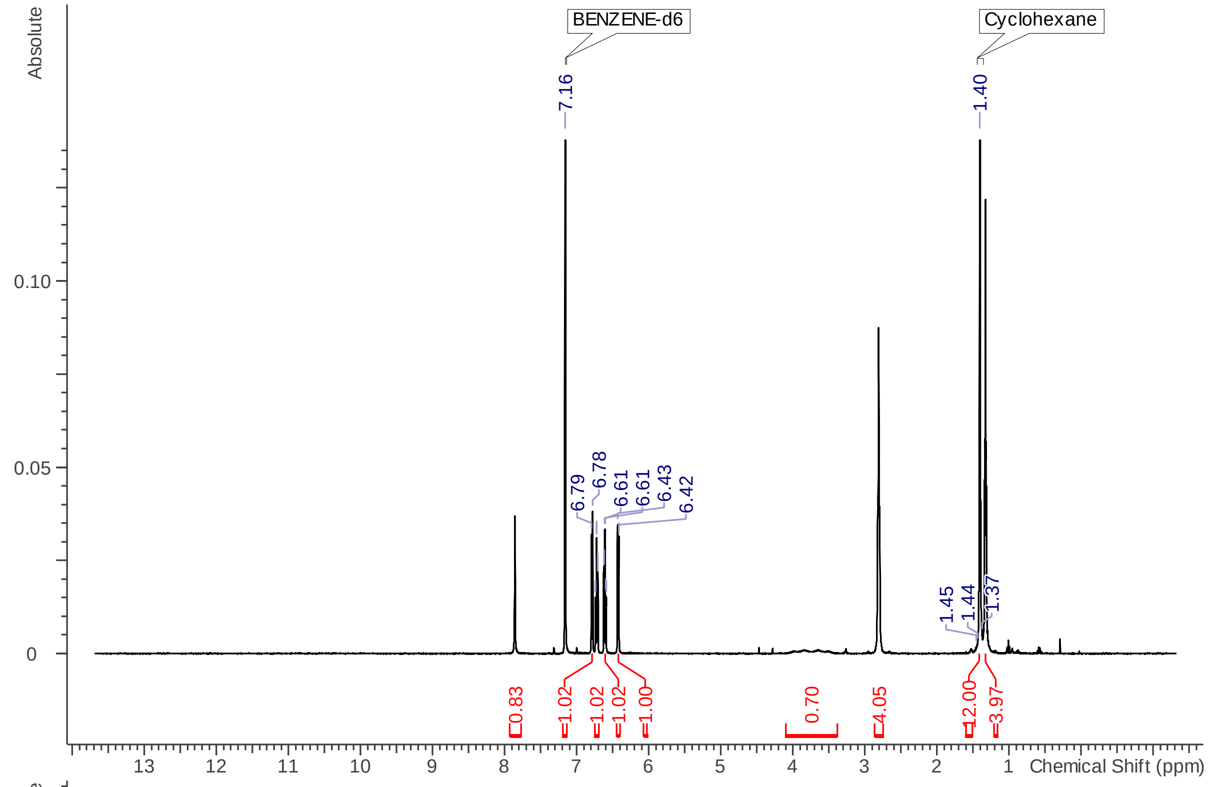

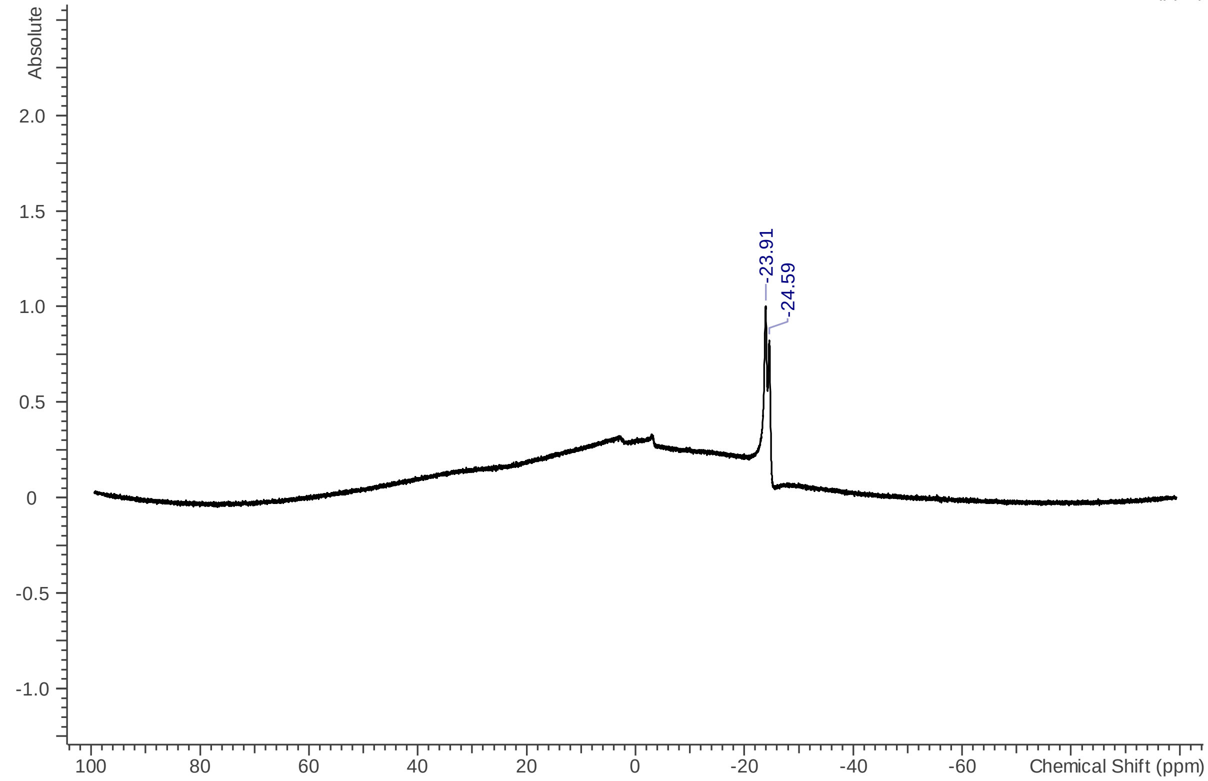

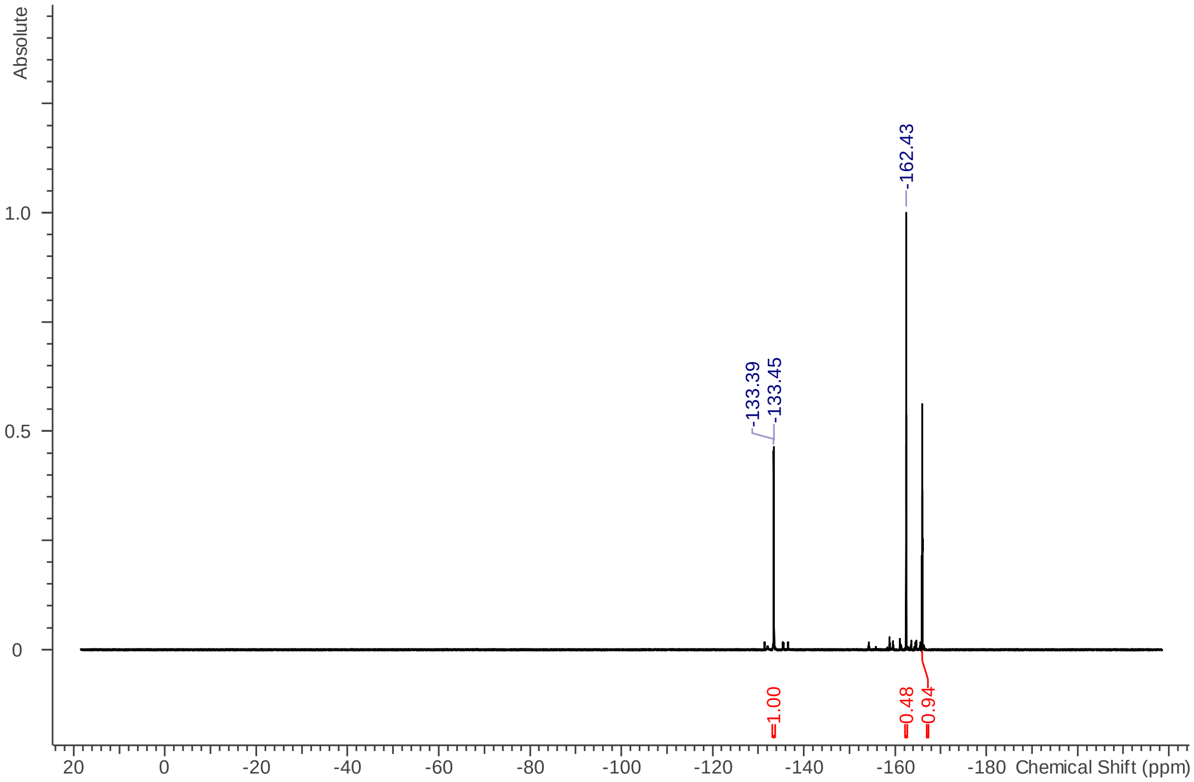

Absolute
1.0
0.5
0
-133.39
-133.45
-162.43
1.00
0.48
0.94
20
0
-20
-40
-60
-80
-100
-120
-140
-160
-180
Chemical Shift (ppm)

Tris(pentafluorophenyl)((1-phenyl-3,4-dihydro-2H-pyrrol-1-ium-5-yl)methyl)borate, 8n

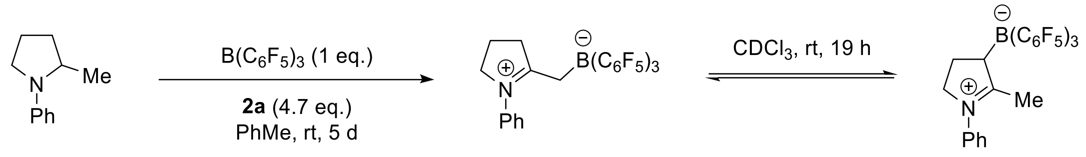

1e (1 eq.) **8e**, 67% **7e**

A 4 mL J. Youngs ampoule equipped with stirrer bar was charged with 1-phenyl-2-methyl-pyrrolidine (76.8 mg, 0.48 mmol), $B(C_6F_5)_3$ (246 mg, 0.48 mmol) and PhMe (3.0 mL) in a glovebox. 2-methallyltrimethylsilane (0.4 mL, 2.28 mmol) was then added, and the ampoule sealed. The reaction flask was stirred at room temperature for 5 days, yielding a clear light pink solution with a white precipitate. The precipitate was isolated by cannula filtration, before being washed with pentane (2 × 2 mL). The remaining solid was then dried in vacuo, to yield **8e** as an amorphous white solid (216 mg, 0.32 mmol, 67%). Single crystals of **8e** suitable for X-ray diffraction were obtained from the slow evaporation of a reaction of the same concentration in C_6D_6. Equilibration of **8e** to **7e** in $CDCl_3$ occurred over 19 h to give a 53:47 mixture of **8e**:**7e**.

8e: ^{1}**H NMR** (400 MHz, $CDCl_3$) δ_H = 7.50–7.42 (3H, m), 6.92–6.85 (2H, m), 4.18 (2H, br. t, *J* = 7.8 Hz), 3.43–3.34 (2H, m), 3.15 (2H, br. t, *J* = 8.1 Hz), 2.21 (2H, tt, *J* = 8.1, 7.9 Hz); ^{11}**B NMR** (160 MHz, $CDCl_3$) δ_B = −13.17.

7e: ^{1}**H NMR** (400 MHz, $CDCl_3$) δ_H = 7.62–7.54 (3 H, m), 7.19–7.13 (2H, m), 4.85–4.72 (1H, m), 3.92 (1H, app. br. t, *J* = 11.7 Hz), 2.93 (1H, app. br. q, *J* = 11.1 Hz), 2.85–2.73 (1H, m), 3.35–2.26 (1H, m), 2.04 (3H, br s); ^{11}**B NMR** (160 MHz, $CDCl_3$) δ_B = −11.69.

8e+7e: ^{19}**F NMR** (376 MHz, $CDCl_3$) δ_F = −132.10 (6F, br. d, *J* = 21.8 Hz), −159.86 (3F, br. t, *J*= 20.4 Hz), −164.35–−164.57 (6F, m).

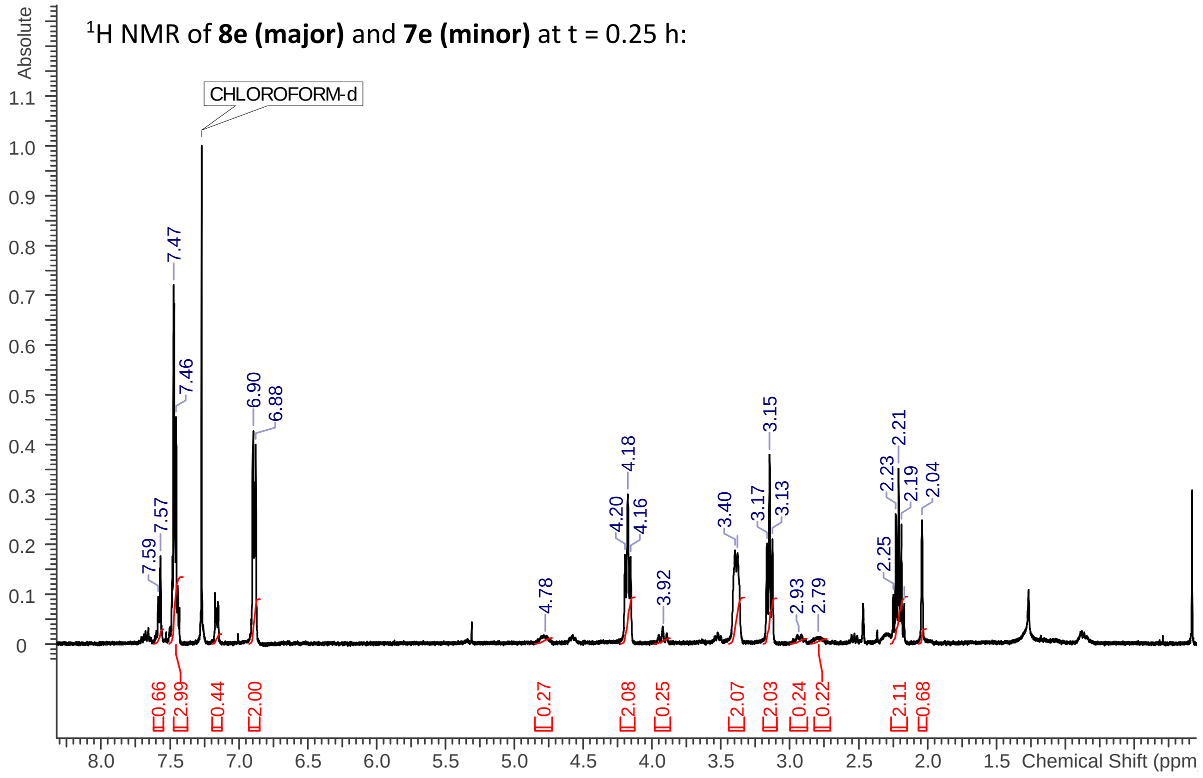

^{11}B NMR of **8e (major)** and **7e (minor)** at t = 0.25 h:

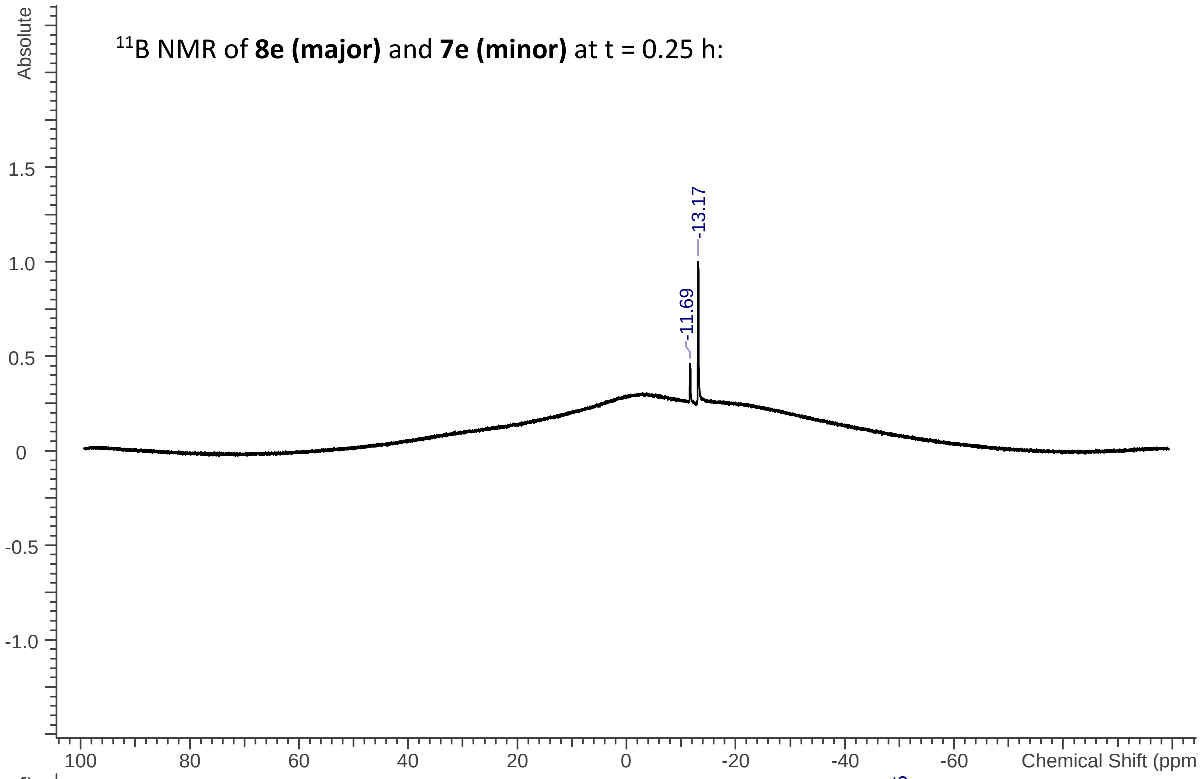

^{19}F NMR of **8e** and **7e** at t = 0.25 h:

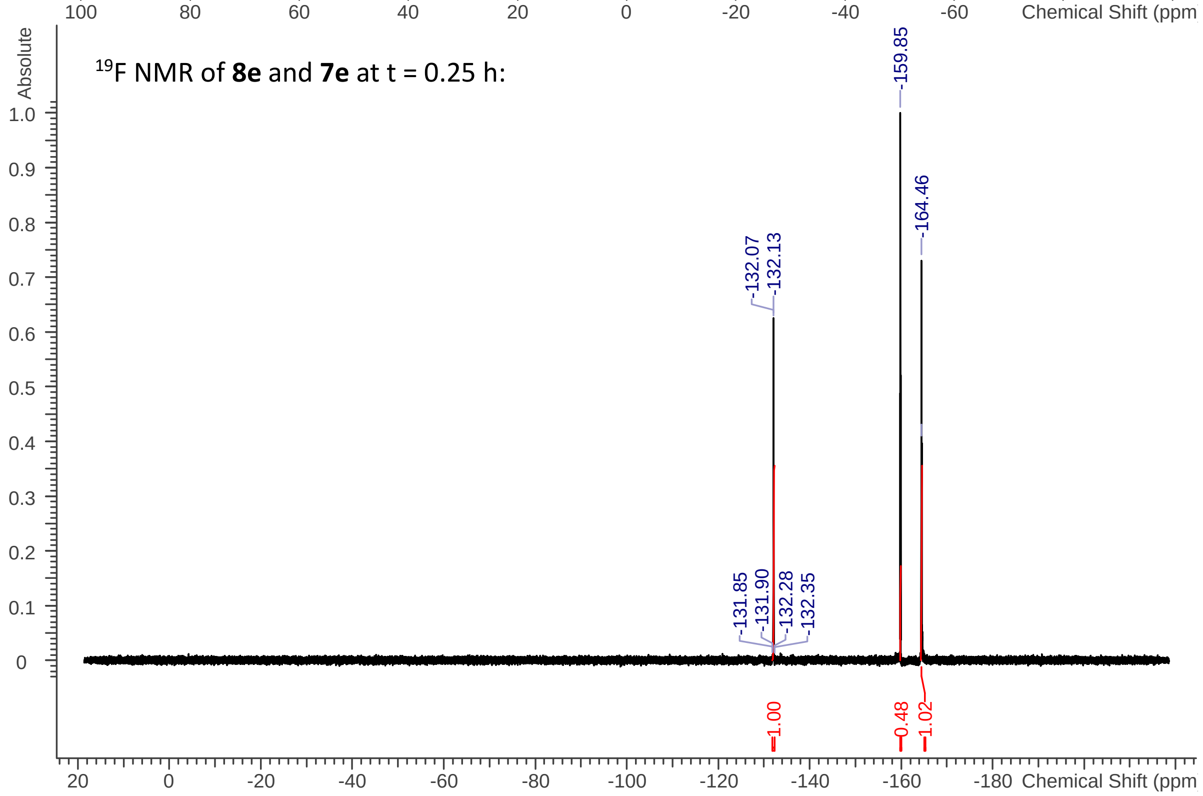

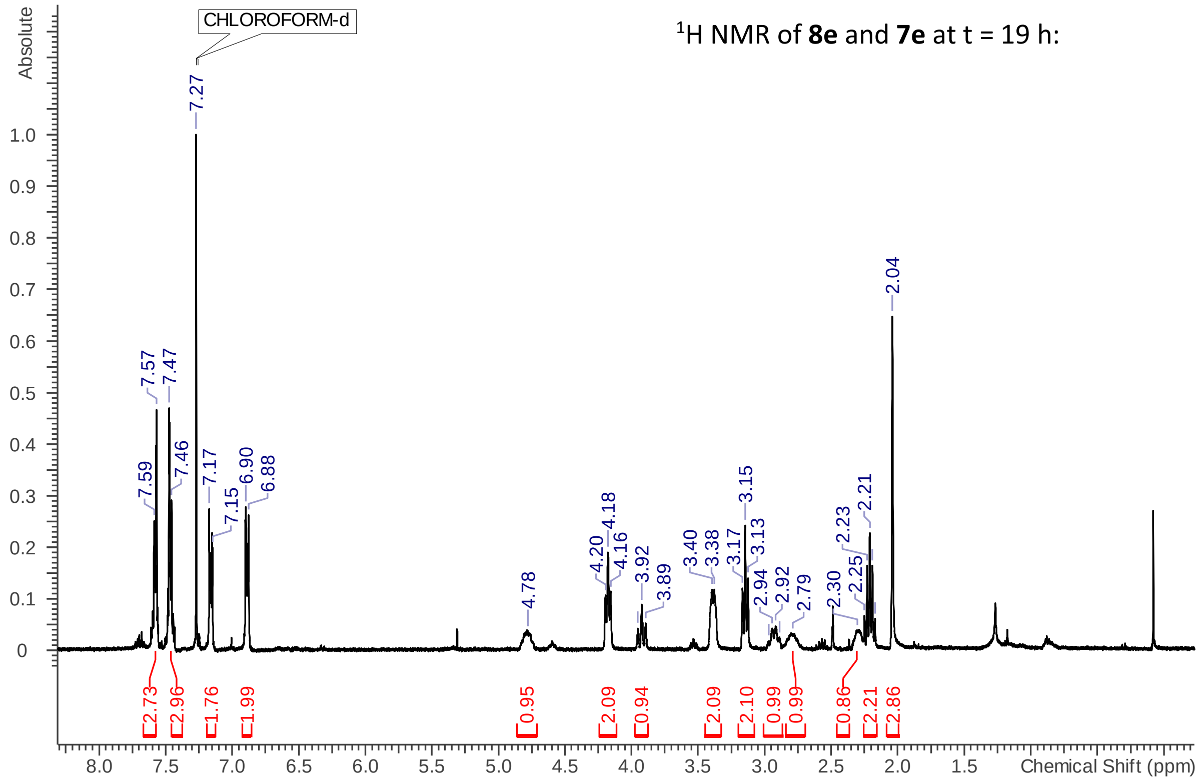
^{1}H NMR of 8e and 7e at t = 19 h:
CHLOROFORM-d
Absolute
Chemical Shift (ppm)

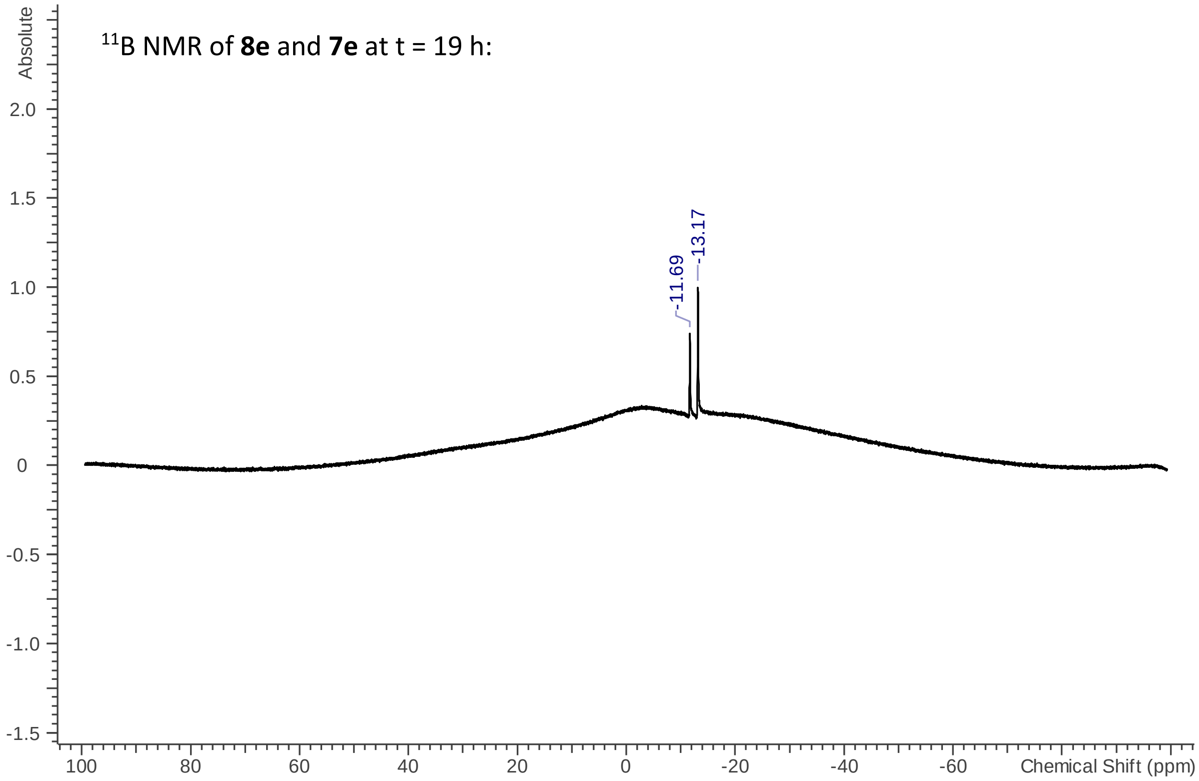
^{11}B NMR of 8e and 7e at t = 19 h:
-11.69
-13.17
Absolute
Chemical Shift (ppm)

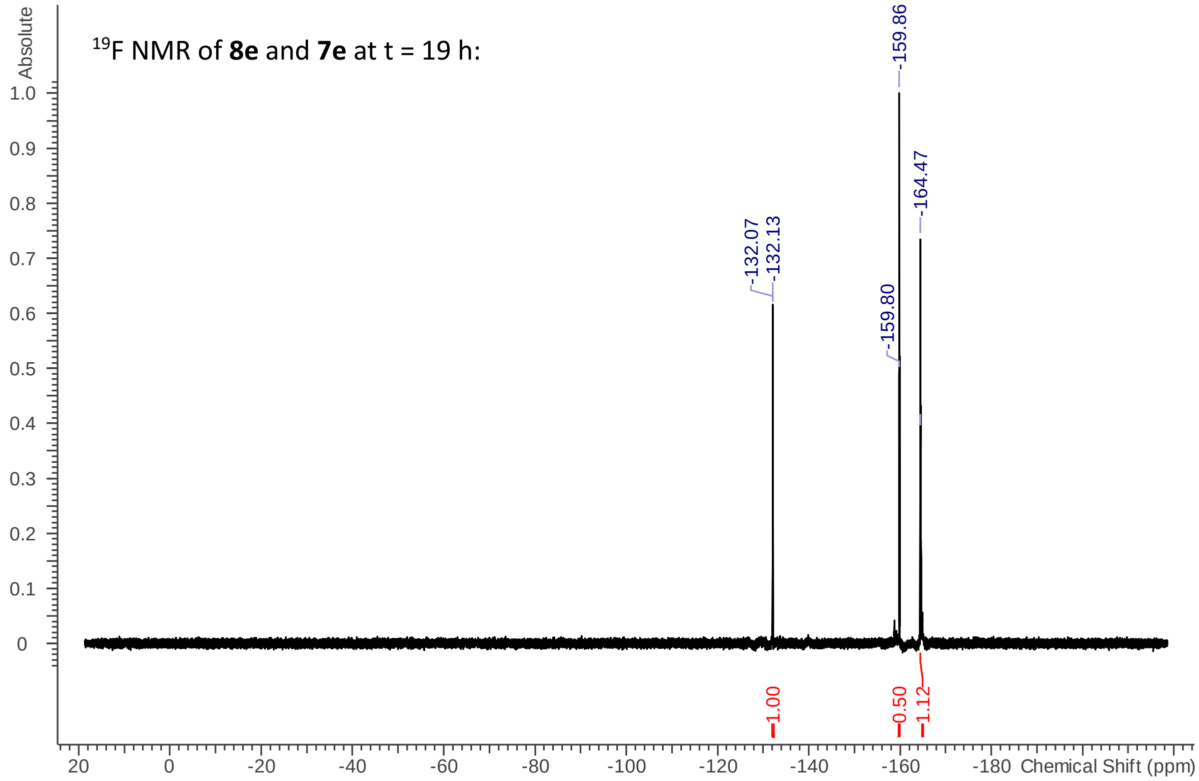

^{19}F NMR of **8e** and **7e** at t = 19 h:

Isobutyltrimethylsilane H_2·2a

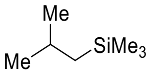

To a 25 mL J. Youngs ampoule equipped with stirrer bar was added $H_2O·B(C_6F_5)_3$ (169.6 mg, 0.32 mmol) under a nitrogen atmosphere. *o*-DCB (4.8 mL) was added, before Et_3SiH (0.1 mL, 0.64 mmol) was added slowly with stirring, and the mixture allowed to stir for 10 minutes. In a separate vial, 1-(2,4-dichlorophenyl)-2-methylpyrrolidine **3r** (340 mg, 1.6 mmol) was dissolved in *o*-DCB (1.2 mL) under a nitrogen atmosphere, before being slowly added to the $B(C_6F_5)_3$ solution. Subsequently, methallyltrimethylsilane **2a** (0.70 mL, 4 mmol) was added, before the reaction was sealed and heated to 140 °C for 22 h. Upon completion, the reaction was distilled using Kugelrohr apparatus (40 °C, 650 mbar) to give the title compound H_2·**2a** as a colourless liquid (56 mg, 0.43 mmol, 13%). **^{1}H NMR** (400 MHz, $CDCl_3$) δ_H = 1.77 (1H, tspt, *J* = 6.8, 6.6 Hz), 0.93 (6H, d, *J* = 6.6 Hz), 0.52 (2H, d, *J* = 6.8 Hz), 0.00 (9H, s); **^{13}C NMR** (101 MHz, $CDCl_3$) δ_C = 27.4 (CH), 26.3 (2×CH_3), 25.0 (CH_2) 0.65 (3×CH_3); Spectroscopic data in accordance with that stated in the literature.[18]

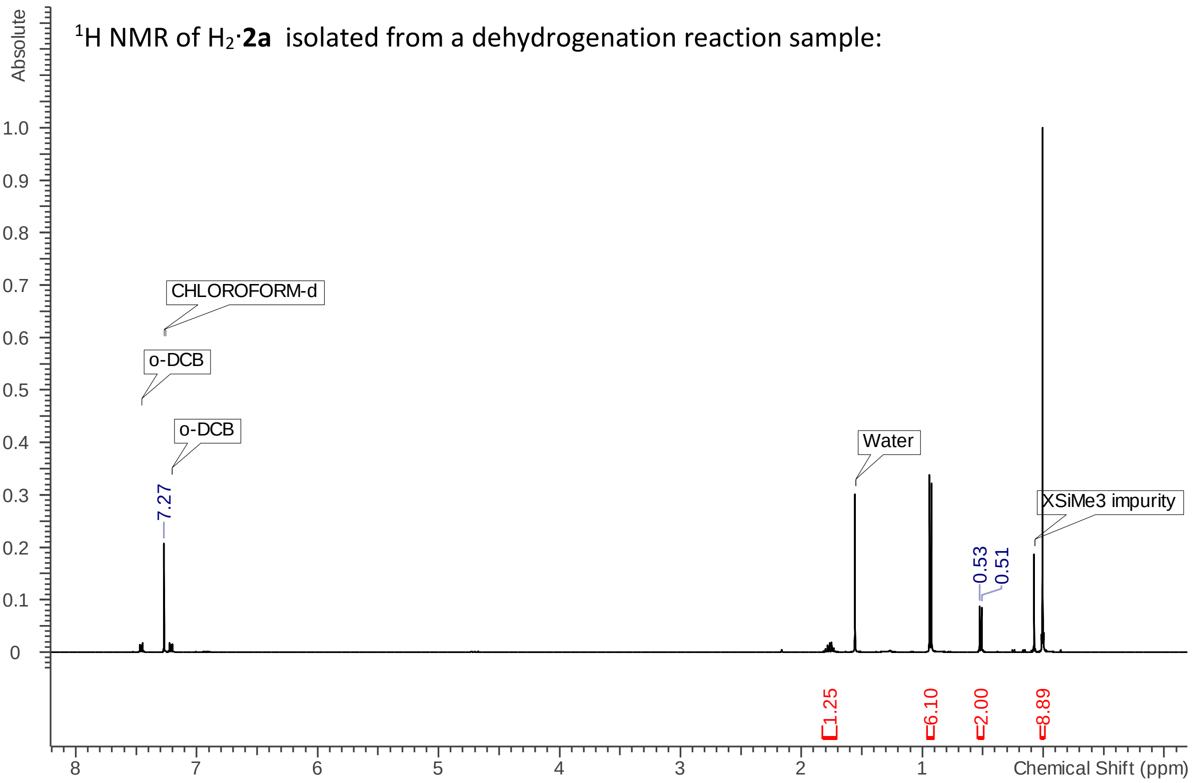

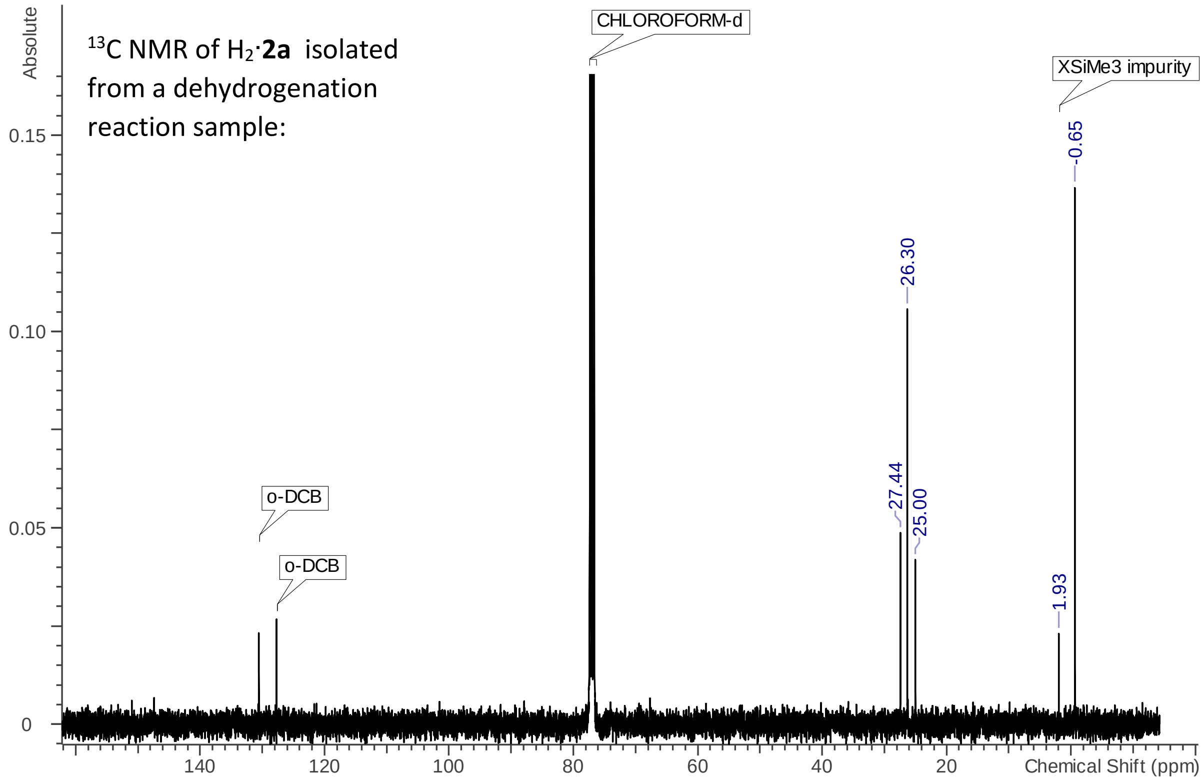

5.2 Reactivity of reaction intermediates

General procedure 9: Catalytic competency of isolated intermediates

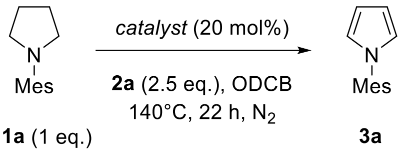

A 4 mL J. Youngs ampoule equipped with stirrer bar was charged with catalyst (20 mol%) under glovebox conditions. This was then sealed and removed from the glovebox, and diluted with o-DCB (0.15 mL) under a nitrogen atmosphere. A solution of *N*-mesitylpyrrolidine **1a** (0.2 mmol, 1 equiv.) in o-DCB (0.6 mL) was then added to the mixture, before methallyltrimethylsilane **2a** (2.5 equiv., stored over mol. sieves) was added, and the ampoule was sealed and stirred at 140 °C for 22 h. Upon completion, the reaction was allowed to cool to room temperature, before being quenched with $NaHCO_3$ (aq., sat, 1.5 mL), and then extracted with DCM (3 × 2 mL). The combined organics were dried with a $MgSO_4$ plug, and concentrated in vacuo. $MeNO_2$ (21.5 µL, 0.2 mmol) was added as an internal standard, allowing an NMR yield of **3a** to be calculated.

Using isolated water-free $B(C_6F_5)_3$:

The reaction was prepared according to general procedure 9 using $B(C_6F_5)_3$ (prepared as in general procedure 6, 20.5 mg, 0.04 mmol), *N*-mesitylpyrrolidine **1a** (37.9 mg, 0.2 mmol) and methallyltrimethylsilane **2a** (88µL, 0.5 mmol) formed *N*-mesitylpyrrole **3a** with an NMR yield of 89%.

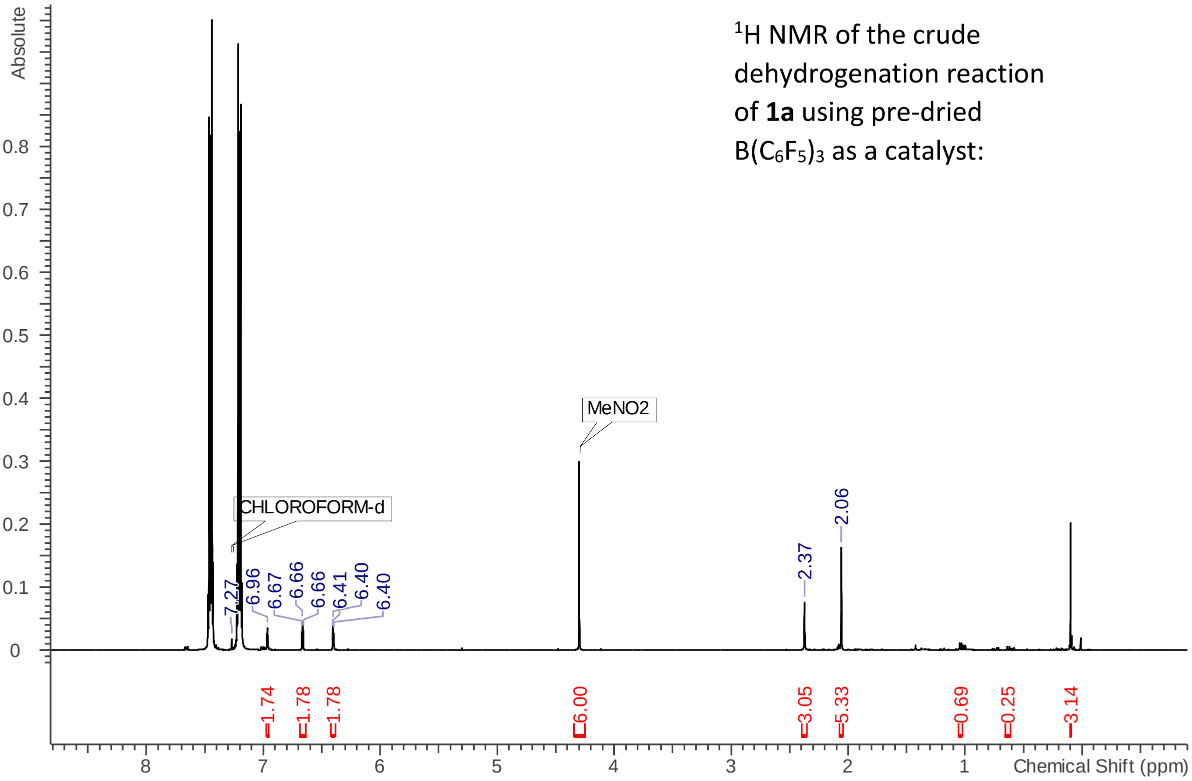

^{1}H NMR of the crude dehydrogenation reaction of **1a** using pre-dried $B(C_6F_5)_3$ as a catalyst:

Using 6a:

The reaction was set up according to general procedure 9 using 1-mesitylpyrrolidin-1-ium trispentafluorophenylhydridoborate **6a** (25.8 mg, 0.04 mmol), *N*-mesitylpyrrolidine **1a** (37.9 mg, 0.2 mmol) and methallyltrimethylsilane **2a** (88 µL, 0.5 mmol) formed *N*-mesitylpyrrole **3a** with an NMR yield of 74% (based off of 0.24 mmol total N-mesitylpyrrolidine content).

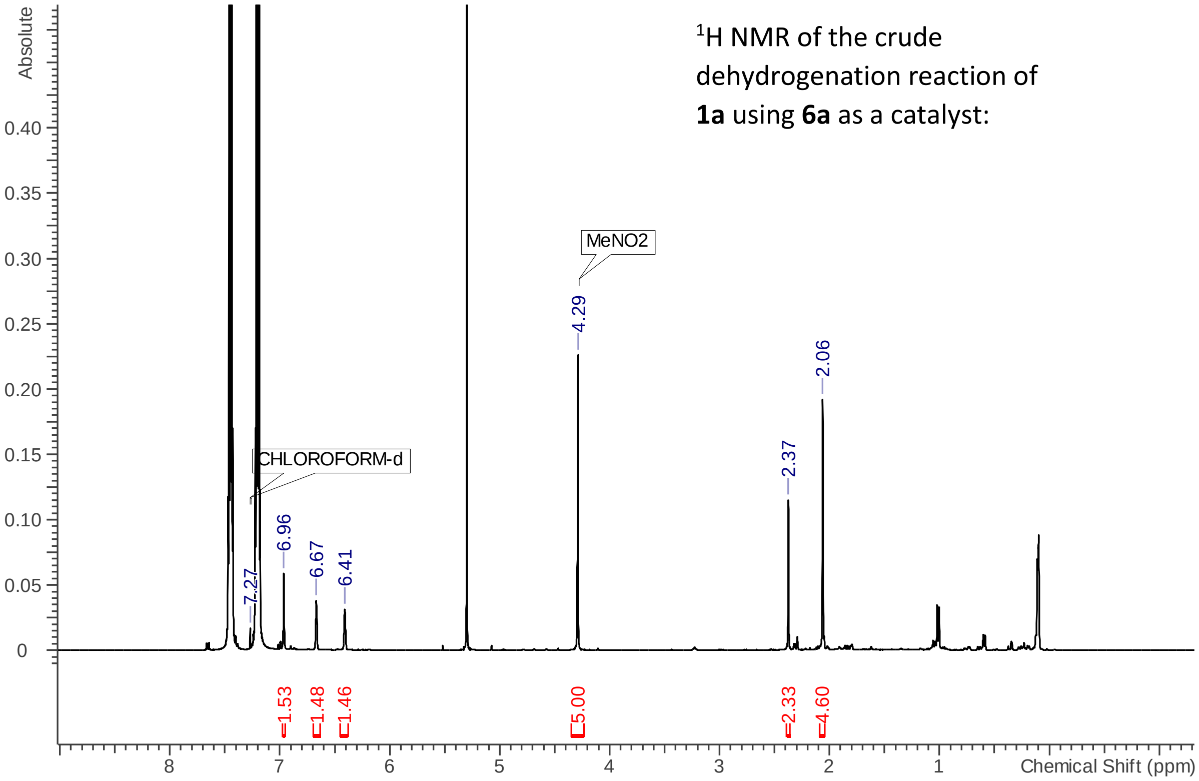

Using 8e:

The reaction was set up according to general procedure 9 using tris(pentafluorophenyl)((1-phenyl-3,4-dihydro-2H-pyrrol-1-ium-5-yl)methyl)borate **8e** (26.8 mg, 0.04 mmol), *N*-mesitylpyrrolidine **1a** (37.9 mg, 0.2 mmol) and methallyltrimethylsilane **2a** (88 µL, 0.5 mmol) formed *N*-mesitylpyrrole **3a** with an NMR yield of 99%, and 2-methyl-1-phenyl-pyrrole, **3e** with an NMR yield of 38%.

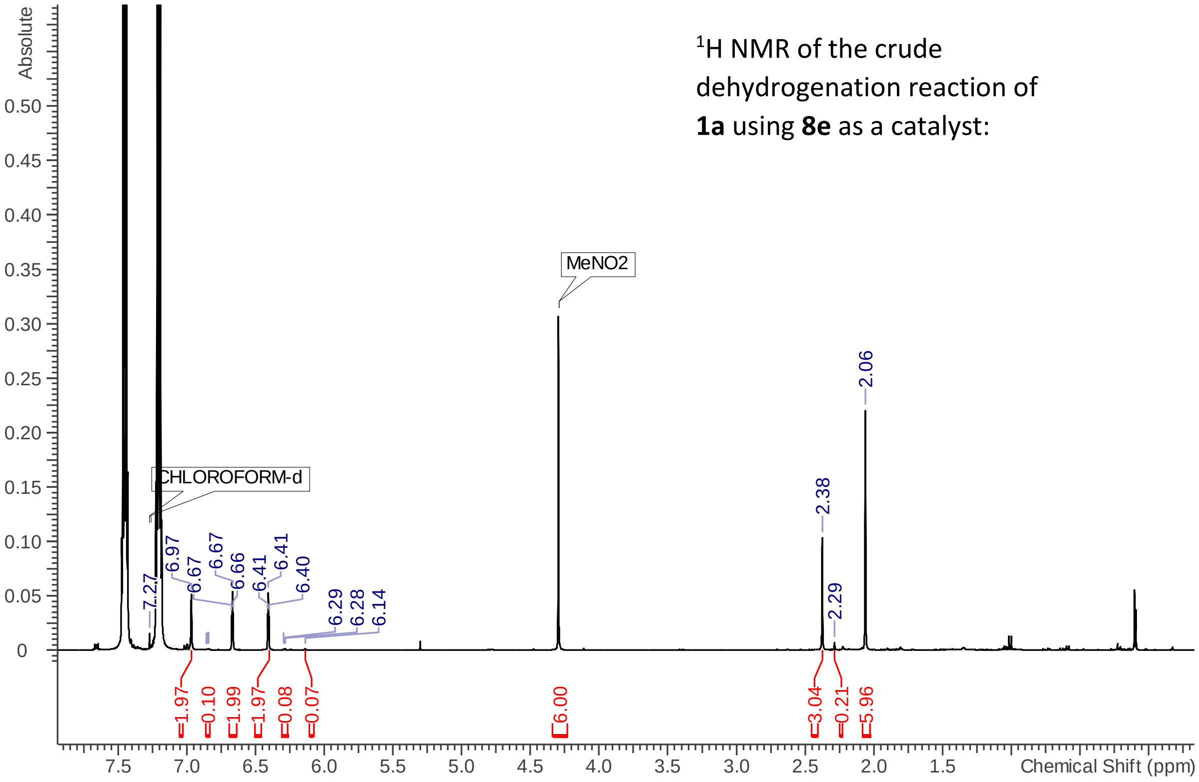

Reduction of methallyltrimethylsilane with 1-(2-chlorophenyl)pyrrolidin-1-ium trispentafluorophenylhydridoborate, 6c

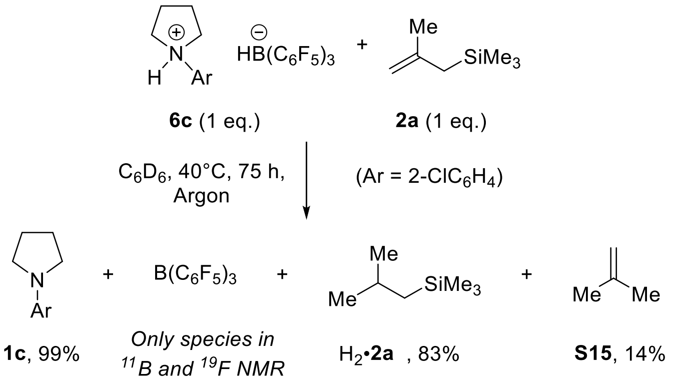

An NMR tube equipped with a J. Youngs tap was charged with 1-(2-chlorophenyl)pyrrolidin-1-ium trispentafluorophenylhydridoborate **6c** (24.3 mg, 0.035 mmol), C_6D_6 (0.7 mL) and cyclohexane (3.8 µL, 0.035 mmol) as an internal standard inside a glovebox. The NMR tube was sealed, and NMR taken prior to alkene addition. Subsequently, methallyltrimethylsilane **2a** (6.1 µL, 0.035 mmol) was added, and the reaction mixture was heated to 40 °C for 75 h (periodically monitored by NMR), before the final composition was analysed by NMR, showing a mixture of **1c** (0.035 mmol, 99%), $B(C_6F_5)_3$ (only species by ^{11}B and ^{19}F NMR), H_2·**2a** (0.029 mmol, 83%) and **S15** (0.005 mmol, 14%).

$B(C_6F_5)_3$: ^{11}B NMR (160 MHz, C_6D_6) δ_B = 58.02 (br. s); **^{19}F NMR** (376 MHz, C_6D_6) δ_F = −128.62–−128.85 (6F, m), −141.64 (3F, app. tt, *J* = 20.9, 6.2 Hz), −159.85–−160.06 (6F, m).

S15: ^{1}H NMR (400 MHz, C_6D_6) δ_H = 4.75 (2H, spt, *J* = 1.2 Hz), 1.60 (6H, t, 1.2 Hz).

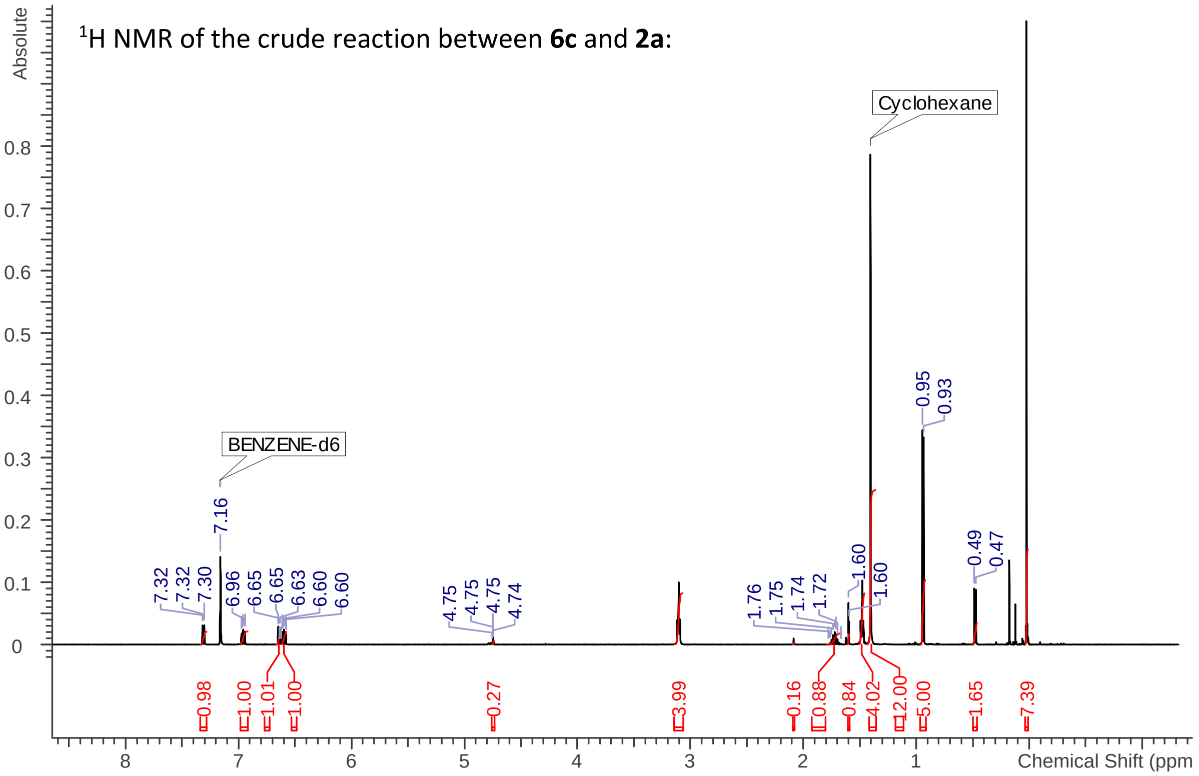

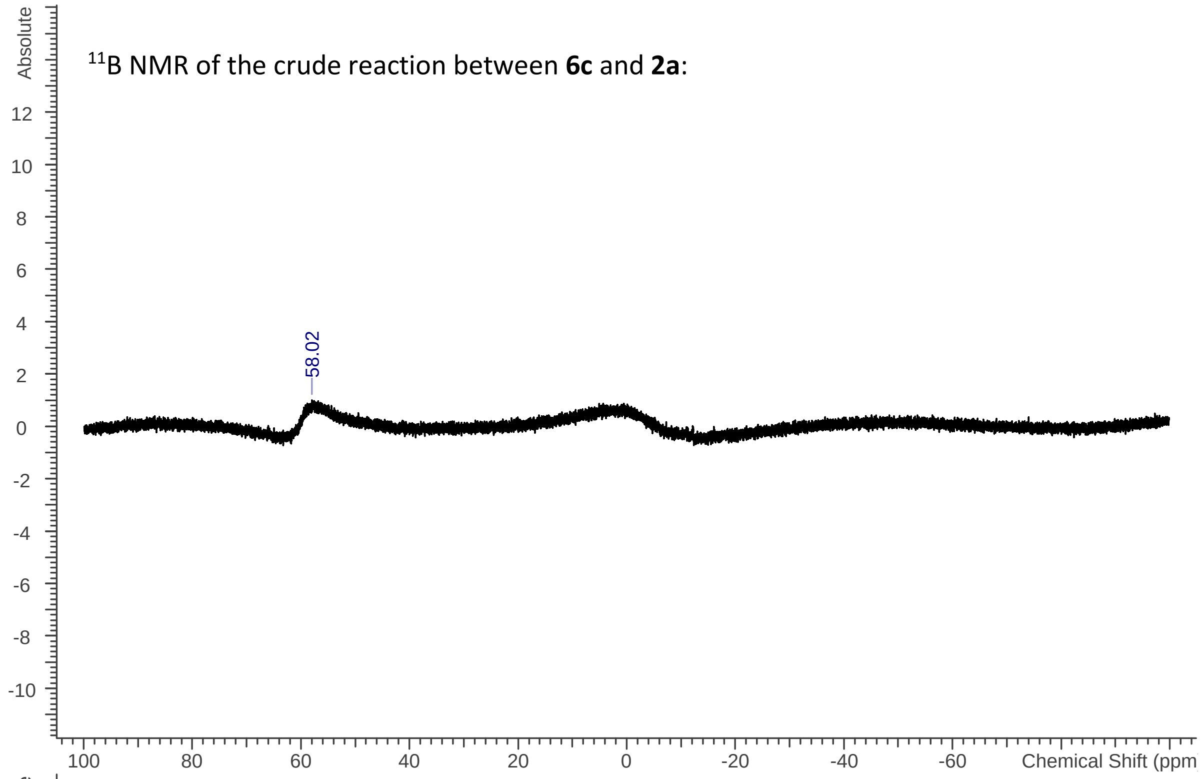

^{11}B NMR of the crude reaction between **6c** and **2a**:
58.02
Absolute
Chemical Shift (ppm

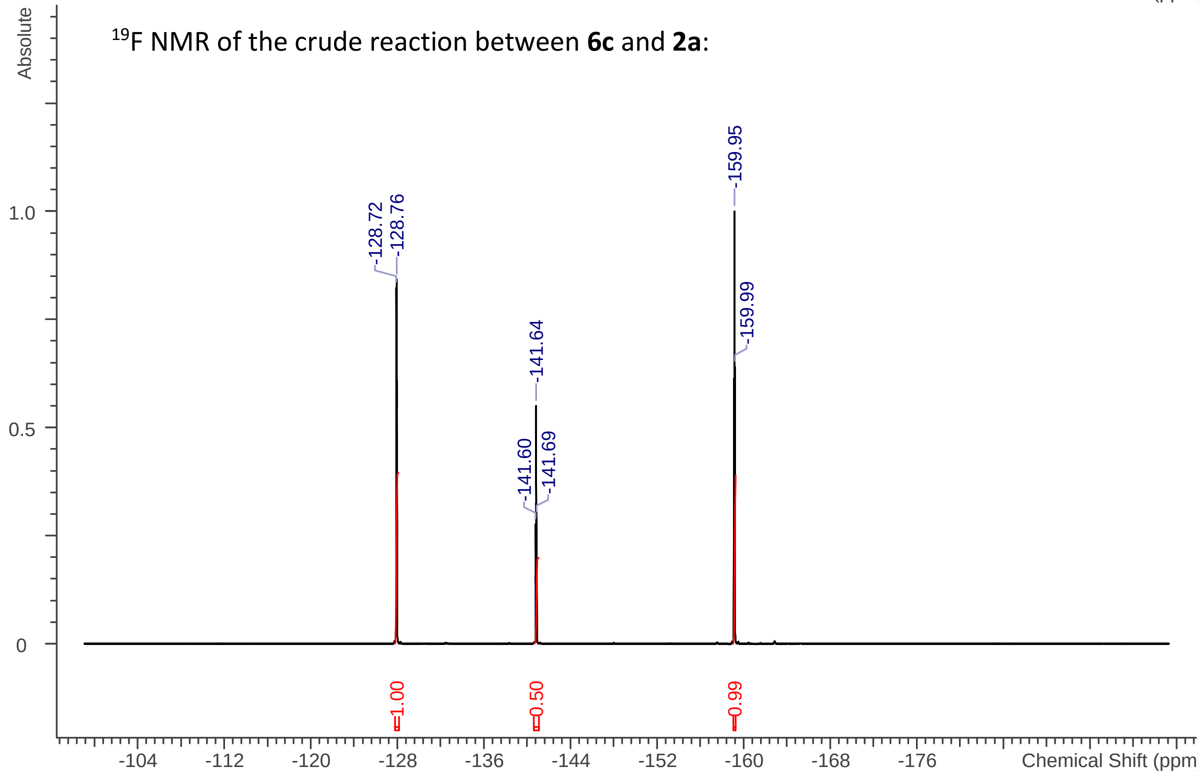

^{19}F NMR of the crude reaction between **6c** and **2a**:
-128.72
-128.76
-141.64
-141.60
-141.69
-159.95
-159.99
1.00
0.50
0.99
Absolute
Chemical Shift (ppm

6. DFT calculations

6.1 Computational methods

All calculations were performed using Gaussian 16 package.[19] The molecular geometries were optimized using B3LYP-D3 exchange-correlation functional.[20] 6-311G (d,p) basis set was used for all atoms. The vibrational analysis was calculated with the keyword "Freq". The electronic energies were further refined with single point energy calculations with M06-2x exchange-correlation functional and same basis set. During the point energy calculation, solvent effects of O-dichlorobenzene were taken into account using SMD model.[21] The thermal correction to Gibbs free energy at 413.15K, and a concentration correction which is 2.89 kcal/mol from P = 1 atm to C = 1 mol/L condition in the solvent at 413.15K. The correction factor for zero-point energy is 0.9888.[22]

6.2 Cartesian coordinates of the B3LYP-D3 /6-311G(d,p) computed structures

$B(C_6F_5)_3$

scf done:−2208.898943

B	0.00009000	-0.00040300	0.00032200
C	-1.36161900	-0.77032700	0.00045500
C	-2.47529700	-0.31101600	0.71296100
C	-1.54238500	-1.96129000	-0.71223800
C	-3.68653500	-0.98537700	0.73245600
C	-2.74485400	-2.65115900	-0.73195300
C	-3.82098100	-2.16037200	0.00020500
C	0.01382100	1.56387800	0.00010900
C	-0.92712500	2.31558700	-0.71294700
C	0.96788500	2.29908900	0.71298900
C	-0.92380400	3.70189500	-0.73273000
C	0.98899600	3.68523400	0.73245600
C	0.03871000	4.38883300	-0.00021700
C	1.34782300	-0.79446000	0.00019100
C	2.46958700	-0.35442500	-0.71185800
C	1.50736900	-1.98901000	0.71195600
C	3.66869100	-1.05008400	-0.73166700
C	2.69744300	-2.70008700	0.73126200
C	3.78221600	-2.22785200	-0.00030400
F	-4.71968900	-0.52196500	1.43994300
F	-4.97872100	-2.81472800	0.00009100
F	-2.39472600	0.81427800	1.44063000
F	-2.88068700	-3.77522200	-1.43953600
F	-0.53672500	-2.47244100	-1.44004400
F	-1.87221800	1.69993200	-1.44100700
F	-1.82928900	4.38116500	-1.44080300
F	1.90212800	1.66711800	1.44108400
F	1.90638600	4.34860200	1.44027200
F	0.05043000	5.71865100	-0.00041100
F	2.40906300	0.77259600	-1.43880100
F	4.70997900	-0.60450600	-1.43866500
F	4.92818800	-2.90258300	-0.00059200
F	2.81320300	-3.82700700	1.43790200
F	0.49266700	-2.48303800	1.43900900

$HB(C_6F_5)_3^-$

scf done:−2209.610868

B	0.04607400	-0.06257500	-0.89184600
C	-1.22173600	-0.96282400	-0.38104400
C	-1.60355200	-1.02096600	0.95586700
C	-2.02652000	-1.68288000	-1.25679400
C	-2.70467100	-1.73110700	1.41291700
C	-3.14136600	-2.40812400	-0.84363000
C	-3.48328800	-2.43065800	0.49990500
C	-0.19254000	1.47569400	-0.36548600
C	-1.15088100	2.26245000	-1.00161500
C	0.45769500	2.09417500	0.69765000
C	-1.43934400	3.57200500	-0.63644000
C	0.20364100	3.40288600	1.09534800
C	-0.75278200	4.14874400	0.42281300
C	1.51866000	-0.66254800	-0.50174600
C	2.65553600	0.03431100	-0.91269300
C	1.78537900	-1.86096700	0.15226200
C	3.95476000	-0.39936900	-0.69316100
C	3.07238300	-2.33660100	0.39345200
C	4.16691500	-1.60126400	-0.03080400
F	-3.02786000	-1.75890800	2.72135700
F	-4.55784800	-3.12726700	0.91710700
F	-0.86754600	-0.37901000	1.88990700
F	-3.89434300	-3.09068400	-1.73043000
F	-1.75470700	-1.72006300	-2.57782600
F	-1.86526000	1.75890500	-2.03155000
F	-2.37479100	4.28936600	-1.29171700
F	1.39272200	1.43356500	1.41444500
F	0.86847100	3.95573000	2.12975200
F	-1.01520300	5.41644200	0.79510400

F	2.52128500	1.21319000	-1.56078900
F	5.01242000	0.32415800	-1.11237000
F	5.41879400	-2.04562800	0.19257800
F	3.26704200	-3.50670900	1.03462400
F	0.78415500	-2.65420300	0.60037900
H	0.01391200	-0.02482100	-2.09670700

1a

scf done:−561.750130

C	0.78420700	1.20577100	0.05067500
C	2.18019700	1.20573900	0.05377000
C	2.91809300	0.02302900	0.01045100
C	2.21978100	-1.18375600	-0.03577400
C	0.82535400	-1.22760200	-0.05907300
C	0.09452300	-0.02194600	-0.02062300
H	2.70466000	2.15558700	0.11277400
H	2.77527300	-2.11696300	-0.06854000
C	-2.13153400	0.46291000	-1.12789100
C	-2.10822100	-0.43257400	1.13477500
C	-3.53799900	-0.03076000	-0.76663800
H	-1.78500000	0.08014800	-2.09396700
H	-2.12027800	1.56302200	-1.18949400
C	-3.52940100	0.02888100	0.76980000
H	-2.08314700	-1.51642300	1.32251500
H	-1.73627900	0.06038700	2.04173000
H	-3.66760300	-1.06441500	-1.10211800
H	-4.30375300	-0.58757200	1.23072600
N	-1.31895500	-0.06581900	-0.03946200
C	0.11004000	-2.55340800	-0.13155700
H	0.75971300	-3.32947400	-0.54158800
H	-0.78587500	-2.47581100	-0.75105200
H	-0.21489000	-2.88612900	0.86050800
C	0.03365400	2.51076300	0.15449400
H	0.65986400	3.28568900	0.60143800
H	-0.86628100	2.39770300	0.76306000
H	-0.28578900	2.87257100	-0.82878600
C	4.42768500	0.04910300	-0.01346400
H	4.85063300	-0.84058300	0.45960900
H	4.82013100	0.92727100	0.50536800
H	4.80282800	0.08240200	-1.04246400
H	-3.68212000	1.06153900	1.09911400
H	-4.32768900	0.57487900	-1.21626000

9

scf done:−560.925981

C	-0.77215500	1.23127100	0.05569800
C	-2.16473100	1.19888700	0.08438800
C	-2.88144900	0.00128700	0.00483600
C	-2.17003300	-1.19570100	-0.10639200
C	-0.77640900	-1.23051900	-0.12810000
C	-0.10879800	-0.00086800	-0.05201200
H	-2.70503100	2.13520600	0.17086500
H	-2.71365400	-2.13109800	-0.18352600
C	2.08383300	0.42857500	-1.01656600
C	2.13840700	-0.38367100	1.14665400
C	3.53122500	0.41493500	-0.68402500
H	1.63366700	0.77573500	-1.94001800
C	3.57989700	-0.42904200	0.61484100
H	1.96406200	0.39039700	1.89813900
H	1.76449200	-1.32959400	1.53205000
H	3.85763000	1.45325800	-0.53626600
H	4.29463300	-0.04120300	1.33798200
N	1.34141800	-0.00019300	-0.06060700
C	-0.04712000	-2.54787300	-0.23863100
H	-0.66249800	-3.28218300	-0.75888100
H	0.17475500	-2.96067200	0.75112300
H	0.89465600	-2.46311900	-0.78560700
C	-0.03070600	2.54498000	0.13840200
H	-0.69195600	3.33374300	0.49568200
H	0.34508800	2.86091500	-0.84020900
H	0.82101000	2.49903000	0.82302300
C	-4.38639900	0.00079200	0.06555400
H	-4.80826600	-0.85193500	-0.46900500
H	-4.80422800	0.91503600	-0.35958500
H	-4.72274100	-0.06253600	1.10559600
H	3.86236600	-1.45586900	0.37964000
H	4.13271600	0.01793100	-1.50463400

10a

scf done:−560.524286

C	0.64571600	1.17286400	0.04736400
C	2.03913100	1.24765200	0.10519600
C	2.84521500	0.11119000	0.04904000

C	2.22082500	-1.13126900	-0.06445400
C	0.83358300	-1.25369600	-0.14130700
C	0.04052800	-0.09094400	-0.09566800
H	2.50542800	2.22254800	0.21621500
H	2.82968600	-2.02987900	-0.10396200
C	-2.19219000	0.49129300	-1.03105800
C	-2.15514300	-0.50782600	1.05895500
C	-3.47270900	0.52430400	-0.64547500
H	-1.75316500	0.91198000	-1.92531000
C	-3.62518400	-0.30422700	0.61654100
H	-1.95183300	-1.50786100	1.44539800
H	-1.87454400	0.21911200	1.83466700
H	-4.28989100	0.94977400	-1.20737800
H	-4.10735200	-1.26581800	0.39500900
N	-1.37385400	-0.24397100	-0.16990700
C	0.19526300	-2.61411800	-0.27086500
H	0.91523300	-3.35238800	-0.62963300
H	-0.65470600	-2.58118300	-0.95483000
H	-0.18470100	-2.96785300	0.69395900
C	-0.17210100	2.43945900	0.15733500
H	0.41603800	3.23361600	0.62142000
H	-1.07618700	2.29081200	0.74935200
H	-0.49645000	2.80003200	-0.82390400
C	4.35037900	0.22321400	0.08429700
H	4.80512700	-0.64985000	0.55872600
H	4.67208300	1.11251200	0.63169000
H	4.76060900	0.29589400	-0.92904200
H	-4.21828000	0.17897700	1.39758500

6a

scf done:−562.139372

C	-1.00160800	-1.29370200	0.01893900
C	-2.37294700	-1.05860700	0.02034300
C	-2.89850600	0.23585500	0.00513200
C	-2.00653900	1.30642200	-0.00799700
C	-0.61878000	1.13621800	-0.01489500
C	-0.14401000	-0.18108100	-0.00208800
H	-3.04879400	-1.90633300	0.03980700
H	-2.39763800	2.31816800	-0.01172500
C	2.12036200	-0.05485800	1.25208800
C	2.12261400	-0.05639500	-1.21902900
C	3.45785200	0.46648000	0.70773600
H	1.53587600	0.67651200	1.80133400
C	3.55849500	-0.11602800	-0.71418800
H	1.87863100	-0.73214800	-2.03768600
H	1.81997700	0.95108400	-1.48794300
H	3.45168300	1.55761800	0.66699900
H	3.91415500	-1.15062600	-0.68968200
N	1.31219600	-0.48093100	0.00374700
C	0.24262400	2.37991600	-0.06704000
H	-0.24356400	3.19124900	0.47599000
H	0.36825600	2.71982200	-1.10020100
H	1.23572200	2.25922000	0.36205300
C	-0.47689200	-2.71377400	0.04533200
H	-1.30191600	-3.42477900	0.05452500
H	0.11914000	-2.92344000	0.94184300
H	0.12499000	-2.95529100	-0.83951100
C	-4.38756800	0.46083100	-0.01775400
H	-4.64884100	1.45993300	0.33380100
H	-4.90894900	-0.26969500	0.60408700
H	-4.77133200	0.35624300	-1.03764600
H	4.23426400	0.44948200	-1.35538000
H	4.29051700	0.16599900	1.34298100
H	2.22746800	-0.94555300	1.87016500
H	1.36440700	-1.49859800	0.00049200

7a

scf done:−2769.464687

C	-4.54442100	1.25098900	-1.96640600
C	-5.90424300	1.46734400	-1.75123200
C	-6.63523600	0.71537800	-0.82798600
C	-5.97452900	-0.28328900	-0.11344200
C	-4.61504900	-0.54482200	-0.29194900
C	-3.91887200	0.24119700	-1.21704900
H	-6.40626200	2.24188000	-2.32167300
H	-6.52614900	-0.87633600	0.60874800
C	-1.54904400	0.80200200	-1.11562100
C	-2.00586300	-1.16481400	-2.23815700
C	-0.23837300	0.39927000	-1.57009800
H	-1.76479100	1.68350200	-0.52538400
C	-0.55469600	-0.76029800	-2.55738400
H	-2.07002800	-2.05311000	-1.60892800

H	-2.64694900	-1.30396800	-3.10916900
H	0.23259400	1.24085000	-2.06862700
H	0.12402600	-1.60043600	-2.44830200
N	-2.51431500	0.00173800	-1.45357500
C	-3.93553700	-1.63695700	0.49016100
H	-4.40759400	-1.77415100	1.46400800
H	-2.88541400	-1.41091200	0.65685600
H	-3.98895300	-2.59442900	-0.03830100
C	-3.78336500	2.07799600	-2.97561000
H	-4.47041300	2.65641500	-3.59350100
H	-3.17861700	1.45359200	-3.63890500
H	-3.10333500	2.78527200	-2.49096100
C	-8.09694000	1.00049200	-0.58952000
H	-8.62358900	0.11498100	-0.22832100
H	-8.59018700	1.34389900	-1.50149000
H	-8.21618800	1.78587600	0.16419000
H	-0.48005800	-0.39596200	-3.58412400
B	0.81688300	0.06897100	-0.25121000
C	1.35834600	1.46054100	0.46554600
C	2.28834000	1.34742000	1.50258800
C	1.03992300	2.77470600	0.14115800
C	2.87021200	2.42593300	2.15244000
C	1.59496800	3.89017600	0.75990500
C	2.52305900	3.71645800	1.77381500
C	2.17868200	-0.63869200	-0.87657300
C	2.80497700	-0.09771400	-2.00265000
C	2.87076900	-1.71429600	-0.31217600
C	3.97613400	-0.58903800	-2.56460200
C	4.04676800	-2.23851300	-0.83949200
C	4.60424300	-1.67756700	-1.97829900
C	-0.02154700	-0.88037200	0.81620500
C	-0.24232000	-2.24224500	0.60953400
C	-0.61399200	-0.38720200	1.98025700
C	-0.90390400	-3.07432300	1.50116400
C	-1.28494800	-1.18022600	2.90690100
C	-1.42265000	-2.53929800	2.67183100
F	3.75362500	2.23758100	3.14094400
F	3.06916600	4.77354400	2.38385000
F	2.64762600	0.12440500	1.94184400
F	1.23309400	5.12683600	0.38793900
F	0.11088500	3.06306000	-0.81663900
F	2.28692300	0.99437600	-2.62156600
F	4.50523400	-0.01741600	-3.65607100
F	2.43957800	-2.31464400	0.81253600
F	4.65063500	-3.27942100	-0.25068900
F	5.73346700	-2.17062400	-2.49736300
F	0.18955400	-2.83929900	-0.52817000
F	-1.08473900	-4.37301800	1.22279700
F	-2.10453300	-3.31017300	3.52621400
F	-1.83519400	-0.63599300	4.00058600
F	-0.58950600	0.93463200	2.26438800

11

scf done:−559.690003

C	0.71720500	-1.23525800	-0.06782300
C	2.10987400	-1.19717600	-0.05411700
C	2.81756400	0.00785400	0.00156400
C	2.09943800	1.20606300	0.03874100
C	0.70519400	1.23387200	0.02317700
C	0.05255100	-0.00304900	-0.03436200
H	2.65642500	-2.13313400	-0.08992500
H	2.63740600	2.14648500	0.08430100
C	-2.19294500	-0.24919900	1.14194500
C	-2.16641100	0.21581900	-1.05936900
C	-3.48602100	-0.15988800	0.81884800
C	-3.58649300	0.15143600	-0.64358900
H	-1.75573000	0.41391500	-2.03980200
H	-4.10279000	1.09908000	-0.85234600
N	-1.40217300	-0.01194700	-0.03516300
C	-0.05311600	2.53840000	0.06569200
H	0.61599100	3.36337900	0.30739000
H	-0.84725900	2.52558700	0.81775000
H	-0.51471000	2.76811400	-0.90022600
C	-0.03614500	-2.54023400	-0.14168700
H	0.65312500	-3.38327800	-0.16565800
H	-0.65655100	-2.59543000	-1.04178800
H	-0.69707200	-2.67933200	0.71928100
C	4.32313300	0.01007900	0.05169200
H	4.73637000	0.93894100	-0.34457400
H	4.74222300	-0.82301600	-0.51555800
H	4.66735600	-0.09044300	1.08616200
H	-4.13099500	-0.61096800	-1.21810400

H	-4.32718500	-0.28203200	1.48338300
H	-1.68435200	-0.44655100	2.07159500

12

scf done:−559.697351

C	0.69020500	1.22834900	0.04694800
C	2.08241100	1.21079500	0.08139500
C	2.81084500	0.01827000	0.02525100
C	2.11154000	-1.18722600	-0.06264700
C	0.71785200	-1.23486300	-0.09444800
C	0.03743700	-0.01125700	-0.04209200
H	2.61349600	2.15333900	0.15737700
H	2.66417100	-2.11925000	-0.11063800
C	-2.18452200	0.31597300	-1.05458400
C	-2.23762000	-0.35329800	1.10878000
C	-3.57374900	0.23704500	-0.68599100
C	-3.62282000	-0.16608500	0.60342500
H	-1.98096800	0.31801600	1.93595600
H	-4.50547300	-0.34153000	1.20217200
N	-1.41020500	-0.02019400	-0.05858700
C	0.00038100	-2.56094200	-0.17519500
H	0.63021800	-3.30750600	-0.65931800
H	-0.93126200	-2.50053500	-0.74266300
H	-0.23636000	-2.94445800	0.82319500
C	-0.06983100	2.53195500	0.10413300
H	0.58775400	3.34569000	0.40804400
H	-0.90001900	2.49744900	0.81552200
H	-0.48532500	2.80042900	-0.87243000
C	4.31695300	0.03961600	0.02992000
H	4.73069100	-0.89760500	0.40530500
H	4.70180700	0.85634400	0.64352300
H	4.69662800	0.18562900	-0.98658100
H	-4.39812100	0.45917800	-1.34607300
H	-1.76264400	0.60262100	-2.00952200
H	-2.02765000	-1.37645100	1.43685900

3a

scf done:−559.333583

C	0.67697900	1.22406300	-0.00382200
C	2.07248200	1.20001600	-0.00967500
C	2.78621900	0.00008100	-0.01043400
C	2.07263100	-1.19992600	-0.00982300
C	0.67712800	-1.22413100	-0.00395600
C	-0.00452000	-0.00007800	-0.00016900
H	2.61391200	2.14107100	-0.01557000
H	2.61416900	-2.14091800	-0.01585800
C	-2.24018800	0.00016100	-1.11809500
C	-2.23612800	-0.00027900	1.12544700
C	-3.55335400	0.00021200	-0.70554300
C	-3.55078500	-0.00005900	0.71763600
H	-1.79327800	-0.00046700	2.10820000
N	-1.43681900	-0.00013900	0.00222400
C	-0.08627300	-2.52487100	-0.00587300
H	0.59330400	-3.37844300	-0.00680000
H	-0.73635200	-2.59808600	-0.88180300
H	-0.73658700	-2.60017900	0.86968200
C	-0.08655200	2.52472200	-0.00569900
H	0.59294800	3.37835400	-0.00531900
H	-0.73789500	2.59934700	0.86913700
H	-0.73561700	2.59847400	-0.88235000
C	4.29572400	0.00018800	0.01868200
H	4.66421000	0.00019700	1.05029100
H	4.70473300	0.88479700	-0.47498300
H	4.70487400	-0.88433700	-0.47501800
H	-4.41744700	0.00039200	-1.35171600
H	-1.80088200	0.00029700	-2.10244200
H	-4.41253800	-0.00013200	1.36692700

2a

scf done:−566.025904

C	-2.35006200	-1.29592100	0.05239800
H	-1.88170400	-2.23939400	-0.20779000
H	-3.27052700	-1.34661600	0.62350600
C	-1.82164800	-0.12665200	-0.32027600
C	-0.52558900	-0.03076000	-1.08706200
H	-0.52263400	0.85894900	-1.72740900
H	-0.40680700	-0.89511100	-1.74985700
Si	1.03174500	0.01243900	0.01662300
C	1.21479300	-1.66311500	0.85994700
H	2.07107800	-1.67311600	1.54105200
H	1.36150100	-2.46212200	0.12667700
H	0.31833900	-1.90112900	1.43898200

C	0.89500400	1.36194900	1.32910600
H	0.05730300	1.17074100	2.00567200
H	0.75039300	2.34977600	0.88155600
H	1.80640500	1.40208700	1.93379100
C	2.52503700	0.35100400	-1.08864300
H	3.45381200	0.34533600	-0.50986900
H	2.44359100	1.32701300	-1.57691400
H	2.61779600	-0.40704400	-1.87243000
C	-2.48539700	1.18540600	0.01747100
H	-3.37376000	1.04558000	0.63635400
H	-2.78386200	1.70613100	-0.89991600
H	-1.79818100	1.85331600	0.54622600

13

scf done:−566.388262

C	-2.28370600	-1.27070100	0.05451300
H	-1.70678500	-2.15333700	-0.21559300
H	-2.47460000	-1.25769200	1.13138600
C	-1.66043900	-0.00007200	-0.40001200
C	-0.54426600	0.00038200	-1.22577700
H	-0.31663800	0.92106700	-1.76097600
H	-0.31640200	-0.91987200	-1.76162000
Si	1.09480700	0.00006500	0.10342100
C	0.97989500	-1.56497400	1.10904900
H	1.89177900	-1.65781500	1.70868700
H	0.91566000	-2.45720200	0.48120700
H	0.13665000	-1.56328100	1.80321200
C	0.98070700	1.56639800	1.10715000
H	0.13726000	1.56582600	1.80107700
H	0.91670900	2.45774900	0.47801200
H	1.89246600	1.65997500	1.70681000
C	2.47589300	-0.00123800	-1.15069000
H	3.42977000	-0.00125100	-0.61193100
H	2.45479700	0.88636600	-1.78700000
H	2.45413200	-0.88962800	-1.78587700
C	-2.28439300	1.27008300	0.05492100
H	-3.27007700	1.34359500	-0.42445900
H	-1.70727100	2.15300000	-0.21381700
H	-2.47681100	1.25624300	1.13149200
H	-3.27008000	-1.34391500	-0.42343700

H_2·**2a**

scf done:−567.256669

C	-3.07798200	-0.92408700	-0.11550500
H	-2.97435700	-1.98260600	0.14013700
H	-3.95611100	-0.53415800	0.40893500
C	-1.81104700	-0.13617100	0.24968400
C	-0.57301400	-0.73792900	-0.45102300
H	-0.70430200	-0.66061900	-1.53967500
H	-0.53988900	-1.81475900	-0.23825600
Si	1.14615600	-0.05593300	-0.00651700
C	1.22422800	0.32435000	1.84260700
H	2.22134200	0.67080300	2.13102900
H	0.99619800	-0.56499900	2.43832100
H	0.50827900	1.10274700	2.12219500
C	1.56106400	1.50015400	-0.99634400
H	0.88748900	2.32973400	-0.76794000
H	1.49457200	1.30614200	-2.07149400
H	2.58227600	1.83185000	-0.78317400
C	2.42172000	-1.38436100	-0.42994000
H	3.43875300	-1.03934700	-0.21957800
H	2.37548400	-1.65224000	-1.49024600
H	2.25189000	-2.29641600	0.15048100
C	-1.99989700	1.35251300	-0.07420600
H	-2.08310800	1.50462800	-1.15586600
H	-1.16439400	1.95916700	0.28304100
H	-2.91112000	1.74203800	0.38905400
H	-3.27405300	-0.85890000	-1.19145400
H	-1.66558400	-0.22681100	1.33409300

TS1

scf done:−2770.661354

C	3.53356900	-1.53329200	1.68769400
C	4.74947100	-1.53364300	1.00582300
C	5.34168700	-0.35535800	0.55215600
C	4.70523200	0.85206600	0.83209800
C	3.48314100	0.91069900	1.49953600
C	2.89472100	-0.29807500	1.90192800
H	5.24409000	-2.48200700	0.82169600
H	5.14905200	1.77831400	0.48253900
C	1.37300200	0.28112400	3.87643400
C	0.54087000	-0.93279100	2.04820800

C	-0.12268700	0.03399200	4.13049800
H	1.64441100	1.33469600	3.90494000
H	2.02220500	-0.26014500	4.57214100
C	-0.50191400	-1.07108900	3.12304500
H	0.69389900	-1.67134700	1.27749600
H	-0.69167300	0.93669400	3.91811800
H	-1.51704300	-0.98401600	2.74297600
N	1.59911000	-0.28557900	2.52499700
C	2.82065200	2.24949200	1.71180600
H	3.15814200	2.71220300	2.64561300
H	1.73634600	2.17279800	1.74473200
H	3.07932800	2.92859700	0.89852700
C	2.95484100	-2.84605300	2.16468900
H	3.75354100	-3.56787800	2.34102600
H	2.28045500	-3.28771800	1.42361000
H	2.39369500	-2.73594100	3.09523500
C	6.60307400	-0.39226900	-0.27109700
H	7.19598800	0.51554700	-0.14100000
H	6.34699500	-0.47269400	-1.33271300
H	7.22743400	-1.25088100	-0.01426300
H	-0.39673800	-2.07569800	3.54950000
H	-0.31631900	-0.25900100	5.16202500
B	-0.90965600	0.04033000	-0.18089100
C	-2.32883600	-0.68503400	0.11249000
C	-2.38256700	-2.02066100	0.51153600
C	-3.57366300	-0.06084800	0.02215600
C	-3.55022800	-2.70636400	0.80070800
C	-4.77485700	-0.70968400	0.29937600
C	-4.76518400	-2.03978100	0.69203800
C	0.08540700	-0.67572700	-1.24702500
C	1.39043900	-0.19635700	-1.36495500
C	-0.21156400	-1.75917700	-2.07247700
C	2.36114500	-0.76702900	-2.17456800
C	0.72733000	-2.35995100	-2.90620200
C	2.02495000	-1.86758500	-2.95172400
C	-0.96489500	1.64295100	-0.38392500
C	-1.15279900	2.21311700	-1.63925600
C	-0.79767000	2.54326700	0.65532000
C	-1.15649700	3.58444000	-1.85711100
C	-0.77792000	3.92082200	0.48840700
C	-0.96109500	4.44626800	-0.78336700
F	-5.94000600	-0.05925900	0.19212700
F	-3.68252200	1.22630900	-0.35591000
F	-1.22852400	-2.72580700	0.64387600
F	-3.52108700	-3.98902800	1.18751700
F	-5.90846200	-2.67309300	0.96900900
F	-0.60726900	2.08799000	1.92532900
F	-0.57211800	4.73770200	1.53295700
F	-0.95043600	5.77019000	-0.97412400
F	-1.34434500	4.08714000	-3.08394300
F	1.76169000	0.89079400	-0.65471600
F	3.60741000	-0.26994600	-2.21733800
F	-1.45172600	-2.28036800	-2.11151500
F	2.93917700	-2.44060100	-3.74178200
F	0.39098700	-3.40800800	-3.66945600
F	-1.35919800	1.41907500	-2.70842300
H	-0.25435600	-0.09676300	0.94937900

TS2

scf done:−1122.677889

C	3.87222500	-0.73786400	0.74719900
C	5.05011100	-0.24345300	0.19085700
C	5.05412300	0.84465200	-0.68657300
C	3.83495500	1.44155900	-1.00741500
C	2.62637600	0.98401400	-0.47905400
C	2.66523100	-0.11285700	0.39165900
H	5.99093200	-0.71651500	0.45217300
H	3.81980800	2.28460600	-1.69060500
C	0.81421600	-1.72083100	0.60394400
C	0.67098200	0.09014300	2.02043300
C	-0.39871400	-1.94032100	1.28242300
H	1.25018500	-2.33444000	-0.17554500
C	-0.30423300	-0.99905200	2.50372500
H	1.34951600	0.46926700	2.78385000
H	0.13896500	0.93237200	1.56779200
H	-0.69455000	-2.97556500	1.44097000
H	0.11831300	-1.54201500	3.35555400
N	1.44405100	-0.61363300	0.96408000
C	1.33340500	1.67480500	-0.83582300
H	1.38956900	2.11287100	-1.83368100
H	1.11430400	2.48818200	-0.13662500
H	0.47963100	0.99762800	-0.81023100

C	3.90073200	-1.90720500	1.70185100
H	4.92223200	-2.12770300	2.01165400
H	3.49417500	-2.81486700	1.24458700
H	3.31081700	-1.70745500	2.60027400
C	6.35056300	1.37438400	-1.24509800
H	6.18961700	1.94762500	-2.15978900
H	7.04864000	0.56433800	-1.46718800
H	6.83723600	2.03580400	-0.52099000
H	-1.24640300	-0.56553400	2.82332400
H	-1.44943100	-1.56069000	0.29540000
C	-2.24530600	1.30341300	-1.23880800
C	-2.42677100	2.59984400	-0.74141400
C	-2.88914500	2.86837800	0.54188700
C	-3.22628800	1.77675300	1.33726600
C	-3.06093600	0.46040700	0.90824400
C	-2.51390600	0.21906800	-0.37502000
H	-2.21137100	3.43203700	-1.40268300
H	-3.64272800	1.94949300	2.32430800
C	-1.30556600	-1.56762200	-1.81571900
C	-3.40403400	-2.08437000	-0.92439100
C	-1.35832900	-3.09868900	-1.81327000
H	-0.31519500	-1.14458100	-1.65013200
C	-2.79563900	-3.43462100	-1.33358400
H	-4.02683400	-2.14003600	-0.03986300
H	-3.99347200	-1.63904800	-1.73060200
H	-0.62066200	-3.51800900	-1.12806500
H	-2.77337200	-4.12355000	-0.48842200
N	-2.21617600	-1.18971100	-0.69066600
C	-3.57977500	-0.62098400	1.83619600
H	-3.56178300	-0.27255700	2.86961800
H	-3.02388000	-1.55569400	1.79472000
H	-4.62516600	-0.84455300	1.60112900
C	-1.83935000	1.21507800	-2.69854400
H	-1.86609700	2.21247400	-3.13661900
H	-2.53023900	0.59987700	-3.27841000
H	-0.82911900	0.83140400	-2.84843900
C	-3.03010100	4.27981300	1.05044900
H	-3.91001400	4.38656600	1.68840300
H	-3.11466500	4.99495500	0.23042300
H	-2.15732100	4.56322100	1.64793900
H	-3.39263700	-3.90215100	-2.11630200
H	-1.14027000	-3.49304500	-2.80535800
H	-1.69279700	-1.18795300	-2.75497800

TS3

scf done:−1126.935409

C	-4.34689500	0.93231500	-0.21141600
C	-5.50776600	0.16869900	-0.33944900
C	-5.50739600	-1.21525200	-0.15889700
C	-4.29920600	-1.84385800	0.15374600
C	-3.11233200	-1.12684500	0.29133800
C	-3.15593500	0.26515500	0.11032100
H	-6.43836300	0.67122700	-0.58200000
H	-4.28088200	-2.92050500	0.28757900
C	-1.26216000	1.23791500	1.36094900
C	-1.15359800	1.46448000	-0.93932600
C	-0.01409500	1.80338200	1.15473000
H	-1.70275200	0.94707500	2.30606900
C	0.05930200	2.19770800	-0.31379800
H	-0.86171900	0.57735100	-1.51348500
H	-1.76170500	2.09280900	-1.58955100
H	0.98936200	0.55647600	1.11968800
H	1.00055300	1.89350100	-0.78704500
N	-1.94434800	1.02572500	0.23272100
C	-1.82034300	-1.83692100	0.62093700
H	-1.93147200	-2.91524100	0.50501200
H	-1.50576900	-1.64836600	1.65246700
H	-1.00525300	-1.51124900	-0.03139000
C	-4.38729100	2.42598900	-0.42145600
H	-5.39180400	2.81393200	-0.24907400
H	-4.11238900	2.68788700	-1.44893200
H	-3.70182700	2.95004500	0.24755900
C	-6.78412000	-2.00961100	-0.26866300
H	-6.59287400	-3.02878700	-0.61042700
H	-7.48743300	-1.54278300	-0.96081000
H	-7.27762600	-2.07692900	0.70646300
H	-0.01855700	3.28107500	-0.43786900
H	0.49818800	2.35216000	1.93438100
C	1.87622100	-0.33055500	1.06806000
H	2.44225400	-0.10918600	1.97136000
H	1.26916600	-1.23189600	1.14410700
C	2.55209900	-0.13238300	-0.15697200

C	2.03510500	-0.81597800	-1.38386400
H	2.24099300	-1.89069200	-1.32285200
H	2.48156900	-0.43479200	-2.30156000
H	0.94610200	-0.72166200	-1.44753500
C	3.73021000	0.70805300	-0.24621800
Si	5.39417800	-0.34414500	-0.07241300
C	5.35653900	-1.17685900	1.60771600
H	5.27148700	-0.44825700	2.41911200
H	6.28256300	-1.73682500	1.76865900
H	4.52756900	-1.88436500	1.69578100
C	5.46571800	-1.59635200	-1.46782200
H	6.43243700	-2.10878400	-1.45242700
H	5.36531700	-1.12127300	-2.44767100
H	4.69266100	-2.36382900	-1.37855400
C	6.76235300	0.93051000	-0.20593400
H	6.68835900	1.68631200	0.58060500
H	6.74419400	1.44116400	-1.17257200
H	7.73758300	0.44427800	-0.10715800
H	3.83062600	1.21253400	-1.21040200
H	3.80293000	1.42801200	0.57332400

TS4

scf done:−2769.421982

C	5.39835300	-0.14103200	-2.12098800
C	6.73176300	-0.33852000	-1.76301400
C	7.14608000	-0.32106200	-0.42995300
C	6.18876100	-0.09956800	0.56138400
C	4.84249500	0.10561900	0.25826300
C	4.46990700	0.08287000	-1.09240500
H	7.46240200	-0.51722500	-2.54511400
H	6.49490200	-0.08318400	1.60216600
C	2.54766700	1.31460100	-2.04105500
C	2.10999000	-0.84408200	-1.47956300
C	1.23835800	1.06800500	-2.48724900
H	3.12573200	2.22272700	-2.15019900
C	0.90932400	-0.21917800	-2.11380300
H	2.50210300	-1.66228600	-2.09412700
H	1.92226000	-1.23804600	-0.48152500
H	0.59869700	1.77432000	-2.98900200
H	0.06807100	-0.79127900	-2.46447900
N	3.08532600	0.25689300	-1.44913100
C	3.83410600	0.36180900	1.34488300
H	4.32021900	0.46883400	2.31411400
H	3.11159000	-0.45540200	1.42895100
H	3.26177200	1.26920300	1.15533100
C	4.98072700	-0.16525700	-3.57209300
H	5.78806000	-0.54431500	-4.19905400
H	4.72572300	0.83551700	-3.93428700
H	4.10436700	-0.79732000	-3.73778900
C	8.59871000	-0.50795500	-0.06946600
H	9.10396400	0.46092100	0.00309000
H	9.12520000	-1.09827900	-0.82219200
H	8.70857600	-1.00749400	0.89539700
H	-0.20922800	-0.04912300	-0.68532000
B	-1.22265700	-0.05253300	0.04641000
C	-1.98953500	1.31454200	-0.37608100
C	-2.17621900	1.62122300	-1.72002100
C	-2.49141100	2.26254600	0.51237400
C	-2.78190600	2.78224100	-2.17739500
C	-3.11272400	3.43784200	0.10313800
C	-3.25608900	3.70307700	-1.25232800
C	-2.01615900	-1.41211200	-0.34012900
C	-1.32484500	-2.59247300	-0.58512900
C	-3.40149000	-1.51401700	-0.44043900
C	-1.92987100	-3.79524300	-0.92029300
C	-4.05544800	-2.69544100	-0.77212100
C	-3.31434100	-3.84485000	-1.01612200
C	-0.59447700	-0.02821700	1.53906700
C	0.44060000	0.85513200	1.82667800
C	-1.02264100	-0.80476000	2.61187200
C	1.01729600	0.98724100	3.07995700
C	-0.46465100	-0.71643500	3.88375200
C	0.56547200	0.18411500	4.11949300
F	-3.57675200	4.31706900	1.00173500
F	-3.84351200	4.83310800	-1.66361300
F	-2.90488600	3.02545700	-3.49232100
F	-1.74711400	0.75311100	-2.67686500
F	0.03672700	-2.60880900	-0.49673700
F	-4.18353300	-0.44632300	-0.18845800
F	-5.39168700	-2.74112200	-0.85425400
F	-3.92743700	-4.98919400	-1.33999500
F	-1.19979300	-4.89719500	-1.15322400

F	2.02124800	1.85632300	3.29617900
F	0.93165500	1.65165700	0.83868700
F	1.12027200	0.27870800	5.33382200
F	-0.91173200	-1.48647600	4.88467300
F	-2.03904900	-1.67638000	2.46499700
F	-2.40943000	2.06705500	1.84410900

TS5

scf done:−2769.426394

C	-4.19882900	-0.01589700	-2.37932200
C	-5.43657100	-0.46510400	-1.92474800
C	-5.82353400	-0.32654500	-0.58940000
C	-4.94117500	0.28786200	0.29773500
C	-3.68692400	0.75042800	-0.10488000
C	-3.33802400	0.58389500	-1.44807000
H	-6.11686400	-0.93034300	-2.63064300
H	-5.22722300	0.40494300	1.33797200
C	-1.78750300	2.40883000	-2.28164700
C	-1.00543800	0.28616100	-2.20974500
C	-0.61900000	2.48317500	-2.92063300
C	-0.05055500	1.09442900	-3.03831100
H	-1.10200500	-0.78603300	-2.22217300
H	0.98818100	1.00401600	-2.72525500
N	-2.05791800	1.05097000	-1.92557900
C	-2.74014500	1.34128200	0.90419500
H	-3.28538400	1.84526900	1.70400400
H	-2.04275600	2.05405200	0.46492400
H	-2.14299100	0.54920600	1.36145900
C	-3.80173600	-0.17730700	-3.82699300
H	-4.66162200	-0.46283100	-4.43356000
H	-3.04237400	-0.95657700	-3.94727400
H	-3.38881900	0.74725800	-4.23942800
C	-7.15288600	-0.85785700	-0.11455500
H	-7.06002600	-1.90782200	0.18219500
H	-7.90907300	-0.80434200	-0.90094000
H	-7.51946200	-0.30277700	0.75133600
H	-0.10846500	0.72426900	-4.07311200
B	0.77211500	-0.05690200	0.10519200
C	2.14523000	-0.38023700	-0.70448300
C	2.13216300	-1.27697200	-1.76965000
C	3.40468800	0.12042800	-0.37706800
C	3.25940600	-1.64956400	-2.48880500
C	4.56259400	-0.22730000	-1.06421600
C	4.49031400	-1.11676600	-2.12895700
C	0.31848100	-1.37982300	0.92848100
C	-0.85748500	-2.08436200	0.72090200
C	1.16226400	-1.90935500	1.90238600
C	-1.19855700	-3.23475200	1.42342900
C	0.86783400	-3.05294000	2.63000200
C	-0.32614300	-3.72335200	2.38522800
C	0.73372800	1.34877300	0.91409300
C	0.25824600	1.51879800	2.21316000
C	1.11240000	2.53346000	0.28390300
C	0.15268700	2.75759600	2.83739000
C	1.02060500	3.79039700	0.86028100
C	0.53771300	3.90439000	2.15816800
F	5.74830600	0.28383000	-0.71038200
F	3.55596500	0.97525000	0.64919600
F	0.95584800	-1.83448400	-2.16456300
F	3.17216800	-2.50691400	-3.51616900
F	5.59439900	-1.45601900	-2.80212100
F	1.58666600	2.49788500	-0.98559000
F	1.38986500	4.88766900	0.18352900
F	0.43749100	5.10402400	2.73967600
F	-0.33532600	2.85509600	4.08143000
F	-1.76407300	-1.65867300	-0.19898400
F	-2.35435300	-3.87130400	1.18115500
F	2.31895300	-1.27735000	2.18518400
F	-0.63188000	-4.82863400	3.07413700
F	1.71031000	-3.51632500	3.56214300
F	-0.18878200	0.46776000	2.93290000
H	-0.10929300	0.11917600	-0.80668400
H	-0.14327100	3.37766900	-3.28810700
H	-2.50678700	3.16566000	-2.01273400

TS6

scf done:−2769.447791

C	-4.33105600	1.06482200	-2.08336100
C	-5.69037100	1.33994200	-1.93245200
C	-6.54528600	0.49420000	-1.22597700
C	-6.00700100	-0.66693500	-0.67470300
C	-4.65228600	-0.98344500	-0.78481000

C	-3.80724400	-0.09506500	-1.47438600
H	-6.09526800	2.23327000	-2.39825600
H	-6.65607800	-1.34861600	-0.13345900
C	-1.41583800	0.49363600	-1.34935900
C	-1.90011300	-1.49503100	-2.44023400
C	-0.19340300	0.08248600	-1.78806100
H	-1.64378400	1.41219900	-0.83391800
C	-0.36630300	-1.26026700	-2.47773100
H	-2.16604000	-2.46150200	-2.01515600
H	-2.36301100	-1.41784400	-3.42979500
H	0.15736200	-2.05776800	-1.94436200
N	-2.41493900	-0.39294000	-1.58890500
C	-4.14457800	-2.26888200	-0.18212300
H	-4.71746900	-2.53101200	0.70841100
H	-3.09777500	-2.20466600	0.10141800
H	-4.24788500	-3.09570600	-0.89383000
C	-3.48221200	1.99788900	-2.91758300
H	-4.10920500	2.54970700	-3.61978300
H	-2.72593200	1.45879500	-3.49150300
H	-2.95520300	2.73560300	-2.30440700
C	-8.00403400	0.83672500	-1.04913500
H	-8.61561200	-0.06184100	-0.94107900
H	-8.38578400	1.40837700	-1.89834000
H	-8.15284200	1.44546500	-0.15073600
H	0.01896000	-1.25009500	-3.49990700
B	1.30311400	0.05571500	0.36585000
C	2.04000700	1.43216800	0.06373400
C	1.34945300	2.63783300	-0.08947100
C	3.43103500	1.53961300	-0.03440100
C	1.96256300	3.84969500	-0.36158600
C	4.08659700	2.73719800	-0.29502300
C	3.34784600	3.89950600	-0.46609800
C	0.17369100	0.01285700	1.47645700
C	0.09530700	0.93552100	2.52345300
C	-0.78244900	-1.00715200	1.51800200
C	-0.87194700	0.87603300	3.52085500
C	-1.74872700	-1.11108000	2.50341700
C	-1.80278100	-0.15171600	3.50914500
C	2.02887300	-1.28983000	-0.05221500
C	2.65961300	-1.44565000	-1.29176900
C	2.13005000	-2.39742600	0.79668100
C	3.29629500	-2.60942100	-1.69159500
C	2.76963200	-3.57769000	0.43873600
C	3.35300900	-3.68718000	-0.81612700
F	1.62489200	-2.35913400	2.04238000
F	5.42045200	2.77976200	-0.37713700
F	3.96029000	5.05575100	-0.72129300
F	1.24091000	4.96602400	-0.51231400
F	-0.90245400	1.79077500	4.49586400
F	-2.73383400	-0.23139700	4.46049300
F	-2.62754100	-2.12202600	2.50977000
F	-0.77998200	-1.96420800	0.56633000
F	2.65694500	-0.43597300	-2.18401700
F	4.22036000	0.46612400	0.14652000
F	2.83737100	-4.60328700	1.29448900
F	3.96701300	-4.81414300	-1.17716700
F	3.85734900	-2.70278400	-2.90244800
F	0.99034300	1.93313900	2.63424600
F	0.00564100	2.66522300	0.03208000
H	0.63471200	0.74292100	-1.96605000

TS7

scf done:−1121.463991

C	2.81289900	-0.71366600	0.80255400
C	4.05344400	-1.07827700	1.33176200
C	5.25236400	-0.72713900	0.71218400
C	5.19968300	0.01106900	-0.47523500
C	3.99156100	0.39775100	-1.04929800
C	2.81391000	0.02671500	-0.38283900
H	4.07842700	-1.65299900	2.25160400
H	6.12514600	0.28672600	-0.96961000
C	0.93667100	1.57407100	-0.83334400
C	0.80255100	-0.42690000	-1.83365300
C	-0.31897300	1.54112700	-1.53608100
H	1.37562600	2.36193300	-0.24101600
C	-0.30403800	0.24041700	-2.22609900
H	1.16143400	-1.41911100	-2.05123300
H	-0.64814000	2.44331500	-2.04952500
N	1.54964800	0.40601400	-0.97897200
C	3.95218900	1.18599600	-2.33551900
H	4.94842200	1.26746500	-2.76941200
H	3.57837700	2.20149500	-2.17195900

H	3.29900700	0.71666300	-3.07614700
C	1.54040200	-1.08369600	1.52541000
H	0.67931100	-1.16862300	0.86022200
H	1.30404500	-0.33110100	2.28509900
H	1.65577800	-2.03661300	2.04407200
C	6.57979400	-1.15483200	1.28449200
H	6.98548000	-2.00010500	0.71956800
H	6.48830100	-1.46466500	2.32660100
H	7.31236600	-0.34623300	1.23082000
H	-1.06987600	-0.13427400	-2.88315700
H	-1.22672400	1.46311800	-0.65054200
C	-2.44964800	-1.11561400	1.26225900
C	-2.73813000	-2.43363600	0.89027400
C	-3.24337500	-2.78347500	-0.35692900
C	-3.49797400	-1.74676000	-1.24839400
C	-3.20884900	-0.41505900	-0.95097600
C	-2.63563600	-0.09005000	0.30440000
H	-2.58732000	-3.21432100	1.62832700
H	-3.93779300	-1.97613300	-2.21357000
C	-1.26011900	1.69631400	1.55307700
C	-3.31016200	2.35029200	0.48614200
C	-1.28492600	3.21843800	1.47618200
H	-0.28948100	1.23869600	1.35215300
C	-2.79295100	3.48934100	1.38119200
H	-3.46719100	2.69534200	-0.53334900
H	-4.24398800	1.91424400	0.84432400
H	-0.76889400	3.57900700	0.58076800
H	-3.03049300	4.47096900	0.97100100
N	-2.22720600	1.29557200	0.49874400
C	-3.59840800	0.60696800	-1.99993900
H	-3.70847500	0.12391100	-2.97171600
H	-2.88256400	1.41858900	-2.11714500
H	-4.56380800	1.06140100	-1.75975900
C	-2.04874500	-0.92251700	2.71304500
H	-2.18108400	-1.86234300	3.24876800
H	-2.67811300	-0.18396400	3.21391700
H	-1.00630000	-0.62769700	2.84237300
C	-3.51035000	-4.22062100	-0.72386000
H	-3.73648200	-4.82300500	0.15812300
H	-2.63503400	-4.66486000	-1.20947500
H	-4.34847600	-4.30606200	-1.41847600
H	-3.24129800	3.42790900	2.37555500
H	-0.81686000	3.68201200	2.34492000
H	-1.57953200	1.39067100	2.54462400

TS8

scf done:−1121.470246

C	1.50494600	1.10055200	1.07685600
C	2.13734600	2.15930300	0.42282800
C	3.16476700	1.96059000	-0.49722100
C	3.57820500	0.65064900	-0.75056300
C	2.99097800	-0.44677600	-0.12273900
C	1.93622400	-0.20276300	0.77600900
H	1.80265900	3.16907400	0.63522800
H	4.39529600	0.47748300	-1.44330900
C	0.54556300	-2.32640900	0.66499200
C	1.12173500	-1.53883800	2.69787300
C	0.05107400	-3.22079400	1.67658000
H	-0.42206700	-1.72944900	0.02209000
C	0.36380800	-2.70697900	2.91600000
H	1.53930800	-0.85571700	3.42147200
H	-0.49110000	-4.13200800	1.47036200
N	1.27543200	-1.32671900	1.38937200
C	3.52813700	-1.83242800	-0.40010800
H	4.58461100	-1.77887000	-0.66488300
H	3.01513400	-2.30847900	-1.24318800
H	3.43525400	-2.49138500	0.46482800
C	0.41225600	1.40677000	2.07005600
H	0.83026700	1.58399800	3.06620800
H	-0.32134000	0.60658000	2.15035300
H	-0.12498600	2.30655100	1.77197900
C	3.83898600	3.12529600	-1.17544500
H	4.75970300	3.39451700	-0.64803400
H	3.19729800	4.00814000	-1.18817300
H	4.11200300	2.88375600	-2.20495900
H	0.10173300	-3.10714300	3.88259800
C	-1.17423800	1.42378100	-1.12469300
C	-1.70525100	2.63675800	-0.66937500
C	-2.64012200	2.73608800	0.35477600
C	-2.98796900	1.55564300	1.00088800
C	-2.48840600	0.31459800	0.60881100
C	-1.63967900	0.22739700	-0.52317900

H	-1.35832400	3.54782500	-1.14510400
H	-3.64970600	1.59593200	1.86003000
C	-0.40594400	-1.32599400	-2.16862400
C	-2.45621600	-2.07019300	-1.15343000
C	-0.67347500	-2.76370800	-2.59577700
H	0.63133200	-1.11634700	-1.91046400
C	-2.19448100	-2.84343900	-2.44879700
H	-2.49078200	-2.74830000	-0.29949700
H	-3.38178000	-1.49833100	-1.17652200
H	-0.18192400	-3.48138800	-1.93177500
H	-2.57702600	-3.86312100	-2.39708500
N	-1.28005200	-1.12423900	-0.96783600
C	-2.85310100	-0.86063800	1.49591000
H	-3.04012800	-0.50182300	2.50901600
H	-2.05918400	-1.60215100	1.56378600
H	-3.76239700	-1.37072600	1.16781100
C	-0.10028300	1.56833700	-2.19141100
H	0.22044500	2.60921400	-2.22312800
H	-0.45417200	1.31908700	-3.19395700
H	0.78961300	0.98013400	-1.97597700
C	-3.23015600	4.06178300	0.75980100
H	-4.16345700	4.24635600	0.21805400
H	-2.55318300	4.88864300	0.53554400
H	-3.46189800	4.08799500	1.82676200
H	-2.67621600	-2.34728300	-3.29503400
H	-0.32143600	-2.95411600	-3.60992300
H	-0.70972500	-0.65959000	-2.97132700
H	1.07028900	-2.73387200	-0.19464800

TS9

scf done:−1128.158688

C	-1.65117500	1.17337100	-1.03336200
C	-1.22198400	2.50275700	-1.04124800
C	-0.89361700	3.18557200	0.12337700
C	-1.05773100	2.50455300	1.32894100
C	-1.49828400	1.18230700	1.40945000
C	-1.75900900	0.48923400	0.20255500
H	-1.14542700	3.01519700	-1.99480800
H	-0.85403500	3.03181800	2.25499700
C	-3.39639400	-1.35421100	-0.35308400
C	-1.78874200	-1.88087600	1.25188400
C	-3.35465400	-2.88870700	-0.32263300
H	-4.12680900	-0.95411300	0.35801700
C	-2.21905300	-3.23584100	0.67782600
H	-0.74601200	-1.83759000	1.56272600
H	-2.41784400	-1.62507100	2.10103200
H	-4.31777200	-3.29446400	-0.01360500
H	-1.39012900	-3.72549000	0.16472300
N	-2.03413300	-0.93925500	0.11785600
C	-1.70480700	0.63892300	2.81049500
H	-1.59576000	1.45011800	3.52989400
H	-0.98190300	-0.13088800	3.08832100
H	-2.70675200	0.22903700	2.94717000
C	-2.02318700	0.57773200	-2.37571500
H	-1.50172400	1.10219100	-3.17635400
H	-3.09344200	0.70121100	-2.56609000
H	-1.79318700	-0.48293400	-2.46993300
C	-0.44166900	4.62287100	0.09797000
H	0.31861300	4.81559100	0.85818500
H	-1.28340900	5.29197900	0.30290000
H	-0.03219000	4.89865900	-0.87553100
H	-2.54683900	-3.90432000	1.47353300
H	-3.13689100	-3.29022000	-1.31331200
H	-3.62037800	-0.94481900	-1.33049400
H	-0.90850300	-1.33162800	-0.70167700
C	0.28498400	-1.71049400	-1.13219900
H	0.09613700	-2.21915200	-2.07483800
H	0.52047700	-2.37879700	-0.30515600
C	1.02418200	-0.51937200	-1.18362400
C	1.24115500	0.18193600	-2.48548700
H	1.06484300	1.25502700	-2.39029900
H	0.63002500	-0.22688500	-3.28803100
H	2.29283800	0.05957900	-2.76872000
C	1.63846500	0.05311700	0.00835000
H	1.12195800	-0.25022000	0.92118400
H	1.67526700	1.14471700	-0.03214400
Si	3.47779500	-0.54150600	0.32838000
C	3.41824500	-2.39345900	0.63366000
H	4.41689400	-2.76877600	0.87571000
H	3.06976100	-2.94119400	-0.24629000
H	2.76464300	-2.64444200	1.47454200
C	4.00153700	0.40573200	1.86097500

H	5.01403700	0.11744200	2.15830300
H	3.33826000	0.20255500	2.70642300
H	4.00375200	1.48471300	1.68320800
C	4.56589200	-0.12809200	-1.14443300
H	5.61470600	-0.29571300	-0.88084500
H	4.47093400	0.91895700	-1.44571600
H	4.35076100	-0.75854400	-2.01152200

TS10

scf done:−2771.841939

C	4.19962300	1.19478600	1.02941800
C	5.24830900	0.56784600	0.35251900
C	5.26887800	-0.80198900	0.10914900
C	4.21693000	-1.55715400	0.62181900
C	3.12827500	-0.97659200	1.27486600
C	3.07785300	0.42893100	1.42113800
H	6.08766200	1.17445900	0.02534100
H	4.22840400	-2.63552200	0.49346200
C	1.35105500	2.31514200	1.51127500
C	1.81251100	0.98710400	3.48760100
C	0.87363400	3.06360400	2.76368800
H	2.06587300	2.88453100	0.92657200
C	0.66521200	1.94656700	3.79832100
H	1.62605800	-0.03091600	3.83439100
H	2.75195100	1.33150300	3.94319900
H	1.64529200	3.75694800	3.11247500
H	-0.29501900	1.45261800	3.62991100
N	1.90427200	1.02031200	2.00958100
C	2.07818100	-1.89767500	1.84624800
H	2.30134300	-2.12848900	2.89357600
H	2.05667700	-2.84675500	1.30713900
H	1.07925900	-1.47672200	1.82133200
C	4.37206300	2.66170400	1.36228200
H	5.43244000	2.87975900	1.50394800
H	3.85495300	2.94144900	2.28073300
H	4.01476400	3.31783100	0.56179700
C	6.37800100	-1.43813000	-0.68954000
H	7.33191600	-0.93120900	-0.52502100
H	6.15184900	-1.38443200	-1.75885500
H	6.50139200	-2.49277400	-0.43365500
H	0.68846200	2.30409300	4.82899300
H	-0.03339300	3.64087200	2.57485300
H	0.51711200	2.10768500	0.84274400
H	0.29030700	0.20986900	1.45834700
H	-0.46725800	0.06834600	1.31328100
C	-2.19463800	-1.22630600	0.16236400
C	-3.30411600	-1.37691500	-0.66929100
C	-2.20709300	-1.99030900	1.32596700
C	-4.36824500	-2.21861600	-0.37507800
C	-3.25739400	-2.83389300	1.66280300
C	-4.34509600	-2.94920400	0.80605900
C	-1.60331700	1.26823300	-0.56482800
C	-2.13359200	2.04083500	0.46695100
C	-1.64500300	1.84196800	-1.83432800
C	-2.60614400	3.33193200	0.27964300
C	-2.13742000	3.11831000	-2.06780200
C	-2.61279300	3.87205000	-1.00036500
C	0.25887000	-0.72513900	-1.01986500
C	1.25293500	0.14057500	-1.49965800
C	0.55941200	-2.08768700	-1.13999900
C	2.43953100	-0.29390000	-2.06564100
C	1.74222900	-2.55851000	-1.69203000
C	2.68430500	-1.65607000	-2.16192900
F	-3.04306700	4.05690200	1.31673500
F	-3.07475100	5.10644000	-1.20436200
F	-2.15766200	3.62979400	-3.30243900
F	-1.19935200	1.15151400	-2.89611700
F	-2.15398400	1.56343500	1.72940600
F	-0.28376000	-3.02517400	-0.67823300
F	1.11200600	1.47314000	-1.39407700
F	3.36498600	0.57811200	-2.47412000
F	3.83948200	-2.09599900	-2.65917600
F	2.00459900	-3.87007400	-1.72555600
F	-3.35662100	-0.70342700	-1.83634600
F	-5.40335600	-2.33391500	-1.21287800
F	-5.35741500	-3.76184100	1.11317200
F	-3.22757800	-3.53907400	2.79861300
F	-1.17646700	-1.93949100	2.19179000
B	-1.06024300	-0.20948100	-0.32197500

7. Crystallographic data

Crystallographic method

The crystal data for all compounds are compiled in the tables below. Crystals of **6a and 8e** were examined using a Bruker D8 Quest diffractometer with a Photon III detector and a microfocus source with Cu-Kα radiation (λ = 1.54178). Intensities were integrated from data recorded on 1° frames by ω or ϕ rotation. A multi-scan absorption correction method with a beam profile was applied.[23] The structures were solved using SHELXS or SHELXT;[24–25] the datasets were refined by full-matrix least-squares on reflections with $F^2 \geq 2\sigma(F^2)$ values, with anisotropic displacement parameters for all non-hydrogen atoms, and with constrained riding hydrogen geometries;[24] U_{iso}(H) was set at 1.2 (1.5 for methyl groups) times U_{eq} of the parent atom. In the case of **6a**, the structure was refined including non-spherical form factors of the individual atoms, which were calculated using quantum mechanical wave-function calculations (Tonto or ORCA); hydrogen positions were assigned without the aid of the riding model, and their displacement parameters were refined anisotropically.[26] The largest features in final difference syntheses were close to heavy atoms and were of no chemical significance. SHELX was employed through OLEX2 for structure solution and refinement.[24–25, 27] The structures have been deposited with the Cambridge Crystallographic Data Centre (CCDC 2296869-2296870). The refinements of **6a** have also been deposited with the Cambridge Crystallographic Data Centre (CCDC 2305441-2305443). This information can be obtained free of charge from www.ccdc.cam.ac.uk/data_request/cif.

Table S5: Crystal data and structure refinement for 6a and 8e.

	**6a**	**8e**
Formula	$C_{31}H_{21}BF_{15}N$	$C_{44}H_{28}BF_{15}N$
Formula Weight	703.30	866.48
Crystal Size, mm^3	0.249 × 0.219 × 0.032	0.423 × 0.347 × 0.271
Crystal System	triclinic	monoclinic
Space group	*P*-1	$P2_1/c$
a, Å	9.5183(3)	11.9657(3)
b, Å	11.9310(4)	12.7303(3)
c, Å	13.3450(4)	25.2919(7)
α, °	87.589(2)	90
β, °	85.238(2)	91.0790(10)
γ, °	72.383(2)	90
V, Å^3	1439.21(8)	3852.0(2)
Z	2	4
Temperature, K	150.0	150.0
ρ_{calc}, g cm^{-3}	1.623	1.494
μ, mm^{-1}	1.457	1.211
F(*000*)	708.0	1756.0
No. of reflections (unique)	23694 (5533)	54123 (7602)
S[a]	1.035	1.038
$R_1(wR_2)$ ($F^2 > 2\sigma(F^2)$)	0.0471 (0.1145)	0.0437 (0.1171)
R_{int}	0.056	0.024
Min./max. diff map, *e*Å^{-3}	−0.26/0.26	−0.34/0.52

[a]Conventional $R = \Sigma||Fo| - |Fc||/\Sigma|Fo|$; $Rw = [\Sigma w(Fo^2 - Fc^2)^2/\Sigma w(Fo^2)^2]^{1/2}$; $S = [\Sigma w(Fo^2 - Fc^2)^2/\text{no. data} - \text{no. params})]^{1/2}$ for all data.

Table S6: Refinements against X-ray data for 6a.

	SHELXS	Olex2.refine	Tonto	ORCA
$R_1(wR_2)$ ($F^2 > 2\sigma(F^2)$)	0.0471 (0.1145)	0.0470 (0.1259)	0.0373 (0.0958)	0.0365 (0.0923)
S	1.03	1.08	1.10	1.09
No. reflections	5533	5533	5533	5531
No. parameters	550	436	622	622
Min./max. diff map, eÅ^{-3}	−0.26/0.26	−0.38/0.36	−0.41/0.40	−0.35/0.36
H-atom treatment	isotropic	isotropic	anisotropic	anisotropic
H1···H1A^i bond distance, Å	2.390	2.436	2.21(3)	2.21(3)

Symmetry operation used to generate equivalent atom: $i = +x, 1 + y, +z$

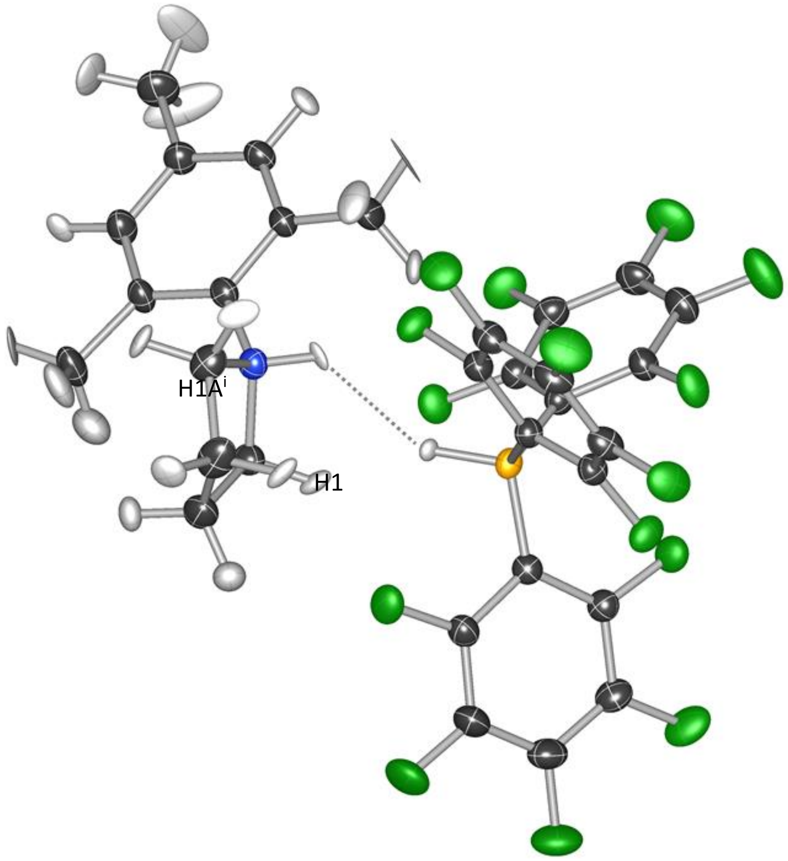

*Figure 1: Optimised structure of **6a** using quantum mechanical wave functions (ORCA), with thermal ellipsoids shown at 30% probability level. The $[HB(C_6F_5)_3]^-$ fragment has been generated by symmetry ($i = +x, 1 + y, +z$) and the analogous fragment belonging to the asymmetric unit has been omitted for clarity. Black = carbon, white = hydrogen. blue = nitrogen, yellow = boron, green = fluorine. H1···H1A^i bond distance is given in Å.*

8. References

1. Shang, M.; Chan, J. Z.; Cao, M.; Chang, Y.; Wang, Q.; Cook, B.; Torker, S.; Wasa, M. C–H Functionalization of Amines via Alkene-Derived Nucleophiles through Cooperative Action of Chiral and Achiral Lewis Acid Catalysts: Applications in Enantioselective Synthesis. *J. Am. Chem. Soc.* **2018**, *140*, 10593–10601.

2. Boga, C.; Manescalchi, F.; Savoia, D. Diastereoselective Synthesis of 2,5-Dimethylpyrrolidines and 2,6-Dimethylpiperidines by Reductive Amination of 2,5-Hexanedione and 2,6-Heptanedione with Hydride Reagents. *Tetrahedron* **1994**, *50*, 4709–4722.

3. Gonsalves, O. S.; Ambre, J. P.; Nemade, P. R. Improving the Yield of Graphene Oxide-Catalysed N-Heterocyclization of Amines through Fed Batch Mode. *New J. Chem.* **2022**, *46*, 17410–17420.

4. Fu, M.-C.; Shang, R.; Cheng, W.-M.; Fu, Y. Boron-Catalyzed N-Alkylation of Amines Using Carboxylic Acids. *Angew. Chem. Int. Ed.* **2015**, *54*, 9042–9046.

5. Wang, T.; Xu, H.; He, J.; Zhang, Y. Investigation towards the Reductive Amination of Levulinic Acid by B(C6F5)3/Hydrosilane System. *Tetrahedron* **2020**, *76*, 131394.

6. Li, G.-Q.; Li, Y.; Dai, L.-X.; You, S.-L. N-Heterocyclic Carbene Catalyzed Ring Expansion of 4-Formyl-β-Lactams: Synthesis of Succinimide Derivatives. *Org. Lett.* **2007**, *9*, 3519–3521.

7. Salamone, M.; Martella, R.; Bietti, M. Hydrogen Abstraction from Cyclic Amines by the Cumyloxyl and Benzyloxyl Radicals. The Role of Stereoelectronic Effects and of Substrate/Radical Hydrogen Bonding. *J. Org. Chem.* **2012**, *77*, 8556–8561.

8. Xu, Z.-M.; Hu, Z.; Huang, Y.; Bao, S.-J.; Niu, Z.; Lang, J.-P.; Al-Enizi, A. M.; Nafady, A.; Ma, S. Introducing Frustrated Lewis Pairs to Metal–Organic Framework for Selective Hydrogenation of N-Heterocycles. *J. Am. Chem. Soc.* **2023**, *145*, 14994–15000.

9. Azizi, N.; Khajeh-Amiri, A.; Ghafuri, H.; Bolourtchian, M.; Saidi, M. R. Iron-Catalyzed Inexpensive and Practical Synthesis of N-Substituted Pyrroles in Water. *Synlett* **2009**, *2009*, 2245–2248.

10. Devi, A.; Shallu; Sharma, M. L.; Singh, J. Paal–Knorr Pyrrole Synthesis Using Recyclable Amberlite IR 120 Acidic Resin: A Green Approach. *Synth. Commun.* **2012**, *42*, 1480–1488.

11. Rohit, K. R.; Meera, G.; Anilkumar, G. A Solvent-Free Manganese(II) -Catalyzed Clauson-Kaas Protocol for the Synthesis of N-Aryl Pyrroles under Microwave Irradiation. *J.Heterocycl. Chem.* **2022**, *59*, 194–200.

12. Manßen, M.; Kahrs, C.; Töben, I.; Bölte, J.-H.; Schmidtmann, M.; Beckhaus, R. From Five to Seven: Ring Expansion of Monoazadiene Titanium Complexes by Insertion of Aldehydes, Ketones and Nitriles. *Chem. Eur. J.* **2017**, *23*, 15827–15833.

13. Ye, C.; Chen, S.; Han, F.; Xie, X.; Ivlev, S.; Houk, K. N.; Meggers, E. Atroposelective Synthesis of Axially Chiral N-Arylpyrroles by Chiral-at-Rhodium Catalysis. *Angew. Chem. Int. Ed.* **2020**, *59*, 13552–13556.

14. Wu, C.; Lou, M.; Sun, M.; Wang, H.; Li, Z.; Qiu, J.; Wang, J.; Liu, Z. Selectively Reductive Amination of Levulinic Acid with Aryl Amines to N-Substituted Aryl Pyrroles. *Green Energy & Environment* **2023**, *8*, 438–443.

15. Maier, A. F. G.; Tussing, S.; Schneider, T.; Flörke, U.; Qu, Z.-W.; Grimme, S.; Paradies, J. Frustrated Lewis Pair Catalyzed Dehydrogenative Oxidation of Indolines and Other Heterocycles. *Angew. Chem. Int. Ed.* **2016**, *55*, 12219–12223.

16. Nomiyama, S.; Tsuchimoto, T. Metal-Free Regioselective β-Alkylation of Pyrroles with Carbonyl Compounds and Hydrosilanes: Use of a Brønsted Acid as a Catalyst. *Adv. Synth. Cat.* **2014**, *356*, 3881–3891.

17. Morozova, V.; Mayer, P.; Berionni, G. Scope and Mechanisms of Frustrated Lewis Pair Catalyzed Hydrogenation Reactions of Electron-Deficient C=C Double Bonds. *Angew. Chem. Int. Ed.* **2015**, *54*, 14508–14512.

18. Greb, L.; Oña-Burgos, P.; Schirmer, B.; Grimme, S.; Stephan, D. W.; Paradies, J. Metal-Free Catalytic Olefin Hydrogenation: Low-Temperature H2 Activation by Frustrated Lewis Pairs. *Angew. Chem. Int. Ed.* **2012**, *51*, 10164–10168.

19. Frisch, M. J.; Trucks, G. W.; Schlegel, H. B.; Scuseria, S. E.; Robb, M. A.;Cheeseman, J. R.; Scalmani, G.; Barone, V.; Petersson, G. A.; Nakatsuji, H.; Li, X.; Caricato, M.; Marenich, A. V.; Bloino, J.; Janesko, B. G.; Gomperts, R.; Mennucci, B.; Hratchian, H. P.; Ortiz, J. V.; Izmaylov, A. F.; Sonnenberg, J. L.; Williams-Young, D.; Ding, F.; Lipparini, F.; Egidi, F.; Goings, J.; Peng, B.; Petrone, A.; Henderson, T.; Ranasinghe, T.; Zakrzewski, V. G.; Gao, J.; Rega, N.; Zheng, G.; Liang, W.; Hada, M.; Ehara, M.; Toyota, K.; Fukuda, R.; Hasegawa, J.; Ishida, M.; Nakajima, T.; Honda, Y.; Kitao, O.; Nakai, H.; Vreven, T.; Throssell, K.; Montgomery, Jr., J. A.; Peralta, J. E.; Ogliaro, F.; Bearpark, M. J.; Heyd, J. J.; Brothers, E. N.; Kudin, K. N.; Staroverov, V. N.; Keith, T. A.; Kobayashi, R.; Normand, J.; Raghavachari, K.; Rendell, A. P.; Burant, J. C.; Iyengar, S. S.; Tomasi, J.; Cossi, M.; Millam, J. M.; Klene, M.; Adamo, C.; Cammi, R.; Ochterski, J. W.; Martin, R. L.; Morokuma, K.; Farkas, O.; Foresman, J. B.; Fox, D. J. Gaussian 16, Revision A.03; 2016.

20. Grimme, S. Accurate Description of van Der Waals Complexes by Density Functional Theory Including Empirical Corrections. *J Comput Chem* **2004**, *25*, 1463–1473.

21. Marenich, A. V.; Cramer, C. J.; Truhlar, D. G. Universal Solvation Model Based on Solute Electron Density and on a Continuum Model of the Solvent Defined by the Bulk Dielectric Constant and Atomic Surface Tensions. *J. Phys. Chem. B* **2009**, *113*, 6378–6396.

22. Merrick, J. P.; Moran, D.; Radom, L. An Evaluation of Harmonic Vibrational Frequency Scale Factors. *J. Phys. Chem. A* **2007**, *111*, 11683–11700.

23. Sheldrick, G. M. SADABS v2008/1 semi-empirical absorption and beam correction program; University of Göttingen: Germany, 2008.

24. Sheldrick, G. M. A Short History of SHELX. *Acta Cryst A* **2008**, *64*, 112–122.

25. Sheldrick, G. M. Crystal Structure Refinement with SHELXL. *Acta Cryst C* **2015**, *71*, 3–8.

26. Kleemiss, F.; Dolomanov, O. V.; Bodensteiner, M.; Peyerimhoff, N.; Midgley, L.; Bourhis, L. J.; Genoni, A.; Malaspina, L. A.; Jayatilaka, D.; Spencer, J. L.; White, F.; Grundkötter-Stock, B.; Steinhauer, S.; Lentz, D.; Puschmann, H.; Grabowsky, S. Accurate Crystal Structures and Chemical Properties from NoSpherA2. *Chem. Sci.* **2021**, *12*, 1675–1692.

27. Dolomanov, O. V.; Bourhis, L. J.; Gildea, R. J.; Howard, J. a. K.; Puschmann, H. OLEX2: A Complete Structure Solution, Refinement and Analysis Program. *J. Appl. Cryst.* **2009**, *42*, 339–341.

28. Sheta, A. M.; Alkayal A.; Mashaly M. A.; Said S. B.; Elmorsy S. S.; Malkov A. V.; Buckley B. R. Selective Electrosynthetic Hydrocarboxylation of α,β-Unsaturated Esters with Carbon Dioxide. *Angew. Chem. Int. Ed*. **2021**, *60*, 21832–21837.
